# Can DNA barcoding accurately discriminate megadiverse Neotropical freshwater fish fauna?

**DOI:** 10.1186/1471-2156-14-20

**Published:** 2013-03-09

**Authors:** Luiz HG Pereira, Robert Hanner, Fausto Foresti, Claudio Oliveira

**Affiliations:** 1 Laboratory of Biology and Genetic of Fish, Department of Morphology, Biosciences Institute, State University of São Paulo, São Paulo, Brazil; 2 Biodiversity Institute of Ontario and Department of Integrative Biology, University of Guelph, Guelph, ON, Canada

**Keywords:** Upper Paraná River basin, COI, Characiformes, Siluriformes, Overlooked species, Biodiversity.

## Abstract

**Background:**

The megadiverse Neotropical freshwater ichthyofauna is the richest in the world with approximately 6,000 recognized species. Interestingly, they are distributed among only 17 orders, and almost 80% of them belong to only three orders: Characiformes, Siluriformes and Perciformes. Moreover, evidence based on molecular data has shown that most of the diversification of the Neotropical ichthyofauna occurred recently. These characteristics make the taxonomy and identification of this fauna a great challenge, even when using molecular approaches. In this context, the present study aimed to test the effectiveness of the barcoding methodology (COI gene) to identify the mega diverse freshwater fish fauna from the Neotropical region. For this purpose, 254 species of fishes were analyzed from the Upper Parana River basin, an area representative of the larger Neotropical region.

**Results:**

Of the 254 species analyzed, 252 were correctly identified by their barcode sequences (99.2%). The main K2P intra- and inter-specific genetic divergence values (0.3% and 6.8%, respectively) were relatively low compared with similar values reported in the literature, reflecting the higher number of closely related species belonging to a few higher taxa and their recent radiation. Moreover, for 84 pairs of species that showed low levels of genetic divergence (<2%), application of a complementary character-based nucleotide diagnostic approach proved useful in discriminating them. Additionally, 14 species displayed high intra-specific genetic divergence (>2%), pointing to at least 23 strong candidates for new species.

**Conclusions:**

Our study is the first to examine a large number of freshwater fish species from the Neotropical area, including a large number of closely related species. The results confirmed the efficacy of the barcoding methodology to identify a recently radiated, megadiverse fauna, discriminating 99.2% of the analyzed species. The power of the barcode sequences to identify species, even with low interspecific divergence, gives us an idea of the distribution of inter-specific genetic divergence in these megadiverse fauna. The results also revealed hidden genetic divergences suggestive of reproductive isolation and putative cryptic speciation in some species (23 candidates for new species). Finally, our study constituted an important contribution to the international Barcoding of Life (iBOL.org) project, providing barcode sequences for use in identification of these species by experts and non-experts, and allowing them to be available for use in other applications.

## Background

The megadiverse Neotropical freshwater ichthyofauna is the richest in the world, with approximately 6,000 recognized species, and contributes 20-25% of the total freshwater fish fauna on Earth [1]. However, the true extent of this diversity is still unknown although it has been estimated that 30-40% of the species inhabiting this region have not been described [1,2]. Despite this exceptional richness, fishes from the Neotropical region belong to only 17 orders, a small number considering that the 954 species found in North America belong to 26 orders [3,4]. Additionally, almost 80% of this fauna belongs to only three orders: Characiformes with 1,962 species, Siluriformes with 2,214 species, and Perciformes with 572 species. Approximately half of these species belong to only three families: Characidae (tetras, piranhas, and relatives) with 1,345 species, Loricariidae (armored catfishes) with 973 species, and Cichlidae (cichlids) with 571 species [1]. Moreover, evidence based on molecular data has determined that most of the diversification of Neotropical ichthyofauna occurred recently, between 3 and 10 MYA [4-9]. All these characteristics make the taxonomic identification of this fauna a great challenge, even when using molecular approaches.

In 2003, DNA barcoding using the standardized cytochrome *c* oxidase subunit I gene (COI) was proposed by Hebert et al. as a method to identify species, [10] and since then, over 1.9 million specimens, belonging to roughly 172,000 species, have been barcoded, including 9,502 fishes (see http://www.boldsystems.org; [11]). The methodology uses a short (~650 bp), standardized DNA fragment from the mitochondrial COI gene to identify species based on differences in their COI sequences [10]. The effectiveness of the barcoding system has been repeatedly demonstrated by the identification of marine [12-22] and freshwater fish species [23-32], with a success rate of well over 90%. However, many fish barcoding surveys have been conducted in relatively species-poor areas, with species belonging to several higher taxa, or relatively rich areas where only a few species were analyzed.

The Upper Parana River basin drains an area of approximately 891,000 km^2^ of Atlantic Rain Forest and South America Savanna (Cerrado) in the most urbanized and exploited area of Brazil. The last inventory of freshwater fish fauna found 310 valid species and 50 other putative species yet to be described [33], of which 80% corresponded to Characiformes and Siluriformes. Although this basin is considered the most well-studied in the Neotropical region, the number of species in the entire region remains uncertain, and new fish species are discovered after each new inventory [33-37]. The aim of the present study was to test the hypothesis that the DNA barcoding methodology can be used effectively to identify the megadiverse freshwater fish fauna of the Upper Parana River basin, a representative area of the Neotropical region. With this purpose, we analyzed 254 species (nearly 70% of the species that occur in this basin) including many congeneric species and closely related genera.

## Results

We obtained barcode sequences for 1,244 specimens belonging to 221 nominal species and 33 species identified only at the genus level, representing 127 genera, 36 families and 10 orders (Table 1/Additional file 1). The number of specimens analyzed ranged from 1 to 56 per species (mean = 4.9) (Additional file 1). The number of genera and families represented by multiple species (>2) were 20 and 19, respectively (Additional file 1; Table 1). A total of 99.7% of the amplified sequences were larger than 500 bp (mean = 647 bp), and no stop codons, deletions or insertions were observed. Four hundred and thirty two nucleotide sites were variable, and most substitutions occurred in the third nucleotide position of the codons (59.9%, 259 sites).

**Table 1:** Summary of fish taxa included in this study

**Order**	**Family**	**Number of genera**	**Number of species**
Characiformes	Acestrorhynchidae	1	1
	Anostomidae	3	13
	Bryconidae	2	4
	Characidae	25	48
	Crenuchidae	1	7
	Curimatidae	2	5
	Cynodontidae	1	1
	Erythrinidae	3	4
	Lebiasinidae	1	1
	Parodontidae	2	4
	Prochilodontidae	1	1
	Serrasalmidae	3	4
	Triportheidae	1	1
Siluriformes	Auchenipteridae	3	3
	Callichthyidae	6	11
	Cetopsidae	1	1
	Clariidae	1	1
	Doradidae	2	2
	Heptapteridae	8	12
	Loricariidae	20	59
	Pimelodidae	6	7
	Pseudopimelodidae	2	3
	Trichomycteridae	3	16
Perciformes	Cichlidae	10	17
	Sciaenidae	1	1
Gymnotiformes	Gymnotidae	1	5
	Hypopomidae	1	1
	Rhamphichthyidae	2	2
	Sternopygidae	2	3
Rajiformes	Potamotrygonidae	1	2
Pleuronectiformes	Achiridae	1	1
Cyprinodontiformes	Poeciliidae	3	5
	Rivulidae	2	2
Clupeiformes	Clupeidae	1	1
Cypriniformes	Cyprinidae	3	4
Synbranchiformes	Synbranchidae	1	1
	**Total**	**127**	**254**

**Figure 1:**
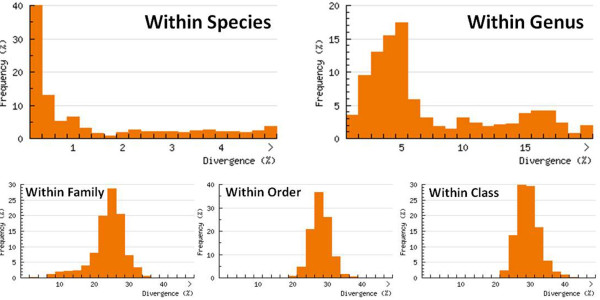
**K2P genetic divergence.** Distribution of K2P genetic divergence at the different taxonomic levels.

Most species have a unique haplotype, or a tight cluster of haplotypes, which allowed the correct discrimination of 99.2% of analyzed species (252 of 254) (Additional file 2). Only one pair of species (*Astyanax schubarti* X *A. trierythropterus*) shared their haplotypes and could not be discriminated. The mean Kimura-2-Parameter (K2P) genetic divergence ranged from 0% to 8.5% (mean = 1.3%) for intra-specific comparisons and from 0% to 24.9% (mean = 6.8%) for congeneric comparisons (Table 2), establishing a barcode gap of about five times between congeneric and intra-specific variation. The mean K2P values of genetic divergence to families, orders and classes are shown in Table 2, with increasing K2P divergence values being associated with increasing taxonomic rank. The analyses of the distribution of K2P divergence values showed that 74.5% of the intra-specific comparisons were less than 2%; however, 12.6% of the divergence values between congeners were also less than 2% (Figure 1).

**Table 2:** Summary of K2P genetic divergence within different taxonomic levels from 1,244 analyzed specimens

**Category**	**Taxa**	**Min dist(%)**	**Mean dist(%)**	**Max dist(%)**	**SE dist(%)**
Within species	224	0	1.3 (0.3*)	8.5	0.02
Within genus	122	0	6.8	24.8	0.05
Within family	36	1.4	20.1	31.5	0.01
Within order	10	15.2	23.3	33.4	0.00
Within class	2	16.8	24.5	38.1	0.00

* mean distance when considering each subcluster as an independent genetic unit in the analysis.

**Figure 2:**
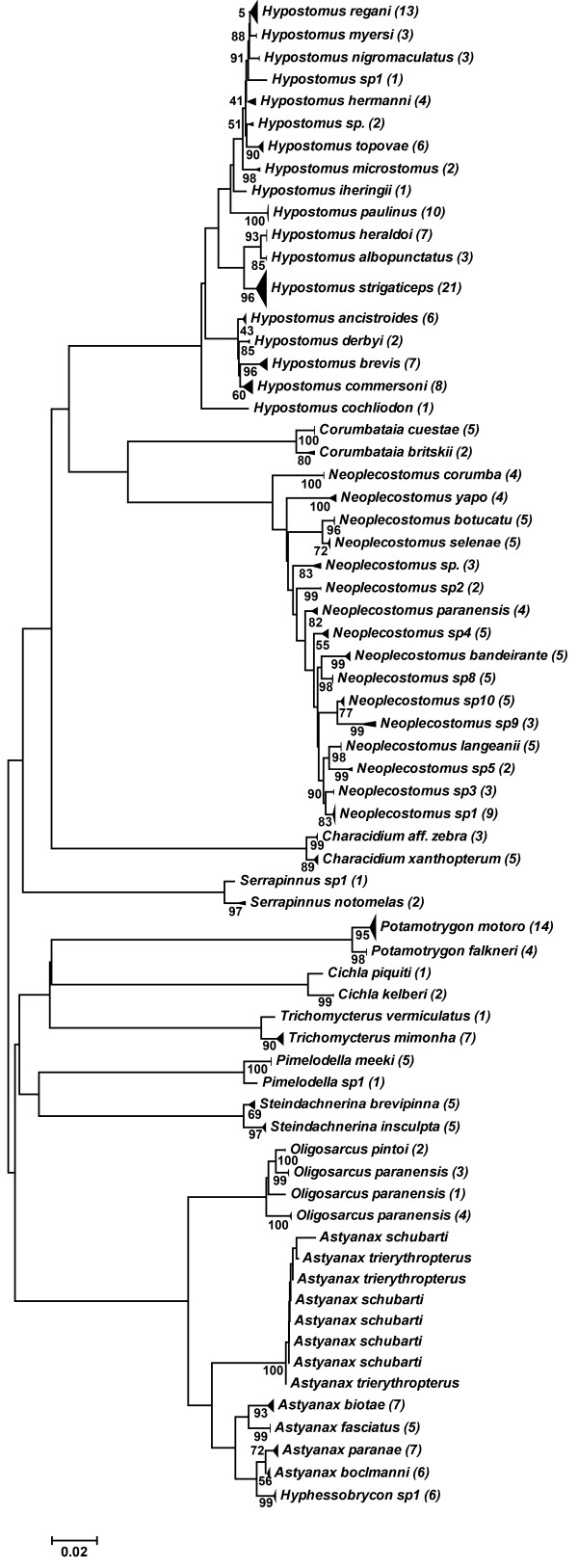
**NJ dendrogram of the pairs of species with low divergence.** NJ dendrogram showing the correct discrimination by distance genetic approach of the pairs of species that displayed K2P divergence values of below 2%. Node values = bootstrap test (1,000 pseudo replicas); values between brackets = number of specimens analyzed.

The nearest neighbor distance (NND) analysis, which determines the lowest distance between a pair of species, found 84 pairs of species (representing 53 species, 20.9% of species analyzed) with K2P divergence values of less than 2% (a threshold value adopted as a “start point” to delimit species in our analysis) (Additional file 3). However, these values still allowed for discrimination between the species, which formed cohesive groups with exclusive haplotypes (Figure 2). Again, just one pair of species shared their haplotypes and could not be discriminated (*A. schubarti* X *A. trierythropterus*) (Figure 2/Additional file 2). In addition, to reinforce the utility of the DNA barcoding technique to identify species, even those with low K2P divergence values (<2%), we applied the nucleotide diagnostic (ND) approach [38] as a complementary methodology of analysis. We identified only the exclusive NDs to that simple pair of species, which ranged from 2 to 11, allowing the discrimination of species (Additional file 4). The genera *Neoplecostomus* and *Hypostomus* showed multiple pairs of species with low K2P genetic divergence values. Thus, all species of each genus were analyzed together to determine the NDs of each species. The 16 species of *Neoplecostomus* showed from 0 to 8 exclusive NDs (Additional file 4) in 45 informative sites. Just one species (*N. sp. 10*) showed no exclusive ND, but it could be easily discriminated by its unique combination of the 45 informative nucleotide positions (Additional file 4). The 18 *Hypostomus* species analyzed showed from 0 to 7 exclusive NDs in 32 informative sites, but five species showed no exclusive NDs (Additional file 4). Therefore, we also included partial NDs in the analysis, which resulted in 38 more informative sites. In total, 70 sites were informative in allowing the discrimination of all analyzed *Hypostomus* by their exclusive combination of characters (Additional file 4).

**Figure 3:**
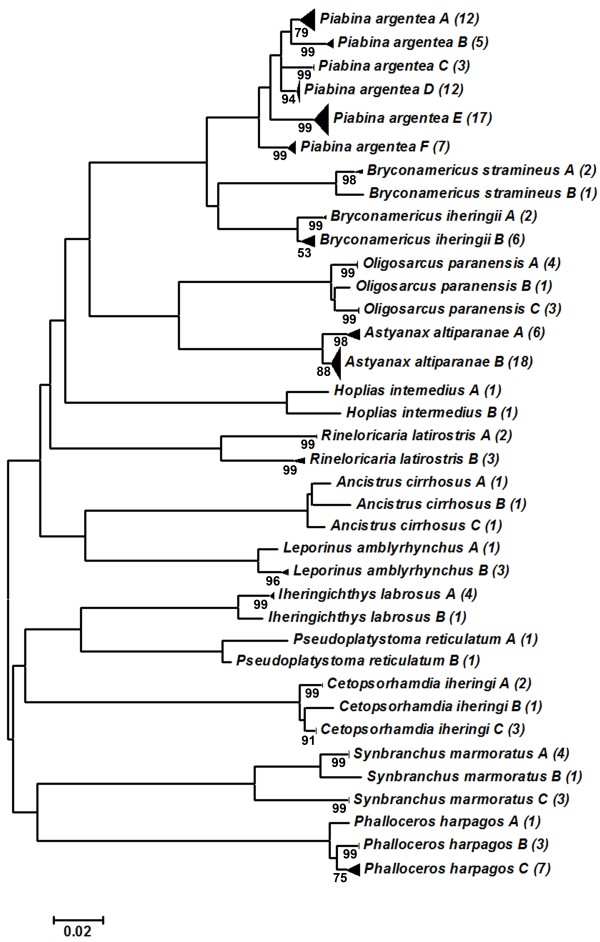
**NJ dendrograma of the species with deep intra-specific divergence.** NJ dendrogram of the 14 species that showed deep intra-specific genetic distance. Node values = bootstrap test (1,000 pseudo replicas); values between brackets = number of specimens analyzed.

In contrast, 14 species (5.5%) exhibited intra-specific K2P distances that exceeded 2% (Table 3), splitting into 2 to 6 subclusters in the Neighbor-Joining (NJ) dendrogram (Figure 3). The K2P genetic distance among the subclusters ranged from 1.4% to 8% against mean values of 0% to 1% into each subcluster (Table 3). These cases suggest the existence of overlooked species or morphological misidentification and are responsible for the high value of the average intra-specific K2P genetic divergence obtained in all species (Table 2). When each subcluster was considered as an independent genetic unit in the analysis, the global mean of the intra-specific K2P genetic divergence was only 0.3% and the barcode gap increase to 22 times among congeneric (mean value = 6.8%) and intra-specific comparisons.

**Table 3:** Species with high intra-specific K2P divergences values

**Species**	**Intra-specific divergences (%)**			**Number of subclusters**	**Inter-subclusters divergences (%)**	**Intra-subclusters divergences (%)**
	**min**	**mean**	**max**			
Ancistrus cirrhosus*	1.7	2.2	2.5	3	1.7 to 2.5	-
Astyanax altiparanae*	0	1.3	2.9	2	2.6	0.4 to 0.5
Bryconamericus iheringii***	0	1.2	2.2	2	1.8	0.2 to 0.8
Bryconamericus stramineus***	0.6	1.7	2.4	2	2.2	0.6
Cetopsorhamdia iheringi*	0	1.2	2.5	3	1.4 to 2.5	0
Hoplias intermedius*	-	4.0	-	2	4.0	-
Iheringichthys labrosus*	0	1.2	2.6	2	2.5	0.3
Leporinus amblyrhynchus***	0.5	1.3	2.2	2	2.1	0.6
Oligosarcus paranensis***	0	1.4	2.3	3	1.6 to 2.1	0.1
Phalloceros harpagus**	0	1.2	2.2	3	1.7 to 2.2	0 to 0.5
Piabina argentea**	0	3.0	6.3	6	1.9 to 5.6	0 to 1.0
Pseudoplatystoma reticulatum**	-	3.1	-	2	3.1	-
Rineloricaria latirostris**	0	4.6	7.3	2	7.3	0 to 0.6
Synbranchus marmoratus*	0	4.7	8.5	3	2.9 to 8.0	0

* = unique species of the genus/group present in the Upper Parana River basin; ** = species belonging to a genus in which all species belonging to the Upper Parana River basin were collected; *** = species belonging to a genus with multiple species reported in the Upper Parana River basin but in which one species was not sampled.

## Discussion

Our survey is the first to examine a large number of fish species from the Neotropical region, including a high number of genera represented by multiple species where low values of inter-specific genetic distance are expected, which could present barriers to species identification (Additional file 1). The barcoding methodology was very effective in allowing the correct discrimination of 252 out of 254 analyzed species (99.2%) when using both the genetic distance and ND approaches demonstrating the existence of a barcode gap for the species analyzed (~5X), and confirming its utility as a powerful tool for species identification. The mean K2P distance values found for conspecific and congeneric comparisons (1.3% and 6.8%, respectively) were somewhat discordant from those found in the literature, which ranged from 0.1% to 0.8% and from 8% to 17.3%, respectively, in 14 out of 21 surveys cited (Table 4). The lower observed mean value of congeneric divergences reflected the large number of closely related species that were analyzed. As previously mentioned, the megadiverse Neotropical ichthyofauna is represented by relatively few higher taxa (only 17 orders), and approximately 80% of its species (~4,500) belong to only three orders (Characiformes, Siluriformes and Perciformes) [1]. These groups comprise some of the most specious families of freshwater fishes in the world [39], and thus include a large number of closely related species. In comparison, in other barcoding fish surveys, even those that analyzed a relatively large number of species, the analysis represented a few related species (same genus) of a relative large number of higher taxa (Table 4), in which the relatively higher conspecific values reflected deeper divergences among these lineages. Carvalho et al. [27] and Pereira et al. [28] studied the fish fauna from the São Francisco and Paraíba do Sul river basins, both in the Neotropical region, and found similar mean values of congeneric K2P genetic distances of approximately 10%, consistent with values shown in the other cited surveys (Table 4). However, these mean values can be due to the lower number of species analyzed (101 and 58, respectively), which also included a few genera represented by two or more species (Table 4). Thus, we believe that with an increased number of species per genus, the mean values of congeneric K2P distance genetic will tend to decrease. In contrast, Rosso et al. [30] analyzing Neotropical freshwater fishes from Pampa plain in Argentina found congeneric K2P genetic distances of only 1.68% (Table 4), but this values reflect the low number of congeneric species analyzed (only three genera with two or more species), which represent species with taxonomic problems [30]. When the authors considered the comparison among all species the main K2P genetic distance value is 13.6%.

**Table 4:** Summary of the DNA barcoding surveys of fishes (by December 2012) highlighting the numbers of species, higher taxa, families and genera with multiple species analyzed

**Survey**	**Region**	**Number of species analyzed**	**Number of high taxa (order)**	**Number of families and families with multiple species (>2)**	**Number of genera and genera with multiple species (>2)**	**Mean value of K2P divergence of conspecific/congeneric comparisons (%)**	**Reference**
Freshwater fishes							
Upper Paraná River basin	Neotropical	254	10	36/20	126/19	1.30/6.80	Present study
Paraíba do Sul River basin – Brazil	Neotropical	58	5	17/8	40/4	0.13/10.36	[28]
São Francisco River basin – Brazil	Neotropical	101	6	22/11	75/6	0.50/10.61	[27]
Canada	North America	190	20	28/15	85/21	0.27/8.37	[23]
Mexico and Guatemala	Central/North America	61	8	15/5	36/6	0.45/5.10	[24]
Cuba	Central America	27	8	10/4	17/2	0.40/8.00	[25]
Tall Lake - Philippines	Asia	23	9	17/2	21/2	0.60/11.07	[26]
North America	North America	752	24	50/18	178/45	0.73/13.67	[29]
Argentina	Neotropical	36	8	18/3	32/1	0.33/1.68	[30]
India	Asia	25	1	9/4	17/2	-	[31]
Mexico	North America	31	4	8/3	16/4	0.78/6.08*	[32]
Marine fishes							
Australia	Oceania	207	14	50/23	113/18	0.39/9.93	[12]
Argentina	South America	125	25	63/9	98/3	0.23/4.04	[13]
Nayband National Park – Iran	Europe	76	11	32/8	56/5	0.18/12.00	[16]
India	Asia	115	7	37/14	79/5	0.30/6.60	[14]
China	Asia	121	15	55/17	85/9	0.15/16.49	[15]
China	Asia	95	15	69/13	86/2	0.32/17.26	[17]
China	Asia	242	23	82/20	162/17	0.18/13.55	[18]
Caribbean	Caribbean	572 (521*)	20*	87/47*	232/39*	0.45/16.30*	[19]
Canada	North America	177	28	81/20	136/9	0.32/4.40	[20]
Korea	Asia	68	14	49/4	62/1	0.41/3.21	[21]
Brazil	South America	135	22	62/12	110/5	0.31/13.29	[22]

* Values based on available data on BOLD.

In addition, the low observed congeneric K2P divergence values reflect a possible recent radiation of Neotropical freshwater fishes, compared to other freshwater fish faunas [4-9]. For example, Montoya-Burgos et al. [8], studying *Hypostomus,* and Hubert et al. [9], working with *Serrasalmus* and *Pygocentrus* from South America, proposed the hypothesis of recent radiation of these species (originating between 2 and 12 million years ago) and suggested that these patterns could apply to other Neotropical fish groups. In fact, our results showed that approximately 60% of congeneric comparisons from the neotropics are less than 5% divergent, contrasting with the values found among genera (mean value = 20%) (Figure 1), reinforcing this hypothesis. In summary, the large number of species associated with the recent radiation of the Neotropical ichthyofauna, could potentially pose a barrier to the use of barcoding (due the possible shared haplotypes). However, the barcoding methodology was able to correctly discriminate species in this megadiverse fauna.

On the other end of the spectrum, the high observed global conspecific mean value can be explained by the 14 species that displayed deep intra-specific divergence, which ranged from 1.4% to 8.0% among their subclusters (Table 3). The NND analyses confirm this observation, showing that 92.5% of species analyzed display intraspecific mean values of 0.36%. In addition, we reanalyzed the species using the subclusters formed by those 14 species as an independent genetic unit. This method resulted in a global conspecific value of 0.3%, consistent with the literature cited.

### Low values of interspecific genetic divergence (<2%)

Generally, barcoding researchers have used a 2% divergence threshold as a heuristic cutoff value for species delimitation [13,23,27,28,40-42]. This limit is based on the distribution of intra and inter-specific K2P genetic distance values in the approximately 172,000 species that have been barcoded (http://www.boldsystems.org). However, in a review of the available literature, almost all barcoding surveys reported cases of inter-specific comparisons with low values, even below the generally accepted limit, but were still able to correctly discriminate between most species [12,23-25,28]. Thus, this value must be used only as a starting point to investigate divergence among specimens, and other characteristics of the species/group studied, such as their evolutionary history, should be considered before defining a species limit [42]. Notably, AFLP (amplified fragment length polymorphism) genome scans of several closely related pairs of taxa from North American waters showed that taxa with divergences of > 2% rarely exhibited evidence of introgressive hybridization in their contact zones [43].

Using 2% as cutoff for delimiting species, 84 pairs of taxa (representing 53 species) showed inter-specific values below this limit and could not be discriminated using this simple divergence threshold (Additional file 3). However, in 83 cases, each species displayed a cohesive cluster of haplotypes, allowing its discrimination (Figure 2), showing a barcode gap ranging from 1.2 to 18 times between congeneric and intra-specific comparison, and thereby reinforcing the idea that the 2% cutoff value could be considered as a reasonable starting point. Only *A. schubarti* and *A. trierythropterus* shared haplotypes and could not be discriminated, but there are morphological evidences that these two species represent only one valid species (*A. schubarti*) (Dr. Ricardo M. Correa e Castro, personal communication). Possible explanations for the observed low inter-specific K2P genetic divergence values include the recent radiation of some groups of species [41], as discussed above, and the possible evolutionary rate variation of COI among different taxa [44,45]. The 53 species studied here represent 12 genera of freshwater fishes, including some of the most specious Neotropical groups (*Astyanax*, *Characidium*, *Pimelodella, Hypostomus* and *Trichomycterus)*[39], and 27 of the 53 species belong to only two genera (*Hypostomus* and *Neoplecostomus*) (Additional file 3). In the case of *Hypostomus*, Montoya-Burgos et al. [8] analyzed the species of this genus and suggested that the processes of divergence and radiation date back to between 4 and 12 million years ago, corroborating the observed low inter-specific values. This result can also explain the difficulties in the identification of these species, even with morphological approaches [46-49]. However, all 18 *Hypostomus* species could be discriminated using both genetic distance (Figure 2) and ND (Additional file 4) approaches. The same pattern was observed in *Neoplecostomus*. Roxo et al. [50] estimated that radiation of the *Neoplecostomus* genus occurred between 1 and 10 million years ago and that their speciation process involved mainly recent headwater capture events. As with *Hypostomus*, the 16 *Neoplecostomus* species were correctly discriminated using both barcoding approaches (genetic distance (Figure 2) and ND (Additional file 4)).

A survey of the evolution of *Astyanax* in the Mesoamerica region found a recent radiation of its species, with 90% of interspecific comparisons ranging from 1% to 5% [51]. Other surveys examining Neotropical freshwater fish fauna provide similar results [9,52]. In addition, our results showed that approximately 60% of congeneric comparisons in *Astyanax* were below 5% and had a global mean value of interspecific K2P genetic distances of 6.8%, relatively smaller than the values cited in the literature. Thus, we conclude that the most likely explanation for the low interspecific values observed in this study is a possible recent radiation process in the megadiverse Neotropical ichthyofauna.

In addition, we used the ND approach as a complement to the distance approach (Additional file 4) and found NDs to all species analyzed using both kinds of characters (only exclusive NDs or combined exclusive and partial shared NDs). The use of the ND approach can be useful in cases with low divergence values to more accurately identify species. Some surveys have used the ND approach as an alternative to the distance approach with a high level of success [29,53-56]. Some authors even advocate the use of the ND approach, as it follows the same principles used in traditional morphological approaches, that is, the use of diagnostic characters [55,57,58]. Based on our experience, we suggest that as a first step, researchers use the fastest genetic distance approach to assign the species to a related group and then apply the ND approach to test the identification within the context of a genus, which simplifies the ND analysis by restricting the number of taxa which must be compared.

### Deep intra-specific divergence (>2%)

The applicability of DNA barcoding to reveal cryptic and potentially new species, has increased our knowledge regarding biodiversity in many taxa and the use of barcoding as a tool for these purposes is becoming a reality, [28,29,59-72]. In the present study, 14 species (5.5%) showed deep intra-specific genetic divergence values (≥ 2%) and were further subdivided into two or more subclusters (Figure 3). Each subcluster showed a tight cluster of haplotypes with significantly higher mean values (1.4% to 8%) among them than the mean values observed within each subcluster (0 to 1%) (Table 3). Deep intra-specific divergence has been reported in barcoding analyses of the most diverse groups, many of which were considered cryptic species [28,29,60-71]. Another, non-exclusive explanation for the high intra-specific genetic divergence is the possibility of these subclusters to represent species not sampled, as most cases represent species that are difficult to identify. However, six of the 14 species reported here represent unique species from the genus/group present in the Upper Parana River basin (Table 3), four other species belong to a genus representing all species that occur in this hydrographic basin, and only four species belong to a genus with multiple reported species in this basin in which one species is not sampled (Table 3). In the last case, the specimens analyzed were morphologically reviewed but could not be assigned to the other unsampled species of the genus. We conclude that all these cases represent cryptic species and are strong candidates for new species.

To reinforce our hypothesis, six of these 14 species are strongly suggested in the literature as species complexes by cytogenetic markers (*Ancistrus cirrhosus*[73]; *Iheringichthys labrosus*[74]; *Synbranchus marmoratus*[75]; *Astyanax altiparanae*[76]; *Hoplias intermedius*[77]; *Piabina argentea*[78]). Furthermore, the intra-specific lineages of 10 species are allopatric, reinforcing the fact that such lineages have independent evolutionary histories. The presence of different haplotype lineages may be explained by possible restricted gene flow due to the fragmented nature of freshwater ecosystems, which can include many physical and chemical barriers [29,41,42]. To complete this scenario, some authors suggest that the freshwater fishes have a limited dispersal capability, especially among those belonging to small-sized groups, which restricts their geographical distribution and may facilitate geographical population subdivision, thereby enabling the possible creation of new species by geographic isolation (allopatry) [29,79,80]. In summary, our results identified at least 23 strong candidates as potential new species including extreme cases, such as *P. argentea,* which subdivided into six subclusters and *S. marmoratus,* which showed the highest value of genetic divergence among subclusters (8%), and exhibited mean values higher than those observed among inter-specific analyses (Table 3). These results reinforce the use of DNA barcoding as a powerful tool to reveal overlooked species, especially among specious and/or problematic groups.

### Intra X Inter-specific distance genetic

The success of the species identification by DNA barcoding is based on the difference between the intra- and inter-specific genetic divergences, the so-called barcoding gap [10,81-83]. In this survey, beside the relatively low values of the inter-specific genetic divergence observed (6.8% - Table 2), the barcoding gap was clear. The overall variation was about five and 22 times greater among congeneric species than within species considering respectively, a mean value of intra-specific K2P genetic divergence of 1.3% (without correction to species with high intra-specific divergence genetic) and 0.3% (considering each subcluster of the species with high intra-specific genetic divergence as a different unit). Even for the 83 cases that displayed low inter-specific divergence genetic values (<2% - Additional file 3), the barcoding gap was clear, with values raging from 1.2 to 18 times (mean = 6 times) greater among congeneric than within species. Thus, our results demonstrate the existence of barcoding gap even in those cases where the inter-specific genetic divergence is low.

The use of 2% divergence threshold as a heuristic cutoff value for species delimitation showed to be useful to analyzing the Neotropical freshwater species since almost 80% of inter-specific comparisons (Figure 1) displayed more than 2% of genetic divergence concordant with the most barcoding surveys (Table 4). On the other hand, the 83 cases of low values of inter-specific K2P genetic divergence confirm the mentioned above that the 2% as a cutoff value should be used only as a start point to delimit species [42]. Thus, we suggest that for these cases, a new cutoff value should be calculated based on the values of genetic divergence observed within the genus. For example, the Table 5 show the 18 genera represented by multiple species (>2) in our survey with the minimum, average and maximum inter-specific genetic divergence values such as the range of the maximum intra-specific genetic divergence value found in each species. For the 13 out of 18 genera, the minimum value of congeneric genetic divergence is several times greater the maximum intra-specific genetic divergence observed among their species. In these cases, the 2% of cutoff value should be enough to discriminate the species. However, for five genera (*Astyanax*, *Characidium*, *Neoplecostomus*, *Hypostomus* and *Trichomycterus*), we observed that maximum intra-specific genetic divergence value exceed the minimum congeneric value observed. These cases should be analyzed carefully to delimit species, because the use of a 2% of cutoff values can hide the real diversity of the group. But, the barcode sequences can only flag these cases and, a more accurately analysis should be conducted by specialist in each group based on an integrative taxonomy. Thus we believe that with the increase of species analyzed per genus should be possible calibrate the barcoding methodology to each group and probably facilitate the discovery of an unsuspected hidden diversity.

**Table 5:** Inter- and intra- specific genetic divergence values of the genera represented by >2 species.

		**Inters-specific genetic divergence**			**Max intra-specific genetic divergence (%)**
***Genera***	**Species number**	**Min (%)**	**Mean (%)**	**Max (%)**	
*Apareiodon*	3	7.8	8.5	10.4	0.9
*Astyanax*	7	0.3	11.6	18.7	0.2-1.9
*Bryconamericus*	3	6.49	10.0	11.1	0.6-1.2
*Characidium*	7	0.9	13.9	18.7	0-0.9
*cichla*	4	1.8	11.3	15.1	-
*Corydoras*	6	4.0	11.4	17.1	0-1.6
*Crenicichla*	3	2.1	9.4	23.2	0.8
*Cyphocharax*	3	2.6	9.7	13.6	0.2-1.6
*Gymnotus*	5	2.3	9.7	17.6	0.3-1.1
*Hisonotus*	4	4.8	14.4	20.1	0.2-1.2
*Hyphessobrycon*	6	2.7	17.6	23.3	0-0.3
*Hypostomus*	18	0.6	3.7	7.0	0-1.4
*Leporinus*	10	3.3	12.0	18.0	0-0.9
*Moenkhausia*	3	17.3	19.4	24.8	0-0.2
*Neoplecostomus*	16	0.7	2.7	6.4	0-1.4
*Pimelodella*	3	1.8	9.3	11.4	0-0.2
*Serrapinnus*	4	1.3	6.1	10.4	-
*Trichomycterus*	14	1.2	8.1	16.9	0-1.2

## Conclusions

Our study is the first to examine a large number of freshwater fish species from the Neotropical area. The results confirm the efficacy of using barcoding methodology to help calibrate our traditional knowledge of species diversity and to enhance our ability to identify this megadiverse fauna. Barcoding discriminated 99.2% of the analyzed species, agreeing with morphological taxonomic analysis, and our study represents an important contribution to the global barcoding library. Our study is the first to include many genera represented by multiple species, which may be why our distribution of inter-specific genetic divergence of the megadiverse Neotropical ichthyofauna is smaller than those of other surveys. This finding most likely indicates recent radiation of this fauna and reflects a high number of closely related species. Moreover, this study also showed the power of using barcode sequences to identify species with low inter-specific divergence using only the divergence genetic approach or in conjunction with the ND approach. The results also revealed cryptic speciation in some species (23 candidates for new species), which is a relevant finding considering that the Upper Parana River basin is the most well studied basin in the Neotropical area, suggesting that the number of overlooked species in the overarching Neotropical area may be large yet manageably revealed using barcoding methodology.

Finally, our study makes an important contribution to the knowledge of the rich ichthyofauna of the Upper Parana River basin, and contributes significantly to the FISH-BOL campaign (and the international Barcoding of Life project of which it is a part) by providing barcode sequence profiles for use in the identification of these species by experts and non-experts alike, and by making them available for use in other applications.

## Methods

### Specimen collection

**Figure 4:**
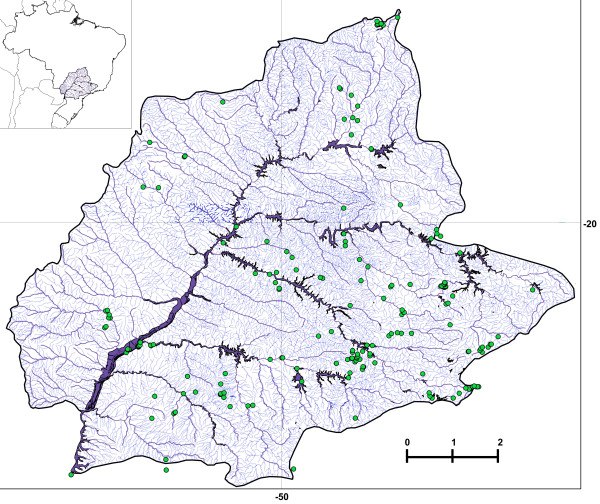
**Sample sites map.** Map of the Upper Parana River Basin showing the 208 sample sites where the 1,244 specimens were obtained. Scale bar = 200 km. Points that appear to be outside of the map represent transition zones between hydrographic basins.

A total of 1,244 fishes were collected at 208 different sites along the Upper Parana River basin (Figure 4). All fishes collected for this study were collected in accordance with Brazilian laws under a permanent scientific collection license in the name of Dr. Claudio Oliveira. Additionally, our laboratory has special federal permission to keep animals and tissues from a public collection in our care. After collection, animals were anesthetized by immersion in 1% benzocaine in water and either preserved in 95% ethanol for molecular studies or fixed in 10% formaldehyde for morphological studies. Morphological vouchers were deposited in the fish collection of the Laboratory of Biology and Genetic of Fish (LBP), Department of Morphology, Biosciences Institute, UNESP, Botucatu, São Paulo, Brazil. Specimen data, including geospatial coordinates of collection sites and other relevant details, are recorded in the publicly accessible BOLD project titled “Fishes from Upper Paraná River, Brazil” (project code: FUPR).

### Extraction, PCR amplification, and sequencing

DNA barcoding was carried out at the Canadian Centre for DNA Barcoding (CCDB), Canada and at the Laboratory of Biology and Genetic of Fish (LBP), UNESP, Botucatu, Brazil. Total genomic DNA was isolated from the fin or muscle tissue of each specimen using one of two different methods: with a DNeasy Tissue Kit (Qiagen), according to the manufacturer’s instructions (LBP); or with vertebrate lysis buffer containing proteinase K digested overnight at 56°C and subsequent extraction using a membrane-based approach on a Biomek NX (http://www.pall.com) liquid handling station using AcroPrep96 (http://www.beckman.com) and 1 ml filter plates with 10 mm PALL glass fiber media [84] according to the CCDB protocol. A segment of approximately 648 bp from the 5’ end of the mitochondrial cytochrome *c* oxidase subunit I (COI) gene was amplified by polymerase chain reaction (PCR) using different combinations of primers: FishF1, FishR1, FishF2, FishR2 [12], the M13-tailed primer cocktails C_FishF1t1 – C_FishR1t1 and C_VF1LFt1 – C_VR1LRt1 [85], and the pair L5698-Asn [86] and H7271-Coi [59]. The 12.5 μl polymerase chain reaction (PCR) mixtures included 6.25 μl 10% trehalose, 2.0 μl ultrapure water, 1.25 μl 10X PCR buffer, 0.625 μl MgCl_2_ (50.0 mM), 0.125 μl of each primer (0.01 mM), 0.0625 μl of each dNTP (0.05 mM), 0.625 μl Taq polymerase and 2.0 μl of DNA template. PCR was carried out in a thermocycler (Veriti® 96-Well Thermal Cycler, Applied Biosystems). The thermocycler conditions followed the protocols of Hajibabaei et al. [87]. Amplified products were verified on 1% agarose gels. At the LBP, the PCR products were purified with ExoSap-IT® (USB Corporation) following the manufacturer’s protocol. At the CCDB, PCR products were labeled with the BigDye Terminator v.3.1 Cycle Sequencing Ready Reaction kit (Applied Biosystems) using standard methods [87] and were bidirectionally sequenced using an ABI3730 capillary sequencer. At the LBP, the cycle sequencing reaction was carried out using a BigDye^TM^ Terminator v.3.1 Cycle Sequencing Ready Reaction kit (Applied Biosystems) in a final volume of 7.0 μl containing 1.4 μl of template, 0.35 μl of primer (10 μM), 1.05 μl of buffer 5X, 0.7 μl of BigDye mix and water. The cycle sequencing conditions included initial denaturation at 96°C for 2 min followed by 35 cycles of denaturation at 96°C for 45 s, annealing at 50°C for 60 s, and extension at 60°C for 4 min. The PCR sequencing products were purified with EDTA/sodium acetate/alcohol following the protocol suggested in the BigDye™ Terminator v.3.1 Cycle Sequencing kit’s manual (Applied Biosystems). All samples were sequenced on an ABI3130 Genetic Analyzer capillary sequencer (Applied Biosystems) following the manufacturer’s instructions. Sequence data, trace files, primer details, and collection localities for specimens are available within the project FUPR on BOLD (http://v3.boldsystems.org/). Sequences have also been deposited in GenBank (Accession numbers in Additional file 1).

### Data analysis

All sequences were analyzed using SeqScape® software v2.6 (Applied Biosystems) to obtain consensus sequences and check the occurrence of deletions, insertions, and stop codons. The sequences were aligned using tools available on BOLD v 3.0 (http://v3.boldsystems.org/). The genetic distances among and within species were calculated using the Kimura-2-Parameter (K2P) distance model [88]. A neighbor-joining (NJ) dendrogram of K2P distances was created using MEGA v 5.0 software to provide a graphic representation of the patterning of divergence among species [89].

The nucleotide diagnostic (ND) approach [38] was carried out using only the exclusive nucleotide diagnostics for the single pair of species or the exclusive nucleotide diagnostics associated with partial shared nucleotide diagnostics for the genus that showed more than two species with low divergence values. The second option was used to provide a great number of informative nucleotide diagnostics, as a large number of species were analyzed. The nucleotide diagnostics were obtained using the BOLD v 3.0 tool (http://v3.boldsystems.org/) and were manually checked using the BioEdit Sequence Alignment Editor v 7.0.5.3 [90].

The species delimitation was initially carried out using 2% divergence as a cutoff value, as employed in others barcoding surveys [13,23,27,28,40-42]. The analysis of the possible cryptic speciation was applied to all species that showed at least one individual that displayed >2% divergence to others specimens.

## Abbreviations

AFLP: Amplified fragment length polymorphism; BOLD: Barcode of life data systems; Bp: Base pair; CCDB: Canadian centre for DNA barcoding; COI: Citocromo c oxidase subunit I; FURP: Fishes from upper parana project; K2P: Kimura-2-parameter; LBP: Laboratory of biology and genetic of fish; MYA: Milion yaers ago; ND: Nucleotide diagnostic; NJ: Nieghbor-joining; NND: Nearest neighbor distance

## Competing interests

The authors declare that they have no competing interests.

## Authors’ contributions

LHGP and CO participated equally in the design of the study. LHGP did most of the laboratory experiments. RH contributed acquisition of part of the sequences and the revision of the manuscript. LHGP and CO analyzed the data. LHGP, CO, RH, and FF discussed the results. LHGP and CO wrote the manuscript. All authors read and approved the final manuscript.

## Supplementary Material

Additional file 1 List of the 1,244 specimens analyzed. Click here for file

Additional file 2 **NJ dendrogram of the 1,244 specimens (254 species) analyzed.** Node values = bootstrap test (1,000 pseudo-replicas). Click here for file

Additional file 3 Pairs of species that showed low K2P distance genetic values (<2%). Click here for file

Additional file 4 Nucleotide diagnostic approach (ND) of all pairs of species that showed low K2P distance genetic values (<2%). Click here for file
