# Supplementary material for: Can DNA barcoding accurately discriminate megadiverse Neotropical freshwater fish fauna?
Source: BMC Genet. 2013 Mar 9;14:20. doi: 10.1186/1471-2156-14-20 (PMC3608943; doi:10.1186/1471-2156-14-20)
Supplement: Additional file 1 — List of the 1,244 specimens analyzed. [file 1471-2156-14-20-S1.docx]

**Additional file 1:** List of the 1,244 specimens analyzed.

| **Order** | **Family** | **Species** | **Lat** | **Lon** | **Collection Voucher** | **BOLD ID Number** | **GenBank Accession Number** |
| --- | --- | --- | --- | --- | --- | --- | --- |
| Characiformes | Acestrorhynchidae | *Acestrorhynchus lacustris* | -15,72550011 | -47,94020081 | LBPV-15173 | FUPR023-09 | JN988661 |
| Characiformes | Acestrorhynchidae | *Acestrorhynchus lacustris* | -15,73490047 | -47,93289948 | LBPV-15174 | FUPR024-09 | JN988662 |
| Characiformes | Acestrorhynchidae | *Acestrorhynchus lacustris* | -15,66730022 | -47,95240021 | LBPV-15206 | FUPR027-09 | JN988665 |
| Characiformes | Acestrorhynchidae | *Acestrorhynchus lacustris* | -15,73550034 | -47,91859818 | LBPV-17212 | FUPR025-09 | JN988663 |
| Characiformes | Acestrorhynchidae | *Acestrorhynchus lacustris* | -15,66730022 | -47,95240021 | LBPV-17213 | FUPR026-09 | JN988664 |
| Characiformes | Anostomidae | *Leporellus vittatus* | -23,77029991 | -46,31100082 | LBPV-11254 | FUPR392-09 | GU701641 |
| Characiformes | Anostomidae | *Leporellus vittatus* | -23,77029991 | -46,31100082 | LBPV-11255 | FUPR393-09 | GU701640 |
| Characiformes | Anostomidae | *Leporellus vittatus* | -23,77029991 | -46,31100082 | LBPV-11256 | FUPR394-09 | JN988978 |
| Characiformes | Anostomidae | *Leporellus vittatus* | -23,77029991 | -46,31100082 | LBPV-31749 | FUPR391-09 | GU701943 |
| Characiformes | Anostomidae | *Leporinus amblyrhynchus* | -22,78560066 | -48,48149872 | LBPV-20121 | FUPR028-09 | JN988979 |
| Characiformes | Anostomidae | *Leporinus amblyrhynchus* | -22,78560066 | -48,48149872 | LBPV-21828 | FUPR029-09 | JN988980 |
| Characiformes | Anostomidae | *Leporinus amblyrhynchus* | -22,76339912 | -48,26160049 | LBPV-21829 | FUPR030-09 | JN988981 |
| Characiformes | Anostomidae | *Leporinus amblyrhynchus* | -22,76339912 | -48,26160049 | LBPV-21830 | FUPR031-09 | JN988982 |
| Characiformes | Anostomidae | *Leporinus elongatus* | -22,76339912 | -48,26160049 | LBPV-19849 | FUPR1206-10 | JN988985 |
| Characiformes | Anostomidae | *Leporinus elongatus* | -22,6215992 | -52,76409912 | LBPV-19850 | FUPR1207-10 | JN988984 |
| Characiformes | Anostomidae | *Leporinus elongatus* | -22,6215992 | -52,76409912 | LBPV-19852 | FUPR1209-10 | JN988983 |
| Characiformes | Anostomidae | *Leporinus friderici* | -25,4197998 | -54,53559875 | LBPV-44953 | FUPR1342-10 | JN988988 |
| Characiformes | Anostomidae | *Leporinus friderici* | -25,4197998 | -54,53559875 | LBPV-44954 | FUPR1343-10 | JN988987 |
| Characiformes | Anostomidae | *Leporinus friderici* | -20,78890038 | -48,32970047 | LBPV-44955 | FUPR1344-10 | JN988986 |
| Characiformes | Anostomidae | *Leporinus friderici* | -25,4197998 | -54,53559875 | LBPV-9056 | FUPR034-09 | JN988991 |
| Characiformes | Anostomidae | *Leporinus friderici* | -25,4197998 | -54,53559875 | LBPV-9213 | FUPR032-09 | JN988989 |
| Characiformes | Anostomidae | *Leporinus friderici* | -25,4197998 | -54,53559875 | LBPV-9215 | FUPR033-09 | JN988990 |
| Characiformes | Anostomidae | *Leporinus lacustris* | -20,78890038 | -48,32970047 | LBPV-17346 | FUPR035-09 | JN988992 |
| Characiformes | Anostomidae | *Leporinus lacustris* | -21,87660027 | -47,41180038 | LBPV-17347 | FUPR036-09 | JN988993 |
| Characiformes | Anostomidae | *Leporinus lacustris* | -21,87660027 | -47,41180038 | LBPV-20138 | FUPR037-09 | JN988994 |
| Characiformes | Anostomidae | *Leporinus lacustris* | -21,87660027 | -47,41180038 | LBPV-20139 | FUPR038-09 | JN988995 |
| Characiformes | Anostomidae | *Leporinus lacustris* | -21,87660027 | -47,41180038 | LBPV-20140 | FUPR039-09 | JN988996 |
| Characiformes | Anostomidae | *Leporinus lacustris* | -25,4197998 | -54,53559875 | LBPV-20141 | FUPR040-09 | JN988997 |
| Characiformes | Anostomidae | *Leporinus lacustris* | -25,4197998 | -54,53559875 | LBPV-21857 | FUPR041-09 | JN988998 |
| Characiformes | Anostomidae | *Leporinus macrocephalus* | -25,4197998 | -54,53559875 | LBPV-19469 | FUPR1203-10 | JN988999 |
| Characiformes | Anostomidae | *Leporinus microphthalmus* | -25,4197998 | -54,53559875 | LBPV-35830 | FUPR204-09 | JN989000 |
| Characiformes | Anostomidae | *Leporinus microphthalmus* | -22,6215992 | -52,76409912 | LBPV-35831 | FUPR205-09 | JN989001 |
| Characiformes | Anostomidae | *Leporinus microphthalmus* | -22,6215992 | -52,76409912 | LBPV-35832 | FUPR206-09 | JN989002 |
| Characiformes | Anostomidae | *Leporinus obtusidens* | -23,64170074 | -51,85900116 | LBPV-15587 | FUPR1202-10 | JN989005 |
| Characiformes | Anostomidae | *Leporinus obtusidens* | -23,92469978 | -50,6242981 | LBPV-19851 | FUPR1208-10 | JN989004 |
| Characiformes | Anostomidae | *Leporinus obtusidens* | -23,92469978 | -50,6242981 | LBPV-19854 | FUPR1210-10 | JN989003 |
| Characiformes | Anostomidae | *Leporinus octofasciatus* | -17,09939957 | -48,76150131 | LBPV-9231 | FUPR042-09 | JN989006 |
| Characiformes | Anostomidae | *Leporinus octofasciatus* | -17,09939957 | -48,76150131 | LBPV-9239 | FUPR043-09 | JN989007 |
| Characiformes | Anostomidae | *Leporinus octofasciatus* | -17,09939957 | -48,76150131 | LBPV-9244 | FUPR044-09 | JN989008 |
| Characiformes | Anostomidae | *Leporinus octofasciatus* | -25,31909943 | -52,48870087 | LBPV-22674 | FUPR045-09 | JN989009 |
| Characiformes | Anostomidae | *Leporinus paranensis* | -25,09210014 | -52,49459839 | LBPV-21936 | FUPR046-09 | JN989013 |
| Characiformes | Anostomidae | *Leporinus paranensis* | -23,73150063 | -52,73720169 | LBPV-21937 | FUPR047-09 | JN989014 |
| Characiformes | Anostomidae | *Leporinus paranensis* | -22,79140091 | -53,34939957 | LBPV-21938 | FUPR048-09 | JN989015 |
| Characiformes | Anostomidae | *Leporinus paranensis* | -22,65029907 | -53,09159851 | LBPV-21939 | FUPR049-09 | JN989016 |
| Characiformes | Anostomidae | *Leporinus paranensis* | -22,65029907 | -53,09159851 | LBPV-22443 | FUPR050-09 | JN989017 |
| Characiformes | Anostomidae | *Leporinus striatus* | -23,67469978 | -52,1189003 | LBPV-16871 | FUPR052-09 | JN989019 |
| Characiformes | Anostomidae | *Leporinus striatus* | -23,67469978 | -52,1189003 | LBPV-17483 | FUPR051-09 | JN989018 |
| Characiformes | Anostomidae | *Leporinus striatus* | -22,38439941 | -47,64419937 | LBPV-25910 | FUPR214-09 | JN989020 |
| Characiformes | Anostomidae | *Schizodon intermedius* | -22,61000061 | -45,49200058 | LBPV-17450 | FUPR053-09 | JN989215 |
| Characiformes | Anostomidae | *Schizodon intermedius* | -22,74300003 | -45,73899841 | LBPV-17451 | FUPR054-09 | JN989216 |
| Characiformes | Anostomidae | *Schizodon intermedius* | -22,74300003 | -45,73899841 | LBPV-17452 | FUPR055-09 | JN989217 |
| Characiformes | Anostomidae | *Schizodon intermedius* | -22,74300003 | -45,73899841 | LBPV-17453 | FUPR056-09 | JN989218 |
| Characiformes | Anostomidae | *Schizodon intermedius* | -23,52700043 | -45,76200104 | LBPV-19847 | FUPR057-09 | JN989219 |
| Characiformes | Anostomidae | *Schizodon nasutus* | -23,52700043 | -45,76200104 | LBPV-20160 | FUPR058-09 | JN989220 |
| Characiformes | Anostomidae | *Schizodon nasutus* | -23,52700043 | -45,76200104 | LBPV-20161 | FUPR059-09 | JN989221 |
| Characiformes | Anostomidae | *Schizodon nasutus* | -23,52700043 | -45,76200104 | LBPV-20162 | FUPR060-09 | JN989222 |
| Characiformes | Anostomidae | *Schizodon nasutus* | -23,52700043 | -45,76200104 | LBPV-21861 | FUPR215-09 | JN989223 |
| Characiformes | Bryconidae | *Brycon nattereri* | -23,33329964 | -48,56669998 | LBPV-37542 | FUPR439-09 | GU701451 |
| Characiformes | Bryconidae | *Brycon nattereri* | -23,33329964 | -48,56669998 | LBPV-37543 | FUPR440-09 | GU701946 |
| Characiformes | Bryconidae | *Brycon nattereri* | -23,33329964 | -48,56669998 | LBPV-37544 | FUPR441-09 | GU701947 |
| Characiformes | Bryconidae | *Brycon nattereri* | -23,33329964 | -48,56669998 | LBPV-37545 | FUPR442-09 | GU701450 |
| Characiformes | Bryconidae | *Brycon orbignyanus* | -21,0128994 | -49,69029999 | LBPV-10480 | FUPR240-09 | JN988763 |
| Characiformes | Bryconidae | *Brycon orbignyanus* | -22,71759987 | -53,29100037 | LBPV-18005 | FUPR443-09 | JN988764 |
| Characiformes | Bryconidae | *Brycon orbignyanus* | -23,52499962 | -45,7879982 | LBPV-18006 | FUPR248-09 | JN988765 |
| Characiformes | Bryconidae | *Brycon orbignyanus* | -21,0128994 | -49,69029999 | LBPV-18007 | FUPR241-09 | GU701950 |
| Characiformes | Bryconidae | *Salminus brasiliensis* | -22,19400024 | -46,37900162 | LBPV-9026 | FUPR325-09 | GU701884 |
| Characiformes | Bryconidae | *Salminus brasiliensis* | -22,19400024 | -46,37900162 | LBPV-9028 | FUPR326-09 | GU701885 |
| Characiformes | Bryconidae | *Salminus brasiliensis* | -22,19400024 | -46,37900162 | LBPV-9029 | FUPR327-09 | GU701514 |
| Characiformes | Bryconidae | *Salminus brasiliensis* | - | - | LBPV-9030 | FUPR329-09 | GU701886 |
| Characiformes | Bryconidae | *Salminus brasiliensis* | - | - | LBPV-9031 | FUPR330-09 | GU701513 |
| Characiformes | Bryconidae | *Salminus brasiliensis* | -22,70700073 | -45,7159996 | LBPV-9032 | FUPR331-09 | GU701517 |
| Characiformes | Bryconidae | *Salminus brasiliensis* | -22,70700073 | -45,7159996 | LBPV-9033 | FUPR332-09 | GU701516 |
| Characiformes | Bryconidae | *Salminus hilarii* | -22,70700073 | -45,7159996 | LBPV-29454 | FUPR133-09 | JN989209 |
| Characiformes | Bryconidae | *Salminus hilarii* | -22,6760006 | -45,55899811 | LBPV-29456 | FUPR134-09 | JN989210 |
| Characiformes | Bryconidae | *Salminus hilarii* | -22,6760006 | -45,55899811 | LBPV-29457 | FUPR135-09 | JN989211 |
| Characiformes | Bryconidae | *Salminus hilarii* | -22,6760006 | -45,55899811 | LBPV-29458 | FUPR136-09 | JN989212 |
| Characiformes | Characidae | *Aphyocharax anisitsi* | -23,65579987 | -46,83110046 | LBPV-19747 | FUPR061-09 | JN988677 |
| Characiformes | Characidae | *Aphyocharax anisitsi* | -23,65579987 | -46,83110046 | LBPV-32159 | FUPR207-09 | JN988678 |
| Characiformes | Characidae | *Aphyocharax anisitsi* | -23,65579987 | -46,83110046 | LBPV-30160 | FUPR239-09 | JN988680 |
| Characiformes | Characidae | *Aphyocharax anisitsi* | -23,65579987 | -46,83110046 | LBPV-32160 | FUPR415-09 | JN988679 |
| Characiformes | Characidae | *Aphyocharax dentatus* | -20,94709969 | -48,14780045 | LBPV-45650 | FUPR1357-10 | JN988683 |
| Characiformes | Characidae | *Aphyocharax dentatus* | -20,94709969 | -48,14780045 | LBPV-45711 | FUPR1359-10 | JN988682 |
| Characiformes | Characidae | *Aphyocharax dentatus* | -20,94709969 | -48,14780045 | LBPV-45712 | FUPR1360-10 | JN988681 |
| Characiformes | Characidae | *Aphyocheirodon hemigrammus* | -20,4932003 | -48,63570023 | LBPV-40025 | FUPR1266-10 | JN988688 |
| Characiformes | Characidae | *Aphyocheirodon hemigrammus* | -22,71759987 | -53,29100037 | LBPV-40026 | FUPR1267-10 | JN988687 |
| Characiformes | Characidae | *Aphyocheirodon hemigrammus* | -23,64159966 | -51,85929871 | LBPV-40027 | FUPR1268-10 | JN988686 |
| Characiformes | Characidae | *Aphyocheirodon hemigrammus* | -22,63260078 | -53,05260086 | LBPV-40028 | FUPR1269-10 | JN988685 |
| Characiformes | Characidae | *Aphyocheirodon hemigrammus* | -22,63260078 | -53,05260086 | LBPV-40029 | FUPR1270-10 | JN988684 |
| Characiformes | Characidae | *Astyanax altiparanae* | -23,33329964 | -48,56669998 | LBPV-17317 | FUPR1037-10 | JN988696 |
| Characiformes | Characidae | *Astyanax altiparanae* | -21,92709923 | -47,36790085 | LBPV-17318 | FUPR1038-10 | JN988695 |
| Characiformes | Characidae | *Astyanax altiparanae* | -22,71759987 | -53,29100037 | LBPV-19523 | FUPR1039-10 | JN988694 |
| Characiformes | Characidae | *Astyanax altiparanae* | -22,71759987 | -53,29100037 | LBPV-19524 | FUPR1040-10 | JN988693 |
| Characiformes | Characidae | *Astyanax altiparanae* | -22,65029907 | -53,09159851 | LBPV-19553 | FUPR1041-10 | JN988715 |
| Characiformes | Characidae | *Astyanax altiparanae* | -22,79140091 | -53,34939957 | LBPV-19554 | FUPR1042-10 | JN988714 |
| Characiformes | Characidae | *Astyanax altiparanae* | -22,82769966 | -48,37070084 | LBPV-20174 | FUPR1129-10 | JN988704 |
| Characiformes | Characidae | *Astyanax altiparanae* | -21,0128994 | -49,69029999 | LBPV-20175 | FUPR1130-10 | JN988703 |
| Characiformes | Characidae | *Astyanax altiparanae* | -22,64710045 | -53,07690048 | LBPV-21444 | FUPR1044-10 | JN988712 |
| Characiformes | Characidae | *Astyanax altiparanae* | -22,64710045 | -53,07690048 | LBPV-21445 | FUPR1045-10 | JN988711 |
| Characiformes | Characidae | *Astyanax altiparanae* | -21,37750053 | -47,65919876 | LBPV-21446 | FUPR1046-10 | JN988710 |
| Characiformes | Characidae | *Astyanax altiparanae* | -21,0128994 | -49,69029999 | LBPV-24768 | FUPR1131-10 | JN988702 |
| Characiformes | Characidae | *Astyanax altiparanae* | -23,09630013 | -48,25139999 | LBPV-24769 | FUPR1132-10 | JN988701 |
| Characiformes | Characidae | *Astyanax altiparanae* | -22,79140091 | -53,34939957 | LBPV-26384 | FUPR1043-10 | JN988713 |
| Characiformes | Characidae | *Astyanax altiparanae* | -23,41250038 | -49,57089996 | LBPV-29960 | FUPR1133-10 | JN988700 |
| Characiformes | Characidae | *Astyanax altiparanae* | -23,13360023 | -49,67620087 | LBPV-29961 | FUPR1134-10 | JN988699 |
| Characiformes | Characidae | *Astyanax altiparanae* | -23,13360023 | -49,67620087 | LBPV-34717 | FUPR1135-10 | JN988698 |
| Characiformes | Characidae | *Astyanax altiparanae* | -21,37750053 | -47,65919876 | LBPV-35319 | FUPR1047-10 | JN988709 |
| Characiformes | Characidae | *Astyanax altiparanae* | -22,67340088 | -53,08580017 | LBPV-35320 | FUPR1048-10 | JN988708 |
| Characiformes | Characidae | *Astyanax altiparanae* | -22,67340088 | -53,08580017 | LBPV-35321 | FUPR1049-10 | JN988707 |
| Characiformes | Characidae | *Astyanax altiparanae* | -22,67340088 | -53,08580017 | LBPV-35322 | FUPR1050-10 | JN988706 |
| Characiformes | Characidae | *Astyanax altiparanae* | -22,82769966 | -48,37070084 | LBPV-35323 | FUPR1051-10 | JN988705 |
| Characiformes | Characidae | *Astyanax altiparanae* | -23,13360023 | -49,67620087 | LBPV-36236 | FUPR1136-10 | JN988697 |
| Characiformes | Characidae | *Astyanax altiparanae* | -23,33329964 | -48,56669998 | LBPV-8910 | FUPR1036-10 | JN988749 |
| Characiformes | Characidae | *Astyanax biotae* | -20,43350029 | -51,26139832 | LBPV-40264 | FUPR999-09 | JN988718 |
| Characiformes | Characidae | *Astyanax biotae* | -21,10700035 | -50,26459885 | LBPV-40265 | FUPR1000-09 | JN988719 |
| Characiformes | Characidae | *Astyanax biotae* | -21,10700035 | -50,26459885 | LBPV-40266 | FUPR1001-09 | JN988720 |
| Characiformes | Characidae | *Astyanax biotae* | -21,10700035 | -50,26459885 | LBPV-40267 | FUPR1002-09 | JN988721 |
| Characiformes | Characidae | *Astyanax biotae* | -21,10700035 | -50,26459885 | LBPV-40268 | FUPR1003-09 | JN988722 |
| Characiformes | Characidae | *Astyanax biotae* | -23,33329964 | -48,56669998 | LIRP-4276a | FUPR1061-10 | JN988717 |
| Characiformes | Characidae | *Astyanax biotae* | -23,33329964 | -48,56669998 | LIRP-4276b | FUPR1062-10 | JN988716 |
| Characiformes | Characidae | *Astyanax bockmanni* | -23,33329964 | -48,56669998 | LBPV-25951 | FUPR1110-10 | JN988728 |
| Characiformes | Characidae | *Astyanax bockmanni* | -20,39959908 | -50,32030106 | LBPV-25952 | FUPR1111-10 | JN988727 |
| Characiformes | Characidae | *Astyanax bockmanni* | -20,08539963 | -50,98310089 | LBPV-25974 | FUPR1112-10 | JN988726 |
| Characiformes | Characidae | *Astyanax bockmanni* | -23,02389908 | -48,82569885 | LBPV-25975 | FUPR1113-10 | JN988725 |
| Characiformes | Characidae | *Astyanax bockmanni* | -23,02389908 | -48,82569885 | LBPV-26330 | FUPR1114-10 | JN988724 |
| Characiformes | Characidae | *Astyanax bockmanni* | -23,02389908 | -48,82569885 | LBPV-33575 | FUPR1187-10 | JN988723 |
| Characiformes | Characidae | *Astyanax fasciatus* | -23,5238 | -45,8896 | LBPV-22587 | FUPR-1070-10 | JQ353530 |
| Characiformes | Characidae | *Astyanax fasciatus* | -23.5819 | -45,9669 | LBPV-32215 | FUPR-1075-10 | JQ353582 |
| Characiformes | Characidae | *Astyanax fasciatus* | -23.5819 | -45,9669 | LBPV-32216 | FUPR-1076-10 | JQ353581 |
| Characiformes | Characidae | *Astyanax fasciatus* | -23.5819 | -45,9669 | LBPV-32217 | FUPR-1077-10 | JQ353580 |
| Characiformes | Characidae | *Astyanax fasciatus* | -23.5819 | -45,9669 | LBPV-32218 | FUPR-1078-10 | JQ353579 |
| Characiformes | Characidae | *Astyanax paranae* | -22,82040024 | -48,10409927 | LBPV-29735 | FUPR1087-10 | JN988740 |
| Characiformes | Characidae | *Astyanax paranae* | -22,82040024 | -48,10409927 | LBPV-29736 | FUPR1088-10 | JN988739 |
| Characiformes | Characidae | *Astyanax paranae* | -22,63209915 | -48,17509842 | LBPV-29737 | FUPR1089-10 | JN988738 |
| Characiformes | Characidae | *Astyanax paranae* | -22,63209915 | -48,17509842 | LBPV-29738 | FUPR1090-10 | [JQ353617](http://www.ncbi.nlm.nih.gov/nuccore/JQ353617) |
| Characiformes | Characidae | *Astyanax paranae* | -22,63209915 | -48,17509842 | LBPV-29739 | FUPR1091-10 | JQ353616 |
| Characiformes | Characidae | *Astyanax paranae* | -22,63209915 | -48,17509842 | LBPV-20740 | FUPR1092-10 | JQ353615 |
| Characiformes | Characidae | *Astyanax paranae* | -22,405 | -47,812 | LBPV-5978 | BAST-525-12 | - |
| Characiformes | Characidae | *Astyanax schubarti* | -22,91670036 | -48,5 | LBPV-31723 | FUPR1124-10 | JN988745 |
| Characiformes | Characidae | *Astyanax schubarti* | -22,91670036 | -48,5 | LBPV-31724 | FUPR1125-10 | JN988744 |
| Characiformes | Characidae | *Astyanax schubarti* | -23,14999962 | -48,26670074 | LBPV-31725 | FUPR1126-10 | JN988743 |
| Characiformes | Characidae | *Astyanax schubarti* | -23,14999962 | -48,26670074 | LBPV-31726 | FUPR1127-10 | JN988742 |
| Characiformes | Characidae | *Astyanax schubarti* | -23,33329964 | -48,56669998 | LBPV-31728 | FUPR1128-10 | JN988741 |
| Characiformes | Characidae | *Astyanax trierythropterus* | -23,77029991 | -46,31100082 | LBPV-26027 | FUPR1179-10 | JN988747 |
| Characiformes | Characidae | *Astyanax trierythropterus* | -23,77029991 | -46,31100082 | LBPV-26028 | FUPR1180-10 | JN988746 |
| Characiformes | Characidae | *Astyanax trierythropterus* | -21,10700035 | -50,26459885 | LBPV-26029 | FUPR1106-10 | JN988748 |
| Characiformes | Characidae | *Bryconamericus iheringi* | -22,71759987 | -53,29100037 | LBPV-18706 | FUPR065-09 | JN988755 |
| Characiformes | Characidae | *Bryconamericus iheringi* | -21,10700035 | -50,26459885 | LBPV-18707 | FUPR066-09 | JN988756 |
| Characiformes | Characidae | *Bryconamericus iheringi* | -20,40320015 | -48,63570023 | LBPV-29777 | FUPR1232-10 | JN988754 |
| Characiformes | Characidae | *Bryconamericus iheringi* | -20,40320015 | -48,63570023 | LBPV-29778 | FUPR1233-10 | JN988753 |
| Characiformes | Characidae | *Bryconamericus iheringi* | -20,40320015 | -48,63570023 | LBPV-29924 | FUPR1237-10 | JN988752 |
| Characiformes | Characidae | *Bryconamericus iheringi* | -20,74550056 | -49,77930069 | LBPV-29925 | FUPR1238-10 | JN988751 |
| Characiformes | Characidae | *Bryconamericus iheringi* | -21,10700035 | -50,26459885 | LBPV-34658 | FUPR180-09 | JN988757 |
| Characiformes | Characidae | *Bryconamericus iheringi* | -21,10700035 | -50,26459885 | LBPV-34762 | FUPR182-09 | JN988758 |
| Characiformes | Characidae | *Bryconamericus turiuba* | -20,74550056 | -49,77930069 | LBPV-31886 | FUPR081-09 | JN988759 |
| Characiformes | Characidae | *Bryconamericus stramineus* | -22,99040031 | -48,42720032 | LBPV-14155 | FUPR063-09 | JN988761 |
| Characiformes | Characidae | *Bryconamericus stramineus* | -22,99040031 | -48,42720032 | LBPV-14156 | FUPR064-09 | JN988762 |
| Characiformes | Characidae | *Bryconamericus stramineus* | -22,36860085 | -47,47719955 | LBPV-44828 | FUPR1333-10 | JN988760 |
| Characiformes | Characidae | *Cheirodon stenodon* | -25,4197998 | -54,53559875 | LBPV-26118 | FUPR1218-10 | JN988795 |
| Characiformes | Characidae | *Cheirodon stenodon* | -25,4197998 | -54,53559875 | LBPV-26119 | FUPR1219-10 | JN988794 |
| Characiformes | Characidae | *Cheirodon stenodon* | -25,4197998 | -54,53559875 | LBPV-26120 | FUPR1220-10 | JN988793 |
| Characiformes | Characidae | *Cheirodon stenodon* | -25,4197998 | -54,53559875 | LBPV-26121 | FUPR1221-10 | JN988792 |
| Characiformes | Characidae | *Galeocharax knerii* | -20,34230042 | -46,7867012 | LBPV-3923 | FUPR067-09 | JN988861 |
| Characiformes | Characidae | *Galeocharax knerii* | -23,93829918 | -50,72900009 | LBPV-20164 | FUPR068-09 | JN988862 |
| Characiformes | Characidae | *Glandulocauda melanogenys* | -21,24559975 | -48,29729843 | LBPV-24539 | FUPR069-09 | JN988870 |
| Characiformes | Characidae | *Glandulocauda melanogenys* | -21,24559975 | -48,29729843 | LBPV-24540 | FUPR070-09 | JN988871 |
| Characiformes | Characidae | *Glandulocauda melanogenys* | -21,24559975 | -48,29729843 | LBPV-24541 | FUPR071-09 | JN988872 |
| Characiformes | Characidae | *Gymnocorymbus ternetzi* | -21,24559975 | -48,29729843 | LBPV-26087 | FUPR072-09 | JN988873 |
| Characiformes | Characidae | *Gymnocorymbus ternetzi* | -21,24559975 | -48,29729843 | LBPV-26088 | FUPR073-09 | JN988874 |
| Characiformes | Characidae | *Gymnocorymbus ternetzi* | -17,69510078 | -40,76979828 | LBPV-26309 | FUPR194-09 | JN988876 |
| Characiformes | Characidae | *Gymnocorymbus ternetzi* | -17,69510078 | -40,76979828 | LBPV-26310 | FUPR074-09 | JN988875 |
| Characiformes | Characidae | *Gymnocorymbus ternetzi* | -17,69510078 | -40,76979828 | LBPV-26311 | FUPR195-09 | JN988877 |
| Characiformes | Characidae | *Hasemania hanseni* | -24,20059967 | -48,41839981 | LBPV-44575 | FUPR1306-10 | JN988889 |
| Characiformes | Characidae | *Hasemania hanseni* | -23,5237999 | -45,88959885 | LBPV-44576 | FUPR1307-10 | JN988888 |
| Characiformes | Characidae | *Hasemania hanseni* | -22,79140091 | -53,34939957 | LBPV-44659 | FUPR1314-10 | JN988887 |
| Characiformes | Characidae | *Hasemania hanseni* | -17,09939957 | -48,76150131 | LBPV-44728 | FUPR1319-10 | JN988886 |
| Characiformes | Characidae | *Hasemania hanseni* | -17,09939957 | -48,76150131 | LBPV-44746 | FUPR1322-10 | JN988885 |
| Characiformes | Characidae | *Hasemania hanseni* | -21,19309998 | -49,12279892 | LBPV-44801 | FUPR1332-10 | JN988884 |
| Characiformes | Characidae | *Hemigrammus marginatus* | -21,19309998 | -49,12279892 | LBPV-17392 | FUPR424-09 | GU701410 |
| Characiformes | Characidae | *Hemigrammus marginatus* | -22,64789963 | -53,07860184 | LBPV-25868 | FUPR176-09 | JN988890 |
| Characiformes | Characidae | *Hemigrammus marginatus* | -22,64789963 | -53,07860184 | LBPV-25869 | FUPR177-09 | JN988891 |
| Characiformes | Characidae | *Hemigrammus marginatus* | -22,64789963 | -53,07860184 | LBPV-29416 | FUPR196-09 | JN988892 |
| Characiformes | Characidae | *Hemigrammus marginatus* | -25,42000008 | -54,5359993 | LBPV-29417 | FUPR197-09 | JN988893 |
| Characiformes | Characidae | *Hemigrammus marginatus* | -21,19309998 | -49,12279892 | LBPV-29418 | FUPR198-09 | JN988894 |
| Characiformes | Characidae | *Hollandichthys multifasciatus* | -22,79140091 | -53,34939957 | LBPV-24548 | FUPR075-09 | JN988898 |
| Characiformes | Characidae | *Hollandichthys multifasciatus* | -22,79140091 | -53,34939957 | LBPV-24549 | FUPR076-09 | JN988899 |
| Characiformes | Characidae | *Hollandichthys multifasciatus* | -22,7859993 | -45,6882019 | LBPV-24550 | FUPR416-09 | GU701423 |
| Characiformes | Characidae | *Hollandichthys multifasciatus* | -22,7859993 | -45,6882019 | LBPV-24551 | FUPR417-09 | GU701411 |
| Characiformes | Characidae | *Hollandichthys multifasciatus* | -22,7859993 | -45,6882019 | LBPV-24552 | FUPR418-09 | GU701419 |
| Characiformes | Characidae | *Hyphessobrycon balbus* | -22,71759987 | -53,29100037 | LBPV-44723 | FUPR1317-10 | JN988918 |
| Characiformes | Characidae | *Hyphessobrycon balbus* | -22,71759987 | -53,29100037 | LBPV-44724 | FUPR1318-10 | JN988917 |
| Characiformes | Characidae | *Hyphessobrycon balbus* | -22,71759987 | -53,29100037 | LBPV-44771 | FUPR1326-10 | JN988916 |
| Characiformes | Characidae | *Hyphessobrycon balbus* | -22,79140091 | -53,34939957 | LBPV-44779 | FUPR1330-10 | JN988915 |
| Characiformes | Characidae | *Hyphessobrycon balbus* | -17,09939957 | -48,76150131 | LBPV-44790 | FUPR1331-10 | JN988914 |
| Characiformes | Characidae | *Hyphessobrycon bifasciatus* | -22,63209915 | -48,17509842 | LBPV-22646 | FUPR420-09 | JN988920 |
| Characiformes | Characidae | *Hyphessobrycon bifasciatus* | -22,71759987 | -53,29100037 | LBPV-22648 | FUPR422-09 | JN988919 |
| Characiformes | Characidae | *Hyphessobrycon bifasciatus* | -23,33329964 | -48,56669998 | LBPV-25895 | FUPR419-09 | HM422469 |
| Characiformes | Characidae | *Hyphessobrycon eques* | -22,67639923 | -45,55939865 | LBPV-17327 | FUPR178-09 | JN988925 |
| Characiformes | Characidae | *Hyphessobrycon eques* | -22,64710045 | -53,07690048 | LBPV-19557 | FUPR179-09 | JN988924 |
| Characiformes | Characidae | *Hyphessobrycon eques* | -22,64710045 | -53,07690048 | LBPV-29401 | FUPR211-09 | JN988923 |
| Characiformes | Characidae | *Hyphessobrycon eques* | -22,64710045 | -53,07690048 | LBPV-29402 | FUPR212-09 | JN988922 |
| Characiformes | Characidae | *Hyphessobrycon eques* | -22,64710045 | -53,07690048 | LBPV-29403 | FUPR213-09 | JN988921 |
| Characiformes | Characidae | *Hyphessobrycon eques* | -22,64710045 | -53,07690048 | LBPV-8220 | FUPR423-09 | GU701409 |
| Characiformes | Characidae | *Hyphessobrycon flammeus* | -21,19309998 | -49,12279892 | LBPV-36303 | FUPR1027-09 | GU701421 |
| Characiformes | Characidae | *Hyphessobrycon flammeus* | -24,07349968 | -52,29109955 | LBPV-36304 | FUPR375-09 | GU701882 |
| Characiformes | Characidae | *Hyphessobrycon flammeus* | -24,07349968 | -52,29109955 | LBPV-36305 | FUPR376-09 | GU701883 |
| Characiformes | Characidae | *Hyphessobrycon flammeus* | -25,09210014 | -52,49459839 | LBPV-36306 | FUPR377-09 | GU701879 |
| Characiformes | Characidae | *Hyphessobrycon flammeus* | -25,09210014 | -52,49459839 | LBPV-36307 | FUPR378-09 | GU701880 |
| Characiformes | Characidae | *Hyphessobrycon flammeus* | -22,78560066 | -48,48149872 | LBPV-40460 | FUPR984-09 | GU701915 |
| Characiformes | Characidae | *Hyphessobrycon flammeus* | -21,19309998 | -49,12279892 | LBPV-40461 | FUPR985-09 | GU701916 |
| Characiformes | Characidae | *Hyphessobrycon flammeus* | -21,19309998 | -49,12279892 | LBPV-40462 | FUPR986-09 | GU701912 |
| Characiformes | Characidae | *Hyphessobrycon flammeus* | -21,19309998 | -49,12279892 | LBPV-40463 | FUPR987-09 | GU701913 |
| Characiformes | Characidae | *Hyphessobrycon flammeus* | -21,19309998 | -49,12279892 | LBPV-40464 | FUPR988-09 | GU701909 |
| Characiformes | Characidae | *Hyphessobrycon sp1* | -17,40239906 | -51,27159882 | LBPV-34942 | FUPR408-09 | GU701734 |
| Characiformes | Characidae | *Hyphessobrycon sp1* | -17,40239906 | -51,27159882 | LBPV-34943 | FUPR409-09 | GU701738 |
| Characiformes | Characidae | *Hyphessobrycon sp1* | -17,40239906 | -51,27159882 | LBPV-34944 | FUPR410-09 | GU701737 |
| Characiformes | Characidae | *Hyphessobrycon sp1* | -22,65029907 | -53,09159851 | LBPV-34945 | FUPR411-09 | GU701740 |
| Characiformes | Characidae | *Hyphessobrycon sp1* | -21,92709923 | -47,36790085 | LBPV-34946 | FUPR412-09 | GU701739 |
| Characiformes | Characidae | *Hyphessobrycon sp1* | -24,07349968 | -52,29109955 | LBPV-34947 | FUPR413-09 | GU701741 |
| Characiformes | Characidae | *Hyphessobrycon sp2* | -17,40239906 | -51,27159882 | LBPV-22647 | FUPR421-09 | HM422470 |
| Characiformes | Characidae | *Knodus moenkhausii* | -21,19309998 | -49,12279892 | LBPV-31934 | FUPR001-09 | JN988963 |
| Characiformes | Characidae | *Knodus moenkhausii* | -21,19309998 | -49,12279892 | LBPV-31935 | FUPR002-09 | JN988964 |
| Characiformes | Characidae | *Knodus moenkhausii* | -20,01029968 | -43,96900177 | LBPV-31936 | FUPR003-09 | JN988965 |
| Characiformes | Characidae | *Knodus moenkhausii* | -20,09659958 | -44,04249954 | LBPV-31962 | FUPR004-09 | JN988966 |
| Characiformes | Characidae | *Knodus moenkhausii* | -20,09659958 | -44,04249954 | LBPV-35950 | FUPR244-09 | JN988967 |
| Characiformes | Characidae | *Knodus moenkhausii* | -21,32699966 | -47,23880005 | LBPV-35951 | FUPR245-09 | JN988968 |
| Characiformes | Characidae | *Knodus moenkhausii* | -22,89929962 | -48,38650131 | LBPV-44501 | FUPR1293-10 | JN988962 |
| Characiformes | Characidae | *Knodus moenkhausii* | -22,89929962 | -48,38650131 | LBPV-44506 | FUPR1294-10 | JN988961 |
| Characiformes | Characidae | *Knodus moenkhausii* | -22,89929962 | -48,38650131 | LBPV-44735 | FUPR1320-10 | JN988960 |
| Characiformes | Characidae | *Knodus moenkhausii* | -23,77129936 | -46,76570129 | LBPV-44740 | FUPR1321-10 | JN988959 |
| Characiformes | Characidae | *Kolpotocheirodon theloura* | -22,67589951 | -45,68289948 | LBPV-25982 | FUPR445-09 | HM376391 |
| Characiformes | Characidae | *Kolpotocheirodon theloura* | -22,67589951 | -45,68289948 | LBPV-44750 | FUPR1323-10 | JN988969 |
| Characiformes | Characidae | *Lophiobrycon weitzmani* | -22,76339912 | -48,26160049 | LBPV-38090 | FUPR395-09 | GU701436 |
| Characiformes | Characidae | *Lophiobrycon weitzmani* | -23,52669907 | -45,81980133 | LBPV-38091 | FUPR396-09 | GU701433 |
| Characiformes | Characidae | *Lophiobrycon weitzmani* | -23,52669907 | -45,81980133 | LBPV-38093 | FUPR397-09 | GU701435 |
| Characiformes | Characidae | *Mimagoniates microlepis* | - | - | LBPV-40276 | FUPR1016-09 | GU701928 |
| Characiformes | Characidae | *Mimagoniates microlepis* | - | - | LBPV-40277 | FUPR1017-09 | GU701929 |
| Characiformes | Characidae | *Mimagoniates microlepis* | - | - | LBPV-40278 | FUPR1018-09 | GU701926 |
| Characiformes | Characidae | *Mimagoniates microlepis* | - | - | LBPV-40279 | FUPR1019-09 | GU701927 |
| Characiformes | Characidae | *Mimagoniates microlepis* | - | - | LBPV-40280 | FUPR1020-09 | JN989038 |
| Characiformes | Characidae | *Moenkhausia forestii* | - | - | LBPV-17341 | FUPR427-09 | GU701432 |
| Characiformes | Characidae | *Moenkhausia forestii* | - | - | LBPV-17342 | FUPR428-09 | GU701434 |
| Characiformes | Characidae | *Moenkhausia forestii* | - | - | LBPV-24751 | FUPR230-09 | JN989043 |
| Characiformes | Characidae | *Moenkhausia forestii* | - | - | LBPV-24752 | FUPR231-09 | JN989042 |
| Characiformes | Characidae | *Moenkhausia forestii* | - | - | LBPV-24753 | FUPR232-09 | JN989041 |
| Characiformes | Characidae | *Moenkhausia forestii* | - | - | LBPV-24754 | FUPR233-09 | JN989040 |
| Characiformes | Characidae | *Moenkhausia forestii* | - | - | LBPV-24755 | FUPR234-09 | JN989039 |
| Characiformes | Characidae | *Moenkhausia intermedia* | - | - | LBPV-20179 | FUPR426-09 | GU701944 |
| Characiformes | Characidae | *Moenkhausia intermedia* | - | - | LBPV-20180 | FUPR242-09 | JN989045 |
| Characiformes | Characidae | *Moenkhausia intermedia* | - | - | LBPV-20181 | FUPR243-09 | JN989044 |
| Characiformes | Characidae | *Moenkhausia sanctaefilomenae* | - | - | LBPV-22521 | FUPR429-09 | GU701430 |
| Characiformes | Characidae | *Moenkhausia sanctaefilomenae* | - | - | LBPV-22522 | FUPR430-09 | GU701431 |
| Characiformes | Characidae | *Odontostilbe microcephala* | -22,79140091 | -53,34939957 | LBPV-26117 | FUPR1217-10 | JN989110 |
| Characiformes | Characidae | *Oligosarcus paranensis* | -22,79140091 | -53,34939957 | LBPV-18701 | FUPR082-09 | JN989111 |
| Characiformes | Characidae | *Oligosarcus paranensis* | -22,79140091 | -53,34939957 | LBPV-18702 | FUPR083-09 | JN989112 |
| Characiformes | Characidae | *Oligosarcus paranensis* | -22,6692009 | -53,09289932 | LBPV-18703 | FUPR084-09 | JN989113 |
| Characiformes | Characidae | *Oligosarcus paranensis* | -23,9545002 | -51,11410141 | LBPV-18786 | FUPR085-09 | JN989114 |
| Characiformes | Characidae | *Oligosarcus paranensis* | -24,07349968 | -52,29109955 | LBPV-22481 | FUPR086-09 | JN989115 |
| Characiformes | Characidae | *Oligosarcus paranensis* | -24,07349968 | -52,29109955 | LBPV-34773 | FUPR319-09 | GU701611 |
| Characiformes | Characidae | *Oligosarcus paranensis* | -24,07349968 | -52,29109955 | LBPV-34774 | FUPR320-09 | GU701610 |
| Characiformes | Characidae | *Oligosarcus paranensis* | -22,67589951 | -45,68289948 | LBPV-34775 | FUPR321-09 | GU701609 |
| Characiformes | Characidae | *Oligosarcus pintoi* | -22,63260078 | -53,05260086 | LBPV-24852 | FUPR322-09 | GU701613 |
| Characiformes | Characidae | *Oligosarcus pintoi* | -22,63260078 | -53,05260086 | LBPV-24853 | FUPR323-09 | GU701612 |
| Characiformes | Characidae | *Piabina anhembi* | -23,72800064 | -46,81399918 | LBPV-20821 | FUPR125-09 | HM144049 |
| Characiformes | Characidae | *Piabina anhembi* | -23,72800064 | -46,81399918 | LBPV-23230 | FUPR123-09 | HM144051 |
| Characiformes | Characidae | *Piabina anhembi* | -22,37800026 | -47,42699814 | LBPV-23360 | FUPR235-09 | HM144048 |
| Characiformes | Characidae | *Piabina anhembi* | -23,72800064 | -46,81399918 | LBPV-23372 | FUPR124-09 | HM144050 |
| Characiformes | Characidae | *Piabina anhembi* | -22,37800026 | -47,42699814 | LBPV-23373 | FUPR236-09 | HM144047 |
| Characiformes | Characidae | *Piabina argentea* | -23,53000069 | -45,79800034 | LBPV-14154 | FUPR062-09 | HM144107 |
| Characiformes | Characidae | *Piabina argentea* | -23,53000069 | -45,79800034 | LBPV-17227 | FUPR087-09 | HM144106 |
| Characiformes | Characidae | *Piabina argentea* | -23,53000069 | -45,79800034 | LBPV-17228 | FUPR088-09 | HM144105 |
| Characiformes | Characidae | *Piabina argentea* | -23,53000069 | -45,79800034 | LBPV-17229 | FUPR089-09 | HM144104 |
| Characiformes | Characidae | *Piabina argentea* | -23,53000069 | -45,79800034 | LBPV-20764 | FUPR090-09 | HM144103 |
| Characiformes | Characidae | *Piabina argentea* | -19,38500023 | -43,65919876 | LBPV-21305 | FUPR091-09 | HM144102 |
| Characiformes | Characidae | *Piabina argentea* | -23,66699982 | -46,18299866 | LBPV-22884 | FUPR189-09 | HM144063 |
| Characiformes | Characidae | *Piabina argentea* | -23,64159966 | -51,85919952 | LBPV-23220 | FUPR109-09 | HM144084 |
| Characiformes | Characidae | *Piabina argentea* | -23,72800064 | -46,81399918 | LBPV-23222 | FUPR237-09 | HM144053 |
| Characiformes | Characidae | *Piabina argentea* | -19,38500023 | -43,65919876 | LBPV-23459 | FUPR092-09 | HM144101 |
| Characiformes | Characidae | *Piabina argentea* | -19,38500023 | -43,65919876 | LBPV-25030 | FUPR093-09 | HM144100 |
| Characiformes | Characidae | *Piabina argentea* | -19,38500023 | -43,65919876 | LBPV-25031 | FUPR094-09 | HM144099 |
| Characiformes | Characidae | *Piabina argentea* | -17,80139923 | -48,37200165 | LBPV-25033 | FUPR095-09 | HM144098 |
| Characiformes | Characidae | *Piabina argentea* | -25,09210014 | -52,49459839 | LBPV-25049 | FUPR100-09 | HM144093 |
| Characiformes | Characidae | *Piabina argentea* | -25,09210014 | -52,49459839 | LBPV-25050 | FUPR101-09 | HM144092 |
| Characiformes | Characidae | *Piabina argentea* | -25,09210014 | -52,49459839 | LBPV-25091 | FUPR102-09 | HM144091 |
| Characiformes | Characidae | *Piabina argentea* | -17,80139923 | -48,37200165 | LBPV-25155 | FUPR096-09 | HM144097 |
| Characiformes | Characidae | *Piabina argentea* | -17,80139923 | -48,37200165 | LBPV-25156 | FUPR097-09 | HM144096 |
| Characiformes | Characidae | *Piabina argentea* | -18,11009979 | -48,50419998 | LBPV-25157 | FUPR098-09 | HM144095 |
| Characiformes | Characidae | *Piabina argentea* | -18,11009979 | -48,50419998 | LBPV-25161 | FUPR099-09 | HM144094 |
| Characiformes | Characidae | *Piabina argentea* | -21,73209953 | -46,42290115 | LBPV-25162 | FUPR103-09 | HM144090 |
| Characiformes | Characidae | *Piabina argentea* | -21,73209953 | -46,42290115 | LBPV-25165 | FUPR104-09 | HM144089 |
| Characiformes | Characidae | *Piabina argentea* | -21,73209953 | -46,42290115 | LBPV-25166 | FUPR105-09 | HM144088 |
| Characiformes | Characidae | *Piabina argentea* | -21,73209953 | -46,42290115 | LBPV-25167 | FUPR106-09 | HM144087 |
| Characiformes | Characidae | *Piabina argentea* | -23,72800064 | -46,81399918 | LBPV-25214 | FUPR238-09 | HM144052 |
| Characiformes | Characidae | *Piabina argentea* | -21,73209953 | -46,42290115 | LBPV-25219 | FUPR107-09 | HM144086 |
| Characiformes | Characidae | *Piabina argentea* | -23,93829918 | -50,72900009 | LBPV-25410 | FUPR108-09 | HM144085 |
| Characiformes | Characidae | *Piabina argentea* | -23,5237999 | -45,88959885 | LBPV-28393 | FUPR126-09 | HM144070 |
| Characiformes | Characidae | *Piabina argentea* | -23,5237999 | -45,88959885 | LBPV-28394 | FUPR127-09 | HM144069 |
| Characiformes | Characidae | *Piabina argentea* | -23,5237999 | -45,88959885 | LBPV-28395 | FUPR128-09 | HM144068 |
| Characiformes | Characidae | *Piabina argentea* | -23,5237999 | -45,88959885 | LBPV-28396 | FUPR129-09 | HM144067 |
| Characiformes | Characidae | *Piabina argentea* | -21,0904007 | -50,57160187 | LBPV-28397 | FUPR130-09 | HM144066 |
| Characiformes | Characidae | *Piabina argentea* | -23,77099991 | -46,76599884 | LBPV-29239 | FUPR199-09 | HM144058 |
| Characiformes | Characidae | *Piabina argentea* | -23,77099991 | -46,76599884 | LBPV-29240 | FUPR200-09 | HM144057 |
| Characiformes | Characidae | *Piabina argentea* | -23,77099991 | -46,76599884 | LBPV-29280 | FUPR201-09 | HM144056 |
| Characiformes | Characidae | *Piabina argentea* | -23,77099991 | -46,76599884 | LBPV-29281 | FUPR202-09 | HM144055 |
| Characiformes | Characidae | *Piabina argentea* | -23,77099991 | -46,76599884 | LBPV-29282 | FUPR203-09 | HM144054 |
| Characiformes | Characidae | *Piabina argentea* | -15,11470032 | -47,04610062 | LBPV-31605 | FUPR110-09 | HM144083 |
| Characiformes | Characidae | *Piabina argentea* | -17,09939957 | -48,76150131 | LBPV-31606 | FUPR111-09 | HM144082 |
| Characiformes | Characidae | *Piabina argentea* | -17,09939957 | -48,76150131 | LBPV-31607 | FUPR112-09 | HM144081 |
| Characiformes | Characidae | *Piabina argentea* | -17,1196003 | -48,73989868 | LBPV-31608 | FUPR113-09 | HM144080 |
| Characiformes | Characidae | *Piabina argentea* | -17,1196003 | -48,73989868 | LBPV-31609 | FUPR114-09 | HM144079 |
| Characiformes | Characidae | *Piabina argentea* | -21,0904007 | -50,57160187 | LBPV-34659 | FUPR181-09 | HM144065 |
| Characiformes | Characidae | *Piabina argentea* | -21,28700066 | -50,13999939 | LBPV-34763 | FUPR183-09 | HM144064 |
| Characiformes | Characidae | *Piabina argentea* | -23,66600037 | -46,18299866 | LBPV-35848 | FUPR190-09 | HM144062 |
| Characiformes | Characidae | *Piabina argentea* | -23,66699982 | -46,18299866 | LBPV-35849 | FUPR191-09 | HM144061 |
| Characiformes | Characidae | *Piabina argentea* | -23,66699982 | -46,18299866 | LBPV-35870 | FUPR192-09 | HM144060 |
| Characiformes | Characidae | *Piabina argentea* | -23,66699982 | -46,18299866 | LBPV-35871 | FUPR193-09 | HM144059 |
| Characiformes | Characidae | *Piabina argentea* | -21,32110023 | -46,51129913 | LBPV-35904 | FUPR115-09 | HM144078 |
| Characiformes | Characidae | *Piabina argentea* | -21,32110023 | -46,51129913 | LBPV-35905 | FUPR116-09 | HM144077 |
| Characiformes | Characidae | *Piabina argentea* | -21,28520012 | -46,49330139 | LBPV-35906 | FUPR117-09 | HM144076 |
| Characiformes | Characidae | *Piabina argentea* | -21,28520012 | -46,49330139 | LBPV-35933 | FUPR118-09 | HM144075 |
| Characiformes | Characidae | *Piabina argentea* | -21,28520012 | -46,49330139 | LBPV-35934 | FUPR119-09 | HM144074 |
| Characiformes | Characidae | *Piabina argentea* | -22,78560066 | -48,48149872 | LBPV-36233 | FUPR120-09 | HM144073 |
| Characiformes | Characidae | *Piabina argentea* | -22,78560066 | -48,48149872 | LBPV-36234 | FUPR121-09 | HM144072 |
| Characiformes | Characidae | *Piabina argentea* | -23,5237999 | -45,88959885 | LBPV-36235 | FUPR122-09 | HM144071 |
| Characiformes | Characidae | *Piaractus mesopotamicus* | -22,79140091 | -53,34939957 | LBPV-23804 | FUPR434-09 | GU701416 |
| Characiformes | Characidae | *Piaractus mesopotamicus* | -22,79140091 | -53,34939957 | LBPV-23805 | FUPR435-09 | GU701429 |
| Characiformes | Characidae | *Piaractus mesopotamicus* | -22,79140091 | -53,34939957 | LBPV-23806 | FUPR436-09 | GU701417 |
| Characiformes | Characidae | *Piaractus mesopotamicus* | -22,79140091 | -53,34939957 | LBPV-23807 | FUPR437-09 | GU701418 |
| Characiformes | Characidae | *Piaractus mesopotamicus* | -22,79140091 | -53,34939957 | LBPV-23808 | FUPR438-09 | GU701428 |
| Characiformes | Characidae | *Planaltina britskii* | -22,79140091 | -53,34939957 | LBPV-17244 | FUPR005-09 | JN989130 |
| Characiformes | Characidae | *Planaltina britskii* | -22,79140091 | -53,34939957 | LBPV-17245 | FUPR006-09 | JN989131 |
| Characiformes | Characidae | *Planaltina britskii* | -22,79140091 | -53,34939957 | LBPV-17246 | FUPR007-09 | JN989132 |
| Characiformes | Characidae | *Planaltina britskii* | -22,79100037 | -53,34899902 | LBPV-17247 | FUPR008-09 | JN989133 |
| Characiformes | Characidae | *Psellogrammus kennedyi* | -22,43090057 | -47,69670105 | LBPV-26407 | FUPR014-09 | JN989169 |
| Characiformes | Characidae | *Psellogrammus kennedyi* | -22,3784008 | -47,21049881 | LBPV-27408 | FUPR015-09 | JN989170 |
| Characiformes | Characidae | *Psellogrammus kennedyi* | -22,3784008 | -47,21049881 | LBPV-31814 | FUPR016-09 | JN989171 |
| Characiformes | Characidae | *Psellogrammus kennedyi* | -18,27140045 | -52,84289932 | LBPV-31815 | FUPR017-09 | JN989172 |
| Characiformes | Characidae | *Psellogrammus kennedyi* | -18,27140045 | -52,84289932 | LBPV-31816 | FUPR132-09 | JN989173 |
| Characiformes | Characidae | *Pseudocorynopoma heterandria* | -18,27140045 | -52,84289932 | LBPV-21418 | FUPR324-09 | GU701559 |
| Characiformes | Characidae | *Pseudocorynopoma heterandria* | -18,27140045 | -52,84289932 | LBPV-40240 | FUPR979-09 | GU701920 |
| Characiformes | Characidae | *Pseudocorynopoma heterandria* | -18,56889915 | -52,08560181 | LBPV-40241 | FUPR980-09 | GU701921 |
| Characiformes | Characidae | *Pseudocorynopoma heterandria* | -18,56889915 | -52,08560181 | LBPV-40242 | FUPR981-09 | GU701917 |
| Characiformes | Characidae | *Pseudocorynopoma heterandria* | -18,56889915 | -52,08560181 | LBPV-40243 | FUPR982-09 | GU701918 |
| Characiformes | Characidae | *Pseudocorynopoma heterandria* | - | - | LBPV-40244 | FUPR983-09 | GU701914 |
| Characiformes | Characidae | *Roeboides descalvadensis* | -21,93129921 | -47,375 | LBPV-42765 | FUPR1281-10 | JN989208 |
| Characiformes | Characidae | *Roeboides descalvadensis* | -21,93129921 | -47,375 | LBPV-42766 | FUPR1282-10 | JN989207 |
| Characiformes | Characidae | *Roeboides descalvadensis* | -21,93129921 | -47,375 | LBPV-42767 | FUPR1283-10 | JN989206 |
| Characiformes | Characidae | *Roeboides descalvadensis* | - | - | LBPV-42769 | FUPR1284-10 | JN989205 |
| Characiformes | Characidae | *Roeboides descalvadensis* | -21,32699966 | -47,23899841 | LBPV-43163 | FUPR1285-10 | JN989204 |
| Characiformes | Characidae | *Roeboides descalvadensis* | -21,32699966 | -47,23899841 | LBPV-43164 | FUPR1286-10 | JN989203 |
| Characiformes | Characidae | *Roeboides descalvadensis* | -21,32699966 | -47,23899841 | LBPV-43165 | FUPR1287-10 | JN989202 |
| Characiformes | Characidae | *Roeboides descalvadensis* | -21,32699966 | -47,23899841 | LBPV-43166 | FUPR1288-10 | JN989201 |
| Characiformes | Characidae | *Roeboides descalvadensis* | -22,19400024 | -46,37900162 | LBPV-43167 | FUPR1289-10 | JN989200 |
| Characiformes | Characidae | *Roeboides descalvadensis* | -22,19400024 | -46,37900162 | LBPV-42768 | FUPR1375-10 | JN989199 |
| Characiformes | Characidae | *Serrapinnus kriegi* | -22,7689991 | -45,99000168 | LBPV-31991 | FUPR444-09 | HM422471 |
| Characiformes | Characidae | *Serrapinnus notomelas* | -22,7689991 | -45,99000168 | LBPV-17249 | FUPR137-09 | JN989224 |
| Characiformes | Characidae | *Serrapinnus notomelas* | -22,7689991 | -45,99000168 | LBPV-17250 | FUPR138-09 | JN989225 |
| Characiformes | Characidae | *Serrapinnus sp1* | -22,7689991 | -45,99000168 | LBPV-19539 | FUPR139-09 | JN989226 |
| Characiformes | Characidae | *Serrapinnus sp2* | -22,76399994 | -45,97600174 | LBPV-26123 | FUPR252-09 | JN989227 |
| Characiformes | Characidae | *New genus under description* | -21,10700035 | -50,26459885 | LBPV-33197 | FUPR311-09 | JN988883 |
| Characiformes | Characidae | *New genus under description* | -22,71759987 | -53,29100037 | LBPV-33198 | FUPR312-09 | GU701752 |
| Characiformes | Characidae | *New genus under description* | -22,71759987 | -53,29100037 | LBPV-33199 | FUPR313-09 | GU701754 |
| Characiformes | Characidae | *New genus under description* | -23,02429962 | -48,8280983 | LBPV-33200 | FUPR314-09 | GU701753 |
| Characiformes | Characidae | *New genus under description* | -23,02429962 | -48,8280983 | LBPV-34937b | FUPR978-09 | GU701952 |
| Characiformes | Crenuchidae | *Characidium aff. zebra* | -21,10700035 | -50,26459885 | LBPV-31162 | FUPR462-09 | GU701969 |
| Characiformes | Crenuchidae | *Characidium aff. zebra* | -22,90430069 | -50,00109863 | LBPV-31216 | FUPR463-09 | GU701970 |
| Characiformes | Crenuchidae | *Characidium aff. zebra* | -21,10700035 | -50,26459885 | LBPV-31238 | FUPR461-09 | GU701971 |
| Characiformes | Crenuchidae | *Characidium gomesi* | -21,34690094 | -47,6332016 | LBPV-10043 | FUPR348-09 | JN988782 |
| Characiformes | Crenuchidae | *Characidium gomesi* | -23,64159966 | -51,85919952 | LBPV-12126 | FUPR342-09 | GU701839 |
| Characiformes | Crenuchidae | *Characidium gomesi* | -23,93829918 | -50,72900009 | LBPV-28408 | FUPR208-09 | JN988781 |
| Characiformes | Crenuchidae | *Characidium gomesi* | -17,09939957 | -48,76150131 | LBPV-33568 | FUPR448-09 | GU701424 |
| Characiformes | Crenuchidae | *Characidium gomesi* | -17,09939957 | -48,76150131 | LBPV-33569 | FUPR447-09 | GU701427 |
| Characiformes | Crenuchidae | *Characidium gomesi* | -22,67340088 | -53,08580017 | LBPV-33572 | FUPR450-09 | GU701422 |
| Characiformes | Crenuchidae | *Characidium gomesi* | -21,34690094 | -47,6332016 | LBPV-33592 | FUPR446-09 | GU701426 |
| Characiformes | Crenuchidae | *Characidium gomesi* | -17,09939957 | -48,76150131 | LBPV-33607 | FUPR449-09 | GU701425 |
| Characiformes | Crenuchidae | *Characidium gomesi* | -21,74729919 | -46,46699905 | LBPV-41988 | FUPR1278-10 | JN988780 |
| Characiformes | Crenuchidae | *Characidium gomesi* | -20,43350029 | -51,26139832 | LBPV-41989 | FUPR1279-10 | JN988779 |
| Characiformes | Crenuchidae | *Characidium oiticicai* | -20,43350029 | -51,26139832 | LBPV-31207 | FUPR456-09 | GU701448 |
| Characiformes | Crenuchidae | *Characidium oiticicai* | -20,23609924 | -48,66719818 | LBPV-31212 | FUPR457-09 | GU701449 |
| Characiformes | Crenuchidae | *Characidium oiticicai* | -20,43350029 | -51,26139832 | LBPV-31213 | FUPR458-09 | GU701446 |
| Characiformes | Crenuchidae | *Characidium oiticicai* | -22,79140091 | -53,34939957 | LBPV-31214 | FUPR459-09 | GU701447 |
| Characiformes | Crenuchidae | *Characidium oiticicai* | -23,01280022 | -47,99769974 | LBPV-31218 | FUPR460-09 | GU701445 |
| Characiformes | Crenuchidae | *Characidium schubarti* | -21,97450066 | -47,41820145 | LBPV-31492 | FUPR455-09 | GU701440 |
| Characiformes | Crenuchidae | *Characidium schubarti* | -21,97450066 | -47,41820145 | LBPV-31498 | FUPR454-09 | GU701439 |
| Characiformes | Crenuchidae | *Characidium schubarti* | -23,01280022 | -47,99769974 | LBPV-33512 | FUPR451-09 | GU701437 |
| Characiformes | Crenuchidae | *Characidium schubarti* | -22,6692009 | -53,09289932 | LBPV-33513 | FUPR452-09 | GU701442 |
| Characiformes | Crenuchidae | *Characidium schubarti* | -22,67340088 | -53,08580017 | LBPV-33526 | FUPR453-09 | GU701441 |
| Characiformes | Crenuchidae | *Characidium sp.* | -18,42449951 | -48,06529999 | LBPV-41974 | FUPR1274-10 | JN988787 |
| Characiformes | Crenuchidae | *Characidium sp.* | -18,42449951 | -48,06529999 | LBPV-41975 | FUPR1275-10 | JN988786 |
| Characiformes | Crenuchidae | *Characidium sp.* | -21,32699966 | -47,23880005 | LBPV-41981 | FUPR1276-10 | JN988785 |
| Characiformes | Crenuchidae | *Characidium sp.* | -21,32699966 | -47,23880005 | LBPV-41982 | FUPR1277-10 | JN988784 |
| Characiformes | Crenuchidae | *Characidium sp.* | -21,97450066 | -47,41820145 | LBPV-41983 | FUPR1280-10 | JN988783 |
| Characiformes | Crenuchidae | *Characidium xanthopterum* | -22,79140091 | -53,34939957 | LBPV-34895 | FUPR344-09 | GU701836 |
| Characiformes | Crenuchidae | *Characidium xanthopterum* | -21,62400055 | -47,80599976 | LBPV-34896 | FUPR345-09 | GU701835 |
| Characiformes | Crenuchidae | *Characidium xanthopterum* | -25,4197998 | -54,53559875 | LBPV-34897 | FUPR346-09 | GU701834 |
| Characiformes | Crenuchidae | *Characidium xanthopterum* | -25,4197998 | -54,53559875 | LBPV-34898 | FUPR347-09 | GU701837 |
| Characiformes | Crenuchidae | *Characidium xanthopterum* | -22,79140091 | -53,34939957 | LBPV-35890 | FUPR343-09 | GU701833 |
| Characiformes | Crenuchidae | *Characidium zebra* | -25,4197998 | -54,53559875 | LBPV-19560 | FUPR145-09 | JN988788 |
| Characiformes | Crenuchidae | *Characidium zebra* | -25,4197998 | -54,53559875 | LBPV-19561 | FUPR146-09 | JN988789 |
| Characiformes | Crenuchidae | *Characidium zebra* | -25,4197998 | -54,53559875 | LBPV-19562 | FUPR147-09 | JN988790 |
| Characiformes | Curimatidae | *Cyphocharax modestus* | -17,77059937 | -48,65570068 | LBPV-19391 | FUPR153-09 | JN988837 |
| Characiformes | Curimatidae | *Cyphocharax modestus* | -19,6154995 | -47,44979858 | LBPV-19392 | FUPR154-09 | JN988838 |
| Characiformes | Curimatidae | *Cyphocharax modestus* | -22,99040031 | -48,42720032 | LBPV-25948 | FUPR152-09 | JN988836 |
| Characiformes | Curimatidae | *Cyphocharax modestus* | -19,6154995 | -47,44979858 | LBPV-32082 | FUPR349-09 | GU701805 |
| Characiformes | Curimatidae | *Cyphocharax modestus* | -19,6154995 | -47,44979858 | LBPV-32083 | FUPR350-09 | GU701806 |
| Characiformes | Curimatidae | *Cyphocharax modestus* | -19,6154995 | -47,44979858 | LBPV-32084 | FUPR351-09 | HM899912 |
| Characiformes | Curimatidae | *Cyphocharax modestus* | -22,9666996 | -48,40000153 | LBPV-32085 | FUPR352-09 | GU701808 |
| Characiformes | Curimatidae | *Cyphocharax modestus* | -22,6692009 | -53,09289932 | LBPV-32086 | FUPR353-09 | GU701807 |
| Characiformes | Curimatidae | *Cyphocharax nagelii* | -22,67340088 | -53,08580017 | LBPV-15935 | FUPR160-09 | JN988844 |
| Characiformes | Curimatidae | *Cyphocharax nagelii* | -22,67340088 | -53,08580017 | LBPV-15936 | FUPR161-09 | JN988845 |
| Characiformes | Curimatidae | *Cyphocharax nagelii* | -22,6692009 | -53,09289932 | LBPV-31729 | FUPR156-09 | JN988840 |
| Characiformes | Curimatidae | *Cyphocharax nagelii* | -22,6692009 | -53,09289932 | LBPV-31730 | FUPR157-09 | JN988841 |
| Characiformes | Curimatidae | *Cyphocharax nagelii* | -22,6692009 | -53,09289932 | LBPV-31731 | FUPR158-09 | JN988842 |
| Characiformes | Curimatidae | *Cyphocharax nagelii* | -22,67340088 | -53,08580017 | LBPV-31732 | FUPR159-09 | JN988843 |
| Characiformes | Curimatidae | *Cyphocharax nagelii* | -22,6692009 | -53,09289932 | LBPV-31915 | FUPR155-09 | JN988839 |
| Characiformes | Curimatidae | *Cyphocharax vanderi* | -22,67340088 | -53,08580017 | LBPV-17372 | FUPR148-09 | JN988846 |
| Characiformes | Curimatidae | *Cyphocharax vanderi* | -22,67340088 | -53,08580017 | LBPV-17373 | FUPR149-09 | JN988847 |
| Characiformes | Curimatidae | *Cyphocharax vanderi* | -22,67340088 | -53,08580017 | LBPV-22449 | FUPR150-09 | JN988848 |
| Characiformes | Curimatidae | *Cyphocharax vanderi* | -22,86910057 | -48,36759949 | LBPV-22450 | FUPR151-09 | JN988849 |
| Characiformes | Curimatidae | *Cyphocharax vanderi* | -22,86910057 | -48,36759949 | LBPV-37587 | FUPR379-09 | GU701800 |
| Characiformes | Curimatidae | *Cyphocharax vanderi* | -22,78560066 | -48,48149872 | LBPV-37588 | FUPR380-09 | GU701799 |
| Characiformes | Curimatidae | *Cyphocharax vanderi* | -22,67589951 | -45,68289948 | LBPV-37589 | FUPR381-09 | GU701803 |
| Characiformes | Curimatidae | *Cyphocharax vanderi* | -22,79140091 | -53,34939957 | LBPV-37590 | FUPR382-09 | GU701802 |
| Characiformes | Curimatidae | *Cyphocharax vanderi* | -22,79140091 | -53,34939957 | LBPV-37591 | FUPR383-09 | GU701804 |
| Characiformes | Curimatidae | *Steindachnerina brevipinna* | -22,98999977 | -48,42699814 | LBPV-32153 | FUPR354-09 | GU701503 |
| Characiformes | Curimatidae | *Steindachnerina brevipinna* | -22,98999977 | -48,42699814 | LBPV-32154 | FUPR355-09 | GU701502 |
| Characiformes | Curimatidae | *Steindachnerina brevipinna* | -22,98999977 | -48,42699814 | LBPV-32155 | FUPR356-09 | GU701501 |
| Characiformes | Curimatidae | *Steindachnerina brevipinna* | -22,98999977 | -48,42699814 | LBPV-32156 | FUPR357-09 | GU701505 |
| Characiformes | Curimatidae | *Steindachnerina brevipinna* | -22,98999977 | -48,42699814 | LBPV-32157 | FUPR358-09 | GU701504 |
| Characiformes | Curimatidae | *Steindachnerina insculpta* | -23,46800041 | -51,31499863 | LBPV-19389 | FUPR217-09 | JN989237 |
| Characiformes | Curimatidae | *Steindachnerina insculpta* | -23,68199921 | -51,31499863 | LBPV-19390 | FUPR218-09 | JN989238 |
| Characiformes | Curimatidae | *Steindachnerina insculpta* | -23,46800041 | -51,31499863 | LBPV-26343 | FUPR216-09 | JN989236 |
| Characiformes | Curimatidae | *Steindachnerina insculpta* | -23,65600014 | -51,24300003 | LBPV-32081 | FUPR219-09 | JN989239 |
| Characiformes | Curimatidae | *Steindachnerina insculpta* | -17,49099922 | -48,36999893 | LBPV-32158 | FUPR359-09 | GU701506 |
| Characiformes | Cynodontidae | *Rhaphiodon vulpinus* | -18,5807991 | -52,09799957 | LBPV-21832 | FUPR384-09 | GU701527 |
| Characiformes | Cynodontidae | *Rhaphiodon vulpinus* | -19,23080063 | -52,98559952 | LBPV-21844 | FUPR385-09 | GU701530 |
| Characiformes | Cynodontidae | *Rhaphiodon vulpinus* | -19,24570084 | -52,65299988 | LBPV-21845 | FUPR386-09 | GU701529 |
| Characiformes | Cynodontidae | *Rhaphiodon vulpinus* | -22,04380035 | -53,72740173 | LBPV-22942 | FUPR387-09 | GU701528 |
| Characiformes | Cynodontidae | *Rhaphiodon vulpinus* | -22,04380035 | -53,72740173 | LBPV-22943 | FUPR388-09 | JN989192 |
| Characiformes | Cynodontidae | *Rhaphiodon vulpinus* | -22,04380035 | -53,72740173 | LBPV-22944 | FUPR389-09 | GU701532 |
| Characiformes | Cynodontidae | *Rhaphiodon vulpinus* | -22,04380035 | -53,72740173 | LBPV-22945 | FUPR390-09 | GU701531 |
| Characiformes | Erythrinidae | *Erythrinus erythrinus* | -20,43350029 | -51,26139832 | LBPV-24835 | FUPR162-09 | JN988855 |
| Characiformes | Erythrinidae | *Erythrinus erythrinus* | -20,08539963 | -50,98310089 | LBPV-24836 | FUPR163-09 | JN988856 |
| Characiformes | Erythrinidae | *Erythrinus erythrinus* | -20,08539963 | -50,98310089 | LBPV-31955 | FUPR164-09 | JN988857 |
| Characiformes | Erythrinidae | *Erythrinus erythrinus* | -20,08539963 | -50,98310089 | LBPV-31956 | FUPR165-09 | JN988858 |
| Characiformes | Erythrinidae | *Hoplerythrinus unitaeniatus* | -22,79140091 | -53,34939957 | LBPV-26699 | FUPR249-09 | JN988903 |
| Characiformes | Erythrinidae | *Hoplerythrinus unitaeniatus* | -22,71759987 | -53,29100037 | LBPV-26700 | FUPR166-09 | JN988900 |
| Characiformes | Erythrinidae | *Hoplerythrinus unitaeniatus* | -22,79140091 | -53,34939957 | LBPV-26701 | FUPR250-09 | JN988904 |
| Characiformes | Erythrinidae | *Hoplerythrinus unitaeniatus* | -22,71759987 | -53,29100037 | LBPV-31940 | FUPR167-09 | JN988901 |
| Characiformes | Erythrinidae | *Hoplerythrinus unitaeniatus* | -22,71759987 | -53,29100037 | LBPV-31984 | FUPR168-09 | JN988902 |
| Characiformes | Erythrinidae | *Hoplias intermedius* | -23,64159966 | -51,85929871 | LBPV-21846 | FUPR209-09 | JN988905 |
| Characiformes | Erythrinidae | *Hoplias intermedius* | -23,93829918 | -50,72900009 | LBPV-21847 | FUPR210-09 | JN988906 |
| Characiformes | Erythrinidae | *Hoplias malabaricus* | -23,93829918 | -50,72900009 | LBPV-32184 | FUPR169-09 | JN988907 |
| Characiformes | Erythrinidae | *Hoplias malabaricus* | -23,93829918 | -50,72900009 | LBPV-32185 | FUPR170-09 | JN988908 |
| Characiformes | Erythrinidae | *Hoplias malabaricus* | -22,79140091 | -53,34939957 | LBPV-32186 | FUPR171-09 | JN988909 |
| Characiformes | Lebiasinidae | *Pyrrhulina australis* | -15,58170033 | -47,50640106 | LBPV-26301 | FUPR018-09 | JN989183 |
| Characiformes | Lebiasinidae | *Pyrrhulina australis* | -23,23060036 | -48,53279877 | LBPV-26302 | FUPR019-09 | JN989184 |
| Characiformes | Lebiasinidae | *Pyrrhulina australis* | -23,23060036 | -48,53279877 | LBPV-32189 | FUPR020-09 | JN989185 |
| Characiformes | Lebiasinidae | *Pyrrhulina australis* | -23,23060036 | -48,53279877 | LBPV-32190 | FUPR021-09 | JN989186 |
| Characiformes | Lebiasinidae | *Pyrrhulina australis* | -15,66730022 | -47,95240021 | LBPV-32191 | FUPR022-09 | JN989187 |
| Characiformes | Parodontidae | *Apareiodon affinis* | -23,57640076 | -45,97449875 | LBPV-20184 | FUPR172-09 | JN988667 |
| Characiformes | Parodontidae | *Apareiodon affinis* | -23,57640076 | -45,97449875 | LBPV-20185 | FUPR173-09 | JN988668 |
| Characiformes | Parodontidae | *Apareiodon affinis* | -23,57640076 | -45,97449875 | LBPV-20186 | FUPR174-09 | JN988669 |
| Characiformes | Parodontidae | *Apareiodon affinis* | -22,78560066 | -48,48149872 | LBPV-24664 | FUPR175-09 | JN988670 |
| Characiformes | Parodontidae | *Apareiodon affinis* | -23,51119995 | -45,85910034 | LBPV-25976 | FUPR402-09 | JN988672 |
| Characiformes | Parodontidae | *Apareiodon affinis* | -23,5237999 | -45,88959885 | LBPV-26375 | FUPR399-09 | JN988671 |
| Characiformes | Parodontidae | *Apareiodon affinis* | -23,52400017 | -45,88999939 | LBPV-26376 | FUPR400-09 | JN988674 |
| Characiformes | Parodontidae | *Apareiodon affinis* | -23,5237999 | -45,88959885 | LBPV-26377 | FUPR401-09 | JN988673 |
| Characiformes | Parodontidae | *Apareiodon ibitiensis* | -19,68300056 | -48,66899872 | LBPV-18631 | FUPR246-09 | JN988675 |
| Characiformes | Parodontidae | *Apareiodon ibitiensis* | -19,68300056 | -48,66899872 | LBPV-18632 | FUPR431-09 | GU701941 |
| Characiformes | Parodontidae | *Apareiodon ibitiensis* | -19,68300056 | -48,66899872 | LBPV-18633 | FUPR247-09 | JN988676 |
| Characiformes | Parodontidae | *Apareiodon ibitiensis* | -19,68300056 | -48,66899872 | LBPV-18634 | FUPR432-09 | GU701940 |
| Characiformes | Parodontidae | *Apareiodon ibitiensis* | -19,68300056 | -48,66899872 | LBPV-18635 | FUPR433-09 | GU701949 |
| Characiformes | Parodontidae | *Apareiodon piracicabae* | -19,68300056 | -48,66899872 | LBPV-34671 | FUPR398-09 | GU701864 |
| Characiformes | Parodontidae | *Parodon nasus* | -23,57640076 | -45,97449875 | LBPV-20439 | FUPR361-09 | GU701584 |
| Characiformes | Parodontidae | *Parodon nasus* | -23,57640076 | -45,97449875 | LBPV-21804 | FUPR363-09 | GU701587 |
| Characiformes | Parodontidae | *Parodon nasus* | -23,57640076 | -45,97449875 | LBPV-20440 | FUPR362-09 | GU701588 |
| Characiformes | Parodontidae | *Parodon nasus* | -23,57640076 | -45,97449875 | LBPV-37820 | FUPR364-09 | GU701591 |
| Characiformes | Prochilodontidae | *Prochilodus lineatus* | -23,93829918 | -50,72900009 | LBPV-26740 | FUPR009-09 | JN989161 |
| Characiformes | Prochilodontidae | *Prochilodus lineatus* | -23,93829918 | -50,72900009 | LBPV-26741 | FUPR010-09 | JN989162 |
| Characiformes | Prochilodontidae | *Prochilodus lineatus* | -23,67469978 | -52,1189003 | LBPV-29202 | FUPR011-09 | JN989163 |
| Characiformes | Prochilodontidae | *Prochilodus lineatus* | -23,67499924 | -52,11899948 | LBPV-31742 | FUPR012-09 | JN989164 |
| Characiformes | Prochilodontidae | *Prochilodus lineatus* | -22,34090042 | -48,93470001 | LBPV-31743 | FUPR013-09 | JN989165 |
| Characiformes | Serrasalmidae | *Metynnis maculatus* | - | - | LBPV-17332 | FUPR077-09 | JN989028 |
| Characiformes | Serrasalmidae | *Metynnis maculatus* | - | - | LBPV-17333 | FUPR078-09 | JN989029 |
| Characiformes | Serrasalmidae | *Metynnis maculatus* | - | - | LBPV-17334 | FUPR079-09 | JN989030 |
| Characiformes | Serrasalmidae | *Metynnis maculatus* | -21,37999916 | -46,47489929 | LBPV-20198 | FUPR080-09 | JN989031 |
| Characiformes | Serrasalmidae | *Metynnis maculatus* | -21,44440079 | -44,60240173 | LBPV-26350 | FUPR317-09 | GU701633 |
| Characiformes | Serrasalmidae | *Metynnis maculatus* | -21,37999916 | -46,47489929 | LBPV-26760 | FUPR316-09 | GU701630 |
| Characiformes | Serrasalmidae | *Metynnis maculatus* | -21,44440079 | -44,60240173 | LBPV-42792 | FUPR1033-10 | JN989027 |
| Characiformes | Serrasalmidae | *Metynnis maculatus* | -21,44440079 | -44,60240173 | LBPV-42793 | FUPR1034-10 | JN989026 |
| Characiformes | Serrasalmidae | *Metynnis maculatus* | -21,44440079 | -44,60240173 | LBPV-42795 | FUPR1035-10 | JN989025 |
| Characiformes | Serrasalmidae | *Serrasalmus maculatus* | -21,27400017 | -43,64400101 | LBPV-20168 | FUPR142-09 | JN989228 |
| Characiformes | Serrasalmidae | *Serrasalmus maculatus* | -21,27400017 | -43,64400101 | LBPV-20169 | FUPR143-09 | JN989229 |
| Characiformes | Serrasalmidae | *Serrasalmus maculatus* | -21,27400017 | -43,64400101 | LBPV-20170 | FUPR144-09 | JN989230 |
| Characiformes | Serrasalmidae | *Serrasalmus maculatus* | -20,64900017 | -46,16500092 | LBPV-26723 | FUPR372-09 | GU701512 |
| Characiformes | Serrasalmidae | *Serrasalmus maculatus* | -20,95599937 | -43,76300049 | LBPV-26724 | FUPR373-09 | GU701511 |
| Characiformes | Serrasalmidae | *Serrasalmus maculatus* | -20,64900017 | -46,16500092 | LBPV-31914 | FUPR368-09 | JN989231 |
| Characiformes | Serrasalmidae | *Serrasalmus marginatus* | -20,95599937 | -43,76300049 | LBPV-17335 | FUPR140-09 | JN989232 |
| Characiformes | Serrasalmidae | *Serrasalmus marginatus* | -20,95599937 | -43,76300049 | LBPV-17336 | FUPR141-09 | JN989233 |
| Characiformes | Serrasalmidae | *Serrasalmus marginatus* | -21,29400063 | -46,48500061 | LBPV-26725 | FUPR365-09 | JN989234 |
| Characiformes | Serrasalmidae | *Serrasalmus marginatus* | -21,29400063 | -46,48500061 | LBPV-26726 | FUPR366-09 | JN989235 |
| Characiformes | Serrasalmidae | *Serrasalmus marginatus* | -21,36199951 | -46,57699966 | LBPV-26727 | FUPR367-09 | GU701508 |
| Characiformes | Serrasalmidae | *Serrasalmus marginatus* | -21,37100029 | -46,54299927 | LBPV-31911 | FUPR369-09 | GU701507 |
| Characiformes | Serrasalmidae | *Serrasalmus marginatus* | -21,37100029 | -46,54299927 | LBPV-31912 | FUPR370-09 | GU701510 |
| Characiformes | Serrasalmidae | *Serrasalmus marginatus* | -24,10400009 | -52,32500076 | LBPV-31913 | FUPR371-09 | GU701509 |
| Characiformes | Triportheidae | *Triportheus nematurus* | -17,12000084 | -48,74000168 | LBPV-15175 | FUPR333-09 | GU701458 |
| Characiformes | Triportheidae | *Triportheus nematurus* | -17,12000084 | -48,74000168 | LBPV-15177 | FUPR334-09 | GU701457 |
| Characiformes | Triportheidae | *Triportheus nematurus* | -17,77099991 | -48,65599823 | LBPV-15178 | FUPR335-09 | GU701461 |
| Characiformes | Triportheidae | *Triportheus nematurus* | -17,77099991 | -48,65599823 | LBPV-15204 | FUPR336-09 | JN989280 |
| Characiformes | Triportheidae | *Triportheus nematurus* | -25,31900024 | -52,48899841 | LBPV-15205 | FUPR337-09 | JN989281 |
| Characiformes | Triportheidae | *Triportheus nematurus* | -25,31900024 | -52,48899841 | LBPV-31043 | FUPR338-09 | GU701945 |
| Characiformes | Triportheidae | *Triportheus nematurus* | -25,09199905 | -52,49499893 | LBPV-31046 | FUPR339-09 | JN989282 |
| Characiformes | Triportheidae | *Triportheus nematurus* | -25,09199905 | -52,49499893 | LBPV-31049 | FUPR340-09 | JN989283 |
| Characiformes | Triportheidae | *Triportheus nematurus* | -22,72960091 | -53,31779861 | LBPV-31050 | FUPR341-09 | JN989284 |
| Clupeiformes | Clupeidae | *Platanichthys platana* | -22,79100037 | -53,34899902 | LBPV-42001 | FUPR1028-10 | JN989138 |
| Clupeiformes | Clupeidae | *Platanichthys platana* | -19,38500023 | -43,65919876 | LBPV-42002 | FUPR1029-10 | JN989137 |
| Clupeiformes | Clupeidae | *Platanichthys platana* | -22,79140091 | -53,34939957 | LBPV-42003 | FUPR1030-10 | JN989136 |
| Clupeiformes | Clupeidae | *Platanichthys platana* | -21,36359978 | -46,47689819 | LBPV-42004 | FUPR1031-10 | JN989135 |
| Clupeiformes | Clupeidae | *Platanichthys platana* | -21,36359978 | -46,47689819 | LBPV-42005 | FUPR1032-10 | JN989134 |
| Cypriniformes | Cyprinidae | *Ctenopharyngodon idella* | -22,63979912 | -48,05310059 | LBPV-49525 | FUPR1365-10 | JN988835 |
| Cypriniformes | Cyprinidae | *Ctenopharyngodon idella* | -22,63979912 | -48,05310059 | LBPV-49526 | FUPR1366-10 | JN988834 |
| Cypriniformes | Cyprinidae | *Ctenopharyngodon idella* | -22,63979912 | -48,05310059 | LBPV-49527 | FUPR1367-10 | JN988833 |
| Cypriniformes | Cyprinidae | *Ctenopharyngodon idella* | -22,63979912 | -48,05310059 | LBPV-49528 | FUPR1368-10 | JN988832 |
| Cypriniformes | Cyprinidae | *Cyprinus carpio* | -22,79140091 | -53,34939957 | LBPV-45301 | FUPR1351-10 | JN988854 |
| Cypriniformes | Cyprinidae | *Cyprinus carpio* | -22,66049957 | -53,0848999 | LBPV-45302 | FUPR1352-10 | JN988853 |
| Cypriniformes | Cyprinidae | *Cyprinus carpio* | -22,66049957 | -53,0848999 | LBPV-45303 | FUPR1353-10 | JN988852 |
| Cypriniformes | Cyprinidae | *Cyprinus carpio* | -22,66049957 | -53,0848999 | LBPV-45304 | FUPR1354-10 | JN988851 |
| Cypriniformes | Cyprinidae | *Cyprinus carpio* | -22,66049957 | -53,0848999 | LBPV-45305 | FUPR1355-10 | JN988850 |
| Cypriniformes | Cyprinidae | *Hypophthalmichthys molitrix* | -22,93919945 | -50,25230026 | LBPV-49535 | FUPR1372-10 | JN988928 |
| Cypriniformes | Cyprinidae | *Hypophthalmichthys molitrix* | -22,93919945 | -50,25230026 | LBPV-49536 | FUPR1373-10 | JN988927 |
| Cypriniformes | Cyprinidae | *Hypophthalmichthys molitrix* | -22,93919945 | -50,25230026 | LBPV-49537 | FUPR1374-10 | JN988926 |
| Cypriniformes | Cyprinidae | *Hypophthalmichthys nobilis* | -22,93919945 | -50,25230026 | LBPV-49530 | FUPR1369-10 | JN988931 |
| Cypriniformes | Cyprinidae | *Hypophthalmichthys nobilis* | -21,0128994 | -49,69029999 | LBPV-49531 | FUPR1370-10 | JN988930 |
| Cypriniformes | Cyprinidae | *Hypophthalmichthys nobilis* | -21,0128994 | -49,69029999 | LBPV-49532 | FUPR1371-10 | JN988929 |
| Cyprinodontiformes | Poeciliidae | *Pamphorichthys hollandi* | -24,07349968 | -52,29109955 | LBPV-26439 | FUPR946-09 | GU701604 |
| Cyprinodontiformes | Poeciliidae | *Pamphorichthys hollandi* | -24,07349968 | -52,29109955 | LBPV-26440 | FUPR947-09 | GU701603 |
| Cyprinodontiformes | Poeciliidae | *Pamphorichthys hollandi* | -24,07349968 | -52,29109955 | LBPV-26441 | FUPR948-09 | GU701602 |
| Cyprinodontiformes | Poeciliidae | *Pamphorichthys hollandi* | -22,7220993 | -53,28649902 | LBPV-26442 | FUPR949-09 | GU701606 |
| Cyprinodontiformes | Poeciliidae | *Pamphorichthys hollandi* | -22,7220993 | -53,28649902 | LBPV-32102 | FUPR950-09 | GU701605 |
| Cyprinodontiformes | Poeciliidae | *Phalloceros harpagos* | -23,57640076 | -45,97449875 | LBPV-29755 | FUPR951-09 | GU701579 |
| Cyprinodontiformes | Poeciliidae | *Phalloceros harpagos* | -23,24180031 | -51,21829987 | LBPV-29983 | FUPR952-09 | GU701583 |
| Cyprinodontiformes | Poeciliidae | *Phalloceros harpagos* | -23,24180031 | -51,21829987 | LBPV-29984 | FUPR953-09 | GU701582 |
| Cyprinodontiformes | Poeciliidae | *Phalloceros harpagos* | -23,24180031 | -51,21829987 | LBPV-29985 | FUPR954-09 | GU701586 |
| Cyprinodontiformes | Poeciliidae | *Phalloceros harpagos* | -23,57640076 | -45,97449875 | LBPV-37497 | FUPR938-09 | GU701581 |
| Cyprinodontiformes | Poeciliidae | *Phalloceros harpagos* | -23,57640076 | -45,97449875 | LBPV-37498 | FUPR939-09 | GU701580 |
| Cyprinodontiformes | Poeciliidae | *Phalloceros harpagos* | -23,24180031 | -51,21829987 | LBPV-37500 | FUPR955-09 | GU701585 |
| Cyprinodontiformes | Poeciliidae | *Phalloceros harpagos* | -23,24180031 | -51,21829987 | LBPV-44523 | FUPR1296-10 | JN989120 |
| Cyprinodontiformes | Poeciliidae | *Phalloceros harpagos* | -22,63470078 | -52,82210159 | LBPV-44589 | FUPR1310-10 | JN989119 |
| Cyprinodontiformes | Poeciliidae | *Phalloceros harpagos* | -22,63470078 | -52,82210159 | LBPV-44759 | FUPR1324-10 | JN989118 |
| Cyprinodontiformes | Poeciliidae | *Phalloceros harpagos* | -22,63470078 | -52,82210159 | LBPV-44760 | FUPR1325-10 | JN989117 |
| Cyprinodontiformes | Poeciliidae | *Phalloceros reisi* | -22,63470078 | -52,82210159 | LBPV-40245 | FUPR989-09 | GU701910 |
| Cyprinodontiformes | Poeciliidae | *Phalloceros reisi* | -22,63470078 | -52,82210159 | LBPV-43526 | FUPR1292-10 | JN989121 |
| Cyprinodontiformes | Poeciliidae | *Poecilia reticulata* | -23,69190025 | -51,32540131 | LBPV-40035 | FUPR1271-10 | JN989142 |
| Cyprinodontiformes | Poeciliidae | *Poecilia reticulata* | -23,69190025 | -51,32540131 | LBPV-44559 | FUPR1302-10 | JN989141 |
| Cyprinodontiformes | Poeciliidae | *Poecilia reticulata* | -23,75720024 | -51,24890137 | LBPV-44560 | FUPR1303-10 | JN989140 |
| Cyprinodontiformes | Poeciliidae | *Poecilia reticulata* | -22,61389923 | -45,51440048 | LBPV-44561 | FUPR1304-10 | JN989139 |
| Cyprinodontiformes | Poeciliidae | *Poecilia vivipara* | -22,61389923 | -45,51440048 | LBPV-40246 | FUPR990-09 | GU701911 |
| Cyprinodontiformes | Poeciliidae | *Poecilia vivipara* | -23,67589951 | -51,31399918 | LBPV-40465 | FUPR991-09 | GU701907 |
| Cyprinodontiformes | Poeciliidae | *Poecilia vivipara* | -23,67589951 | -51,31399918 | LBPV-40466 | FUPR992-09 | GU701908 |
| Cyprinodontiformes | Poeciliidae | *Poecilia vivipara* | -24,0781002 | -52,29629898 | LBPV-40467 | FUPR993-09 | GU701904 |
| Cyprinodontiformes | Rivulidae | *Atlantirivulus santensis* | -23,77029991 | -46,31100082 | LBPV-36309 | FUPR930-09 | GU701515 |
| Cyprinodontiformes | Rivulidae | *Atlantirivulus santensis* | -21,62409973 | -47,80630112 | LBPV-40281 | FUPR1021-09 | GU701924 |
| Cyprinodontiformes | Rivulidae | *Atlantirivulus santensis* | -21,62409973 | -47,80630112 | LBPV-40282 | FUPR1022-09 | GU701925 |
| Cyprinodontiformes | Rivulidae | *Atlantirivulus santensis* | -21,34690094 | -47,6332016 | LBPV-40283 | FUPR1023-09 | GU701922 |
| Cyprinodontiformes | Rivulidae | *Atlantirivulus santensis* | -23,77029991 | -46,31100082 | LBPV-40284 | FUPR1024-09 | GU701923 |
| Cyprinodontiformes | Rivulidae | *Atlantirivulus santensis* | -23,77029991 | -46,31100082 | LBPV-40285 | FUPR1025-09 | GU701919 |
| Cyprinodontiformes | Rivulidae | *Melanorivulus apiamici* | - | - | LBPV-31953 | FUPR936-09 | GU701519 |
| Cyprinodontiformes | Rivulidae | *Melanorivulus apiamici* | - | - | LBPV-31954 | FUPR937-09 | GU701518 |
| Gymnotiformes | Gymnotidae | *Gymnotus inaequilabiatus* | -23,02429962 | -48,8280983 | LBPV-25850 | FUPR904-09 | GU701760 |
| Gymnotiformes | Gymnotidae | *Gymnotus inaequilabiatus* | -23,02429962 | -48,8280983 | LBPV-26331 | FUPR903-09 | GU701766 |
| Gymnotiformes | Gymnotidae | *Gymnotus inaequilabiatus* | -23,02429962 | -48,8280983 | LBPV-31794 | FUPR908-09 | GU701761 |
| Gymnotiformes | Gymnotidae | *Gymnotus inaequilabiatus* | -15,72850037 | -47,91090012 | LBPV-31930 | FUPR899-09 | GU701765 |
| Gymnotiformes | Gymnotidae | *Gymnotus inaequilabiatus* | -23,02429962 | -48,8280983 | LBPV-31931 | FUPR900-09 | GU701764 |
| Gymnotiformes | Gymnotidae | *Gymnotus inaequilabiatus* | -22,64789963 | -53,07860184 | LBPV-34744 | FUPR881-09 | GU701781 |
| Gymnotiformes | Gymnotidae | *Gymnotus pantanal* | -25,29630089 | -49,74890137 | LBPV-31927 | FUPR891-09 | GU701773 |
| Gymnotiformes | Gymnotidae | *Gymnotus pantanal* | -25,29630089 | -49,74890137 | LBPV-31928 | FUPR892-09 | GU701777 |
| Gymnotiformes | Gymnotidae | *Gymnotus pantanal* | -25,29630089 | -49,74890137 | LBPV-31929 | FUPR893-09 | GU701776 |
| Gymnotiformes | Gymnotidae | *Gymnotus pantanal* | -25,29630089 | -49,74890137 | LBPV-31932 | FUPR901-09 | GU701763 |
| Gymnotiformes | Gymnotidae | *Gymnotus pantanal* | -23,02429962 | -48,8280983 | LBPV-34742 | FUPR889-09 | GU701775 |
| Gymnotiformes | Gymnotidae | *Gymnotus pantanal* | -25,29630089 | -49,74890137 | LBPV-34743 | FUPR890-09 | GU701774 |
| Gymnotiformes | Gymnotidae | *Gymnotus pantherinus* | -23,51119995 | -45,85910034 | LBPV-19571 | FUPR894-09 | GU701770 |
| Gymnotiformes | Gymnotidae | *Gymnotus pantherinus* | -23,51119995 | -45,85910034 | LBPV-22560 | FUPR895-09 | GU701769 |
| Gymnotiformes | Gymnotidae | *Gymnotus pantherinus* | -23,51119995 | -45,85910034 | LBPV-22561 | FUPR896-09 | GU701768 |
| Gymnotiformes | Gymnotidae | *Gymnotus pantherinus* | -23,51119995 | -45,85910034 | LBPV-22562 | FUPR897-09 | GU701772 |
| Gymnotiformes | Gymnotidae | *Gymnotus pantherinus* | - | - | LBPV-24532 | FUPR898-09 | GU701771 |
| Gymnotiformes | Gymnotidae | *Gymnotus sp.* | -23,52400017 | -45,88999939 | LBPV-31959 | FUPR885-09 | HM376402 |
| Gymnotiformes | Gymnotidae | *Gymnotus sylvius* | -24,07349968 | -52,29109955 | LBPV-25851 | FUPR905-09 | GU701759 |
| Gymnotiformes | Gymnotidae | *Gymnotus sylvius* | -24,07349968 | -52,29109955 | LBPV-25852 | FUPR906-09 | GU701758 |
| Gymnotiformes | Gymnotidae | *Gymnotus sylvius* | -24,07349968 | -52,29109955 | LBPV-25853 | FUPR907-09 | GU701762 |
| Gymnotiformes | Gymnotidae | *Gymnotus sylvius* | -22,44890022 | -45,34640121 | LBPV-27380 | FUPR886-09 | GU701778 |
| Gymnotiformes | Gymnotidae | *Gymnotus sylvius* | -22,44890022 | -45,34640121 | LBPV-27381 | FUPR887-09 | JN988880 |
| Gymnotiformes | Gymnotidae | *Gymnotus sylvius* | -22,94140053 | -48,58420181 | LBPV-27382 | FUPR888-09 | JN988881 |
| Gymnotiformes | Gymnotidae | *Gymnotus sylvius* | -22,94140053 | -48,58420181 | LBPV-31933 | FUPR902-09 | GU701767 |
| Gymnotiformes | Gymnotidae | *Gymnotus sylvius* | -22,44890022 | -45,34640121 | LBPV-31958 | FUPR884-09 | GU701779 |
| Gymnotiformes | Gymnotidae | *Gymnotus sylvius* | -23,52400017 | -45,88999939 | LBPV-8831 | FUPR883-09 | GU701782 |
| Gymnotiformes | Gymnotidae | *Gymnotus sylvius* | -23,52400017 | -45,88999939 | LBPV-9823 | FUPR882-09 | GU701780 |
| Gymnotiformes | Hypopomidae | *Brachyhypopomus pinnicaudatus* | -23,02429962 | -48,8280983 | LBPV-32096 | FUPR912-09 | GU701852 |
| Gymnotiformes | Hypopomidae | *Brachyhypopomus pinnicaudatus* | -22,64710045 | -53,07690048 | LBPV-32097 | FUPR913-09 | GU701851 |
| Gymnotiformes | Hypopomidae | *Brachyhypopomus pinnicaudatus* | -23,14999962 | -48,26670074 | LBPV-32353 | FUPR909-09 | GU701847 |
| Gymnotiformes | Hypopomidae | *Brachyhypopomus pinnicaudatus* | -23,14999962 | -48,26670074 | LBPV-32354 | FUPR910-09 | GU701849 |
| Gymnotiformes | Hypopomidae | *Brachyhypopomus pinnicaudatus* | -23,52669907 | -45,81980133 | LBPV-32355 | FUPR911-09 | GU701848 |
| Gymnotiformes | Rhamphichthyidae | *Gymnorhamphichthys britskii* | -17,69510078 | -40,76979828 | LBPV-45898 | FUPR1362-10 | JN988879 |
| Gymnotiformes | Rhamphichthyidae | *Gymnorhamphichthys britskii* | -21,97450066 | -47,41820145 | LBPV-45899 | FUPR1363-10 | JN988878 |
| Gymnotiformes | Rhamphichthyidae | *Rhamphichthys hahni* | -18,40600014 | -48,07899857 | LBPV-19226 | FUPR967-09 | JN989191 |
| Gymnotiformes | Rhamphichthyidae | *Rhamphichthys hahni* | -18,27140045 | -52,84289932 | LBPV-19227 | FUPR968-09 | HM899914 |
| Gymnotiformes | Rhamphichthyidae | *Rhamphichthys hahni* | -15,73550034 | -47,91859818 | LBPV-19738 | FUPR964-09 | GU701535 |
| Gymnotiformes | Rhamphichthyidae | *Rhamphichthys hahni* | -15,73550034 | -47,91859818 | LBPV-19739 | FUPR965-09 | GU701534 |
| Gymnotiformes | Rhamphichthyidae | *Rhamphichthys hahni* | -15,74699974 | -47,95050049 | LBPV-19740 | FUPR966-09 | GU701533 |
| Gymnotiformes | Rhamphichthyidae | *Rhamphichthys hahni* | -18,5807991 | -52,09799957 | LBPV-44992 | FUPR1350-10 | JN989190 |
| Gymnotiformes | Sternopygidae | *Eigenmannia trilineata* | -22,79140091 | -53,34939957 | LBPV-24743 | FUPR914-09 | GU701795 |
| Gymnotiformes | Sternopygidae | *Eigenmannia trilineata* | -22,79140091 | -53,34939957 | LBPV-24744 | FUPR915-09 | GU701794 |
| Gymnotiformes | Sternopygidae | *Eigenmannia trilineata* | -22,91139984 | -48,48789978 | LBPV-24745 | FUPR916-09 | GU701798 |
| Gymnotiformes | Sternopygidae | *Eigenmannia trilineata* | -22,91139984 | -48,48789978 | LBPV-24746 | FUPR917-09 | GU701797 |
| Gymnotiformes | Sternopygidae | *Eigenmannia trilineata* | -22,91139984 | -48,48789978 | LBPV-24747 | FUPR918-09 | GU701801 |
| Gymnotiformes | Sternopygidae | *Eigenmannia virescens* | -22,91139984 | -48,48789978 | LBPV-17142 | FUPR919-09 | GU701791 |
| Gymnotiformes | Sternopygidae | *Eigenmannia virescens* | -22,91139984 | -48,48789978 | LBPV-17144 | FUPR921-09 | GU701793 |
| Gymnotiformes | Sternopygidae | *Eigenmannia virescens* | -20,08539963 | -50,98310089 | LBPV-17145 | FUPR922-09 | GU701796 |
| Gymnotiformes | Sternopygidae | *Eigenmannia virescens* | -20,43350029 | -51,26139832 | LBPV-17147 | FUPR924-09 | GU701792 |
| Gymnotiformes | Sternopygidae | *Sternopygus macrurus* | -17,49099922 | -48,36999893 | LBPV-17270 | FUPR940-09 | JN989240 |
| Gymnotiformes | Sternopygidae | *Sternopygus macrurus* | -17,49099922 | -48,36999893 | LBPV-17271 | FUPR941-09 | JN989241 |
| Gymnotiformes | Sternopygidae | *Sternopygus macrurus* | -17,49099922 | -48,36999893 | LBPV-17272 | FUPR942-09 | GU701495 |
| Gymnotiformes | Sternopygidae | *Sternopygus macrurus* | -20,23699951 | -46,6629982 | LBPV-17273 | FUPR943-09 | GU701499 |
| Gymnotiformes | Sternopygidae | *Sternopygus macrurus* | -20,28700066 | -46,58200073 | LBPV-17274 | FUPR944-09 | GU701498 |
| Gymnotiformes | Sternopygidae | *Sternopygus macrurus* | -20,14599991 | -46,66999817 | LBPV-19229 | FUPR945-09 | GU701500 |
| Gymnotiformes | Sternopygidae | *Sternopygus macrurus* | -20,14599991 | -46,66999817 | LBPV-40269 | FUPR1004-09 | JN989242 |
| Gymnotiformes | Sternopygidae | *Sternopygus macrurus* | -20,14599991 | -46,66999817 | LBPV-40270 | FUPR1005-09 | GU701939 |
| Perciformes | Cichlidae | *Astronotus crassipinnis* | -21,0128994 | -49,69029999 | LBPV-26679 | FUPR969-09 | GU701855 |
| Perciformes | Cichlidae | *Astronotus crassipinnis* | -21,0128994 | -49,69029999 | LBPV-26680 | FUPR970-09 | GU701859 |
| Perciformes | Cichlidae | *Astronotus crassipinnis* | -21,0128994 | -49,69029999 | LBPV-26681 | FUPR971-09 | GU701858 |
| Perciformes | Cichlidae | *Astronotus crassipinnis* | -22,79140091 | -53,34939957 | LBPV-26682 | FUPR972-09 | GU701861 |
| Perciformes | Cichlidae | *Astronotus crassipinnis* | -22,79140091 | -53,34939957 | LBPV-26683 | FUPR973-09 | GU701860 |
| Perciformes | Cichlidae | *Astronotus crassipinnis* | -21,28510094 | -46,49349976 | LBPV-26684 | FUPR974-09 | GU701862 |
| Perciformes | Cichlidae | *Astronotus crassipinnis* | -22,65029907 | -53,09159851 | LBPV-45694 | FUPR1358-10 | JN988692 |
| Perciformes | Cichlidae | *Australoheros facetus* | -23,14999962 | -48,26670074 | LBPV-43486 | FUPR1290-10 | JN988750 |
| Perciformes | Cichlidae | *Cichla kelberi* | -22,72949982 | -53,31779861 | LBPV-26692 | FUPR1224-10 | JN988797 |
| Perciformes | Cichlidae | *Cichla kelberi* | -22,72949982 | -53,31779861 | LBPV-26693 | FUPR1225-10 | JN988796 |
| Perciformes | Cichlidae | *Cichla monoculus* | -22,72949982 | -53,31779861 | LBPV-26695 | FUPR1226-10 | JN988799 |
| Perciformes | Cichlidae | *Cichla monoculus* | -22,72949982 | -53,31779861 | LBPV-26696 | FUPR1227-10 | JN988798 |
| Perciformes | Cichlidae | *Cichla piquiti* | -21,4076004 | -50,05709839 | LBPV-20149 | FUPR1211-10 | JN988800 |
| Perciformes | Cichlidae | *Cichla sp.* | -20,62059975 | -49,89459991 | LBPV-32030 | FUPR1247-10 | JN988806 |
| Perciformes | Cichlidae | *Cichlasoma paranaense* | -20,62059975 | -49,89459991 | LBPV-25907 | FUPR1216-10 | JN988805 |
| Perciformes | Cichlidae | *Cichlasoma paranaense* | -22,43670082 | -49,2112999 | LBPV-29783 | FUPR1234-10 | JN988804 |
| Perciformes | Cichlidae | *Cichlasoma paranaense* | -22,43670082 | -49,2112999 | LBPV-31795 | FUPR1243-10 | JN988803 |
| Perciformes | Cichlidae | *Cichlasoma paranaense* | -22,43670082 | -49,2112999 | LBPV-31796 | FUPR1244-10 | JN988802 |
| Perciformes | Cichlidae | *Cichlasoma paranaense* | -21,19309998 | -49,12279892 | LBPV-32210 | FUPR1251-10 | JN988801 |
| Perciformes | Cichlidae | *Crenicichla britskii* | -21,92709923 | -47,36790085 | LBPV-26402 | FUPR1222-10 | JN988828 |
| Perciformes | Cichlidae | *Crenicichla britskii* | -21,92709923 | -47,36790085 | LBPV-26405 | FUPR1223-10 | JN988827 |
| Perciformes | Cichlidae | *Crenicichla britskii* | -21,92709923 | -47,36790085 | LBPV-31751 | FUPR1242-10 | JN988826 |
| Perciformes | Cichlidae | *Crenicichla britskii* | -21,92709923 | -47,36790085 | LBPV-31906 | FUPR1245-10 | JN988825 |
| Perciformes | Cichlidae | *Crenicichla britskii* | -22,63209915 | -48,17509842 | LBPV-31941 | FUPR1246-10 | JN988824 |
| Perciformes | Cichlidae | *Crenicichla britskii* | -22,63209915 | -48,17509842 | LBPV-37243 | FUPR1262-10 | JN988823 |
| Perciformes | Cichlidae | *Crenicichla britskii* | -23,52669907 | -45,81980133 | LBPV-40271 | FUPR1006-09 | GU701937 |
| Perciformes | Cichlidae | *Crenicichla britskii* | -21,92709923 | -47,36790085 | LBPV-40272 | FUPR1007-09 | GU701938 |
| Perciformes | Cichlidae | *Crenicichla britskii* | -21,92709923 | -47,36790085 | LBPV-40273 | FUPR1008-09 | GU701934 |
| Perciformes | Cichlidae | *Crenicichla britskii* | -21,92709923 | -47,36790085 | LBPV-40275 | FUPR1010-09 | GU701935 |
| Perciformes | Cichlidae | *Crenicichla cf. lepidota* | -22,63209915 | -48,17509842 | LBPV-37244 | FUPR1263-10 | JN988830 |
| Perciformes | Cichlidae | *Crenicichla haroldoi* | -23,14999962 | -48,26670074 | LBPV-31750 | FUPR1241-10 | JN988829 |
| Perciformes | Cichlidae | *Crenicichla niederleinii* | -23,14999962 | -48,26670074 | LBPV-40274 | FUPR1009-09 | JN988831 |
| Perciformes | Cichlidae | *Geophagus brasiliensis* | -22,79140091 | -53,34939957 | LBPV-28089 | FUPR1230-10 | JN988868 |
| Perciformes | Cichlidae | *Geophagus brasiliensis* | -22,79140091 | -53,34939957 | LBPV-28090 | FUPR1231-10 | JN988867 |
| Perciformes | Cichlidae | *Geophagus brasiliensis* | -22,79140091 | -53,34939957 | LBPV-34675 | FUPR1258-10 | JN988866 |
| Perciformes | Cichlidae | *Geophagus brasiliensis* | -22,36420059 | -47,5135994 | LBPV-34676 | FUPR1259-10 | JN988865 |
| Perciformes | Cichlidae | *Geophagus brasiliensis* | -22,67340088 | -53,08580017 | LBPV-40176 | FUPR1272-10 | JN988864 |
| Perciformes | Cichlidae | *Geophagus brasiliensis* | -23,77029991 | -46,31100082 | LBPV-40180 | FUPR1273-10 | JN988863 |
| Perciformes | Cichlidae | *Geophagus proximus* | -23,77029991 | -46,31100082 | LBPV-37219 | FUPR931-09 | JN988869 |
| Perciformes | Cichlidae | *Geophagus proximus* | -23,77029991 | -46,31100082 | LBPV-37220 | FUPR932-09 | GU701784 |
| Perciformes | Cichlidae | *Geophagus proximus* | -21,32699966 | -47,23880005 | LBPV-37221 | FUPR933-09 | GU701783 |
| Perciformes | Cichlidae | *Geophagus proximus* | -21,32699966 | -47,23880005 | LBPV-37222 | FUPR934-09 | GU701786 |
| Perciformes | Cichlidae | *Geophagus proximus* | -21,32699966 | -47,23880005 | LBPV-37223 | FUPR935-09 | GU701785 |
| Perciformes | Cichlidae | *Laetacara araguaiae* | -22,67589951 | -45,68289948 | LBPV-19752 | FUPR1204-10 | JN988977 |
| Perciformes | Cichlidae | *Laetacara araguaiae* | -22,67589951 | -45,68289948 | LBPV-19753 | FUPR1205-10 | JN988976 |
| Perciformes | Cichlidae | *Laetacara araguaiae* | -21,32909966 | -46,50130081 | LBPV-24778 | FUPR1213-10 | JN988975 |
| Perciformes | Cichlidae | *Laetacara araguaiae* | -22,67589951 | -45,68289948 | LBPV-24779 | FUPR1214-10 | JN988974 |
| Perciformes | Cichlidae | *Laetacara araguaiae* | -22,67589951 | -45,68289948 | LBPV-25861 | FUPR1215-10 | JN988973 |
| Perciformes | Cichlidae | *Laetacara araguaiae* | -22,67589951 | -45,68289948 | LBPV-32098 | FUPR1248-10 | JN988972 |
| Perciformes | Cichlidae | *Laetacara araguaiae* | -22,78560066 | -48,48149872 | LBPV-32099 | FUPR1249-10 | JN988971 |
| Perciformes | Cichlidae | *Laetacara araguaiae* | -23,51119995 | -45,85910034 | LBPV-32211 | FUPR1252-10 | JN988970 |
| Perciformes | Cichlidae | *Oreochromis niloticus* | -22,63260078 | -53,05260086 | LBPV-45740 | FUPR1361-10 | JN989116 |
| Perciformes | Cichlidae | *Satanoperca pappaterra* | -22,61400032 | -45,51399994 | LBPV-26750 | FUPR1229-10 | JN989214 |
| Perciformes | Cichlidae | *Satanoperca pappaterra* | -22,61400032 | -45,51399994 | LBPV-32101 | FUPR1250-10 | JN989213 |
| Perciformes | Cichlidae | *Tilapia rendalli* | -23,23060036 | -48,53279877 | LBPV-29857 | FUPR1235-10 | JN989250 |
| Perciformes | Cichlidae | *Tilapia rendalli* | -23,23060036 | -48,53279877 | LBPV-29858 | FUPR1236-10 | JN989249 |
| Perciformes | Cichlidae | *Tilapia rendalli* | -23,23060036 | -48,53279877 | LBPV-29972 | FUPR1239-10 | JN989248 |
| Perciformes | Cichlidae | *Tilapia rendalli* | -23,23060036 | -48,53279877 | LBPV-44541 | FUPR1297-10 | JN989247 |
| Perciformes | Cichlidae | *Tilapia rendalli* | -23,23060036 | -48,53279877 | LBPV-44542 | FUPR1298-10 | JN989246 |
| Perciformes | Cichlidae | *Tilapia rendalli* | -23,23060036 | -48,53279877 | LBPV-44774 | FUPR1327-10 | JN989245 |
| Perciformes | Cichlidae | *Tilapia rendalli* | -23,23060036 | -48,53279877 | LBPV-44775 | FUPR1328-10 | JN989244 |
| Perciformes | Cichlidae | *Tilapia rendalli* | -23,23060036 | -48,53279877 | LBPV-44776 | FUPR1329-10 | JN989243 |
| Perciformes | Sciaenidae | *Plagioscion squamosissimus* | -21,62409973 | -47,80630112 | LBPV-20157 | FUPR927-09 | JN989129 |
| Perciformes | Sciaenidae | *Plagioscion squamosissimus* | -21,62409973 | -47,80630112 | LBPV-20158 | FUPR928-09 | GU701891 |
| Perciformes | Sciaenidae | *Plagioscion squamosissimus* | -22,79140091 | -53,34939957 | LBPV-20159 | FUPR929-09 | GU701564 |
| Perciformes | Sciaenidae | *Plagioscion squamosissimus* | -21,62409973 | -47,80630112 | LBPV-26731 | FUPR925-09 | GU701562 |
| Perciformes | Sciaenidae | *Plagioscion squamosissimus* | -21,62409973 | -47,80630112 | LBPV-26732 | FUPR926-09 | JN989128 |
| Pleuronectiformes | Achiridae | *Catathyridium jenynsii* | -22,63969994 | -48,05310059 | LBPV-9849 | FUPR1193-10 | JN988777 |
| Pleuronectiformes | Achiridae | *Catathyridium jenynsii* | -22,63969994 | -48,05310059 | LBPV-9850 | FUPR1194-10 | JN988776 |
| Pleuronectiformes | Achiridae | *Catathyridium jenynsii* | -22,63969994 | -48,05310059 | LBPV-9854 | FUPR1195-10 | JN988775 |
| Pleuronectiformes | Achiridae | *Catathyridium jenynsii* | -22,63969994 | -48,05310059 | LBPV-9858 | FUPR1196-10 | JN988774 |
| Pleuronectiformes | Achiridae | *Catathyridium jenynsii* | -23,10040092 | -48,24010086 | LBPV-9859 | FUPR1197-10 | JN988773 |
| Pleuronectiformes | Achiridae | *Catathyridium jenynsii* | -23,10040092 | -48,24010086 | LBPV-9860 | FUPR1198-10 | JN988772 |
| Pleuronectiformes | Achiridae | *Catathyridium jenynsii* | -21,19309998 | -49,12279892 | LBPV-9861 | FUPR1199-10 | JN988771 |
| Pleuronectiformes | Achiridae | *Catathyridium jenynsii* | -21,19309998 | -49,12279892 | LBPV-9863 | FUPR1200-10 | JN988770 |
| Pleuronectiformes | Achiridae | *Catathyridium jenynsii* | -22,65029907 | -53,0788002 | LBPV-9866 | FUPR1201-10 | JN988769 |
| Rajiformes | Potamotrygonidae | *Potamotrygon falkneri* | -22,78560066 | -48,48149872 | LBPV-23266 | FUPR267-09 | JN989143 |
| Rajiformes | Potamotrygonidae | *Potamotrygon falkneri* | -22,65029907 | -53,09159851 | LBPV-23269 | FUPR268-09 | JN989144 |
| Rajiformes | Potamotrygonidae | *Potamotrygon falkneri* | -22,65029907 | -53,09159851 | LBPV-23279 | FUPR269-09 | JN989145 |
| Rajiformes | Potamotrygonidae | *Potamotrygon falkneri* | -22,63260078 | -53,05260086 | LBPV-23292 | FUPR270-09 | JN989146 |
| Rajiformes | Potamotrygonidae | *Potamotrygon motoro* | -22,64999962 | -53,09199905 | LBPV-36951 | FUPR253-09 | JN989150 |
| Rajiformes | Potamotrygonidae | *Potamotrygon motoro* | -22,63260078 | -53,05260086 | LBPV-36952 | FUPR254-09 | JN989147 |
| Rajiformes | Potamotrygonidae | *Potamotrygon motoro* | -22,64990044 | -53,09120178 | LBPV-36953 | FUPR255-09 | JN989148 |
| Rajiformes | Potamotrygonidae | *Potamotrygon motoro* | -22,64990044 | -53,09120178 | LBPV-36955 | FUPR256-09 | JN989149 |
| Rajiformes | Potamotrygonidae | *Potamotrygon motoro* | -22,6692009 | -53,08470154 | LBPV-36961 | FUPR257-09 | JN989151 |
| Rajiformes | Potamotrygonidae | *Potamotrygon motoro* | -22,6692009 | -53,08470154 | LBPV-36967 | FUPR258-09 | JN989152 |
| Rajiformes | Potamotrygonidae | *Potamotrygon motoro* | -22,6692009 | -53,08470154 | LBPV-36971 | FUPR259-09 | JN989153 |
| Rajiformes | Potamotrygonidae | *Potamotrygon motoro* | -23,51119995 | -45,85910034 | LBPV-36973 | FUPR263-09 | JN989157 |
| Rajiformes | Potamotrygonidae | *Potamotrygon motoro* | -23,51119995 | -45,85910034 | LBPV-36977 | FUPR264-09 | JN989158 |
| Rajiformes | Potamotrygonidae | *Potamotrygon motoro* | -22,67340088 | -53,08580017 | LBPV-36978 | FUPR260-09 | JN989154 |
| Rajiformes | Potamotrygonidae | *Potamotrygon motoro* | -23,51119995 | -45,85910034 | LBPV-36979 | FUPR265-09 | JN989159 |
| Rajiformes | Potamotrygonidae | *Potamotrygon motoro* | -22,67340088 | -53,08580017 | LBPV-36980 | FUPR261-09 | JN989155 |
| Rajiformes | Potamotrygonidae | *Potamotrygon motoro* | -23,51119995 | -45,85910034 | LBPV-36981 | FUPR262-09 | JN989156 |
| Rajiformes | Potamotrygonidae | *Potamotrygon motoro* | -23,51119995 | -45,85910034 | LBPV-36986 | FUPR266-09 | JN989160 |
| Siluriformes | Auchenipteridae | *Auchenipterus osteomystax* | -22,71759987 | -53,29100037 | LBPV-21932 | FUPR754-09 | GU701850 |
| Siluriformes | Auchenipteridae | *Auchenipterus osteomystax* | -22,71759987 | -53,29100037 | LBPV-21933 | FUPR755-09 | GU701854 |
| Siluriformes | Auchenipteridae | *Auchenipterus osteomystax* | -22,71759987 | -53,29100037 | LBPV-36606 | FUPR756-09 | GU701853 |
| Siluriformes | Auchenipteridae | *Auchenipterus osteomystax* | -21,10700035 | -50,26459885 | LBPV-36607 | FUPR757-09 | GU701857 |
| Siluriformes | Auchenipteridae | *Auchenipterus osteomystax* | -22,64999962 | -53,0909996 | LBPV-36618 | FUPR758-09 | GU701856 |
| Siluriformes | Auchenipteridae | *Tatia neivai* | -22,34090042 | -48,93470001 | LBPV-34797 | FUPR514-09 | HM376393 |
| Siluriformes | Auchenipteridae | *Trachelyopterus galeatus* | -23,23060036 | -48,53279877 | LBPV-17298 | FUPR509-09 | GU701485 |
| Siluriformes | Auchenipteridae | *Trachelyopterus galeatus* | -23,02389908 | -48,82569885 | LBPV-17299 | FUPR510-09 | GU701484 |
| Siluriformes | Auchenipteridae | *Trachelyopterus galeatus* | -22,94140053 | -48,58420181 | LBPV-17300 | FUPR511-09 | GU701488 |
| Siluriformes | Auchenipteridae | *Trachelyopterus galeatus* | -21,0128994 | -49,69029999 | LBPV-21934 | FUPR772-09 | GU701490 |
| Siluriformes | Auchenipteridae | *Trachelyopterus galeatus* | -21,0128994 | -49,69029999 | LBPV-21935 | FUPR773-09 | GU701489 |
| Siluriformes | Auchenipteridae | *Trachelyopterus galeatus* | -23,02389908 | -48,82569885 | LBPV-26663 | FUPR512-09 | GU701487 |
| Siluriformes | Auchenipteridae | *Trachelyopterus galeatus* | -21,0128994 | -49,69029999 | LBPV-26664 | FUPR513-09 | GU701486 |
| Siluriformes | Auchenipteridae | *Trachelyopterus galeatus* | -22,91670036 | -48,5 | LBPV-44982 | FUPR1347-10 | JN989254 |
| Siluriformes | Auchenipteridae | *Trachelyopterus galeatus* | -20,74550056 | -49,77930069 | LBPV-44983 | FUPR1348-10 | JN989253 |
| Siluriformes | Auchenipteridae | *Trachelyopterus galeatus* | -20,94709969 | -48,14780045 | LBPV-44984 | FUPR1349-10 | JN989252 |
| Siluriformes | Auchenipteridae | *Trachelyopterus galeatus* | -20,94709969 | -48,14780045 | LBPV-45956 | FUPR1364-10 | JN989251 |
| Siluriformes | Callichthyidae | *Aspidoras fuscoguttatus* | -22,63260078 | -53,05260086 | LBPV-10916 | FUPR271-09 | JN988689 |
| Siluriformes | Callichthyidae | *Aspidoras fuscoguttatus* | -22,64789963 | -53,07860184 | LBPV-17398 | FUPR272-09 | JN988690 |
| Siluriformes | Callichthyidae | *Aspidoras fuscoguttatus* | -21,0128994 | -49,69029999 | LBPV-17399 | FUPR273-09 | JN988691 |
| Siluriformes | Callichthyidae | *Callichthys callichthys* | -23,01280022 | -47,99769974 | LBPV-32362 | FUPR274-09 | JN988766 |
| Siluriformes | Callichthyidae | *Callichthys callichthys* | -23,01280022 | -47,99769974 | LBPV-32363 | FUPR275-09 | JN988767 |
| Siluriformes | Callichthyidae | *Callichthys callichthys* | -22,66049957 | -53,0848999 | LBPV-32364 | FUPR276-09 | JN988768 |
| Siluriformes | Callichthyidae | *Corydoras aeneus* | -22,7859993 | -45,6882019 | LBPV-24837 | FUPR277-09 | JN988809 |
| Siluriformes | Callichthyidae | *Corydoras aeneus* | -22,63209915 | -48,17509842 | LBPV-24838 | FUPR278-09 | JN988810 |
| Siluriformes | Callichthyidae | *Corydoras aeneus* | -22,63209915 | -48,17509842 | LBPV-31943 | FUPR279-09 | JN988811 |
| Siluriformes | Callichthyidae | *Corydoras aeneus* | -22,63209915 | -48,17509842 | LBPV-31944 | FUPR280-09 | JN988812 |
| Siluriformes | Callichthyidae | *Corydoras aeneus* | -22,36860085 | -47,47719955 | LBPV-31945 | FUPR281-09 | JN988813 |
| Siluriformes | Callichthyidae | *Corydoras difluviatilis* | -22,71759987 | -53,29100037 | LBPV-40007 | FUPR1011-09 | GU701936 |
| Siluriformes | Callichthyidae | *Corydoras difluviatilis* | -22,79140091 | -53,34939957 | LBPV-40008 | FUPR1012-09 | GU701932 |
| Siluriformes | Callichthyidae | *Corydoras difluviatilis* | -22,79140091 | -53,34939957 | LBPV-40009 | FUPR1013-09 | GU701933 |
| Siluriformes | Callichthyidae | *Corydoras difluviatilis* | -23,52669907 | -45,81980133 | LBPV-40010 | FUPR1014-09 | GU701930 |
| Siluriformes | Callichthyidae | *Corydoras difluviatilis* | -23,52669907 | -45,81980133 | LBPV-40011 | FUPR1015-09 | GU701931 |
| Siluriformes | Callichthyidae | *Corydoras difluviatilis* | -21,62409973 | -47,80630112 | LBPV-4624 | FUPR794-09 | JN988814 |
| Siluriformes | Callichthyidae | *Corydoras ehrhardti* | -22,94120026 | -48,58420181 | LBPV-32755 | FUPR515-09 | GU701814 |
| Siluriformes | Callichthyidae | *Corydoras ehrhardti* | -22,94120026 | -48,58420181 | LBPV-32756 | FUPR516-09 | GU701816 |
| Siluriformes | Callichthyidae | *Corydoras ehrhardti* | -22,94140053 | -48,58420181 | LBPV-32757 | FUPR517-09 | GU701815 |
| Siluriformes | Callichthyidae | *Corydoras ehrhardti* | -22,94140053 | -48,58420181 | LBPV-36124 | FUPR816-09 | GU701819 |
| Siluriformes | Callichthyidae | *Corydoras ehrhardti* | -21,7791996 | -48,5306015 | LBPV-36126 | FUPR817-09 | GU701818 |
| Siluriformes | Callichthyidae | *Corydoras ehrhardti* | -21,7791996 | -48,5306015 | LBPV-36128 | FUPR818-09 | GU701817 |
| Siluriformes | Callichthyidae | *Corydoras flaveolus* | -21,7791996 | -48,5306015 | LBPV-17225 | FUPR282-09 | JN988816 |
| Siluriformes | Callichthyidae | *Corydoras flaveolus* | -21,7791996 | -48,5306015 | LBPV-17226 | FUPR283-09 | JN988815 |
| Siluriformes | Callichthyidae | *Corydoras flaveolus* | -22,94140053 | -48,58420181 | LBPV-21755 | FUPR806-09 | JN988817 |
| Siluriformes | Callichthyidae | *Corydoras flaveolus* | -21,7791996 | -48,5306015 | LBPV-21756 | FUPR284-09 | GU701955 |
| Siluriformes | Callichthyidae | *Corydoras nattereri* | -19,68330002 | -48,66910172 | LBPV-32330 | FUPR285-09 | JN988818 |
| Siluriformes | Callichthyidae | *Corydoras nattereri* | -19,68330002 | -48,66910172 | LBPV-32331 | FUPR286-09 | JN988819 |
| Siluriformes | Callichthyidae | *Corydoras nattereri* | -19,68330002 | -48,66910172 | LBPV-32332 | FUPR287-09 | JN988820 |
| Siluriformes | Callichthyidae | *Corydoras nattereri* | -19,68330002 | -48,66910172 | LBPV-32333 | FUPR288-09 | JN988821 |
| Siluriformes | Callichthyidae | *Corydoras nattereri* | -22,79140091 | -53,34939957 | LBPV-32334 | FUPR289-09 | JN988822 |
| Siluriformes | Callichthyidae | *Corydoras paleatus* | -22,79140091 | -53,34939957 | LBPV-36114 | FUPR819-09 | GU701809 |
| Siluriformes | Callichthyidae | *Corydoras paleatus* | -23,64159966 | -51,85929871 | LBPV-36116 | FUPR820-09 | GU701871 |
| Siluriformes | Callichthyidae | *Corydoras paleatus* | -23,64159966 | -51,85929871 | LBPV-36117 | FUPR821-09 | GU701811 |
| Siluriformes | Callichthyidae | *Corydoras paleatus* | -23,64159966 | -51,85929871 | LBPV-36119 | FUPR822-09 | GU701810 |
| Siluriformes | Callichthyidae | *Corydoras paleatus* | -21,19309998 | -49,12279892 | LBPV-36121 | FUPR823-09 | GU701813 |
| Siluriformes | Callichthyidae | *Corydoras paleatus* | -21,19309998 | -49,12279892 | LBPV-36122 | FUPR824-09 | GU701812 |
| Siluriformes | Callichthyidae | *Hoplosternum littorale* | -20,23620033 | -48,67829895 | LBPV-15162 | FUPR291-09 | JN988910 |
| Siluriformes | Callichthyidae | *Hoplosternum littorale* | -20,23620033 | -48,67829895 | LBPV-15165 | FUPR292-09 | JN988911 |
| Siluriformes | Callichthyidae | *Hoplosternum littorale* | -20,40320015 | -48,63570023 | LBPV-15166 | FUPR293-09 | JN988912 |
| Siluriformes | Callichthyidae | *Hoplosternum littorale* | -20,23620033 | -48,67829895 | LBPV-25928 | FUPR294-09 | JN988913 |
| Siluriformes | Callichthyidae | *Lepthoplosternum pectorale* | -22,79140091 | -53,34939957 | LBPV-17678 | FUPR519-09 | GU701968 |
| Siluriformes | Callichthyidae | *Lepthoplosternum pectorale* | -22,79140091 | -53,34939957 | LBPV-17680 | FUPR520-09 | GU701967 |
| Siluriformes | Callichthyidae | *Lepthoplosternum pectorale* | -22,7220993 | -53,28649902 | LBPV-26094 | FUPR295-09 | JN989021 |
| Siluriformes | Callichthyidae | *Lepthoplosternum pectorale* | -22,71759987 | -53,29100037 | LBPV-26425 | FUPR518-09 | GU701639 |
| Siluriformes | Callichthyidae | *Megalechis thoracata* | - | - | LBPV-17673 | FUPR522-09 | GU701890 |
| Siluriformes | Callichthyidae | *Megalechis thoracata* | - | - | LBPV-17679 | FUPR523-09 | GU701965 |
| Siluriformes | Cetopsidae | *Cetopsis gobioides* | -22,65029907 | -53,0788002 | LBPV-33195 | FUPR524-09 | HM376394 |
| Siluriformes | Cetopsidae | *Cetopsis gobioides* | -22,79140091 | -53,34939957 | LBPV-38283 | FUPR1265-10 | JN988778 |
| Siluriformes | Clariidae | *Clarias gariepinus* | -22,63260078 | -53,05260086 | LBPV-31862 | FUPR532-09 | GU701826 |
| Siluriformes | Clariidae | *Clarias gariepinus* | -22,63260078 | -53,05260086 | LBPV-31863 | FUPR533-09 | GU701825 |
| Siluriformes | Clariidae | *Clarias gariepinus* | -22,63260078 | -53,05260086 | LBPV-31864 | FUPR534-09 | GU701829 |
| Siluriformes | Clariidae | *Clarias gariepinus* | -22,91010094 | -48,38899994 | LBPV-31865 | FUPR535-09 | GU701828 |
| Siluriformes | Clariidae | *Clarias gariepinus* | -21,19309998 | -49,12279892 | LBPV-31866 | FUPR536-09 | GU701827 |
| Siluriformes | Doradidae | *Pterodoras granulosus* | -15,74860001 | -47,82089996 | LBPV-19258 | FUPR528-09 | JN989181 |
| Siluriformes | Doradidae | *Pterodoras granulosus* | -15,73690033 | -47,9151001 | LBPV-19259 | FUPR526-09 | JN989179 |
| Siluriformes | Doradidae | *Pterodoras granulosus* | -15,71689987 | -47,86569977 | LBPV-19260 | FUPR529-09 | GU701898 |
| Siluriformes | Doradidae | *Pterodoras granulosus* | -15,74860001 | -47,82089996 | LBPV-19261 | FUPR527-09 | JN989180 |
| Siluriformes | Doradidae | *Pterodoras granulosus* | -15,73690033 | -47,9151001 | LBPV-26764 | FUPR525-09 | GU701552 |
| Siluriformes | Doradidae | *Rhinodoras dorbignyi* | -21,89669991 | -53,79029846 | LBPV-10334 | FUPR531-09 | JN989196 |
| Siluriformes | Doradidae | *Rhinodoras dorbignyi* | -22,04380035 | -53,72740173 | LBPV-17032 | FUPR530-09 | HM376395 |
| Siluriformes | Heptapteridae | *Cetopsorhamdia iheringi* | -21,10700035 | -50,26459885 | LBPV-11231 | FUPR774-09 | JN989188 |
| Siluriformes | Heptapteridae | *Cetopsorhamdia iheringi* | -22,67340088 | -53,08580017 | LBPV-21315 | FUPR467-09 | GU701840 |
| Siluriformes | Heptapteridae | *Cetopsorhamdia iheringi* | -22,67340088 | -53,08580017 | LBPV-21316 | FUPR468-09 | GU701842 |
| Siluriformes | Heptapteridae | *Cetopsorhamdia iheringi* | -22,63260078 | -53,05260086 | LBPV-37805 | FUPR464-09 | GU701838 |
| Siluriformes | Heptapteridae | *Cetopsorhamdia iheringi* | -22,66259956 | -53,10469818 | LBPV-37806 | FUPR465-09 | GU701841 |
| Siluriformes | Heptapteridae | *Cetopsorhamdia iheringi* | -22,67340088 | -53,08580017 | LBPV-37807 | FUPR466-09 | GU701876 |
| Siluriformes | Heptapteridae | *Chasmocranus brachynema* | -25,4197998 | -54,53559875 | LBPV-29946 | FUPR469-09 | GU701832 |
| Siluriformes | Heptapteridae | *Chasmocranus brachynema* | -25,4197998 | -54,53559875 | LBPV-29947 | FUPR470-09 | GU701831 |
| Siluriformes | Heptapteridae | *Chasmocranus brachynema* | -25,4197998 | -54,53559875 | LBPV-29948 | FUPR471-09 | GU701897 |
| Siluriformes | Heptapteridae | *Chasmocranus brachynema* | -25,4197998 | -54,53559875 | LBPV-29949 | FUPR472-09 | GU701830 |
| Siluriformes | Heptapteridae | *Chasmocranus sp.* | -25,4197998 | -54,53559875 | LBPV-44587 | FUPR1309-10 | JN988791 |
| Siluriformes | Heptapteridae | *Heptapterus multiradiatus* | -22,64789963 | -53,07860184 | LBPV-37224 | FUPR791-09 | GU701751 |
| Siluriformes | Heptapteridae | *Heptapterus multiradiatus* | -21,39209938 | -46,50310135 | LBPV-37225 | FUPR792-09 | GU701750 |
| Siluriformes | Heptapteridae | *Imparfinis mirini* | -25,4197998 | -54,53559875 | LBPV-21428 | FUPR1212-10 | JN988958 |
| Siluriformes | Heptapteridae | *Imparfinis mirini* | -25,4197998 | -54,53559875 | LBPV-22575 | FUPR478-09 | GU701658 |
| Siluriformes | Heptapteridae | *Imparfinis mirini* | -25,4197998 | -54,53559875 | LBPV-32341 | FUPR1253-10 | JN988957 |
| Siluriformes | Heptapteridae | *Imparfinis mirini* | -25,4197998 | -54,53559875 | LBPV-32342 | FUPR1254-10 | JN988956 |
| Siluriformes | Heptapteridae | *Imparfinis mirini* | -25,4197998 | -54,53559875 | LBPV-32343 | FUPR1255-10 | JN988955 |
| Siluriformes | Heptapteridae | *Imparfinis mirini* | -25,4197998 | -54,53559875 | LBPV-32344 | FUPR1256-10 | JN988954 |
| Siluriformes | Heptapteridae | *Imparfinis mirini* | -22,90369987 | -49,98339844 | LBPV-32345 | FUPR1257-10 | JN988953 |
| Siluriformes | Heptapteridae | *Imparfinis schubarti* | -25,4197998 | -54,53559875 | LBPV-26417 | FUPR479-09 | GU701649 |
| Siluriformes | Heptapteridae | *Imparfinis schubarti* | -23,02429962 | -48,8280983 | LBPV-34203 | FUPR775-09 | GU701538 |
| Siluriformes | Heptapteridae | *Imparfinis schubarti* | -23,02429962 | -48,8280983 | LBPV-34204 | FUPR776-09 | GU701542 |
| Siluriformes | Heptapteridae | *Imparfinis schubarti* | -22,79140091 | -53,34939957 | LBPV-35478 | FUPR473-09 | GU701653 |
| Siluriformes | Heptapteridae | *Imparfinis schubarti* | -22,79140091 | -53,34939957 | LBPV-35479 | FUPR474-09 | GU701652 |
| Siluriformes | Heptapteridae | *Imparfinis schubarti* | -22,57180023 | -53,04800034 | LBPV-35480 | FUPR475-09 | GU701656 |
| Siluriformes | Heptapteridae | *Imparfinis schubarti* | -22,57180023 | -53,04800034 | LBPV-35481 | FUPR476-09 | GU701655 |
| Siluriformes | Heptapteridae | *Imparfinis schubarti* | -22,57180023 | -53,04800034 | LBPV-35482 | FUPR477-09 | GU701654 |
| Siluriformes | Heptapteridae | *Phenacorhamdia tenebrosa* | -22,37800026 | -47,42699814 | LBPV-32265 | FUPR482-09 | GU701889 |
| Siluriformes | Heptapteridae | *Phenacorhamdia tenebrosa* | -22,37800026 | -47,42699814 | LBPV-32266 | FUPR483-09 | GU701887 |
| Siluriformes | Heptapteridae | *Phenacorhamdia tenebrosa* | -22,37800026 | -47,42699814 | LBPV-32267 | FUPR484-09 | GU701578 |
| Siluriformes | Heptapteridae | *Phenacorhamdia tenebrosa* | -22,63500023 | -52,8219986 | LBPV-35803 | FUPR480-09 | GU701577 |
| Siluriformes | Heptapteridae | *Phenacorhamdia tenebrosa* | -22,63470078 | -52,82210159 | LBPV-35804 | FUPR481-09 | GU701576 |
| Siluriformes | Heptapteridae | *Pimelodella avanhandavae* | -22,79140091 | -53,34939957 | LBPV-31967 | FUPR486-09 | GU701573 |
| Siluriformes | Heptapteridae | *Pimelodella avanhandavae* | -22,79140091 | -53,34939957 | LBPV-31968 | FUPR487-09 | GU701572 |
| Siluriformes | Heptapteridae | *Pimelodella avanhandavae* | -22,79140091 | -53,34939957 | LBPV-31969 | FUPR488-09 | GU701575 |
| Siluriformes | Heptapteridae | *Pimelodella avanhandavae* | -22,79140091 | -53,34939957 | LBPV-31970 | FUPR489-09 | GU701574 |
| Siluriformes | Heptapteridae | *Pimelodella meeki* | -22,63209915 | -48,16749954 | LBPV-9130 | FUPR1188-10 | JN989126 |
| Siluriformes | Heptapteridae | *Pimelodella meeki* | -22,63209915 | -48,16749954 | LBPV-9131 | FUPR1189-10 | JN989125 |
| Siluriformes | Heptapteridae | *Pimelodella meeki* | -22,7220993 | -53,28649902 | LBPV-9132 | FUPR1190-10 | JN989124 |
| Siluriformes | Heptapteridae | *Pimelodella meeki* | -22,7220993 | -53,28649902 | LBPV-9133 | FUPR1191-10 | JN989123 |
| Siluriformes | Heptapteridae | *Pimelodella meeki* | -22,63209915 | -48,17509842 | LBPV-9134 | FUPR1192-10 | JN989122 |
| Siluriformes | Heptapteridae | *Pimelodella sp1* | -22,63209915 | -48,17509842 | LBPV-37213 | FUPR485-09 | HM376392 |
| Siluriformes | Heptapteridae | *Rhamdia quelen* | -15,66730022 | -47,95240021 | LBPV-35805 | FUPR777-09 | GU701541 |
| Siluriformes | Heptapteridae | *Rhamdia quelen* | -15,7269001 | -47,91889954 | LBPV-35806 | FUPR778-09 | GU701544 |
| Siluriformes | Heptapteridae | *Rhamdia quelen* | -15,7269001 | -47,91889954 | LBPV-35807 | FUPR779-09 | GU701543 |
| Siluriformes | Heptapteridae | *Rhamdiopsis microcephala* | -15,7269001 | -47,91889954 | LBPV-29183 | FUPR490-09 | JN989189 |
| Siluriformes | Heptapteridae | *Rhamdiopsis microcephala* | -15,7269001 | -47,91889954 | LBPV-29184 | FUPR491-09 | GU701537 |
| Siluriformes | Heptapteridae | *Rhamdiopsis microcephala* | -15,7269001 | -47,91889954 | LBPV-29185 | FUPR492-09 | GU701536 |
| Siluriformes | Heptapteridae | *Rhamdiopsis microcephala* | -15,7269001 | -47,91889954 | LBPV-29186 | FUPR493-09 | GU701540 |
| Siluriformes | Heptapteridae | *Rhamdiopsis microcephala* | -15,73550034 | -47,91859818 | LBPV-29187 | FUPR494-09 | GU701539 |
| Siluriformes | Loricariidae | *Ancistrus cirrhosus* | -23,57640076 | -45,97449875 | LBPV-34759 | FUPR787-09 | JN988666 |
| Siluriformes | Loricariidae | *Ancistrus cirrhosus* | -23,65579987 | -46,83110046 | LBPV-35959 | FUPR780-09 | GU701863 |
| Siluriformes | Loricariidae | *Ancistrus cirrhosus* | -23,57640076 | -45,97449875 | LBPV-35987 | FUPR782-09 | GU701865 |
| Siluriformes | Loricariidae | *Corumbataia britskii* | -22,91010094 | -48,38899994 | LBPV-44923 | FUPR1340-10 | JN988808 |
| Siluriformes | Loricariidae | *Corumbataia britskii* | -22,76339912 | -48,26160049 | LBPV-44934 | FUPR1341-10 | JN988807 |
| Siluriformes | Loricariidae | *Corumbataia cuestae* | -23,51119995 | -45,85910034 | LBPV-18714 | FUPR495-09 | GU701820 |
| Siluriformes | Loricariidae | *Corumbataia cuestae* | -23,51119995 | -45,85910034 | LBPV-18715 | FUPR496-09 | GU701822 |
| Siluriformes | Loricariidae | *Corumbataia cuestae* | -23,51119995 | -45,85910034 | LBPV-18716 | FUPR497-09 | GU701821 |
| Siluriformes | Loricariidae | *Corumbataia cuestae* | -23,51119995 | -45,85910034 | LBPV-18717 | FUPR498-09 | GU701824 |
| Siluriformes | Loricariidae | *Corumbataia cuestae* | -23,51119995 | -45,85910034 | LBPV-18718 | FUPR499-09 | GU701823 |
| Siluriformes | Loricariidae | *Farlowella amazonum* | -20,08539963 | -50,98310089 | LBPV-26397 | FUPR500-09 | GU701788 |
| Siluriformes | Loricariidae | *Farlowella amazonum* | -22,65029907 | -53,09159851 | LBPV-26398 | FUPR501-09 | GU701787 |
| Siluriformes | Loricariidae | *Farlowella amazonum* | -22,93919945 | -50,25230026 | LBPV-26399 | FUPR502-09 | GU701790 |
| Siluriformes | Loricariidae | *Farlowella amazonum* | -22,93919945 | -50,25230026 | LBPV-26400 | FUPR503-09 | GU701789 |
| Siluriformes | Loricariidae | *Farlowella amazonum* | -22,93919945 | -50,25230026 | LBPV-26401 | FUPR504-09 | GU701894 |
| Siluriformes | Loricariidae | *Farlowella amazonum* | -20,34230042 | -46,7867012 | LBPV-44975 | FUPR1345-10 | JN988860 |
| Siluriformes | Loricariidae | *Farlowella amazonum* | -20,34230042 | -46,7867012 | LBPV-44976 | FUPR1346-10 | JN988859 |
| Siluriformes | Loricariidae | *Harttia gracilis* | -24,07349968 | -52,29109955 | LBPV-29820 | FUPR290-09 | JN988882 |
| Siluriformes | Loricariidae | *Harttia gracilis* | -24,20059967 | -48,41839981 | LBPV-29821 | FUPR506-09 | GU701899 |
| Siluriformes | Loricariidae | *Harttia gracilis* | -24,20059967 | -48,41839981 | LBPV-29822 | FUPR507-09 | GU701757 |
| Siluriformes | Loricariidae | *Harttia gracilis* | -24,20059967 | -48,41839981 | LBPV-29823 | FUPR508-09 | GU701756 |
| Siluriformes | Loricariidae | *Harttia gracilis* | -24,20059967 | -48,41839981 | LBPV-29838 | FUPR537-09 | GU701755 |
| Siluriformes | Loricariidae | *Hisonotus aff. francirochai* | -21,39209938 | -46,50310135 | LBPV-34980 | FUPR550-09 | JN988895 |
| Siluriformes | Loricariidae | *Hisonotus aff. francirochai* | -21,39209938 | -46,50310135 | LBPV-34981 | FUPR551-09 | JN988896 |
| Siluriformes | Loricariidae | *Hisonotus cf. paulinus* | -21,39209938 | -46,50310135 | LBPV-21478 | FUPR809-09 | JN313380 |
| Siluriformes | Loricariidae | *Hisonotus insperatus* | -23,14999962 | -48,26670074 | LBPV-13770 | FUPR545-09 | GU701748 |
| Siluriformes | Loricariidae | *Hisonotus insperatus* | -21,39209938 | -46,50310135 | LBPV-32260 | FUPR538-09 | GU701747 |
| Siluriformes | Loricariidae | *Hisonotus insperatus* | -23,14999962 | -48,26670074 | LBPV-32261 | FUPR539-09 | GU701888 |
| Siluriformes | Loricariidae | *Hisonotus insperatus* | -23,14999962 | -48,26670074 | LBPV-32262 | FUPR540-09 | GU701746 |
| Siluriformes | Loricariidae | *Hisonotus insperatus* | -23,14999962 | -48,26670074 | LBPV-32263 | FUPR541-09 | GU701438 |
| Siluriformes | Loricariidae | *Hisonotus insperatus* | -23,14999962 | -48,26670074 | LBPV-32264 | FUPR542-09 | GU701749 |
| Siluriformes | Loricariidae | *Hisonotus nigricauda* | -22,79140091 | -53,34939957 | LBPV-38162 | FUPR826-09 | JN988897 |
| Siluriformes | Loricariidae | *Hisonotus nigricauda* | -22,79140091 | -53,34939957 | LBPV-38164 | FUPR828-09 | GU701743 |
| Siluriformes | Loricariidae | *Hisonotus nigricauda* | -22,79140091 | -53,34939957 | LBPV-38165 | FUPR829-09 | GU701742 |
| Siluriformes | Loricariidae | *Hypostomus albopunctatus* | -17,09939957 | -48,76150131 | LBPV-29927 | FUPR552-09 | GU701728 |
| Siluriformes | Loricariidae | *Hypostomus albopunctatus* | -17,09939957 | -48,76150131 | LBPV-29928 | FUPR553-09 | GU701731 |
| Siluriformes | Loricariidae | *Hypostomus albopunctatus* | -17,09939957 | -48,76150131 | LBPV-29929 | FUPR554-09 | GU701730 |
| Siluriformes | Loricariidae | *Hypostomus ancistroides* | -22,36420059 | -47,5135994 | LBPV-25962 | FUPR573-09 | JN988948 |
| Siluriformes | Loricariidae | *Hypostomus ancistroides* | -23,30310059 | -52,03170013 | LBPV-25964 | FUPR615-09 | GU701726 |
| Siluriformes | Loricariidae | *Hypostomus ancistroides* | -21,36129951 | -46,55630112 | LBPV-25965 | FUPR616-09 | GU701725 |
| Siluriformes | Loricariidae | *Hypostomus ancistroides* | -21,36129951 | -46,55630112 | LBPV-25966 | FUPR617-09 | GU701729 |
| Siluriformes | Loricariidae | *Hypostomus ancistroides* | -21,32699966 | -47,23880005 | LBPV-32288 | FUPR613-09 | GU701723 |
| Siluriformes | Loricariidae | *Hypostomus ancistroides* | -23,30310059 | -52,03170013 | LBPV-32289 | FUPR614-09 | GU701727 |
| Siluriformes | Loricariidae | *Hypostomus brevis* | -21,29360008 | -46,48529816 | LBPV-16365 | FUPR557-09 | JN988933 |
| Siluriformes | Loricariidae | *Hypostomus brevis* | -21,29360008 | -46,48529816 | LBPV-16367 | FUPR558-09 | JN988934 |
| Siluriformes | Loricariidae | *Hypostomus brevis* | -21,29360008 | -46,48529816 | LBPV-16368 | FUPR559-09 | GU701724 |
| Siluriformes | Loricariidae | *Hypostomus brevis* | -17,09939957 | -48,76150131 | LBPV-16369 | FUPR560-09 | JN988935 |
| Siluriformes | Loricariidae | *Hypostomus brevis* | -21,06669998 | -50,11500168 | LBPV-17561 | FUPR878-09 | GU701721 |
| Siluriformes | Loricariidae | *Hypostomus brevis* | -17,09939957 | -48,76150131 | LBPV-17565 | FUPR866-09 | GU701712 |
| Siluriformes | Loricariidae | *Hypostomus brevis* | -17,09939957 | -48,76150131 | LBPV-17566 | FUPR867-09 | GU701715 |
| Siluriformes | Loricariidae | *Hypostomus cochliodon* | -21,06669998 | -50,11500168 | LBPV-17564 | FUPR861-09 | HM376401 |
| Siluriformes | Loricariidae | *Hypostomus commersoni* | -21,92709923 | -47,36790085 | LBPV-16635 | FUPR875-09 | JN988938 |
| Siluriformes | Loricariidae | *Hypostomus commersoni* | -21,92709923 | -47,36790085 | LBPV-16636 | FUPR876-09 | GU701719 |
| Siluriformes | Loricariidae | *Hypostomus commersoni* | -21,92709923 | -47,36790085 | LBPV-16640 | FUPR877-09 | GU701722 |
| Siluriformes | Loricariidae | *Hypostomus commersoni* | -22,93919945 | -50,25230026 | LBPV-16641 | FUPR870-09 | GU701717 |
| Siluriformes | Loricariidae | *Hypostomus commersoni* | -22,93919945 | -50,25230026 | LBPV-16668 | FUPR872-09 | GU701716 |
| Siluriformes | Loricariidae | *Hypostomus commersoni* | -21,92709923 | -47,36790085 | LBPV-16669 | FUPR873-09 | JN988937 |
| Siluriformes | Loricariidae | *Hypostomus commersoni* | -22,93919945 | -50,25230026 | LBPV-16670 | FUPR871-09 | JN988936 |
| Siluriformes | Loricariidae | *Hypostomus commersoni* | -21,92709923 | -47,36790085 | LBPV-16671 | FUPR874-09 | GU701720 |
| Siluriformes | Loricariidae | *Hypostomus derbyi* | -21,92709923 | -47,36790085 | LBPV-16643 | FUPR865-09 | JN988940 |
| Siluriformes | Loricariidae | *Hypostomus derbyi* | -21,92709923 | -47,36790085 | LBPV-16644 | FUPR864-09 | GU701713 |
| Siluriformes | Loricariidae | *Hypostomus heraldoi* | -22,94120026 | -48,58440018 | LBPV-20912 | FUPR590-09 | GU701705 |
| Siluriformes | Loricariidae | *Hypostomus heraldoi* | -22,78429985 | -48,48160172 | LBPV-20867 | FUPR595-09 | GU701710 |
| Siluriformes | Loricariidae | *Hypostomus heraldoi* | -22,94120026 | -48,58440018 | LBPV-20868 | FUPR555-09 | GU701733 |
| Siluriformes | Loricariidae | *Hypostomus heraldoi* | -23,64170074 | -51,85900116 | LBPV-20909 | FUPR591-09 | GU701704 |
| Siluriformes | Loricariidae | *Hypostomus heraldoi* | -22,93560028 | -48,32080078 | LBPV-20914 | FUPR594-09 | GU701711 |
| Siluriformes | Loricariidae | *Hypostomus heraldoi* | -22,93560028 | -48,32080078 | LBPV-20915 | FUPR592-09 | GU701708 |
| Siluriformes | Loricariidae | *Hypostomus heraldoi* | -22,36860085 | -47,47719955 | LBPV-22594 | FUPR596-09 | GU701709 |
| Siluriformes | Loricariidae | *Hypostomus hermanni* | -22,93919945 | -50,25230026 | LBPV-11271 | FUPR561-09 | GU701703 |
| Siluriformes | Loricariidae | *Hypostomus hermanni* | -22,93919945 | -50,25230026 | LBPV-11274 | FUPR562-09 | GU701702 |
| Siluriformes | Loricariidae | *Hypostomus hermanni* | -21,57480049 | -46,32360077 | LBPV-11275 | FUPR563-09 | GU701706 |
| Siluriformes | Loricariidae | *Hypostomus hermanni* | -21,57480049 | -46,32360077 | LBPV-20922 | FUPR593-09 | GU701707 |
| Siluriformes | Loricariidae | *Hypostomus iheringii* | -21,57480049 | -46,32360077 | LBPV-17039 | FUPR569-09 | GU701699 |
| Siluriformes | Loricariidae | *Hypostomus microstomus* | -21,57480049 | -46,32360077 | LBPV-16651 | FUPR849-09 | GU701694 |
| Siluriformes | Loricariidae | *Hypostomus microstomus* | -21,57480049 | -46,32360077 | LBPV-16652 | FUPR850-09 | GU701698 |
| Siluriformes | Loricariidae | *Hypostomus myersi* | -21,43309975 | -50,06129837 | LBPV-16637 | FUPR852-09 | GU701693 |
| Siluriformes | Loricariidae | *Hypostomus myersi* | -21,43309975 | -50,06129837 | LBPV-16638 | FUPR853-09 | GU701692 |
| Siluriformes | Loricariidae | *Hypostomus myersi* | -21,17679977 | -49,17250061 | LBPV-16639 | FUPR854-09 | GU701696 |
| Siluriformes | Loricariidae | *Hypostomus nigromaculatus* | -21,17679977 | -49,17250061 | LBPV-35845 | FUPR570-09 | GU701686 |
| Siluriformes | Loricariidae | *Hypostomus nigromaculatus* | -22,36420059 | -47,5135994 | LBPV-35846 | FUPR571-09 | GU701689 |
| Siluriformes | Loricariidae | *Hypostomus nigromaculatus* | -22,36420059 | -47,5135994 | LBPV-35847 | FUPR572-09 | GU701688 |
| Siluriformes | Loricariidae | *Hypostomus paulinus* | -24,07349968 | -52,29109955 | LBPV-27984 | FUPR579-09 | GU701684 |
| Siluriformes | Loricariidae | *Hypostomus paulinus* | -24,07349968 | -52,29109955 | LBPV-27985 | FUPR580-09 | GU701683 |
| Siluriformes | Loricariidae | *Hypostomus paulinus* | -23,68190002 | -51,31520081 | LBPV-27986 | FUPR581-09 | GU701687 |
| Siluriformes | Loricariidae | *Hypostomus paulinus* | -23,68190002 | -51,31520081 | LBPV-28384 | FUPR606-09 | GU701662 |
| Siluriformes | Loricariidae | *Hypostomus paulinus* | -23,68190002 | -51,31520081 | LBPV-28425 | FUPR607-09 | GU701661 |
| Siluriformes | Loricariidae | *Hypostomus paulinus* | -17,1196003 | -48,73989868 | LBPV-28426 | FUPR608-09 | GU701665 |
| Siluriformes | Loricariidae | *Hypostomus paulinus* | -17,1196003 | -48,73989868 | LBPV-28427 | FUPR609-09 | GU701664 |
| Siluriformes | Loricariidae | *Hypostomus paulinus* | -17,25600052 | -48,50559998 | LBPV-28428 | FUPR610-09 | GU701663 |
| Siluriformes | Loricariidae | *Hypostomus paulinus* | -22,36420059 | -47,5135994 | LBPV-29212 | FUPR577-09 | GU701681 |
| Siluriformes | Loricariidae | *Hypostomus paulinus* | -24,07349968 | -52,29109955 | LBPV-29213 | FUPR578-09 | GU701685 |
| Siluriformes | Loricariidae | *Hypostomus regani* | -24,20059967 | -48,41839981 | LBPV-11266 | FUPR587-09 | GU701678 |
| Siluriformes | Loricariidae | *Hypostomus regani* | -24,20059967 | -48,41839981 | LBPV-11267 | FUPR588-09 | GU701682 |
| Siluriformes | Loricariidae | *Hypostomus regani* | -24,20059967 | -48,41839981 | LBPV-11268 | FUPR589-09 | GU701963 |
| Siluriformes | Loricariidae | *Hypostomus regani* | -24,20059967 | -48,41839981 | LBPV-17040 | FUPR585-09 | JN988949 |
| Siluriformes | Loricariidae | *Hypostomus regani* | -24,20059967 | -48,41839981 | LBPV-17041 | FUPR586-09 | JN988950 |
| Siluriformes | Loricariidae | *Hypostomus regani* | -21,19309998 | -49,12279892 | LBPV-17541 | FUPR851-09 | GU701690 |
| Siluriformes | Loricariidae | *Hypostomus regani* | -21,19309998 | -49,12279892 | LBPV-17559 | FUPR855-09 | GU701695 |
| Siluriformes | Loricariidae | *Hypostomus regani* | -19,6154995 | -47,44979858 | LBPV-24686 | FUPR575-09 | GU701966 |
| Siluriformes | Loricariidae | *Hypostomus regani* | -19,6154995 | -47,44979858 | LBPV-24689 | FUPR576-09 | GU701964 |
| Siluriformes | Loricariidae | *Hypostomus regani* | -17,25600052 | -48,50559998 | LBPV-32028 | FUPR556-09 | GU701732 |
| Siluriformes | Loricariidae | *Hypostomus regani* | -19,6154995 | -47,44979858 | LBPV-35854 | FUPR582-09 | GU701676 |
| Siluriformes | Loricariidae | *Hypostomus regani* | -19,6154995 | -47,44979858 | LBPV-35855 | FUPR583-09 | GU701680 |
| Siluriformes | Loricariidae | *Hypostomus regani* | -19,6154995 | -47,44979858 | LBPV-35856 | FUPR584-09 | GU701679 |
| Siluriformes | Loricariidae | *Hypostomus sp.* | -23,25 | -46,96670151 | LBPV-16653 | FUPR859-09 | JN988947 |
| Siluriformes | Loricariidae | *Hypostomus sp.* | -23,25 | -46,96670151 | LBPV-17549 | FUPR856-09 | GU701697 |
| Siluriformes | Loricariidae | *Hypostomus sp1* | -23,25 | -46,96670151 | LBPV-18622 | FUPR574-09 | GU701691 |
| Siluriformes | Loricariidae | *Hypostomus strigaticeps* | -22,79140091 | -53,34939957 | LBPV-10567 | FUPR602-09 | GU701877 |
| Siluriformes | Loricariidae | *Hypostomus strigaticeps* | -22,65029907 | -53,09159851 | LBPV-10982 | FUPR599-09 | GU701668 |
| Siluriformes | Loricariidae | *Hypostomus strigaticeps* | -23,25 | -46,96670151 | LBPV-11257 | FUPR564-09 | JN988941 |
| Siluriformes | Loricariidae | *Hypostomus strigaticeps* | -23,23060036 | -48,53279877 | LBPV-11258 | FUPR604-09 | GU701674 |
| Siluriformes | Loricariidae | *Hypostomus strigaticeps* | -25,4197998 | -54,53559875 | LBPV-11258b | FUPR976-09 | GU701954 |
| Siluriformes | Loricariidae | *Hypostomus strigaticeps* | -20,23620033 | -48,67829895 | LBPV-11259 | FUPR605-09 | GU701951 |
| Siluriformes | Loricariidae | *Hypostomus strigaticeps* | -25,4197998 | -54,53559875 | LBPV-11259b | FUPR977-09 | GU701673 |
| Siluriformes | Loricariidae | *Hypostomus strigaticeps* | -23,64159966 | -51,85929871 | LBPV-16648 | FUPR857-09 | JN988945 |
| Siluriformes | Loricariidae | *Hypostomus strigaticeps* | -25,4197998 | -54,53559875 | LBPV-16649 | FUPR879-09 | GU701667 |
| Siluriformes | Loricariidae | *Hypostomus strigaticeps* | -25,4197998 | -54,53559875 | LBPV-16650 | FUPR880-09 | GU701666 |
| Siluriformes | Loricariidae | *Hypostomus strigaticeps* | -23,64159966 | -51,85929871 | LBPV-16654 | FUPR858-09 | JN988946 |
| Siluriformes | Loricariidae | *Hypostomus strigaticeps* | -20,23620033 | -48,67829895 | LBPV-16666 | FUPR831-09 | GU701953 |
| Siluriformes | Loricariidae | *Hypostomus strigaticeps* | -23,64159966 | -51,85929871 | LBPV-16667 | FUPR832-09 | JN988951 |
| Siluriformes | Loricariidae | *Hypostomus strigaticeps* | -23,64159966 | -51,85929871 | LBPV-17528 | FUPR860-09 | GU701701 |
| Siluriformes | Loricariidae | *Hypostomus strigaticeps* | -25,4197998 | -54,53559875 | LBPV-17562 | FUPR869-09 | GU701718 |
| Siluriformes | Loricariidae | *Hypostomus strigaticeps* | -23,68190002 | -51,31520081 | LBPV-17563 | FUPR868-09 | GU701714 |
| Siluriformes | Loricariidae | *Hypostomus strigaticeps* | -22,65029907 | -53,09159851 | LBPV-23445 | FUPR600-09 | GU701672 |
| Siluriformes | Loricariidae | *Hypostomus strigaticeps* | -22,79140091 | -53,34939957 | LBPV-23446 | FUPR601-09 | GU701671 |
| Siluriformes | Loricariidae | *Hypostomus strigaticeps* | -23,23060036 | -48,53279877 | LBPV-25930 | FUPR603-09 | GU701675 |
| Siluriformes | Loricariidae | *Hypostomus strigaticeps* | -23,25 | -46,96670151 | LBPV-31517 | FUPR597-09 | GU701670 |
| Siluriformes | Loricariidae | *Hypostomus strigaticeps* | -22,65029907 | -53,09159851 | LBPV-31518 | FUPR598-09 | GU701669 |
| Siluriformes | Loricariidae | *Hypostomus topavae* | -25,4197998 | -54,53559875 | LBPV-17214 | FUPR565-09 | JN988942 |
| Siluriformes | Loricariidae | *Hypostomus topavae* | -25,4197998 | -54,53559875 | LBPV-17215 | FUPR566-09 | JN988943 |
| Siluriformes | Loricariidae | *Hypostomus topavae* | -21,29039955 | -46,49509811 | LBPV-17216 | FUPR567-09 | JN988944 |
| Siluriformes | Loricariidae | *Hypostomus topavae* | -21,28499985 | -46,49330139 | LBPV-17217 | FUPR568-09 | GU701700 |
| Siluriformes | Loricariidae | *Hypostomus topavae* | -22,65029907 | -53,09159851 | LBPV-18246 | FUPR611-09 | GU701659 |
| Siluriformes | Loricariidae | *Hypostomus topavae* | -25,4197998 | -54,53559875 | LBPV-18247 | FUPR612-09 | JN988952 |
| Siluriformes | Loricariidae | *Isbrueckerichthys saxicola* | -23,67469978 | -52,1189003 | LBPV-29763 | FUPR621-09 | GU701647 |
| Siluriformes | Loricariidae | *Isbrueckerichthys saxicola* | -23,67469978 | -52,1189003 | LBPV-29764 | FUPR622-09 | GU701651 |
| Siluriformes | Loricariidae | *Isbrueckerichthys saxicola* | -23,67469978 | -52,1189003 | LBPV-29767 | FUPR623-09 | GU701650 |
| Siluriformes | Loricariidae | *Isbrueckerichthys saxicola* | -22,74720001 | -48,47489929 | LBPV-29994 | FUPR618-09 | GU701648 |
| Siluriformes | Loricariidae | *Isbrueckerichthys saxicola* | -22,74720001 | -48,47489929 | LBPV-29995 | FUPR619-09 | GU701961 |
| Siluriformes | Loricariidae | *Isbrueckerichthys saxicola* | -22,74720001 | -48,47489929 | LBPV-29996 | FUPR620-09 | GU701962 |
| Siluriformes | Loricariidae | *Isbrueckerichthys saxicola* | -22,90399933 | -49,98300171 | LBPV-40259 | FUPR994-09 | GU701905 |
| Siluriformes | Loricariidae | *Isbrueckerichthys saxicola* | -17,73889923 | -48,4742012 | LBPV-40260 | FUPR995-09 | GU701906 |
| Siluriformes | Loricariidae | *Isbrueckerichthys saxicola* | -17,73889923 | -48,4742012 | LBPV-40261 | FUPR996-09 | GU701902 |
| Siluriformes | Loricariidae | *Isbrueckerichthys saxicola* | -17,73889923 | -48,4742012 | LBPV-40262 | FUPR997-09 | GU701903 |
| Siluriformes | Loricariidae | *Isbrueckerichthys saxicola* | -17,73889923 | -48,4742012 | LBPV-40263 | FUPR998-09 | GU701901 |
| Siluriformes | Loricariidae | *Loricaria lentiginosa* | -22,65029907 | -53,09159851 | LBPV-37206 | FUPR759-09 | GU701637 |
| Siluriformes | Loricariidae | *Loricaria lentiginosa* | -22,79140091 | -53,34939957 | LBPV-37207 | FUPR760-09 | GU701636 |
| Siluriformes | Loricariidae | *Loricariichthys platymetopon* | -23,93829918 | -50,72900009 | LBPV-17294 | FUPR803-09 | GU701634 |
| Siluriformes | Loricariidae | *Loricariichthys platymetopon* | -22,79140091 | -53,34939957 | LBPV-17295 | FUPR296-09 | JN989022 |
| Siluriformes | Loricariidae | *Loricariichthys platymetopon* | -23,93829918 | -50,72900009 | LBPV-19262 | FUPR801-09 | GU701635 |
| Siluriformes | Loricariidae | *Loricariichthys platymetopon* | -23,93829918 | -50,72900009 | LBPV-26392 | FUPR804-09 | JN989024 |
| Siluriformes | Loricariidae | *Loricariichthys platymetopon* | - | - | LBPV-26393 | FUPR805-09 | GU701638 |
| Siluriformes | Loricariidae | *Loricariichthys platymetopon* | -22,79140091 | -53,34939957 | LBPV-26395 | FUPR297-09 | JN989023 |
| Siluriformes | Loricariidae | *Megalancistrus parananus* | - | - | LBPV-36608 | FUPR767-09 | GU701632 |
| Siluriformes | Loricariidae | *Megalancistrus parananus* | - | - | LBPV-36609 | FUPR768-09 | GU701878 |
| Siluriformes | Loricariidae | *Megalancistrus parananus* | - | - | LBPV-36943 | FUPR769-09 | GU701874 |
| Siluriformes | Loricariidae | *Megalancistrus parananus* | - | - | LBPV-36944 | FUPR770-09 | GU701875 |
| Siluriformes | Loricariidae | *Microlepidogaster sp.* | - | - | LBPV-44512 | FUPR1295-10 | JN989037 |
| Siluriformes | Loricariidae | *Microlepidogaster sp.* | - | - | LBPV-44556 | FUPR1299-10 | JN989036 |
| Siluriformes | Loricariidae | *Microlepidogaster sp.* | - | - | LBPV-44557 | FUPR1300-10 | JN989035 |
| Siluriformes | Loricariidae | *Microlepidogaster sp.* | - | - | LBPV-44558 | FUPR1301-10 | JN989034 |
| Siluriformes | Loricariidae | *Neoplecostomus bandeirante* | - | - | LBPV-18612 | FUPR1399-10 | JN989097 |
| Siluriformes | Loricariidae | *Neoplecostomus bandeirante* | - | - | LBPV-18613 | FUPR1400-10 | JN989096 |
| Siluriformes | Loricariidae | *Neoplecostomus bandeirante* | - | - | LBPV-18614 | FUPR1401-10 | JN989095 |
| Siluriformes | Loricariidae | *Neoplecostomus bandeirante* | - | - | LBPV-18615 | FUPR1402-10 | JN989094 |
| Siluriformes | Loricariidae | *Neoplecostomus bandeirante* | - | - | LBPV-18616 | FUPR1403-10 | JN989093 |
| Siluriformes | Loricariidae | *Neoplecostomus botucatu* | -24,20509911 | -52,7983017 | LBPV-34832 | FUPR1425-10 | JN989075 |
| Siluriformes | Loricariidae | *Neoplecostomus botucatu* | -22,8689003 | -48,3669014 | LBPV-34833 | FUPR1426-10 | JN989074 |
| Siluriformes | Loricariidae | *Neoplecostomus botucatu* | -22,78560066 | -48,48149872 | LBPV-34834 | FUPR1427-10 | JN989073 |
| Siluriformes | Loricariidae | *Neoplecostomus botucatu* | -22,65029907 | -53,0788002 | LBPV-34835 | FUPR1428-10 | JN989072 |
| Siluriformes | Loricariidae | *Neoplecostomus botucatu* | -22,65029907 | -53,0788002 | LBPV-34836 | FUPR1429-10 | JN989071 |
| Siluriformes | Loricariidae | *Neoplecostomus corumba* | -22,63979912 | -48,05310059 | LBPV-33410 | FUPR1448-10 | JN989049 |
| Siluriformes | Loricariidae | *Neoplecostomus corumba* | -22,63979912 | -48,05310059 | LBPV-33411 | FUPR1449-10 | JN989048 |
| Siluriformes | Loricariidae | *Neoplecostomus corumba* | -22,63979912 | -48,05310059 | LBPV-33413 | FUPR1450-10 | JN989047 |
| Siluriformes | Loricariidae | *Neoplecostomus corumba* | -24,20509911 | -52,7983017 | LBPV-33415 | FUPR1451-10 | JN989046 |
| Siluriformes | Loricariidae | *Neoplecostomus langeanii* | -24,20509911 | -52,7983017 | LBPV-27990 | FUPR1417-10 | JN989092 |
| Siluriformes | Loricariidae | *Neoplecostomus langeanii* | -22,63260078 | -53,05260086 | LBPV-27991 | FUPR1418-10 | JN989091 |
| Siluriformes | Loricariidae | *Neoplecostomus langeanii* | -22,63260078 | -53,05260086 | LBPV-29260 | FUPR1419-10 | JN989090 |
| Siluriformes | Loricariidae | *Neoplecostomus langeanii* | -22,63260078 | -53,05260086 | LBPV-29290 | FUPR1420-10 | JN989089 |
| Siluriformes | Loricariidae | *Neoplecostomus langeanii* | -23,52700043 | -45,7621994 | LBPV-29291 | FUPR1421-10 | JN989088 |
| Siluriformes | Loricariidae | *Neoplecostomus paranensis* | -23,5237999 | -45,88959885 | LBPV-17438 | FUPR1376-10 | JN989053 |
| Siluriformes | Loricariidae | *Neoplecostomus paranensis* | -23,5237999 | -45,88959885 | LBPV-17445 | FUPR1377-10 | JN989052 |
| Siluriformes | Loricariidae | *Neoplecostomus paranensis* | -23,5237999 | -45,88959885 | LBPV-17447 | FUPR1378-10 | JN989051 |
| Siluriformes | Loricariidae | *Neoplecostomus paranensis* | -23,77029991 | -46,31100082 | LBPV-17449 | FUPR1379-10 | JN989050 |
| Siluriformes | Loricariidae | *Neoplecostomus selenae* | -22,63260078 | -53,05260086 | LBPV-34842 | FUPR634-09 | GU701614 |
| Siluriformes | Loricariidae | *Neoplecostomus selenae* | -22,63260078 | -53,05260086 | LBPV-34843 | FUPR635-09 | GU701617 |
| Siluriformes | Loricariidae | *Neoplecostomus selenae* | -22,63260078 | -53,05260086 | LBPV-34844 | FUPR636-09 | GU701616 |
| Siluriformes | Loricariidae | *Neoplecostomus selenae* | -22,63260078 | -53,05260086 | LBPV-34845 | FUPR637-09 | GU701615 |
| Siluriformes | Loricariidae | *Neoplecostomus selenae* | -23,23060036 | -48,53279877 | LBPV-34846 | FUPR638-09 | GU701618 |
| Siluriformes | Loricariidae | *Neoplecostomus sp.* | -21,62409973 | -47,80630112 | LBPV-44849 | FUPR1334-10 | JN989056 |
| Siluriformes | Loricariidae | *Neoplecostomus sp.* | -21,62409973 | -47,80630112 | LBPV-44871 | FUPR1335-10 | JN989055 |
| Siluriformes | Loricariidae | *Neoplecostomus sp.* | -21,62409973 | -47,80630112 | LBPV-44872 | FUPR1336-10 | JN989054 |
| Siluriformes | Loricariidae | *Neoplecostomus sp1* | -21,62409973 | -47,80630112 | LBPV-29832 | FUPR1390-10 | JN989060 |
| Siluriformes | Loricariidae | *Neoplecostomus sp1* | -22,63260078 | -53,05260086 | LBPV-29833 | FUPR1391-10 | JN989059 |
| Siluriformes | Loricariidae | *Neoplecostomus sp1* | -22,86910057 | -48,36759949 | LBPV-29835 | FUPR1392-10 | JN989058 |
| Siluriformes | Loricariidae | *Neoplecostomus sp1* | -22,86910057 | -48,36759949 | LBPV-29845 | FUPR1393-10 | JN989057 |
| Siluriformes | Loricariidae | *Neoplecostomus sp1* | -22,86910057 | -48,36759949 | LBPV-29846 | FUPR1394-10 | JN989065 |
| Siluriformes | Loricariidae | *Neoplecostomus sp1* | -22,6692009 | -53,09289932 | LBPV-31514 | FUPR1395-10 | JN989064 |
| Siluriformes | Loricariidae | *Neoplecostomus sp1* | -22,6692009 | -53,09289932 | LBPV-33432 | FUPR1396-10 | JN989063 |
| Siluriformes | Loricariidae | *Neoplecostomus sp1* | -22,72949982 | -53,31779861 | LBPV-33433 | FUPR1397-10 | JN989062 |
| Siluriformes | Loricariidae | *Neoplecostomus sp1* | -22,72949982 | -53,31779861 | LBPV-33435 | FUPR1398-10 | JN989061 |
| Siluriformes | Loricariidae | *Neoplecostomus sp10* | -22,72949982 | -53,31779861 | LBPV-33407 | FUPR1414-10 | JN989068 |
| Siluriformes | Loricariidae | *Neoplecostomus sp10* | -22,89940071 | -48,38669968 | LBPV-33408 | FUPR1415-10 | JN989067 |
| Siluriformes | Loricariidae | *Neoplecostomus sp10* | -22,89940071 | -48,38669968 | LBPV-33409 | FUPR1416-10 | JN989066 |
| Siluriformes | Loricariidae | *Neoplecostomus sp10* | -22,72949982 | -53,31779861 | LBPV-33421 | FUPR1412-10 | JN989070 |
| Siluriformes | Loricariidae | *Neoplecostomus sp10* | -22,72949982 | -53,31779861 | LBPV-33425 | FUPR1413-10 | JN989069 |
| Siluriformes | Loricariidae | *Neoplecostomus sp2* | -22,89940071 | -48,38669968 | LBPV-33436 | FUPR1385-10 | JN989077 |
| Siluriformes | Loricariidae | *Neoplecostomus sp2* | -22,89940071 | -48,38669968 | LBPV-33439 | FUPR1386-10 | JN989076 |
| Siluriformes | Loricariidae | *Neoplecostomus sp3* | -22,79140091 | -53,34939957 | LBPV-33403 | FUPR1387-10 | JN989080 |
| Siluriformes | Loricariidae | *Neoplecostomus sp3* | -22,79140091 | -53,34939957 | LBPV-33404 | FUPR1388-10 | JN989079 |
| Siluriformes | Loricariidae | *Neoplecostomus sp3* | -21,10700035 | -50,26459885 | LBPV-33405 | FUPR1389-10 | JN989078 |
| Siluriformes | Loricariidae | *Neoplecostomus sp4* | -21,10700035 | -50,26459885 | LBPV-32377 | FUPR1380-10 | JN989085 |
| Siluriformes | Loricariidae | *Neoplecostomus sp4* | -21,10700035 | -50,26459885 | LBPV-32378 | FUPR1381-10 | JN989084 |
| Siluriformes | Loricariidae | *Neoplecostomus sp4* | -23,77129936 | -46,76570129 | LBPV-32379 | FUPR1382-10 | JN989083 |
| Siluriformes | Loricariidae | *Neoplecostomus sp4* | -25,4197998 | -54,53559875 | LBPV-32382 | FUPR1383-10 | JN989082 |
| Siluriformes | Loricariidae | *Neoplecostomus sp4* | -25,4197998 | -54,53559875 | LBPV-32383 | FUPR1384-10 | JN989081 |
| Siluriformes | Loricariidae | *Neoplecostomus sp5* | -21,24559975 | -48,29729843 | LBPV-29991 | FUPR1422-10 | JN989087 |
| Siluriformes | Loricariidae | *Neoplecostomus sp5* | -21,24559975 | -48,29729843 | LBPV-29992 | FUPR1423-10 | JN989086 |
| Siluriformes | Loricariidae | *Neoplecostomus sp8* | -21,24559975 | -48,29729843 | LBPV-33426 | FUPR1404-10 | JN989102 |
| Siluriformes | Loricariidae | *Neoplecostomus sp8* | -21,24559975 | -48,29729843 | LBPV-33427 | FUPR1405-10 | JN989101 |
| Siluriformes | Loricariidae | *Neoplecostomus sp8* | -21,24559975 | -48,29729843 | LBPV-33428 | FUPR1406-10 | JN989100 |
| Siluriformes | Loricariidae | *Neoplecostomus sp8* | -22,44890022 | -45,34640121 | LBPV-33429 | FUPR1407-10 | JN989099 |
| Siluriformes | Loricariidae | *Neoplecostomus sp8* | -22,44890022 | -45,34640121 | LBPV-33442 | FUPR1408-10 | JN989098 |
| Siluriformes | Loricariidae | *Neoplecostomus sp9* | -22,71759987 | -53,29100037 | LBPV-10228 | FUPR1409-10 | JN989105 |
| Siluriformes | Loricariidae | *Neoplecostomus sp9* | -22,71759987 | -53,29100037 | LBPV-10244 | FUPR1410-10 | JN989104 |
| Siluriformes | Loricariidae | *Neoplecostomus sp9* | -21,97450066 | -47,41820145 | LBPV-10248 | FUPR1411-10 | JN989103 |
| Siluriformes | Loricariidae | *Neoplecostomus yapo* | -22,71759987 | -53,29100037 | LBPV-24680 | FUPR1430-10 | JN989109 |
| Siluriformes | Loricariidae | *Neoplecostomus yapo* | -22,71759987 | -53,29100037 | LBPV-24681 | FUPR1431-10 | JN989108 |
| Siluriformes | Loricariidae | *Neoplecostomus yapo* | -22,7220993 | -53,28649902 | LBPV-29762 | FUPR1432-10 | JN989107 |
| Siluriformes | Loricariidae | *Neoplecostomus yapo* | -22,79140091 | -53,34939957 | LBPV-29907 | FUPR1433-10 | JN989106 |
| Siluriformes | Loricariidae | *Otothyropsis marapoama* | -22,65029907 | -53,0788002 | LBPV-23268 | FUPR642-09 | GU701608 |
| Siluriformes | Loricariidae | *Otothyropsis marapoama* | -21,17679977 | -49,17250061 | LBPV-24604 | FUPR643-09 | GU701607 |
| Siluriformes | Loricariidae | *Pareiorhina carrancas* | -22,79140091 | -53,34939957 | LBPV-37564 | FUPR846-09 | GU701595 |
| Siluriformes | Loricariidae | *Pareiorhina carrancas* | -22,79140091 | -53,34939957 | LBPV-37565 | FUPR845-09 | GU701596 |
| Siluriformes | Loricariidae | *Pareiorhina carrancas* | -22,93919945 | -50,25230026 | LBPV-37566 | FUPR848-09 | GU701598 |
| Siluriformes | Loricariidae | *Pareiorhina carrancas* | -22,79140091 | -53,34939957 | LBPV-37567 | FUPR844-09 | GU701593 |
| Siluriformes | Loricariidae | *Pareiorhina carrancas* | - | - | LBPV-37568 | FUPR847-09 | GU701599 |
| Siluriformes | Loricariidae | *Pareiorhina sp.* | -22,93919945 | -50,25230026 | LBPV-24190 | FUPR644-09 | GU701590 |
| Siluriformes | Loricariidae | *Pareiorhina sp.* | -19,68330002 | -48,66910172 | LBPV-24191 | FUPR645-09 | GU701589 |
| Siluriformes | Loricariidae | *Pareiorhina sp.* | -23,57640076 | -45,97449875 | LBPV-24210 | FUPR646-09 | GU701592 |
| Siluriformes | Loricariidae | *Pareiorhina sp.* | -23,57640076 | -45,97449875 | LBPV-24211 | FUPR647-09 | GU701895 |
| Siluriformes | Loricariidae | *Pareiorhina sp.* | -23,57640076 | -45,97449875 | LBPV-24212 | FUPR648-09 | GU701594 |
| Siluriformes | Loricariidae | *Proloricaria prolixa* | -21,92709923 | -47,36790085 | LBPV-34923 | FUPR761-09 | GU701560 |
| Siluriformes | Loricariidae | *Proloricaria prolixa* | -22,3784008 | -47,21049881 | LBPV-34924 | FUPR762-09 | JN989166 |
| Siluriformes | Loricariidae | *Proloricaria prolixa* | -22,3784008 | -47,21049881 | LBPV-34925 | FUPR763-09 | JN989167 |
| Siluriformes | Loricariidae | *Proloricaria prolixa* | -22,3784008 | -47,21049881 | LBPV-34926 | FUPR764-09 | HM899913 |
| Siluriformes | Loricariidae | *Proloricaria prolixa* | -22,3784008 | -47,21049881 | LBPV-34927 | FUPR765-09 | JN989168 |
| Siluriformes | Loricariidae | *Proloricaria prolixa* | -22,3784008 | -47,21049881 | LBPV-34928 | FUPR766-09 | GU701561 |
| Siluriformes | Loricariidae | *Pseudotocinclus tietensis* | -23,55550003 | -46,04069901 | LBPV-21475 | FUPR299-09 | JN989176 |
| Siluriformes | Loricariidae | *Pseudotocinclus tietensis* | -15,73690033 | -47,9151001 | LBPV-21476 | FUPR808-09 | JN989178 |
| Siluriformes | Loricariidae | *Pseudotocinclus tietensis* | -15,73690033 | -47,9151001 | LBPV-21477 | FUPR300-09 | JN989177 |
| Siluriformes | Loricariidae | *Pterygoplichthys anisitsi* | -15,71689987 | -47,86569977 | LBPV-26349 | FUPR653-09 | GU701547 |
| Siluriformes | Loricariidae | *Pterygoplichthys anisitsi* | -15,58170033 | -47,50640106 | LBPV-26685 | FUPR788-09 | GU701551 |
| Siluriformes | Loricariidae | *Pterygoplichthys anisitsi* | -15,71689987 | -47,86569977 | LBPV-26686 | FUPR652-09 | GU701548 |
| Siluriformes | Loricariidae | *Pterygoplichthys anisitsi* | -15,58170033 | -47,50640106 | LBPV-31754 | FUPR789-09 | GU701550 |
| Siluriformes | Loricariidae | *Pterygoplichthys anisitsi* | -15,58199978 | -47,50600052 | LBPV-31755 | FUPR790-09 | JN989182 |
| Siluriformes | Loricariidae | *Pterygoplichthys anisitsi* | -15,71689987 | -47,86569977 | LBPV-32034 | FUPR649-09 | GU701546 |
| Siluriformes | Loricariidae | *Pterygoplichthys anisitsi* | -15,71689987 | -47,86569977 | LBPV-32035 | FUPR650-09 | GU701545 |
| Siluriformes | Loricariidae | *Pterygoplichthys anisitsi* | -15,71689987 | -47,86569977 | LBPV-32036 | FUPR651-09 | GU701549 |
| Siluriformes | Loricariidae | *Rhinelepis aspera* | -22,04380035 | -53,72740173 | LBPV-26751 | FUPR813-09 | JN989194 |
| Siluriformes | Loricariidae | *Rhinelepis aspera* | -22,04380035 | -53,72740173 | LBPV-26754 | FUPR814-09 | HM376400 |
| Siluriformes | Loricariidae | *Rhinelepis aspera* | -22,04380035 | -53,72740173 | LBPV-26755 | FUPR815-09 | JN989195 |
| Siluriformes | Loricariidae | *Rhinelepis aspera* | -22,04380035 | -53,72740173 | LBPV-31974 | FUPR811-09 | JN989193 |
| Siluriformes | Loricariidae | *Rhinolekos britskii* | -21,89669991 | -53,79029846 | LBPV-34406 | FUPR625-09 | GU701621 |
| Siluriformes | Loricariidae | *Rhinolekos britskii* | -21,89669991 | -53,79029846 | LBPV-34407 | FUPR626-09 | GU701625 |
| Siluriformes | Loricariidae | *Rhinolekos britskii* | -21,89669991 | -53,79029846 | LBPV-34421 | FUPR627-09 | GU701624 |
| Siluriformes | Loricariidae | *Rhinolekos britskii* | -21,89669991 | -53,79029846 | LBPV-34422 | FUPR628-09 | GU701628 |
| Siluriformes | Loricariidae | *Rhinolekos sp.* | -22,23329926 | -53,78409958 | LBPV-33303 | FUPR633-09 | GU701622 |
| Siluriformes | Loricariidae | *Rhinolekos sp.* | -23,23060036 | -48,53279877 | LBPV-34401 | FUPR629-09 | GU701619 |
| Siluriformes | Loricariidae | *Rhinolekos sp.* | -22,04789925 | -53,69390106 | LBPV-34402 | FUPR630-09 | GU701620 |
| Siluriformes | Loricariidae | *Rhinolekos sp.* | -22,04789925 | -53,69390106 | LBPV-34403 | FUPR631-09 | JN989033 |
| Siluriformes | Loricariidae | *Rhinolekos sp.* | -22,23329926 | -53,78409958 | LBPV-34404 | FUPR632-09 | GU701623 |
| Siluriformes | Loricariidae | *Rineloricaria latirostris* | -21,88419914 | -53,75360107 | LBPV-19533 | FUPR656-09 | GU701523 |
| Siluriformes | Loricariidae | *Rineloricaria latirostris* | -21,98049927 | -53,71440125 | LBPV-19534 | FUPR657-09 | GU701526 |
| Siluriformes | Loricariidae | *Rineloricaria latirostris* | -22,25819969 | -53,81069946 | LBPV-26332 | FUPR654-09 | GU701520 |
| Siluriformes | Loricariidae | *Rineloricaria latirostris* | -21,88419914 | -53,75360107 | LBPV-26333 | FUPR655-09 | GU701524 |
| Siluriformes | Loricariidae | *Rineloricaria latirostris* | -21,93129921 | -47,375 | LBPV-38088 | FUPR975-09 | GU701525 |
| Siluriformes | Loricariidae | *Rineloricaria pentamaculata* | -21,93129921 | -47,375 | LBPV-29769 | FUPR663-09 | GU701521 |
| Siluriformes | Loricariidae | *Rineloricaria pentamaculata* | -21,93129921 | -47,375 | LBPV-34798 | FUPR658-09 | GU701881 |
| Siluriformes | Loricariidae | *Rineloricaria pentamaculata* | -21,93129921 | -47,375 | LBPV-34799b | FUPR1026-09 | GU701420 |
| Siluriformes | Loricariidae | *Rineloricaria pentamaculata* | -21,93129921 | -47,375 | LBPV-34800 | FUPR660-09 | JN989197 |
| Siluriformes | Loricariidae | *Rineloricaria pentamaculata* | -21,93129921 | -47,375 | LBPV-35401 | FUPR661-09 | JN989198 |
| Siluriformes | Loricariidae | *Rineloricaria pentamaculata* | -21,93129921 | -47,375 | LBPV-35402 | FUPR662-09 | GU701522 |
| Siluriformes | Pimelodidae | *Hypophthalmus oremaculatus* | -21,0128994 | -49,69029999 | LBPV-36613 | FUPR664-09 | GU701736 |
| Siluriformes | Pimelodidae | *Hypophthalmus oremaculatus* | -21,0128994 | -49,69029999 | LBPV-36617 | FUPR665-09 | GU701735 |
| Siluriformes | Pimelodidae | *Hypophthalmus oremaculatus* | -21,06669998 | -50,11500168 | LBPV-37265 | FUPR666-09 | JN988932 |
| Siluriformes | Pimelodidae | *Iheringichthys labrosus* | -22,79140091 | -53,34939957 | LBPV-29269 | FUPR671-09 | HM376396 |
| Siluriformes | Pimelodidae | *Iheringichthys labrosus* | -22,79140091 | -53,34939957 | LBPV-37208 | FUPR667-09 | GU701872 |
| Siluriformes | Pimelodidae | *Iheringichthys labrosus* | -22,79140091 | -53,34939957 | LBPV-37209 | FUPR668-09 | GU701873 |
| Siluriformes | Pimelodidae | *Iheringichthys labrosus* | -22,79140091 | -53,34939957 | LBPV-37210 | FUPR669-09 | GU701657 |
| Siluriformes | Pimelodidae | *Iheringichthys labrosus* | -22,79140091 | -53,34939957 | LBPV-37211 | FUPR670-09 | GU701660 |
| Siluriformes | Pimelodidae | *Pimelodus maculatus* | -22,63209915 | -48,17509842 | LBPV-29302 | FUPR672-09 | GU701571 |
| Siluriformes | Pimelodidae | *Pimelodus maculatus* | -22,63209915 | -48,17509842 | LBPV-32040 | FUPR673-09 | GU701570 |
| Siluriformes | Pimelodidae | *Pimelodus maculatus* | -22,63209915 | -48,17509842 | LBPV-37266 | FUPR674-09 | JN989127 |
| Siluriformes | Pimelodidae | *Pinirampus pirinampu* | -21,10700035 | -50,26459885 | LBPV-26733 | FUPR675-09 | GU701563 |
| Siluriformes | Pimelodidae | *Pinirampus pirinampu* | -23,57640076 | -45,97449875 | LBPV-26734 | FUPR676-09 | GU701567 |
| Siluriformes | Pimelodidae | *Pinirampus pirinampu* | -22,71719933 | -53,31779861 | LBPV-26735 | FUPR677-09 | GU701566 |
| Siluriformes | Pimelodidae | *Pinirampus pirinampu* | -22,71719933 | -53,31779861 | LBPV-26736 | FUPR678-09 | GU701900 |
| Siluriformes | Pimelodidae | *Pinirampus pirinampu* | -21,62409973 | -47,80630112 | LBPV-26737 | FUPR679-09 | GU701565 |
| Siluriformes | Pimelodidae | *Pinirampus pirinampu* | -20,23609924 | -48,67829895 | LBPV-36615 | FUPR680-09 | GU701569 |
| Siluriformes | Pimelodidae | *Pinirampus pirinampu* | -21,62409973 | -47,80630112 | LBPV-36945 | FUPR681-09 | GU701568 |
| Siluriformes | Pimelodidae | *Pseudoplatystoma corruscans* | -22,86260033 | -48,10580063 | LBPV-26757 | FUPR298-09 | JN989174 |
| Siluriformes | Pimelodidae | *Pseudoplatystoma corruscans* | -22,86260033 | -48,10580063 | LBPV-36614 | FUPR684-09 | GU701553 |
| Siluriformes | Pimelodidae | *Pseudoplatystoma corruscans* | -22,86260033 | -48,10580063 | LBPV-36616 | FUPR685-09 | GU701555 |
| Siluriformes | Pimelodidae | *Pseudoplatystoma corruscans* | -22,86260033 | -48,10580063 | LBPV-38169 | FUPR836-09 | GU701558 |
| Siluriformes | Pimelodidae | *Pseudoplatystoma reticulatum* | -23,57640076 | -45,97449875 | LBPV-36610 | FUPR682-09 | GU701554 |
| Siluriformes | Pimelodidae | *Pseudoplatystoma reticulatum* | -22,78560066 | -48,48149872 | LBPV-36611 | FUPR683-09 | JN989175 |
| Siluriformes | Pimelodidae | *Steindachneridion scriptum* | -24,10400009 | -52,32500076 | LBPV-25638 | FUPR686-09 | HM376397 |
| Siluriformes | Pimelodidae | *Zungaro jahu* | -22,72960091 | -53,31779861 | LBPV-15141 | FUPR689-09 | GU701452 |
| Siluriformes | Pimelodidae | *Zungaro jahu* | -22,72960091 | -53,31779861 | LBPV-15142 | FUPR690-09 | GU701456 |
| Siluriformes | Pimelodidae | *Zungaro jahu* | -21,10700035 | -50,26459885 | LBPV-15143 | FUPR691-09 | GU701455 |
| Siluriformes | Pimelodidae | *Zungaro jahu* | -22,72960091 | -53,31779861 | LBPV-26717 | FUPR687-09 | GU701453 |
| Siluriformes | Pimelodidae | *Zungaro jahu* | -22,72960091 | -53,31779861 | LBPV-26738 | FUPR688-09 | GU701896 |
| Siluriformes | Pimelodidae | *Zungaro jahu* | -21,10700035 | -50,26459885 | LBPV-36602 | FUPR692-09 | GU701454 |
| Siluriformes | Pseudopimelodidae | *Microglanis garavelloi* | - | - | LBPV-10557 | FUPR695-09 | GU701443 |
| Siluriformes | Pseudopimelodidae | *Microglanis garavelloi* | - | - | LBPV-10561 | FUPR696-09 | GU701629 |
| Siluriformes | Pseudopimelodidae | *Microglanis garavelloi* | - | - | LBPV-10562 | FUPR697-09 | JN989032 |
| Siluriformes | Pseudopimelodidae | *Microglanis garavelloi* | -21,44440079 | -44,60240173 | LBPV-22539 | FUPR693-09 | GU701627 |
| Siluriformes | Pseudopimelodidae | *Microglanis garavelloi* | - | - | LBPV-22540 | FUPR694-09 | GU701626 |
| Siluriformes | Pseudopimelodidae | *Pseudopimelodus mangurus* | - | - | LBPV-37226 | FUPR698-09 | GU701444 |
| Siluriformes | Pseudopimelodidae | *Pseudopimelodus mangurus* | - | - | LBPV-37227 | FUPR699-09 | GU701870 |
| Siluriformes | Pseudopimelodidae | *Pseudopimelodus mangurus* | - | - | LBPV-37228 | FUPR700-09 | GU701557 |
| Siluriformes | Pseudopimelodidae | *Pseudopimelodus sp.* | -22,86260033 | -48,10580063 | LBPV-17433 | FUPR702-09 | GU701556 |
| Siluriformes | Trichomycteridae | *Ituglanis sp.* | -17,73889923 | -48,4742012 | LBPV-35885 | FUPR703-09 | GU701643 |
| Siluriformes | Trichomycteridae | *Ituglanis sp.* | -17,09939957 | -48,76150131 | LBPV-35886 | FUPR704-09 | GU701642 |
| Siluriformes | Trichomycteridae | *Ituglanis sp.* | -17,09939957 | -48,76150131 | LBPV-35887 | FUPR705-09 | GU701646 |
| Siluriformes | Trichomycteridae | *Ituglanis sp.* | -17,09939957 | -48,76150131 | LBPV-35888 | FUPR706-09 | GU701645 |
| Siluriformes | Trichomycteridae | *Ituglanis sp.* | -21,19309998 | -49,12279892 | LBPV-35889 | FUPR707-09 | GU701644 |
| Siluriformes | Trichomycteridae | *Parastegophilus paulensis* | -22,7220993 | -53,28649902 | LBPV-34425 | FUPR708-09 | GU701597 |
| Siluriformes | Trichomycteridae | *Parastegophilus paulensis* | -22,7220993 | -53,28649902 | LBPV-34426 | FUPR709-09 | GU701601 |
| Siluriformes | Trichomycteridae | *Parastegophilus paulensis* | -22,7220993 | -53,28649902 | LBPV-34427 | FUPR710-09 | GU701600 |
| Siluriformes | Trichomycteridae | *Parastegophilus paulensis* | -22,79140091 | -53,34939957 | LBPV-38147 | FUPR711-09 | GU701957 |
| Siluriformes | Trichomycteridae | *Parastegophilus paulensis* | -22,79140091 | -53,34939957 | LBPV-38148 | FUPR712-09 | GU701959 |
| Siluriformes | Trichomycteridae | *Parastegophilus paulensis* | -22,79140091 | -53,34939957 | LBPV-38149 | FUPR713-09 | GU701960 |
| Siluriformes | Trichomycteridae | *Trichomycterus brasiliensis* | -21,625 | -47,80630112 | LBPV-31575 | FUPR715-09 | GU701479 |
| Siluriformes | Trichomycteridae | *Trichomycterus brasiliensis* | -21,625 | -47,80630112 | LBPV-31576 | FUPR716-09 | GU701478 |
| Siluriformes | Trichomycteridae | *Trichomycterus brasiliensis* | -20,94709969 | -48,14780045 | LBPV-31669 | FUPR714-09 | GU701475 |
| Siluriformes | Trichomycteridae | *Trichomycterus diabolus* | -20,23620033 | -48,67829895 | LBPV-19860 | FUPR301-09 | JN989255 |
| Siluriformes | Trichomycteridae | *Trichomycterus diabolus* | -20,23620033 | -48,67829895 | LBPV-19861 | FUPR302-09 | JN989256 |
| Siluriformes | Trichomycteridae | *Trichomycterus diabolus* | -23,52669907 | -45,81980133 | LBPV-21295 | FUPR310-09 | JN989259 |
| Siluriformes | Trichomycteridae | *Trichomycterus diabolus* | -22,79140091 | -53,34939957 | LBPV-21297 | FUPR303-09 | JN989257 |
| Siluriformes | Trichomycteridae | *Trichomycterus diabolus* | -23,52669907 | -45,81980133 | LBPV-21298 | FUPR304-09 | JN989258 |
| Siluriformes | Trichomycteridae | *Trichomycterus iheringi* | -18,42449951 | -48,06529999 | LBPV-19838 | FUPR743-09 | GU701412 |
| Siluriformes | Trichomycteridae | *Trichomycterus iheringi* | -24,0739994 | -52,29100037 | LBPV-21759 | FUPR751-09 | GU701892 |
| Siluriformes | Trichomycteridae | *Trichomycterus iheringi* | -25,09199905 | -52,49499893 | LBPV-21760 | FUPR752-09 | GU701893 |
| Siluriformes | Trichomycteridae | *Trichomycterus iheringi* | - | - | LBPV-21761 | FUPR753-09 | GU701476 |
| Siluriformes | Trichomycteridae | *Trichomycterus iheringi* | -22,72999954 | -53,31800079 | LBPV-21802 | FUPR749-09 | GU701474 |
| Siluriformes | Trichomycteridae | *Trichomycterus iheringi* | -24,0739994 | -52,29100037 | LBPV-21803 | FUPR750-09 | GU701477 |
| Siluriformes | Trichomycteridae | *Trichomycterus iheringi* | -18,42449951 | -48,06529999 | LBPV-24562 | FUPR745-09 | GU701468 |
| Siluriformes | Trichomycteridae | *Trichomycterus iheringi* | -21,10700035 | -50,26499939 | LBPV-24563 | FUPR746-09 | GU701467 |
| Siluriformes | Trichomycteridae | *Trichomycterus iheringi* | -21,10700035 | -50,26499939 | LBPV-24564 | FUPR747-09 | GU701466 |
| Siluriformes | Trichomycteridae | *Trichomycterus iheringi* | -22,72999954 | -53,31800079 | LBPV-24565 | FUPR748-09 | GU701469 |
| Siluriformes | Trichomycteridae | *Trichomycterus iheringi* | -18,42449951 | -48,06529999 | LBPV-25889 | FUPR744-09 | GU701464 |
| Siluriformes | Trichomycteridae | *Trichomycterus iheringi* | -18,42449951 | -48,06529999 | LBPV-7645 | FUPR723-09 | JN989260 |
| Siluriformes | Trichomycteridae | *Trichomycterus iheringi* | -18,42449951 | -48,06529999 | LBPV-7647 | FUPR725-09 | JN989261 |
| Siluriformes | Trichomycteridae | *Trichomycterus iheringi* | -23,52669907 | -45,81980133 | LBPV-7660 | FUPR722-09 | GU701473 |
| Siluriformes | Trichomycteridae | *Trichomycterus maracaya* | -21,92709923 | -47,36790085 | LBPV-29274 | FUPR305-09 | JN989262 |
| Siluriformes | Trichomycteridae | *Trichomycterus maracaya* | -22,71759987 | -53,29100037 | LBPV-29275 | FUPR306-09 | JN989263 |
| Siluriformes | Trichomycteridae | *Trichomycterus maracaya* | -22,71759987 | -53,29100037 | LBPV-29276 | FUPR307-09 | JN989264 |
| Siluriformes | Trichomycteridae | *Trichomycterus maracaya* | -22,93540001 | -48,32210159 | LBPV-29277 | FUPR308-09 | JN989265 |
| Siluriformes | Trichomycteridae | *Trichomycterus maracaya* | -22,93540001 | -48,32210159 | LBPV-29278 | FUPR309-09 | JN989266 |
| Siluriformes | Trichomycteridae | *Trichomycterus mimonha* | -22,64389992 | -52,81499863 | LBPV-37823 | FUPR738-09 | GU701472 |
| Siluriformes | Trichomycteridae | *Trichomycterus mimonha* | -22,64220047 | -52,81639862 | LBPV-37824 | FUPR739-09 | GU701471 |
| Siluriformes | Trichomycteridae | *Trichomycterus mimonha* | -22,64220047 | -52,81639862 | LBPV-37825 | FUPR740-09 | GU701470 |
| Siluriformes | Trichomycteridae | *Trichomycterus mimonha* | -22,64389992 | -52,81499863 | LBPV-37826 | FUPR733-09 | GU701460 |
| Siluriformes | Trichomycteridae | *Trichomycterus mimonha* | -22,64389992 | -52,81499863 | LBPV-37827 | FUPR734-09 | GU701459 |
| Siluriformes | Trichomycteridae | *Trichomycterus mimonha* | -22,64389992 | -52,81499863 | LBPV-37828 | FUPR735-09 | GU701463 |
| Siluriformes | Trichomycteridae | *Trichomycterus mimonha* | -22,64389992 | -52,81499863 | LBPV-37829 | FUPR736-09 | GU701462 |
| Siluriformes | Trichomycteridae | *Trichomycterus paolence* | -22,36860085 | -47,47719955 | LBPV-36308 | FUPR726-09 | HM376398 |
| Siluriformes | Trichomycteridae | *Trichomycterus pauciradiatus* | -22,36860085 | -47,47719955 | LBPV-18617 | FUPR717-09 | GU701958 |
| Siluriformes | Trichomycteridae | *Trichomycterus pauciradiatus* | -22,36420059 | -47,5135994 | LBPV-38145 | FUPR842-09 | GU701413 |
| Siluriformes | Trichomycteridae | *Trichomycterus pauciradiatus* | -22,36420059 | -47,5135994 | LBPV-38146 | FUPR843-09 | GU701414 |
| Siluriformes | Trichomycteridae | *Trichomycterus sp1* | -23,23060036 | -48,53279877 | LBPV-47965 | FUPR1456-10 | JN989268 |
| Siluriformes | Trichomycteridae | *Trichomycterus sp1* | - | - | LBPV-47976 | FUPR1457-10 | JN989267 |
| Siluriformes | Trichomycteridae | *Trichomycterus sp2* | -22,63969994 | -48,05310059 | LBPV-47987 | FUPR1458-10 | JN989272 |
| Siluriformes | Trichomycteridae | *Trichomycterus sp2* | -22,63969994 | -48,05310059 | LBPV-47988 | FUPR1459-10 | JN989271 |
| Siluriformes | Trichomycteridae | *Trichomycterus sp2* | -22,63969994 | -48,05310059 | LBPV-47989 | FUPR1460-10 | JN989270 |
| Siluriformes | Trichomycteridae | *Trichomycterus sp2* | -22,63969994 | -48,05310059 | LBPV-47990 | FUPR1461-10 | JN989269 |
| Siluriformes | Trichomycteridae | *Trichomycterus sp3* | -22,63969994 | -48,05310059 | LBPV-47992 | FUPR1462-10 | JN989275 |
| Siluriformes | Trichomycteridae | *Trichomycterus sp3* | -21,62409973 | -47,80630112 | LBPV-47994 | FUPR1463-10 | JN989274 |
| Siluriformes | Trichomycteridae | *Trichomycterus sp3* | -21,62409973 | -47,80630112 | LBPV-47995 | FUPR1464-10 | JN989273 |
| Siluriformes | Trichomycteridae | *Trichomycterus sp4* | -23,9545002 | -51,11410141 | LBPV-47075 | FUPR1465-10 | JN989276 |
| Siluriformes | Trichomycteridae | *Trichomycterus sp4* | -21,62409973 | -47,80630112 | LBPV-47966 | FUPR1466-10 | JN989277 |
| Siluriformes | Trichomycteridae | *Trichomycterus sp5* | -23,9545002 | -51,11410141 | LBPV-47993 | FUPR1467-10 | JN989278 |
| Siluriformes | Trichomycteridae | *Trichomycterus sp6* | -23,9545002 | -51,11410141 | LBPV-47991 | FUPR1468-10 | JN989279 |
| Siluriformes | Trichomycteridae | *Trichomycterus vermiculatus* | -17,12000084 | -48,74000168 | LBPV-29199 | FUPR737-09 | GU701465 |
| Synbranchiformes | Synbranchidae | *Synbranchus marmoratus* | -20,23699951 | -46,6629982 | LBPV-29988 | FUPR961-09 | GU701493 |
| Synbranchiformes | Synbranchidae | *Synbranchus marmoratus* | -20,14599991 | -46,66999817 | LBPV-29989 | FUPR962-09 | GU701497 |
| Synbranchiformes | Synbranchidae | *Synbranchus marmoratus* | -20,14599991 | -46,66999817 | LBPV-29990 | FUPR963-09 | GU701496 |
| Synbranchiformes | Synbranchidae | *Synbranchus marmoratus* | -20,14599991 | -46,66999817 | LBPV-31797 | FUPR956-09 | GU701492 |
| Synbranchiformes | Synbranchidae | *Synbranchus marmoratus* | -20,14599991 | -46,66999817 | LBPV-31798 | FUPR957-09 | GU701491 |
| Synbranchiformes | Synbranchidae | *Synbranchus marmoratus* | -20,14599991 | -46,66999817 | LBPV-31799 | FUPR958-09 | GU701494 |
| Synbranchiformes | Synbranchidae | *Synbranchus marmoratus* | -20,14599991 | -46,66999817 | LBPV-31957 | FUPR959-09 | HM902742 |
| Synbranchiformes | Synbranchidae | *Synbranchus marmoratus* | -20,28700066 | -46,58200073 | LBPV-32293 | FUPR960-09 | HM422472 |
