# Supplementary material for: Can DNA barcoding accurately discriminate megadiverse Neotropical freshwater fish fauna?
Source: BMC Genet. 2013 Mar 9;14:20. doi: 10.1186/1471-2156-14-20 (PMC3608943; doi:10.1186/1471-2156-14-20)
Supplement: Additional file 2 — NJ dendrogram of the 1,244 specimens (254 species) analyzed. Node values = bootstrap test (1,000 pseudo-replicas). [file 1471-2156-14-20-S2.pdf]

Additional file 2 - NJ dendrogram of the 1,244 specimens (254 species) analyzed.

Node values = bootstrap test (1,000 pseudo-replicas).

Distance Model: Kimura-2-Paramater

Codon Positions: 1st, 2nd, 3rd

Sequence count: 1244

Species count: 254

Genus count: 127

Family count: 36

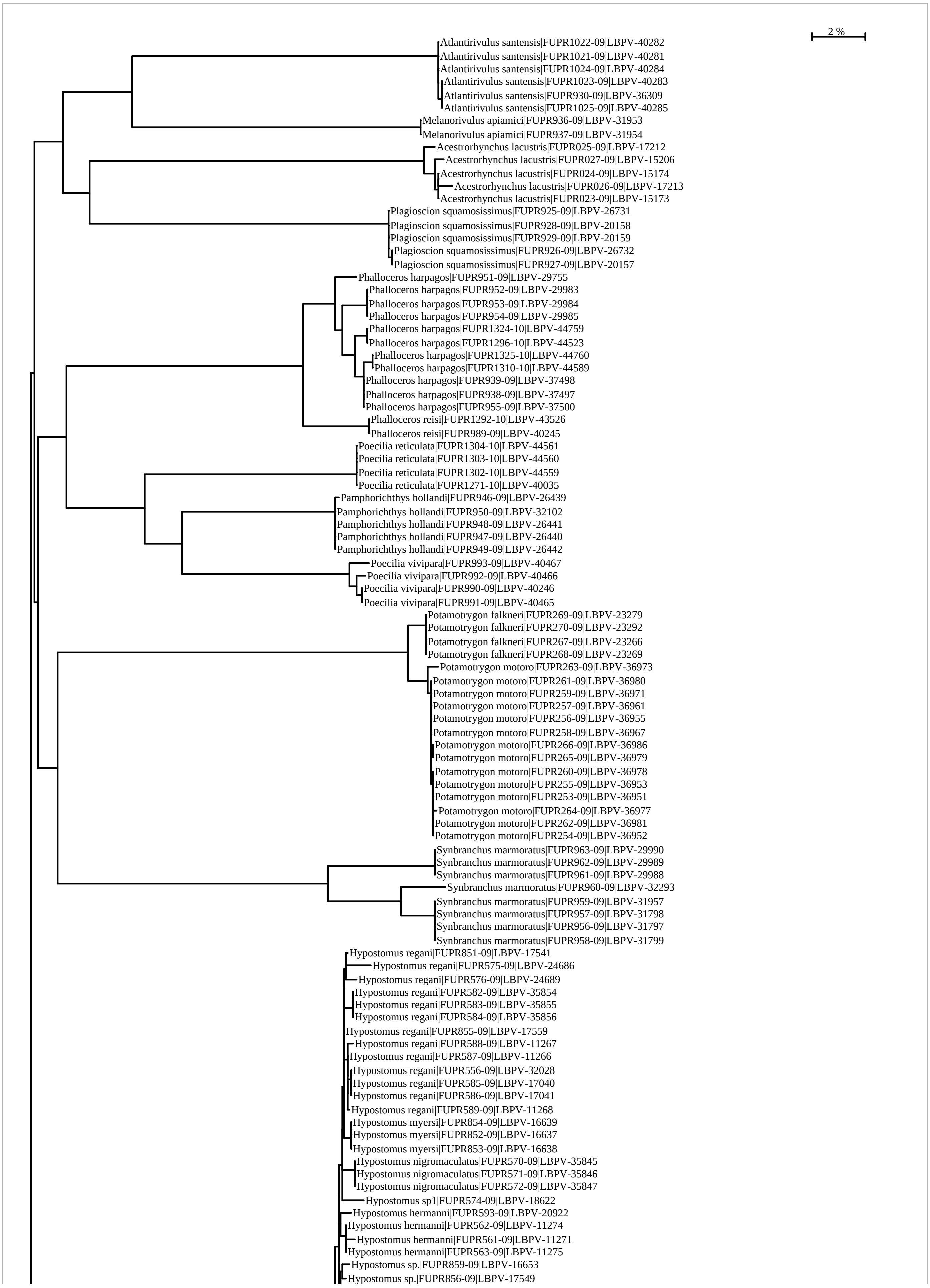
2 %
Atlantirivulus santensis|FUPR1022-09|LBPV-40282
Atlantirivulus santensis|FUPR1021-09|LBPV-40281
Atlantirivulus santensis|FUPR1024-09|LBPV-40284
Atlantirivulus santensis|FUPR1023-09|LBPV-40283
Atlantirivulus santensis|FUPR930-09|LBPV-36309
Atlantirivulus santensis|FUPR1025-09|LBPV-40285
Melanorivulus apiamici|FUPR936-09|LBPV-31953
Melanorivulus apiamici|FUPR937-09|LBPV-31954
Acestrorhynchus lacustris|FUPR025-09|LBPV-17212
Acestrorhynchus lacustris|FUPR027-09|LBPV-15206
Acestrorhynchus lacustris|FUPR024-09|LBPV-15174
Acestrorhynchus lacustris|FUPR026-09|LBPV-17213
Acestrorhynchus lacustris|FUPR023-09|LBPV-15173
Plagioscion squamosissimus|FUPR925-09|LBPV-26731
Plagioscion squamosissimus|FUPR928-09|LBPV-20158
Plagioscion squamosissimus|FUPR929-09|LBPV-20159
Plagioscion squamosissimus|FUPR926-09|LBPV-26732
Plagioscion squamosissimus|FUPR927-09|LBPV-20157
Phalloceros harpagos|FUPR951-09|LBPV-29755
Phalloceros harpagos|FUPR952-09|LBPV-29983
Phalloceros harpagos|FUPR953-09|LBPV-29984
Phalloceros harpagos|FUPR954-09|LBPV-29985
Phalloceros harpagos|FUPR1324-10|LBPV-44759
Phalloceros harpagos|FUPR1296-10|LBPV-44523
Phalloceros harpagos|FUPR1325-10|LBPV-44760
Phalloceros harpagos|FUPR1310-10|LBPV-44589
Phalloceros harpagos|FUPR939-09|LBPV-37498
Phalloceros harpagos|FUPR938-09|LBPV-37497
Phalloceros harpagos|FUPR955-09|LBPV-37500
Phalloceros reisi|FUPR1292-10|LBPV-43526
Phalloceros reisi|FUPR989-09|LBPV-40245
Poecilia reticulata|FUPR1304-10|LBPV-44561
Poecilia reticulata|FUPR1303-10|LBPV-44560
Poecilia reticulata|FUPR1302-10|LBPV-44559
Poecilia reticulata|FUPR1271-10|LBPV-40035
Pamphorichthys hollandi|FUPR946-09|LBPV-26439
Pamphorichthys hollandi|FUPR950-09|LBPV-32102
Pamphorichthys hollandi|FUPR948-09|LBPV-26441
Pamphorichthys hollandi|FUPR947-09|LBPV-26440
Pamphorichthys hollandi|FUPR949-09|LBPV-26442
Poecilia vivipara|FUPR993-09|LBPV-40467
Poecilia vivipara|FUPR992-09|LBPV-40466
Poecilia vivipara|FUPR990-09|LBPV-40246
Poecilia vivipara|FUPR991-09|LBPV-40465
Potamotrygon falkneri|FUPR269-09|LBPV-23279
Potamotrygon falkneri|FUPR270-09|LBPV-23292
Potamotrygon falkneri|FUPR267-09|LBPV-23266
Potamotrygon falkneri|FUPR268-09|LBPV-23269
Potamotrygon motoro|FUPR263-09|LBPV-36973
Potamotrygon motoro|FUPR261-09|LBPV-36980
Potamotrygon motoro|FUPR259-09|LBPV-36971
Potamotrygon motoro|FUPR257-09|LBPV-36961
Potamotrygon motoro|FUPR256-09|LBPV-36955
Potamotrygon motoro|FUPR258-09|LBPV-36967
Potamotrygon motoro|FUPR266-09|LBPV-36986
Potamotrygon motoro|FUPR265-09|LBPV-36979
Potamotrygon motoro|FUPR260-09|LBPV-36978
Potamotrygon motoro|FUPR255-09|LBPV-36953
Potamotrygon motoro|FUPR253-09|LBPV-36951
Potamotrygon motoro|FUPR264-09|LBPV-36977
Potamotrygon motoro|FUPR262-09|LBPV-36981
Potamotrygon motoro|FUPR254-09|LBPV-36952
Synbranchus marmoratus|FUPR963-09|LBPV-29990
Synbranchus marmoratus|FUPR962-09|LBPV-29989
Synbranchus marmoratus|FUPR961-09|LBPV-29988
Synbranchus marmoratus|FUPR960-09|LBPV-32293
Synbranchus marmoratus|FUPR959-09|LBPV-31957
Synbranchus marmoratus|FUPR957-09|LBPV-31798
Synbranchus marmoratus|FUPR956-09|LBPV-31797
Synbranchus marmoratus|FUPR958-09|LBPV-31799
Hypostomus regani|FUPR851-09|LBPV-17541
Hypostomus regani|FUPR575-09|LBPV-24686
Hypostomus regani|FUPR576-09|LBPV-24689
Hypostomus regani|FUPR582-09|LBPV-35854
Hypostomus regani|FUPR583-09|LBPV-35855
Hypostomus regani|FUPR584-09|LBPV-35856
Hypostomus regani|FUPR855-09|LBPV-17559
Hypostomus regani|FUPR588-09|LBPV-11267
Hypostomus regani|FUPR587-09|LBPV-11266
Hypostomus regani|FUPR556-09|LBPV-32028
Hypostomus regani|FUPR585-09|LBPV-17040
Hypostomus regani|FUPR586-09|LBPV-17041
Hypostomus regani|FUPR589-09|LBPV-11268
Hypostomus myersi|FUPR854-09|LBPV-16639
Hypostomus myersi|FUPR852-09|LBPV-16637
Hypostomus myersi|FUPR853-09|LBPV-16638
Hypostomus nigromaculatus|FUPR570-09|LBPV-35845
Hypostomus nigromaculatus|FUPR571-09|LBPV-35846
Hypostomus nigromaculatus|FUPR572-09|LBPV-35847
Hypostomus sp1|FUPR574-09|LBPV-18622
Hypostomus hermanni|FUPR593-09|LBPV-20922
Hypostomus hermanni|FUPR562-09|LBPV-11274
Hypostomus hermanni|FUPR561-09|LBPV-11271
Hypostomus hermanni|FUPR563-09|LBPV-11275
Hypostomus sp.|FUPR859-09|LBPV-16653
Hypostomus sp.|FUPR856-09|LBPV-17549

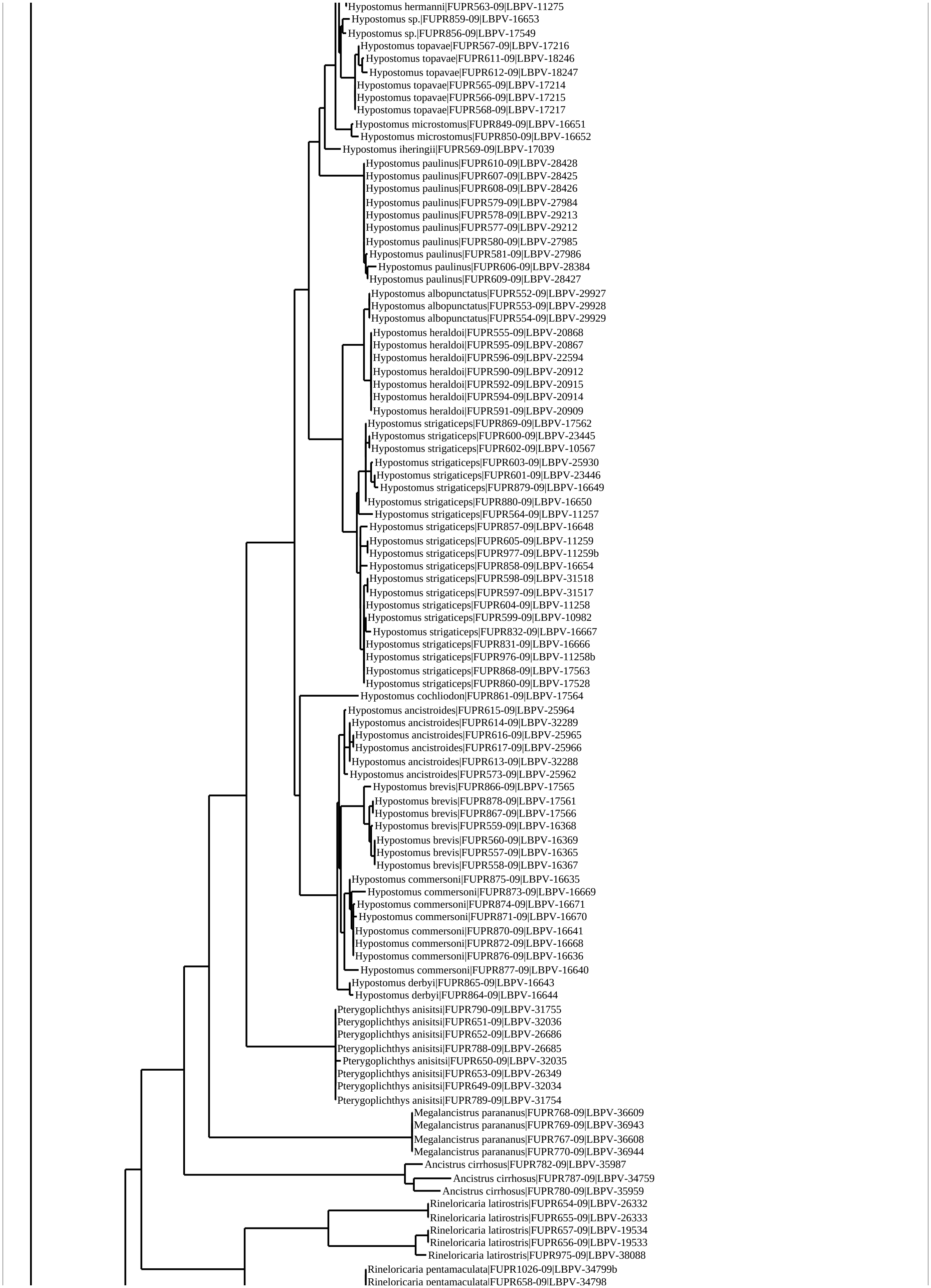
Hypostomus hermanni|FUPR563-09|LBPV-11275
Hypostomus sp.|FUPR859-09|LBPV-16653
Hypostomus sp.|FUPR856-09|LBPV-17549
Hypostomus topavae|FUPR567-09|LBPV-17216
Hypostomus topavae|FUPR611-09|LBPV-18246
Hypostomus topavae|FUPR612-09|LBPV-18247
Hypostomus topavae|FUPR565-09|LBPV-17214
Hypostomus topavae|FUPR566-09|LBPV-17215
Hypostomus topavae|FUPR568-09|LBPV-17217
Hypostomus microstomus|FUPR849-09|LBPV-16651
Hypostomus microstomus|FUPR850-09|LBPV-16652
Hypostomus iheringii|FUPR569-09|LBPV-17039
Hypostomus paulinus|FUPR610-09|LBPV-28428
Hypostomus paulinus|FUPR607-09|LBPV-28425
Hypostomus paulinus|FUPR608-09|LBPV-28426
Hypostomus paulinus|FUPR579-09|LBPV-27984
Hypostomus paulinus|FUPR578-09|LBPV-29213
Hypostomus paulinus|FUPR577-09|LBPV-29212
Hypostomus paulinus|FUPR580-09|LBPV-27985
Hypostomus paulinus|FUPR581-09|LBPV-27986
Hypostomus paulinus|FUPR606-09|LBPV-28384
Hypostomus paulinus|FUPR609-09|LBPV-28427
Hypostomus albopunctatus|FUPR552-09|LBPV-29927
Hypostomus albopunctatus|FUPR553-09|LBPV-29928
Hypostomus albopunctatus|FUPR554-09|LBPV-29929
Hypostomus heraldoi|FUPR555-09|LBPV-20868
Hypostomus heraldoi|FUPR595-09|LBPV-20867
Hypostomus heraldoi|FUPR596-09|LBPV-22594
Hypostomus heraldoi|FUPR590-09|LBPV-20912
Hypostomus heraldoi|FUPR592-09|LBPV-20915
Hypostomus heraldoi|FUPR594-09|LBPV-20914
Hypostomus heraldoi|FUPR591-09|LBPV-20909
Hypostomus strigaticeps|FUPR869-09|LBPV-17562
Hypostomus strigaticeps|FUPR600-09|LBPV-23445
Hypostomus strigaticeps|FUPR602-09|LBPV-10567
Hypostomus strigaticeps|FUPR603-09|LBPV-25930
Hypostomus strigaticeps|FUPR601-09|LBPV-23446
Hypostomus strigaticeps|FUPR879-09|LBPV-16649
Hypostomus strigaticeps|FUPR880-09|LBPV-16650
Hypostomus strigaticeps|FUPR564-09|LBPV-11257
Hypostomus strigaticeps|FUPR857-09|LBPV-16648
Hypostomus strigaticeps|FUPR605-09|LBPV-11259
Hypostomus strigaticeps|FUPR977-09|LBPV-11259b
Hypostomus strigaticeps|FUPR858-09|LBPV-16654
Hypostomus strigaticeps|FUPR598-09|LBPV-31518
Hypostomus strigaticeps|FUPR597-09|LBPV-31517
Hypostomus strigaticeps|FUPR604-09|LBPV-11258
Hypostomus strigaticeps|FUPR599-09|LBPV-10982
Hypostomus strigaticeps|FUPR832-09|LBPV-16667
Hypostomus strigaticeps|FUPR831-09|LBPV-16666
Hypostomus strigaticeps|FUPR976-09|LBPV-11258b
Hypostomus strigaticeps|FUPR868-09|LBPV-17563
Hypostomus strigaticeps|FUPR860-09|LBPV-17528
Hypostomus cochliodon|FUPR861-09|LBPV-17564
Hypostomus ancistroides|FUPR615-09|LBPV-25964
Hypostomus ancistroides|FUPR614-09|LBPV-32289
Hypostomus ancistroides|FUPR616-09|LBPV-25965
Hypostomus ancistroides|FUPR617-09|LBPV-25966
Hypostomus ancistroides|FUPR613-09|LBPV-32288
Hypostomus ancistroides|FUPR573-09|LBPV-25962
Hypostomus brevis|FUPR866-09|LBPV-17565
Hypostomus brevis|FUPR878-09|LBPV-17561
Hypostomus brevis|FUPR867-09|LBPV-17566
Hypostomus brevis|FUPR559-09|LBPV-16368
Hypostomus brevis|FUPR560-09|LBPV-16369
Hypostomus brevis|FUPR557-09|LBPV-16365
Hypostomus brevis|FUPR558-09|LBPV-16367
Hypostomus commersoni|FUPR875-09|LBPV-16635
Hypostomus commersoni|FUPR873-09|LBPV-16669
Hypostomus commersoni|FUPR874-09|LBPV-16671
Hypostomus commersoni|FUPR871-09|LBPV-16670
Hypostomus commersoni|FUPR870-09|LBPV-16641
Hypostomus commersoni|FUPR872-09|LBPV-16668
Hypostomus commersoni|FUPR876-09|LBPV-16636
Hypostomus commersoni|FUPR877-09|LBPV-16640
Hypostomus derbyi|FUPR865-09|LBPV-16643
Hypostomus derbyi|FUPR864-09|LBPV-16644
Pterygoplichthys anisitsi|FUPR790-09|LBPV-31755
Pterygoplichthys anisitsi|FUPR651-09|LBPV-32036
Pterygoplichthys anisitsi|FUPR652-09|LBPV-26686
Pterygoplichthys anisitsi|FUPR788-09|LBPV-26685
Pterygoplichthys anisitsi|FUPR650-09|LBPV-32035
Pterygoplichthys anisitsi|FUPR653-09|LBPV-26349
Pterygoplichthys anisitsi|FUPR649-09|LBPV-32034
Pterygoplichthys anisitsi|FUPR789-09|LBPV-31754
Megalancistrus parananus|FUPR768-09|LBPV-36609
Megalancistrus parananus|FUPR769-09|LBPV-36943
Megalancistrus parananus|FUPR767-09|LBPV-36608
Megalancistrus parananus|FUPR770-09|LBPV-36944
Ancistrus cirrhosus|FUPR782-09|LBPV-35987
Ancistrus cirrhosus|FUPR787-09|LBPV-34759
Ancistrus cirrhosus|FUPR780-09|LBPV-35959
Rineloricaria latirostris|FUPR654-09|LBPV-26332
Rineloricaria latirostris|FUPR655-09|LBPV-26333
Rineloricaria latirostris|FUPR657-09|LBPV-19534
Rineloricaria latirostris|FUPR656-09|LBPV-19533
Rineloricaria latirostris|FUPR975-09|LBPV-38088
Rineloricaria pentamaculata|FUPR1026-09|LBPV-34799b
Rineloricaria pentamaculata|FUPR658-09|LBPV-34798

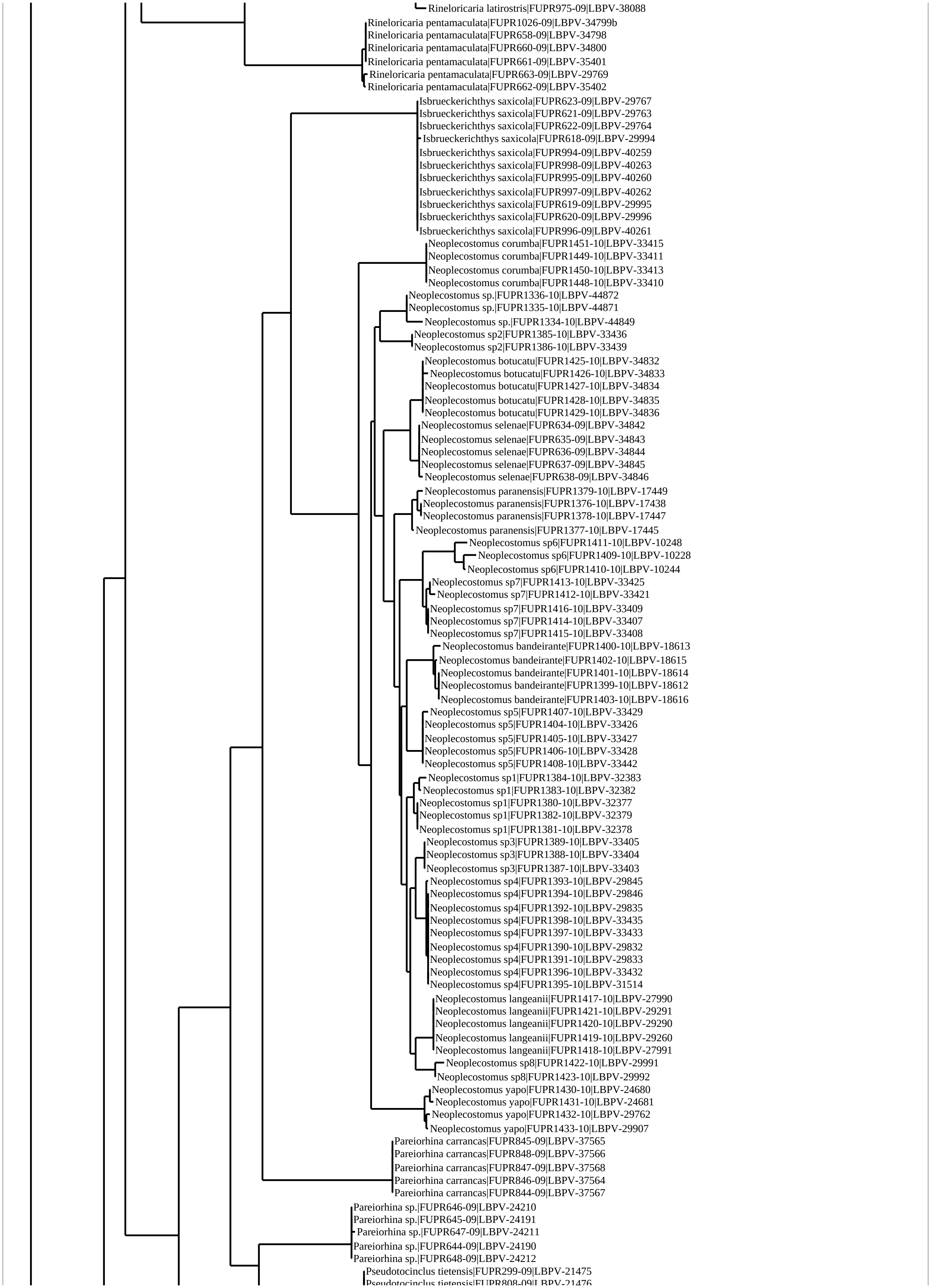
Rineloricaria latirostris|FUPR975-09|LBPV-38088
Rineloricaria pentamaculata|FUPR1026-09|LBPV-34799b
Rineloricaria pentamaculata|FUPR658-09|LBPV-34798
Rineloricaria pentamaculata|FUPR660-09|LBPV-34800
Rineloricaria pentamaculata|FUPR661-09|LBPV-35401
Rineloricaria pentamaculata|FUPR663-09|LBPV-29769
Rineloricaria pentamaculata|FUPR662-09|LBPV-35402
Isbrueckerichthys saxicola|FUPR623-09|LBPV-29767
Isbrueckerichthys saxicola|FUPR621-09|LBPV-29763
Isbrueckerichthys saxicola|FUPR622-09|LBPV-29764
Isbrueckerichthys saxicola|FUPR618-09|LBPV-29994
Isbrueckerichthys saxicola|FUPR994-09|LBPV-40259
Isbrueckerichthys saxicola|FUPR998-09|LBPV-40263
Isbrueckerichthys saxicola|FUPR995-09|LBPV-40260
Isbrueckerichthys saxicola|FUPR997-09|LBPV-40262
Isbrueckerichthys saxicola|FUPR619-09|LBPV-29995
Isbrueckerichthys saxicola|FUPR620-09|LBPV-29996
Isbrueckerichthys saxicola|FUPR996-09|LBPV-40261
Neoplecostomus corumba|FUPR1451-10|LBPV-33415
Neoplecostomus corumba|FUPR1449-10|LBPV-33411
Neoplecostomus corumba|FUPR1450-10|LBPV-33413
Neoplecostomus corumba|FUPR1448-10|LBPV-33410
Neoplecostomus sp.|FUPR1336-10|LBPV-44872
Neoplecostomus sp.|FUPR1335-10|LBPV-44871
Neoplecostomus sp.|FUPR1334-10|LBPV-44849
Neoplecostomus sp2|FUPR1385-10|LBPV-33436
Neoplecostomus sp2|FUPR1386-10|LBPV-33439
Neoplecostomus botucatu|FUPR1425-10|LBPV-34832
Neoplecostomus botucatu|FUPR1426-10|LBPV-34833
Neoplecostomus botucatu|FUPR1427-10|LBPV-34834
Neoplecostomus botucatu|FUPR1428-10|LBPV-34835
Neoplecostomus botucatu|FUPR1429-10|LBPV-34836
Neoplecostomus selenae|FUPR634-09|LBPV-34842
Neoplecostomus selenae|FUPR635-09|LBPV-34843
Neoplecostomus selenae|FUPR636-09|LBPV-34844
Neoplecostomus selenae|FUPR637-09|LBPV-34845
Neoplecostomus selenae|FUPR638-09|LBPV-34846
Neoplecostomus paranensis|FUPR1379-10|LBPV-17449
Neoplecostomus paranensis|FUPR1376-10|LBPV-17438
Neoplecostomus paranensis|FUPR1378-10|LBPV-17447
Neoplecostomus paranensis|FUPR1377-10|LBPV-17445
Neoplecostomus sp6|FUPR1411-10|LBPV-10248
Neoplecostomus sp6|FUPR1409-10|LBPV-10228
Neoplecostomus sp6|FUPR1410-10|LBPV-10244
Neoplecostomus sp7|FUPR1413-10|LBPV-33425
Neoplecostomus sp7|FUPR1412-10|LBPV-33421
Neoplecostomus sp7|FUPR1416-10|LBPV-33409
Neoplecostomus sp7|FUPR1414-10|LBPV-33407
Neoplecostomus sp7|FUPR1415-10|LBPV-33408
Neoplecostomus bandeirante|FUPR1400-10|LBPV-18613
Neoplecostomus bandeirante|FUPR1402-10|LBPV-18615
Neoplecostomus bandeirante|FUPR1401-10|LBPV-18614
Neoplecostomus bandeirante|FUPR1399-10|LBPV-18612
Neoplecostomus bandeirante|FUPR1403-10|LBPV-18616
Neoplecostomus sp5|FUPR1407-10|LBPV-33429
Neoplecostomus sp5|FUPR1404-10|LBPV-33426
Neoplecostomus sp5|FUPR1405-10|LBPV-33427
Neoplecostomus sp5|FUPR1406-10|LBPV-33428
Neoplecostomus sp5|FUPR1408-10|LBPV-33442
Neoplecostomus sp1|FUPR1384-10|LBPV-32383
Neoplecostomus sp1|FUPR1383-10|LBPV-32382
Neoplecostomus sp1|FUPR1380-10|LBPV-32377
Neoplecostomus sp1|FUPR1382-10|LBPV-32379
Neoplecostomus sp1|FUPR1381-10|LBPV-32378
Neoplecostomus sp3|FUPR1389-10|LBPV-33405
Neoplecostomus sp3|FUPR1388-10|LBPV-33404
Neoplecostomus sp3|FUPR1387-10|LBPV-33403
Neoplecostomus sp4|FUPR1393-10|LBPV-29845
Neoplecostomus sp4|FUPR1394-10|LBPV-29846
Neoplecostomus sp4|FUPR1392-10|LBPV-29835
Neoplecostomus sp4|FUPR1398-10|LBPV-33435
Neoplecostomus sp4|FUPR1397-10|LBPV-33433
Neoplecostomus sp4|FUPR1390-10|LBPV-29832
Neoplecostomus sp4|FUPR1391-10|LBPV-29833
Neoplecostomus sp4|FUPR1396-10|LBPV-33432
Neoplecostomus sp4|FUPR1395-10|LBPV-31514
Neoplecostomus langeanii|FUPR1417-10|LBPV-27990
Neoplecostomus langeanii|FUPR1421-10|LBPV-29291
Neoplecostomus langeanii|FUPR1420-10|LBPV-29290
Neoplecostomus langeanii|FUPR1419-10|LBPV-29260
Neoplecostomus langeanii|FUPR1418-10|LBPV-27991
Neoplecostomus sp8|FUPR1422-10|LBPV-29991
Neoplecostomus sp8|FUPR1423-10|LBPV-29992
Neoplecostomus yapo|FUPR1430-10|LBPV-24680
Neoplecostomus yapo|FUPR1431-10|LBPV-24681
Neoplecostomus yapo|FUPR1432-10|LBPV-29762
Neoplecostomus yapo|FUPR1433-10|LBPV-29907
Pareiorhina carrancas|FUPR845-09|LBPV-37565
Pareiorhina carrancas|FUPR848-09|LBPV-37566
Pareiorhina carrancas|FUPR847-09|LBPV-37568
Pareiorhina carrancas|FUPR846-09|LBPV-37564
Pareiorhina carrancas|FUPR844-09|LBPV-37567
Pareiorhina sp.|FUPR646-09|LBPV-24210
Pareiorhina sp.|FUPR645-09|LBPV-24191
Pareiorhina sp.|FUPR647-09|LBPV-24211
Pareiorhina sp.|FUPR644-09|LBPV-24190
Pareiorhina sp.|FUPR648-09|LBPV-24212
Pseudotocinclus tietensis|FUPR299-09|LBPV-21475
Pseudotocinclus tietensis|FUPR808-09|LBPV-21476

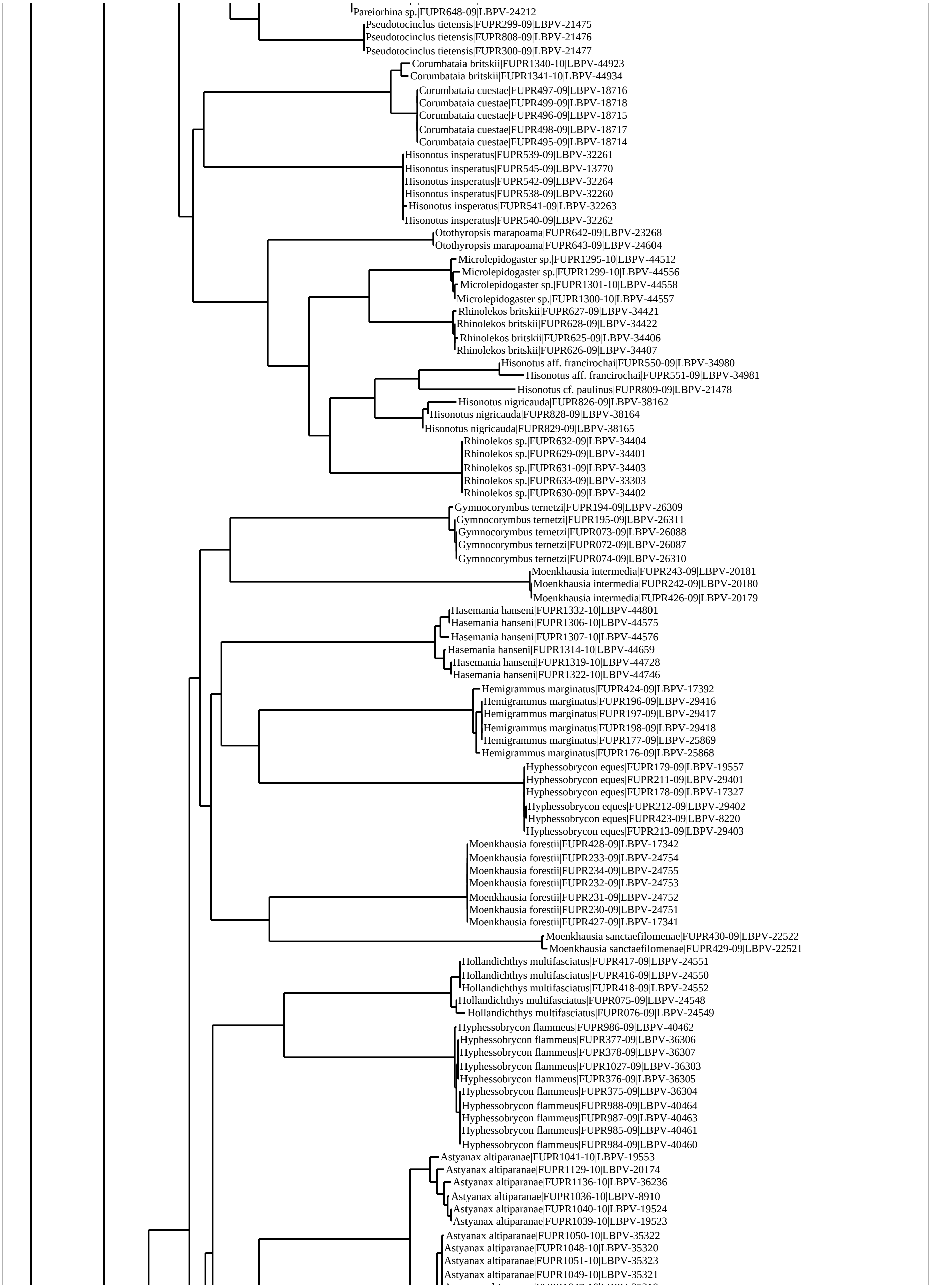
Pareiorhina sp.|FUPR648-09|LBPV-24212
Pseudotocinclus tietensis|FUPR299-09|LBPV-21475
Pseudotocinclus tietensis|FUPR808-09|LBPV-21476
Pseudotocinclus tietensis|FUPR300-09|LBPV-21477
Corumbataia britskii|FUPR1340-10|LBPV-44923
Corumbataia britskii|FUPR1341-10|LBPV-44934
Corumbataia cuestae|FUPR497-09|LBPV-18716
Corumbataia cuestae|FUPR499-09|LBPV-18718
Corumbataia cuestae|FUPR496-09|LBPV-18715
Corumbataia cuestae|FUPR498-09|LBPV-18717
Corumbataia cuestae|FUPR495-09|LBPV-18714
Hisonotus insperatus|FUPR539-09|LBPV-32261
Hisonotus insperatus|FUPR545-09|LBPV-13770
Hisonotus insperatus|FUPR542-09|LBPV-32264
Hisonotus insperatus|FUPR538-09|LBPV-32260
Hisonotus insperatus|FUPR541-09|LBPV-32263
Hisonotus insperatus|FUPR540-09|LBPV-32262
Otothyropsis marapoama|FUPR642-09|LBPV-23268
Otothyropsis marapoama|FUPR643-09|LBPV-24604
Microlepidogaster sp.|FUPR1295-10|LBPV-44512
Microlepidogaster sp.|FUPR1299-10|LBPV-44556
Microlepidogaster sp.|FUPR1301-10|LBPV-44558
Microlepidogaster sp.|FUPR1300-10|LBPV-44557
Rhinolekos britskii|FUPR627-09|LBPV-34421
Rhinolekos britskii|FUPR628-09|LBPV-34422
Rhinolekos britskii|FUPR625-09|LBPV-34406
Rhinolekos britskii|FUPR626-09|LBPV-34407
Hisonotus aff. francirochai|FUPR550-09|LBPV-34980
Hisonotus aff. francirochai|FUPR551-09|LBPV-34981
Hisonotus cf. paulinus|FUPR809-09|LBPV-21478
Hisonotus nigricauda|FUPR826-09|LBPV-38162
Hisonotus nigricauda|FUPR828-09|LBPV-38164
Hisonotus nigricauda|FUPR829-09|LBPV-38165
Rhinolekos sp.|FUPR632-09|LBPV-34404
Rhinolekos sp.|FUPR629-09|LBPV-34401
Rhinolekos sp.|FUPR631-09|LBPV-34403
Rhinolekos sp.|FUPR633-09|LBPV-33303
Rhinolekos sp.|FUPR630-09|LBPV-34402
Gymnocorymbus ternetzi|FUPR194-09|LBPV-26309
Gymnocorymbus ternetzi|FUPR195-09|LBPV-26311
Gymnocorymbus ternetzi|FUPR073-09|LBPV-26088
Gymnocorymbus ternetzi|FUPR072-09|LBPV-26087
Gymnocorymbus ternetzi|FUPR074-09|LBPV-26310
Moenkhausia intermedia|FUPR243-09|LBPV-20181
Moenkhausia intermedia|FUPR242-09|LBPV-20180
Moenkhausia intermedia|FUPR426-09|LBPV-20179
Hasemania hanseni|FUPR1332-10|LBPV-44801
Hasemania hanseni|FUPR1306-10|LBPV-44575
Hasemania hanseni|FUPR1307-10|LBPV-44576
Hasemania hanseni|FUPR1314-10|LBPV-44659
Hasemania hanseni|FUPR1319-10|LBPV-44728
Hasemania hanseni|FUPR1322-10|LBPV-44746
Hemigrammus marginatus|FUPR424-09|LBPV-17392
Hemigrammus marginatus|FUPR196-09|LBPV-29416
Hemigrammus marginatus|FUPR197-09|LBPV-29417
Hemigrammus marginatus|FUPR198-09|LBPV-29418
Hemigrammus marginatus|FUPR177-09|LBPV-25869
Hemigrammus marginatus|FUPR176-09|LBPV-25868
Hyphessobrycon eques|FUPR179-09|LBPV-19557
Hyphessobrycon eques|FUPR211-09|LBPV-29401
Hyphessobrycon eques|FUPR178-09|LBPV-17327
Hyphessobrycon eques|FUPR212-09|LBPV-29402
Hyphessobrycon eques|FUPR423-09|LBPV-8220
Hyphessobrycon eques|FUPR213-09|LBPV-29403
Moenkhausia forestii|FUPR428-09|LBPV-17342
Moenkhausia forestii|FUPR233-09|LBPV-24754
Moenkhausia forestii|FUPR234-09|LBPV-24755
Moenkhausia forestii|FUPR232-09|LBPV-24753
Moenkhausia forestii|FUPR231-09|LBPV-24752
Moenkhausia forestii|FUPR230-09|LBPV-24751
Moenkhausia forestii|FUPR427-09|LBPV-17341
Moenkhausia sanctaefilomenae|FUPR430-09|LBPV-22522
Moenkhausia sanctaefilomenae|FUPR429-09|LBPV-22521
Hollandichthys multifasciatus|FUPR417-09|LBPV-24551
Hollandichthys multifasciatus|FUPR416-09|LBPV-24550
Hollandichthys multifasciatus|FUPR418-09|LBPV-24552
Hollandichthys multifasciatus|FUPR075-09|LBPV-24548
Hollandichthys multifasciatus|FUPR076-09|LBPV-24549
Hyphessobrycon flammeus|FUPR986-09|LBPV-40462
Hyphessobrycon flammeus|FUPR377-09|LBPV-36306
Hyphessobrycon flammeus|FUPR378-09|LBPV-36307
Hyphessobrycon flammeus|FUPR1027-09|LBPV-36303
Hyphessobrycon flammeus|FUPR376-09|LBPV-36305
Hyphessobrycon flammeus|FUPR375-09|LBPV-36304
Hyphessobrycon flammeus|FUPR988-09|LBPV-40464
Hyphessobrycon flammeus|FUPR987-09|LBPV-40463
Hyphessobrycon flammeus|FUPR985-09|LBPV-40461
Hyphessobrycon flammeus|FUPR984-09|LBPV-40460
Astyanax altiparanae|FUPR1041-10|LBPV-19553
Astyanax altiparanae|FUPR1129-10|LBPV-20174
Astyanax altiparanae|FUPR1136-10|LBPV-36236
Astyanax altiparanae|FUPR1036-10|LBPV-8910
Astyanax altiparanae|FUPR1040-10|LBPV-19524
Astyanax altiparanae|FUPR1039-10|LBPV-19523
Astyanax altiparanae|FUPR1050-10|LBPV-35322
Astyanax altiparanae|FUPR1048-10|LBPV-35320
Astyanax altiparanae|FUPR1051-10|LBPV-35323
Astyanax altiparanae|FUPR1049-10|LBPV-35321

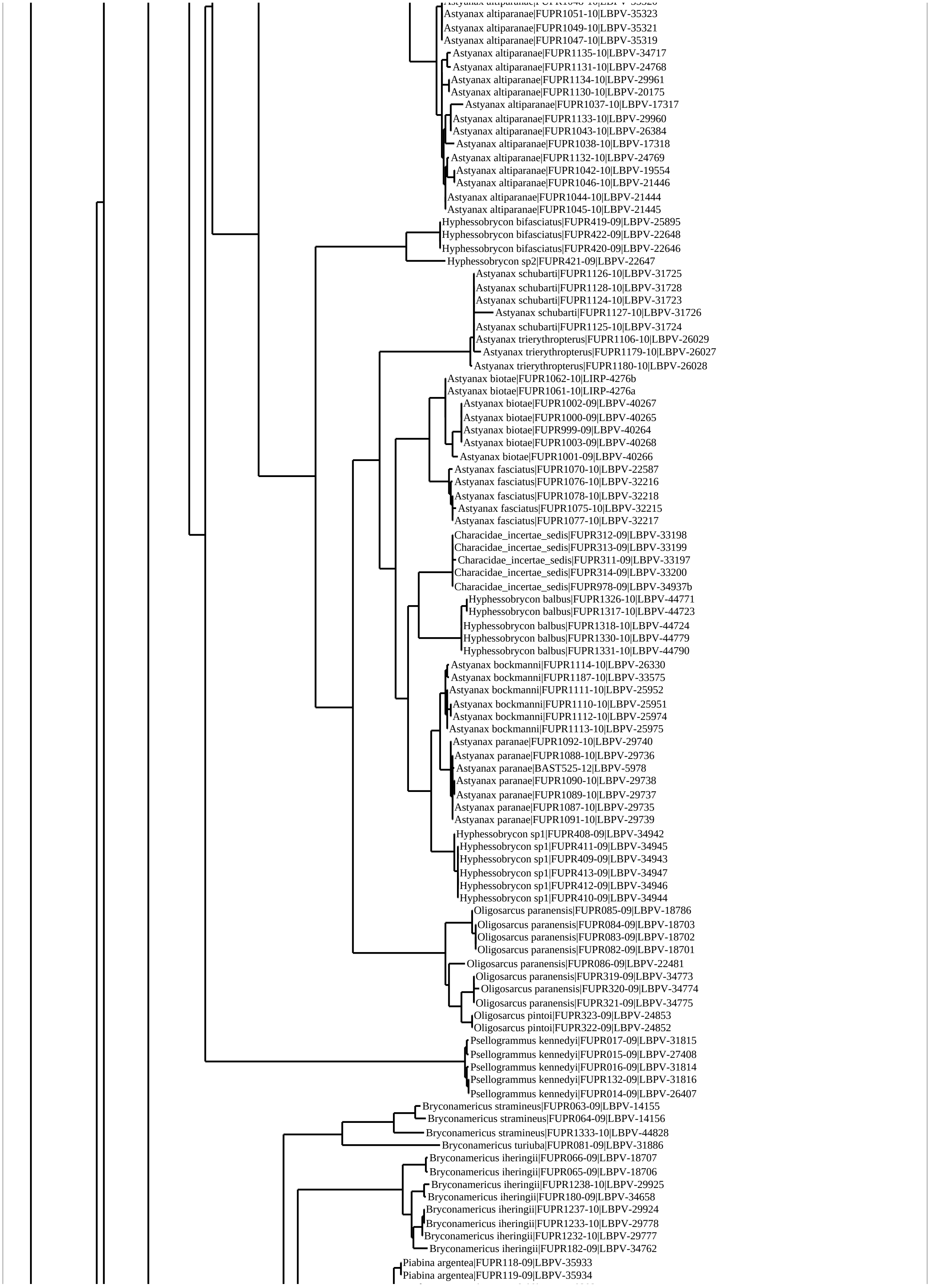
Astyanax altiparanae|FUPR1051-10|LBPV-35323
Astyanax altiparanae|FUPR1049-10|LBPV-35321
Astyanax altiparanae|FUPR1047-10|LBPV-35319
Astyanax altiparanae|FUPR1135-10|LBPV-34717
Astyanax altiparanae|FUPR1131-10|LBPV-24768
Astyanax altiparanae|FUPR1134-10|LBPV-29961
Astyanax altiparanae|FUPR1130-10|LBPV-20175
Astyanax altiparanae|FUPR1037-10|LBPV-17317
Astyanax altiparanae|FUPR1133-10|LBPV-29960
Astyanax altiparanae|FUPR1043-10|LBPV-26384
Astyanax altiparanae|FUPR1038-10|LBPV-17318
Astyanax altiparanae|FUPR1132-10|LBPV-24769
Astyanax altiparanae|FUPR1042-10|LBPV-19554
Astyanax altiparanae|FUPR1046-10|LBPV-21446
Astyanax altiparanae|FUPR1044-10|LBPV-21444
Astyanax altiparanae|FUPR1045-10|LBPV-21445
Hyphessobrycon bifasciatus|FUPR419-09|LBPV-25895
Hyphessobrycon bifasciatus|FUPR422-09|LBPV-22648
Hyphessobrycon bifasciatus|FUPR420-09|LBPV-22646
Hyphessobrycon sp2|FUPR421-09|LBPV-22647
Astyanax schubarti|FUPR1126-10|LBPV-31725
Astyanax schubarti|FUPR1128-10|LBPV-31728
Astyanax schubarti|FUPR1124-10|LBPV-31723
Astyanax schubarti|FUPR1127-10|LBPV-31726
Astyanax schubarti|FUPR1125-10|LBPV-31724
Astyanax trierythropterus|FUPR1106-10|LBPV-26029
Astyanax trierythropterus|FUPR1179-10|LBPV-26027
Astyanax trierythropterus|FUPR1180-10|LBPV-26028
Astyanax biotae|FUPR1062-10|LIRP-4276b
Astyanax biotae|FUPR1061-10|LIRP-4276a
Astyanax biotae|FUPR1002-09|LBPV-40267
Astyanax biotae|FUPR1000-09|LBPV-40265
Astyanax biotae|FUPR999-09|LBPV-40264
Astyanax biotae|FUPR1003-09|LBPV-40268
Astyanax biotae|FUPR1001-09|LBPV-40266
Astyanax fasciatus|FUPR1070-10|LBPV-22587
Astyanax fasciatus|FUPR1076-10|LBPV-32216
Astyanax fasciatus|FUPR1078-10|LBPV-32218
Astyanax fasciatus|FUPR1075-10|LBPV-32215
Astyanax fasciatus|FUPR1077-10|LBPV-32217
Characidae_incertae_sedis|FUPR312-09|LBPV-33198
Characidae_incertae_sedis|FUPR313-09|LBPV-33199
Characidae_incertae_sedis|FUPR311-09|LBPV-33197
Characidae_incertae_sedis|FUPR314-09|LBPV-33200
Characidae_incertae_sedis|FUPR978-09|LBPV-34937b
Hyphessobrycon balbus|FUPR1326-10|LBPV-44771
Hyphessobrycon balbus|FUPR1317-10|LBPV-44723
Hyphessobrycon balbus|FUPR1318-10|LBPV-44724
Hyphessobrycon balbus|FUPR1330-10|LBPV-44779
Hyphessobrycon balbus|FUPR1331-10|LBPV-44790
Astyanax bockmanni|FUPR1114-10|LBPV-26330
Astyanax bockmanni|FUPR1187-10|LBPV-33575
Astyanax bockmanni|FUPR1111-10|LBPV-25952
Astyanax bockmanni|FUPR1110-10|LBPV-25951
Astyanax bockmanni|FUPR1112-10|LBPV-25974
Astyanax bockmanni|FUPR1113-10|LBPV-25975
Astyanax paranae|FUPR1092-10|LBPV-29740
Astyanax paranae|FUPR1088-10|LBPV-29736
Astyanax paranae|BAST525-12|LBPV-5978
Astyanax paranae|FUPR1090-10|LBPV-29738
Astyanax paranae|FUPR1089-10|LBPV-29737
Astyanax paranae|FUPR1087-10|LBPV-29735
Astyanax paranae|FUPR1091-10|LBPV-29739
Hyphessobrycon sp1|FUPR408-09|LBPV-34942
Hyphessobrycon sp1|FUPR411-09|LBPV-34945
Hyphessobrycon sp1|FUPR409-09|LBPV-34943
Hyphessobrycon sp1|FUPR413-09|LBPV-34947
Hyphessobrycon sp1|FUPR412-09|LBPV-34946
Hyphessobrycon sp1|FUPR410-09|LBPV-34944
Oligosarcus paranensis|FUPR085-09|LBPV-18786
Oligosarcus paranensis|FUPR084-09|LBPV-18703
Oligosarcus paranensis|FUPR083-09|LBPV-18702
Oligosarcus paranensis|FUPR082-09|LBPV-18701
Oligosarcus paranensis|FUPR086-09|LBPV-22481
Oligosarcus paranensis|FUPR319-09|LBPV-34773
Oligosarcus paranensis|FUPR320-09|LBPV-34774
Oligosarcus paranensis|FUPR321-09|LBPV-34775
Oligosarcus pintoi|FUPR323-09|LBPV-24853
Oligosarcus pintoi|FUPR322-09|LBPV-24852
Psellogrammus kennedyi|FUPR017-09|LBPV-31815
Psellogrammus kennedyi|FUPR015-09|LBPV-27408
Psellogrammus kennedyi|FUPR016-09|LBPV-31814
Psellogrammus kennedyi|FUPR132-09|LBPV-31816
Psellogrammus kennedyi|FUPR014-09|LBPV-26407
Bryconamericus stramineus|FUPR063-09|LBPV-14155
Bryconamericus stramineus|FUPR064-09|LBPV-14156
Bryconamericus stramineus|FUPR1333-10|LBPV-44828
Bryconamericus turiuba|FUPR081-09|LBPV-31886
Bryconamericus iheringii|FUPR066-09|LBPV-18707
Bryconamericus iheringii|FUPR065-09|LBPV-18706
Bryconamericus iheringii|FUPR1238-10|LBPV-29925
Bryconamericus iheringii|FUPR180-09|LBPV-34658
Bryconamericus iheringii|FUPR1237-10|LBPV-29924
Bryconamericus iheringii|FUPR1233-10|LBPV-29778
Bryconamericus iheringii|FUPR1232-10|LBPV-29777
Bryconamericus iheringii|FUPR182-09|LBPV-34762
Piabina argentea|FUPR118-09|LBPV-35933
Piabina argentea|FUPR119-09|LBPV-35934

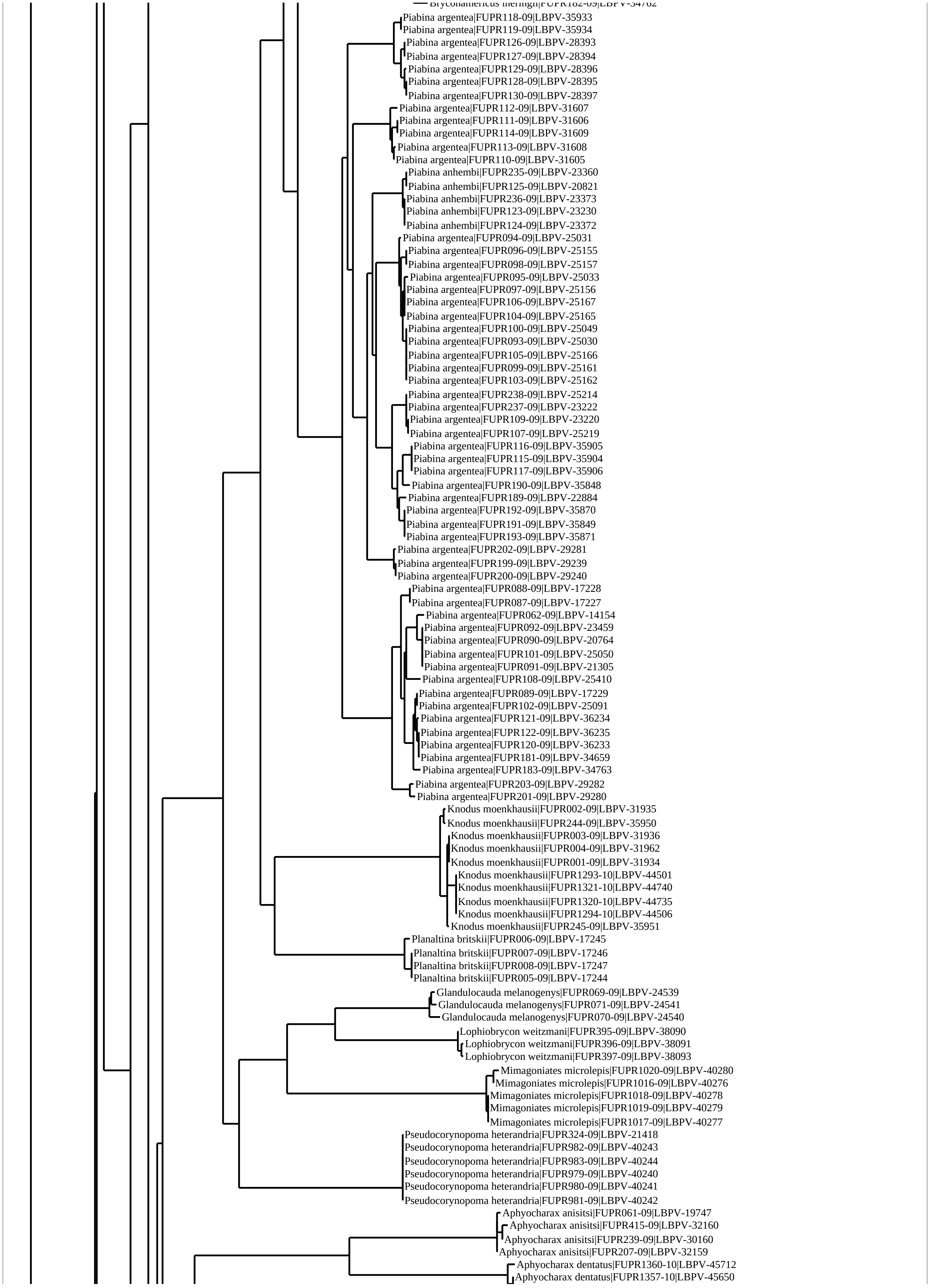
Piabina argentea|FUPR118-09|LBPV-35933
Piabina argentea|FUPR119-09|LBPV-35934
Piabina argentea|FUPR126-09|LBPV-28393
Piabina argentea|FUPR127-09|LBPV-28394
Piabina argentea|FUPR129-09|LBPV-28396
Piabina argentea|FUPR128-09|LBPV-28395
Piabina argentea|FUPR130-09|LBPV-28397
Piabina argentea|FUPR112-09|LBPV-31607
Piabina argentea|FUPR111-09|LBPV-31606
Piabina argentea|FUPR114-09|LBPV-31609
Piabina argentea|FUPR113-09|LBPV-31608
Piabina argentea|FUPR110-09|LBPV-31605
Piabina anhembi|FUPR235-09|LBPV-23360
Piabina anhembi|FUPR125-09|LBPV-20821
Piabina anhembi|FUPR236-09|LBPV-23373
Piabina anhembi|FUPR123-09|LBPV-23230
Piabina anhembi|FUPR124-09|LBPV-23372
Piabina argentea|FUPR094-09|LBPV-25031
Piabina argentea|FUPR096-09|LBPV-25155
Piabina argentea|FUPR098-09|LBPV-25157
Piabina argentea|FUPR095-09|LBPV-25033
Piabina argentea|FUPR097-09|LBPV-25156
Piabina argentea|FUPR106-09|LBPV-25167
Piabina argentea|FUPR104-09|LBPV-25165
Piabina argentea|FUPR100-09|LBPV-25049
Piabina argentea|FUPR093-09|LBPV-25030
Piabina argentea|FUPR105-09|LBPV-25166
Piabina argentea|FUPR099-09|LBPV-25161
Piabina argentea|FUPR103-09|LBPV-25162
Piabina argentea|FUPR238-09|LBPV-25214
Piabina argentea|FUPR237-09|LBPV-23222
Piabina argentea|FUPR109-09|LBPV-23220
Piabina argentea|FUPR107-09|LBPV-25219
Piabina argentea|FUPR116-09|LBPV-35905
Piabina argentea|FUPR115-09|LBPV-35904
Piabina argentea|FUPR117-09|LBPV-35906
Piabina argentea|FUPR190-09|LBPV-35848
Piabina argentea|FUPR189-09|LBPV-22884
Piabina argentea|FUPR192-09|LBPV-35870
Piabina argentea|FUPR191-09|LBPV-35849
Piabina argentea|FUPR193-09|LBPV-35871
Piabina argentea|FUPR202-09|LBPV-29281
Piabina argentea|FUPR199-09|LBPV-29239
Piabina argentea|FUPR200-09|LBPV-29240
Piabina argentea|FUPR088-09|LBPV-17228
Piabina argentea|FUPR087-09|LBPV-17227
Piabina argentea|FUPR062-09|LBPV-14154
Piabina argentea|FUPR092-09|LBPV-23459
Piabina argentea|FUPR090-09|LBPV-20764
Piabina argentea|FUPR101-09|LBPV-25050
Piabina argentea|FUPR091-09|LBPV-21305
Piabina argentea|FUPR108-09|LBPV-25410
Piabina argentea|FUPR089-09|LBPV-17229
Piabina argentea|FUPR102-09|LBPV-25091
Piabina argentea|FUPR121-09|LBPV-36234
Piabina argentea|FUPR122-09|LBPV-36235
Piabina argentea|FUPR120-09|LBPV-36233
Piabina argentea|FUPR181-09|LBPV-34659
Piabina argentea|FUPR183-09|LBPV-34763
Piabina argentea|FUPR203-09|LBPV-29282
Piabina argentea|FUPR201-09|LBPV-29280
Knodus moenkhausii|FUPR002-09|LBPV-31935
Knodus moenkhausii|FUPR244-09|LBPV-35950
Knodus moenkhausii|FUPR003-09|LBPV-31936
Knodus moenkhausii|FUPR004-09|LBPV-31962
Knodus moenkhausii|FUPR001-09|LBPV-31934
Knodus moenkhausii|FUPR1293-10|LBPV-44501
Knodus moenkhausii|FUPR1321-10|LBPV-44740
Knodus moenkhausii|FUPR1320-10|LBPV-44735
Knodus moenkhausii|FUPR1294-10|LBPV-44506
Knodus moenkhausii|FUPR245-09|LBPV-35951
Planaltina britskii|FUPR006-09|LBPV-17245
Planaltina britskii|FUPR007-09|LBPV-17246
Planaltina britskii|FUPR008-09|LBPV-17247
Planaltina britskii|FUPR005-09|LBPV-17244
Glandulocauda melanogenys|FUPR069-09|LBPV-24539
Glandulocauda melanogenys|FUPR071-09|LBPV-24541
Glandulocauda melanogenys|FUPR070-09|LBPV-24540
Lophiobrycon weitzmani|FUPR395-09|LBPV-38090
Lophiobrycon weitzmani|FUPR396-09|LBPV-38091
Lophiobrycon weitzmani|FUPR397-09|LBPV-38093
Mimagoniates microlepis|FUPR1020-09|LBPV-40280
Mimagoniates microlepis|FUPR1016-09|LBPV-40276
Mimagoniates microlepis|FUPR1018-09|LBPV-40278
Mimagoniates microlepis|FUPR1019-09|LBPV-40279
Mimagoniates microlepis|FUPR1017-09|LBPV-40277
Pseudocorynopoma heterandria|FUPR324-09|LBPV-21418
Pseudocorynopoma heterandria|FUPR982-09|LBPV-40243
Pseudocorynopoma heterandria|FUPR983-09|LBPV-40244
Pseudocorynopoma heterandria|FUPR979-09|LBPV-40240
Pseudocorynopoma heterandria|FUPR980-09|LBPV-40241
Pseudocorynopoma heterandria|FUPR981-09|LBPV-40242
Aphyocharax anisitsi|FUPR061-09|LBPV-19747
Aphyocharax anisitsi|FUPR415-09|LBPV-32160
Aphyocharax anisitsi|FUPR239-09|LBPV-30160
Aphyocharax anisitsi|FUPR207-09|LBPV-32159
Aphyocharax dentatus|FUPR1360-10|LBPV-45712
Aphyocharax dentatus|FUPR1357-10|LBPV-45650

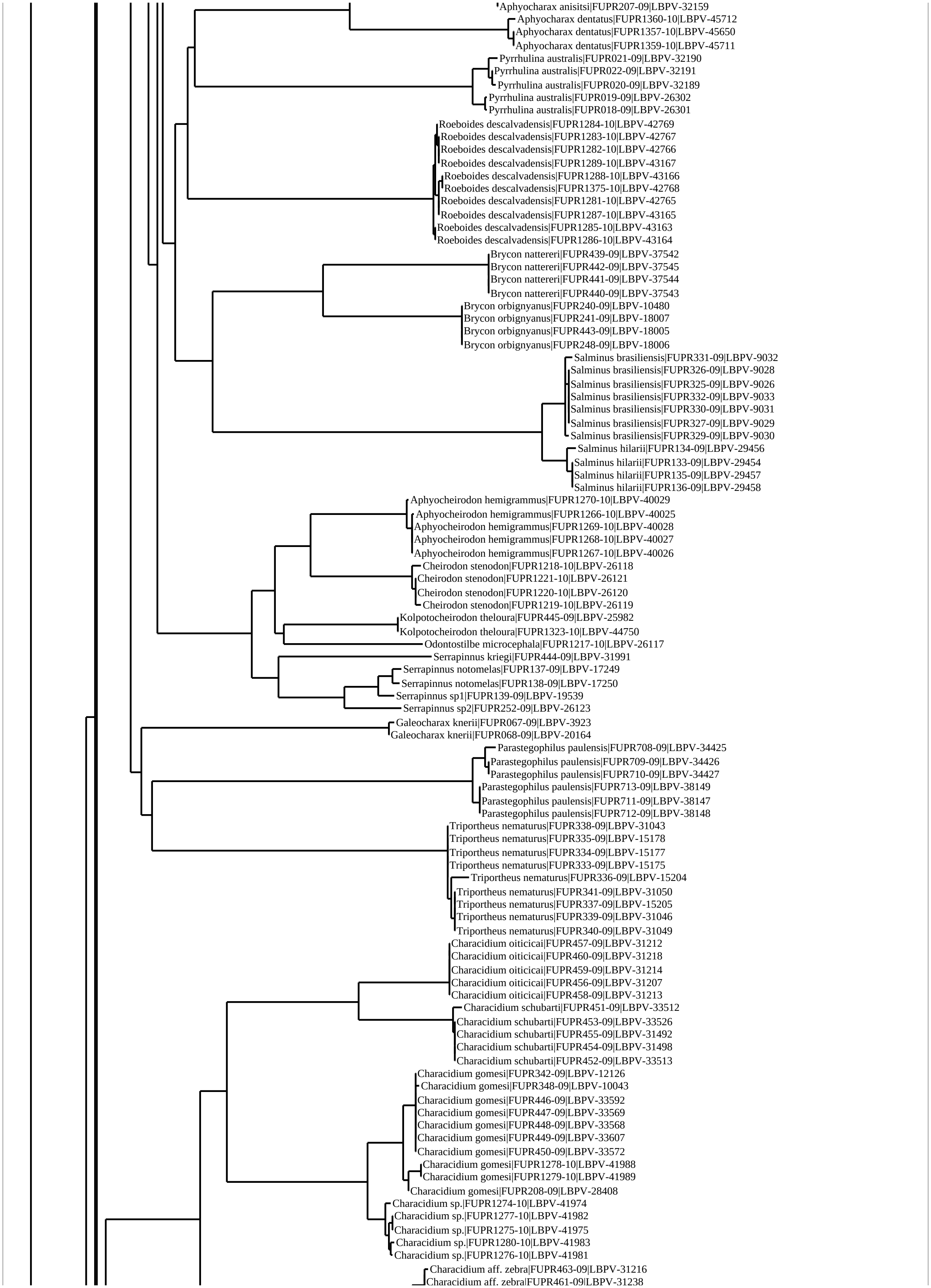
Aphyocharax anisitsi|FUPR207-09|LBPV-32159
Aphyocharax dentatus|FUPR1360-10|LBPV-45712
Aphyocharax dentatus|FUPR1357-10|LBPV-45650
Aphyocharax dentatus|FUPR1359-10|LBPV-45711
Pyrrhulina australis|FUPR021-09|LBPV-32190
Pyrrhulina australis|FUPR022-09|LBPV-32191
Pyrrhulina australis|FUPR020-09|LBPV-32189
Pyrrhulina australis|FUPR019-09|LBPV-26302
Pyrrhulina australis|FUPR018-09|LBPV-26301
Roeboides descalvadensis|FUPR1284-10|LBPV-42769
Roeboides descalvadensis|FUPR1283-10|LBPV-42767
Roeboides descalvadensis|FUPR1282-10|LBPV-42766
Roeboides descalvadensis|FUPR1289-10|LBPV-43167
Roeboides descalvadensis|FUPR1288-10|LBPV-43166
Roeboides descalvadensis|FUPR1375-10|LBPV-42768
Roeboides descalvadensis|FUPR1281-10|LBPV-42765
Roeboides descalvadensis|FUPR1287-10|LBPV-43165
Roeboides descalvadensis|FUPR1285-10|LBPV-43163
Roeboides descalvadensis|FUPR1286-10|LBPV-43164
Brycon nattereri|FUPR439-09|LBPV-37542
Brycon nattereri|FUPR442-09|LBPV-37545
Brycon nattereri|FUPR441-09|LBPV-37544
Brycon nattereri|FUPR440-09|LBPV-37543
Brycon orbignyanus|FUPR240-09|LBPV-10480
Brycon orbignyanus|FUPR241-09|LBPV-18007
Brycon orbignyanus|FUPR443-09|LBPV-18005
Brycon orbignyanus|FUPR248-09|LBPV-18006
Salminus brasiliensis|FUPR331-09|LBPV-9032
Salminus brasiliensis|FUPR326-09|LBPV-9028
Salminus brasiliensis|FUPR325-09|LBPV-9026
Salminus brasiliensis|FUPR332-09|LBPV-9033
Salminus brasiliensis|FUPR330-09|LBPV-9031
Salminus brasiliensis|FUPR327-09|LBPV-9029
Salminus brasiliensis|FUPR329-09|LBPV-9030
Salminus hilarii|FUPR134-09|LBPV-29456
Salminus hilarii|FUPR133-09|LBPV-29454
Salminus hilarii|FUPR135-09|LBPV-29457
Salminus hilarii|FUPR136-09|LBPV-29458
Aphyocheirodon hemigrammus|FUPR1270-10|LBPV-40029
Aphyocheirodon hemigrammus|FUPR1266-10|LBPV-40025
Aphyocheirodon hemigrammus|FUPR1269-10|LBPV-40028
Aphyocheirodon hemigrammus|FUPR1268-10|LBPV-40027
Aphyocheirodon hemigrammus|FUPR1267-10|LBPV-40026
Cheirodon stenodon|FUPR1218-10|LBPV-26118
Cheirodon stenodon|FUPR1221-10|LBPV-26121
Cheirodon stenodon|FUPR1220-10|LBPV-26120
Cheirodon stenodon|FUPR1219-10|LBPV-26119
Kolpotocheirodon theloura|FUPR445-09|LBPV-25982
Kolpotocheirodon theloura|FUPR1323-10|LBPV-44750
Odontostilbe microcephala|FUPR1217-10|LBPV-26117
Serrapinnus kriegi|FUPR444-09|LBPV-31991
Serrapinnus notomelas|FUPR137-09|LBPV-17249
Serrapinnus notomelas|FUPR138-09|LBPV-17250
Serrapinnus sp1|FUPR139-09|LBPV-19539
Serrapinnus sp2|FUPR252-09|LBPV-26123
Galeocharax knerii|FUPR067-09|LBPV-3923
Galeocharax knerii|FUPR068-09|LBPV-20164
Parastegophilus paulensis|FUPR708-09|LBPV-34425
Parastegophilus paulensis|FUPR709-09|LBPV-34426
Parastegophilus paulensis|FUPR710-09|LBPV-34427
Parastegophilus paulensis|FUPR713-09|LBPV-38149
Parastegophilus paulensis|FUPR711-09|LBPV-38147
Parastegophilus paulensis|FUPR712-09|LBPV-38148
Triportheus nematurus|FUPR338-09|LBPV-31043
Triportheus nematurus|FUPR335-09|LBPV-15178
Triportheus nematurus|FUPR334-09|LBPV-15177
Triportheus nematurus|FUPR333-09|LBPV-15175
Triportheus nematurus|FUPR336-09|LBPV-15204
Triportheus nematurus|FUPR341-09|LBPV-31050
Triportheus nematurus|FUPR337-09|LBPV-15205
Triportheus nematurus|FUPR339-09|LBPV-31046
Triportheus nematurus|FUPR340-09|LBPV-31049
Characidium oiticicai|FUPR457-09|LBPV-31212
Characidium oiticicai|FUPR460-09|LBPV-31218
Characidium oiticicai|FUPR459-09|LBPV-31214
Characidium oiticicai|FUPR456-09|LBPV-31207
Characidium oiticicai|FUPR458-09|LBPV-31213
Characidium schubarti|FUPR451-09|LBPV-33512
Characidium schubarti|FUPR453-09|LBPV-33526
Characidium schubarti|FUPR455-09|LBPV-31492
Characidium schubarti|FUPR454-09|LBPV-31498
Characidium schubarti|FUPR452-09|LBPV-33513
Characidium gomesi|FUPR342-09|LBPV-12126
Characidium gomesi|FUPR348-09|LBPV-10043
Characidium gomesi|FUPR446-09|LBPV-33592
Characidium gomesi|FUPR447-09|LBPV-33569
Characidium gomesi|FUPR448-09|LBPV-33568
Characidium gomesi|FUPR449-09|LBPV-33607
Characidium gomesi|FUPR450-09|LBPV-33572
Characidium gomesi|FUPR1278-10|LBPV-41988
Characidium gomesi|FUPR1279-10|LBPV-41989
Characidium gomesi|FUPR208-09|LBPV-28408
Characidium sp.|FUPR1274-10|LBPV-41974
Characidium sp.|FUPR1277-10|LBPV-41982
Characidium sp.|FUPR1275-10|LBPV-41975
Characidium sp.|FUPR1280-10|LBPV-41983
Characidium sp.|FUPR1276-10|LBPV-41981
Characidium aff. zebra|FUPR463-09|LBPV-31216
Characidium aff. zebra|FUPR461-09|LBPV-31238

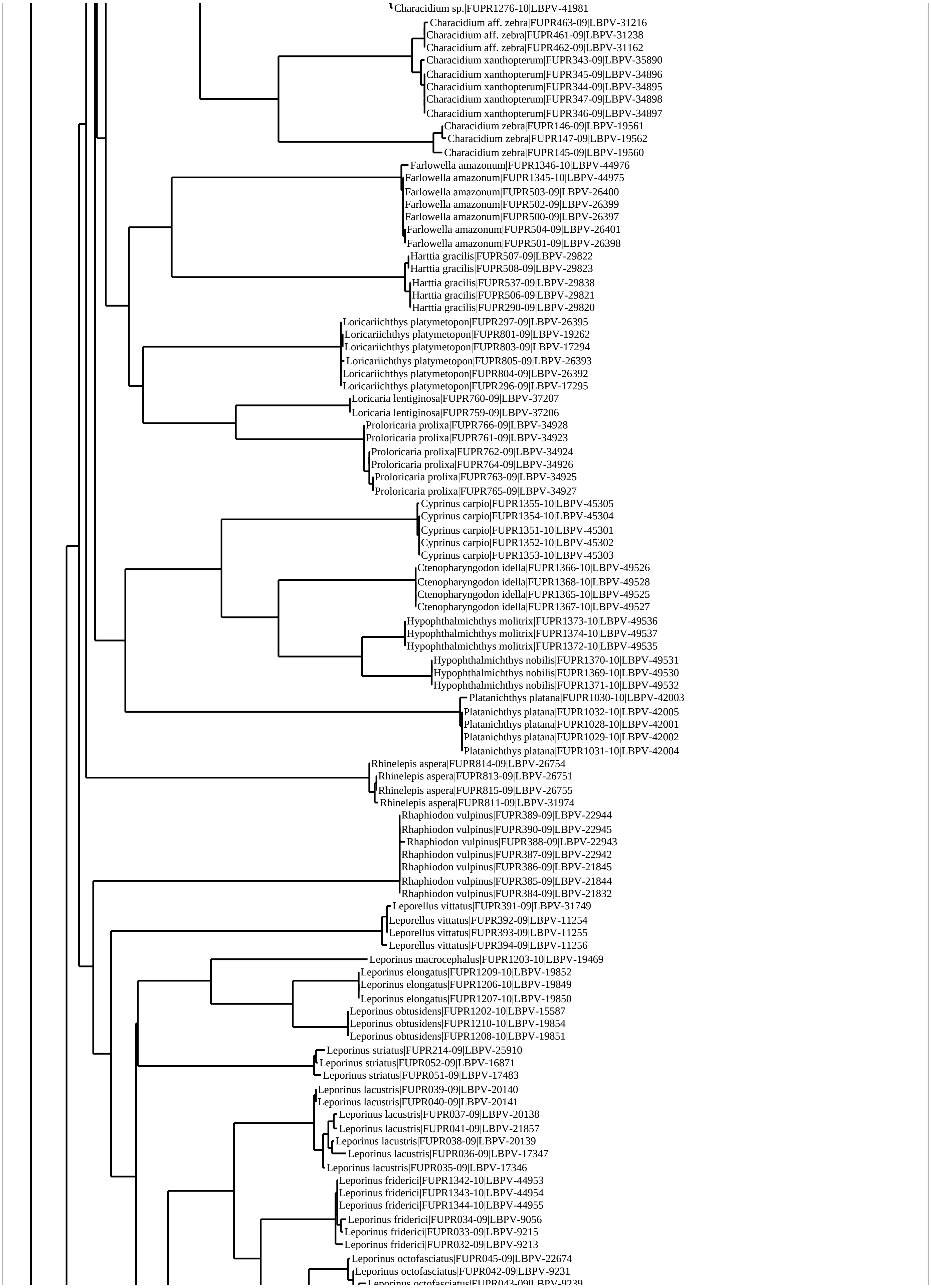
Characidium sp.|FUPR1276-10|LBPV-41981
Characidium aff. zebra|FUPR463-09|LBPV-31216
Characidium aff. zebra|FUPR461-09|LBPV-31238
Characidium aff. zebra|FUPR462-09|LBPV-31162
Characidium xanthopterum|FUPR343-09|LBPV-35890
Characidium xanthopterum|FUPR345-09|LBPV-34896
Characidium xanthopterum|FUPR344-09|LBPV-34895
Characidium xanthopterum|FUPR347-09|LBPV-34898
Characidium xanthopterum|FUPR346-09|LBPV-34897
Characidium zebra|FUPR146-09|LBPV-19561
Characidium zebra|FUPR147-09|LBPV-19562
Characidium zebra|FUPR145-09|LBPV-19560
Farlowella amazonum|FUPR1346-10|LBPV-44976
Farlowella amazonum|FUPR1345-10|LBPV-44975
Farlowella amazonum|FUPR503-09|LBPV-26400
Farlowella amazonum|FUPR502-09|LBPV-26399
Farlowella amazonum|FUPR500-09|LBPV-26397
Farlowella amazonum|FUPR504-09|LBPV-26401
Farlowella amazonum|FUPR501-09|LBPV-26398
Harttia gracilis|FUPR507-09|LBPV-29822
Harttia gracilis|FUPR508-09|LBPV-29823
Harttia gracilis|FUPR537-09|LBPV-29838
Harttia gracilis|FUPR506-09|LBPV-29821
Harttia gracilis|FUPR290-09|LBPV-29820
Loricariichthys platymetopon|FUPR297-09|LBPV-26395
Loricariichthys platymetopon|FUPR801-09|LBPV-19262
Loricariichthys platymetopon|FUPR803-09|LBPV-17294
Loricariichthys platymetopon|FUPR805-09|LBPV-26393
Loricariichthys platymetopon|FUPR804-09|LBPV-26392
Loricariichthys platymetopon|FUPR296-09|LBPV-17295
Loricaria lentiginosa|FUPR760-09|LBPV-37207
Loricaria lentiginosa|FUPR759-09|LBPV-37206
Proloricaria prolixa|FUPR766-09|LBPV-34928
Proloricaria prolixa|FUPR761-09|LBPV-34923
Proloricaria prolixa|FUPR762-09|LBPV-34924
Proloricaria prolixa|FUPR764-09|LBPV-34926
Proloricaria prolixa|FUPR763-09|LBPV-34925
Proloricaria prolixa|FUPR765-09|LBPV-34927
Cyprinus carpio|FUPR1355-10|LBPV-45305
Cyprinus carpio|FUPR1354-10|LBPV-45304
Cyprinus carpio|FUPR1351-10|LBPV-45301
Cyprinus carpio|FUPR1352-10|LBPV-45302
Cyprinus carpio|FUPR1353-10|LBPV-45303
Ctenopharyngodon idella|FUPR1366-10|LBPV-49526
Ctenopharyngodon idella|FUPR1368-10|LBPV-49528
Ctenopharyngodon idella|FUPR1365-10|LBPV-49525
Ctenopharyngodon idella|FUPR1367-10|LBPV-49527
Hypophthalmichthys molitrix|FUPR1373-10|LBPV-49536
Hypophthalmichthys molitrix|FUPR1374-10|LBPV-49537
Hypophthalmichthys molitrix|FUPR1372-10|LBPV-49535
Hypophthalmichthys nobilis|FUPR1370-10|LBPV-49531
Hypophthalmichthys nobilis|FUPR1369-10|LBPV-49530
Hypophthalmichthys nobilis|FUPR1371-10|LBPV-49532
Platanichthys platana|FUPR1030-10|LBPV-42003
Platanichthys platana|FUPR1032-10|LBPV-42005
Platanichthys platana|FUPR1028-10|LBPV-42001
Platanichthys platana|FUPR1029-10|LBPV-42002
Platanichthys platana|FUPR1031-10|LBPV-42004
Rhinelepis aspera|FUPR814-09|LBPV-26754
Rhinelepis aspera|FUPR813-09|LBPV-26751
Rhinelepis aspera|FUPR815-09|LBPV-26755
Rhinelepis aspera|FUPR811-09|LBPV-31974
Rhaphiodon vulpinus|FUPR389-09|LBPV-22944
Rhaphiodon vulpinus|FUPR390-09|LBPV-22945
Rhaphiodon vulpinus|FUPR388-09|LBPV-22943
Rhaphiodon vulpinus|FUPR387-09|LBPV-22942
Rhaphiodon vulpinus|FUPR386-09|LBPV-21845
Rhaphiodon vulpinus|FUPR385-09|LBPV-21844
Rhaphiodon vulpinus|FUPR384-09|LBPV-21832
Leporellus vittatus|FUPR391-09|LBPV-31749
Leporellus vittatus|FUPR392-09|LBPV-11254
Leporellus vittatus|FUPR393-09|LBPV-11255
Leporellus vittatus|FUPR394-09|LBPV-11256
Leporinus macrocephalus|FUPR1203-10|LBPV-19469
Leporinus elongatus|FUPR1209-10|LBPV-19852
Leporinus elongatus|FUPR1206-10|LBPV-19849
Leporinus elongatus|FUPR1207-10|LBPV-19850
Leporinus obtusidens|FUPR1202-10|LBPV-15587
Leporinus obtusidens|FUPR1210-10|LBPV-19854
Leporinus obtusidens|FUPR1208-10|LBPV-19851
Leporinus striatus|FUPR214-09|LBPV-25910
Leporinus striatus|FUPR052-09|LBPV-16871
Leporinus striatus|FUPR051-09|LBPV-17483
Leporinus lacustris|FUPR039-09|LBPV-20140
Leporinus lacustris|FUPR040-09|LBPV-20141
Leporinus lacustris|FUPR037-09|LBPV-20138
Leporinus lacustris|FUPR041-09|LBPV-21857
Leporinus lacustris|FUPR038-09|LBPV-20139
Leporinus lacustris|FUPR036-09|LBPV-17347
Leporinus lacustris|FUPR035-09|LBPV-17346
Leporinus friderici|FUPR1342-10|LBPV-44953
Leporinus friderici|FUPR1343-10|LBPV-44954
Leporinus friderici|FUPR1344-10|LBPV-44955
Leporinus friderici|FUPR034-09|LBPV-9056
Leporinus friderici|FUPR033-09|LBPV-9215
Leporinus friderici|FUPR032-09|LBPV-9213
Leporinus octofasciatus|FUPR045-09|LBPV-22674
Leporinus octofasciatus|FUPR042-09|LBPV-9231
Leporinus octofasciatus|FUPR043-09|LBPV-9239

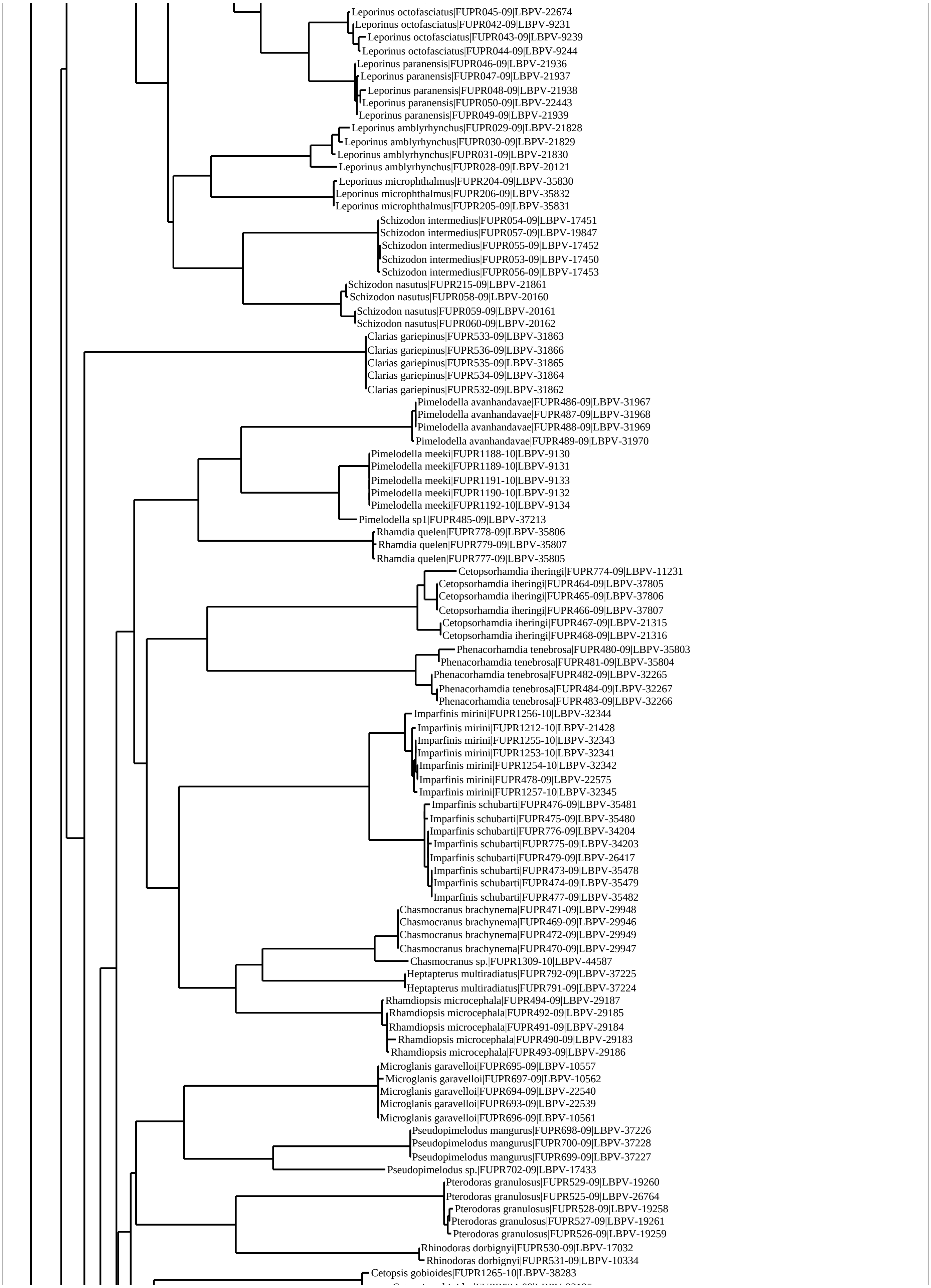
Leporinus octofasciatus|FUPR045-09|LBPV-22674
Leporinus octofasciatus|FUPR042-09|LBPV-9231
Leporinus octofasciatus|FUPR043-09|LBPV-9239
Leporinus octofasciatus|FUPR044-09|LBPV-9244
Leporinus paranensis|FUPR046-09|LBPV-21936
Leporinus paranensis|FUPR047-09|LBPV-21937
Leporinus paranensis|FUPR048-09|LBPV-21938
Leporinus paranensis|FUPR050-09|LBPV-22443
Leporinus paranensis|FUPR049-09|LBPV-21939
Leporinus amblyrhynchus|FUPR029-09|LBPV-21828
Leporinus amblyrhynchus|FUPR030-09|LBPV-21829
Leporinus amblyrhynchus|FUPR031-09|LBPV-21830
Leporinus amblyrhynchus|FUPR028-09|LBPV-20121
Leporinus microphthalmus|FUPR204-09|LBPV-35830
Leporinus microphthalmus|FUPR206-09|LBPV-35832
Leporinus microphthalmus|FUPR205-09|LBPV-35831
Schizodon intermedius|FUPR054-09|LBPV-17451
Schizodon intermedius|FUPR057-09|LBPV-19847
Schizodon intermedius|FUPR055-09|LBPV-17452
Schizodon intermedius|FUPR053-09|LBPV-17450
Schizodon intermedius|FUPR056-09|LBPV-17453
Schizodon nasutus|FUPR215-09|LBPV-21861
Schizodon nasutus|FUPR058-09|LBPV-20160
Schizodon nasutus|FUPR059-09|LBPV-20161
Schizodon nasutus|FUPR060-09|LBPV-20162
Clarias gariepinus|FUPR533-09|LBPV-31863
Clarias gariepinus|FUPR536-09|LBPV-31866
Clarias gariepinus|FUPR535-09|LBPV-31865
Clarias gariepinus|FUPR534-09|LBPV-31864
Clarias gariepinus|FUPR532-09|LBPV-31862
Pimelodella avanhandavae|FUPR486-09|LBPV-31967
Pimelodella avanhandavae|FUPR487-09|LBPV-31968
Pimelodella avanhandavae|FUPR488-09|LBPV-31969
Pimelodella avanhandavae|FUPR489-09|LBPV-31970
Pimelodella meeki|FUPR1188-10|LBPV-9130
Pimelodella meeki|FUPR1189-10|LBPV-9131
Pimelodella meeki|FUPR1191-10|LBPV-9133
Pimelodella meeki|FUPR1190-10|LBPV-9132
Pimelodella meeki|FUPR1192-10|LBPV-9134
Pimelodella sp1|FUPR485-09|LBPV-37213
Rhamdia quelen|FUPR778-09|LBPV-35806
Rhamdia quelen|FUPR779-09|LBPV-35807
Rhamdia quelen|FUPR777-09|LBPV-35805
Cetopsorhamdia iheringi|FUPR774-09|LBPV-11231
Cetopsorhamdia iheringi|FUPR464-09|LBPV-37805
Cetopsorhamdia iheringi|FUPR465-09|LBPV-37806
Cetopsorhamdia iheringi|FUPR466-09|LBPV-37807
Cetopsorhamdia iheringi|FUPR467-09|LBPV-21315
Cetopsorhamdia iheringi|FUPR468-09|LBPV-21316
Phenacorhamdia tenebrosa|FUPR480-09|LBPV-35803
Phenacorhamdia tenebrosa|FUPR481-09|LBPV-35804
Phenacorhamdia tenebrosa|FUPR482-09|LBPV-32265
Phenacorhamdia tenebrosa|FUPR484-09|LBPV-32267
Phenacorhamdia tenebrosa|FUPR483-09|LBPV-32266
Imparfinis mirini|FUPR1256-10|LBPV-32344
Imparfinis mirini|FUPR1212-10|LBPV-21428
Imparfinis mirini|FUPR1255-10|LBPV-32343
Imparfinis mirini|FUPR1253-10|LBPV-32341
Imparfinis mirini|FUPR1254-10|LBPV-32342
Imparfinis mirini|FUPR478-09|LBPV-22575
Imparfinis mirini|FUPR1257-10|LBPV-32345
Imparfinis schubarti|FUPR476-09|LBPV-35481
Imparfinis schubarti|FUPR475-09|LBPV-35480
Imparfinis schubarti|FUPR776-09|LBPV-34204
Imparfinis schubarti|FUPR775-09|LBPV-34203
Imparfinis schubarti|FUPR479-09|LBPV-26417
Imparfinis schubarti|FUPR473-09|LBPV-35478
Imparfinis schubarti|FUPR474-09|LBPV-35479
Imparfinis schubarti|FUPR477-09|LBPV-35482
Chasmocranus brachynema|FUPR471-09|LBPV-29948
Chasmocranus brachynema|FUPR469-09|LBPV-29946
Chasmocranus brachynema|FUPR472-09|LBPV-29949
Chasmocranus brachynema|FUPR470-09|LBPV-29947
Chasmocranus sp.|FUPR1309-10|LBPV-44587
Heptapterus multiradiatus|FUPR792-09|LBPV-37225
Heptapterus multiradiatus|FUPR791-09|LBPV-37224
Rhamdiopsis microcephala|FUPR494-09|LBPV-29187
Rhamdiopsis microcephala|FUPR492-09|LBPV-29185
Rhamdiopsis microcephala|FUPR491-09|LBPV-29184
Rhamdiopsis microcephala|FUPR490-09|LBPV-29183
Rhamdiopsis microcephala|FUPR493-09|LBPV-29186
Microglanis garavelloi|FUPR695-09|LBPV-10557
Microglanis garavelloi|FUPR697-09|LBPV-10562
Microglanis garavelloi|FUPR694-09|LBPV-22540
Microglanis garavelloi|FUPR693-09|LBPV-22539
Microglanis garavelloi|FUPR696-09|LBPV-10561
Pseudopimelodus mangurus|FUPR698-09|LBPV-37226
Pseudopimelodus mangurus|FUPR700-09|LBPV-37228
Pseudopimelodus mangurus|FUPR699-09|LBPV-37227
Pseudopimelodus sp.|FUPR702-09|LBPV-17433
Pterodoras granulosus|FUPR529-09|LBPV-19260
Pterodoras granulosus|FUPR525-09|LBPV-26764
Pterodoras granulosus|FUPR528-09|LBPV-19258
Pterodoras granulosus|FUPR527-09|LBPV-19261
Pterodoras granulosus|FUPR526-09|LBPV-19259
Rhinodoras dorbignyi|FUPR530-09|LBPV-17032
Rhinodoras dorbignyi|FUPR531-09|LBPV-10334
Cetopsis gobioides|FUPR1265-10|LBPV-38283

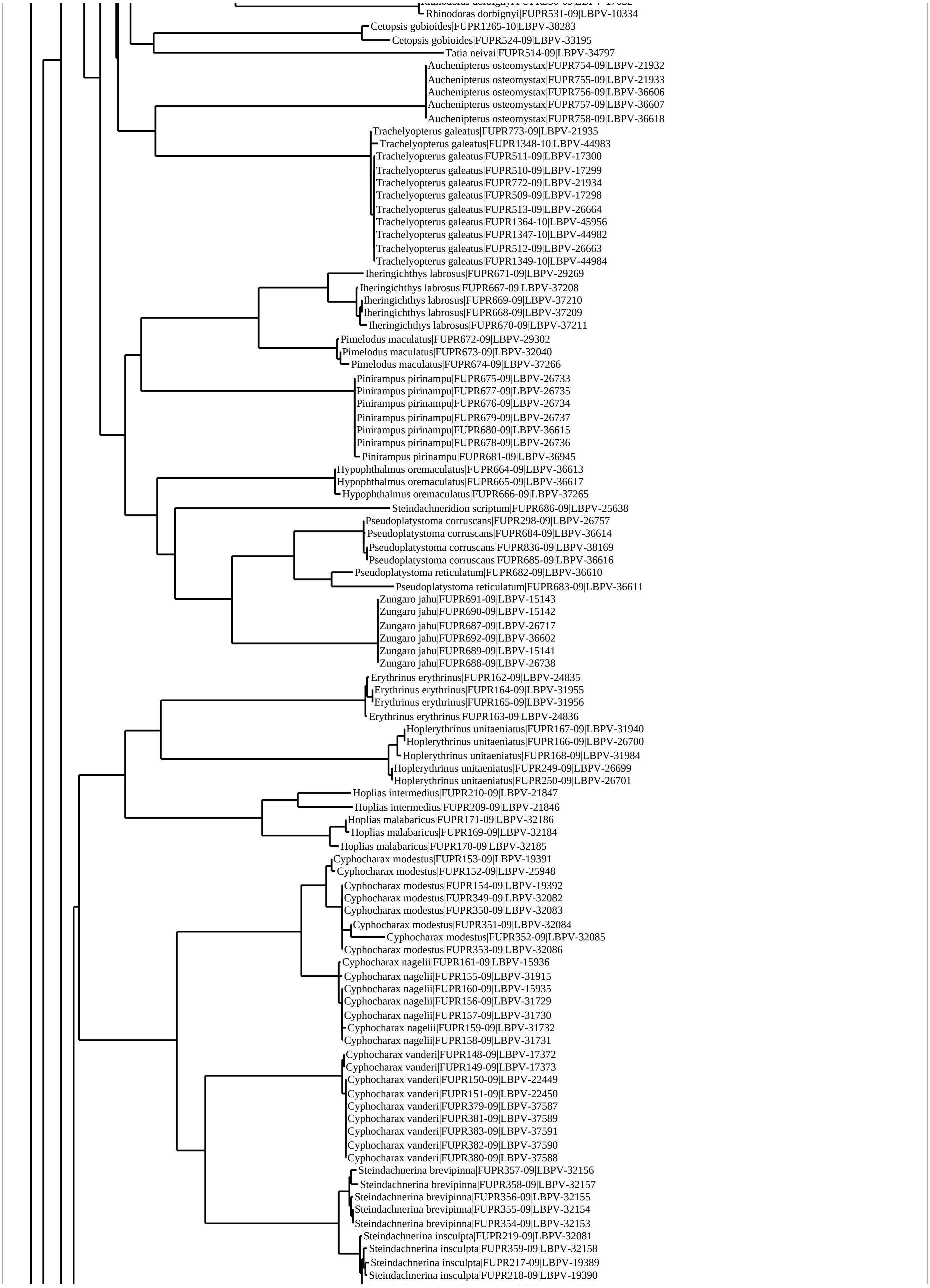
Rhinodoras dorbignyi|FUPR531-09|LBPV-10334
Cetopsis gobioides|FUPR1265-10|LBPV-38283
Cetopsis gobioides|FUPR524-09|LBPV-33195
Tatia neivai|FUPR514-09|LBPV-34797
Auchenipterus osteomystax|FUPR754-09|LBPV-21932
Auchenipterus osteomystax|FUPR755-09|LBPV-21933
Auchenipterus osteomystax|FUPR756-09|LBPV-36606
Auchenipterus osteomystax|FUPR757-09|LBPV-36607
Auchenipterus osteomystax|FUPR758-09|LBPV-36618
Trachelyopterus galeatus|FUPR773-09|LBPV-21935
Trachelyopterus galeatus|FUPR1348-10|LBPV-44983
Trachelyopterus galeatus|FUPR511-09|LBPV-17300
Trachelyopterus galeatus|FUPR510-09|LBPV-17299
Trachelyopterus galeatus|FUPR772-09|LBPV-21934
Trachelyopterus galeatus|FUPR509-09|LBPV-17298
Trachelyopterus galeatus|FUPR513-09|LBPV-26664
Trachelyopterus galeatus|FUPR1364-10|LBPV-45956
Trachelyopterus galeatus|FUPR1347-10|LBPV-44982
Trachelyopterus galeatus|FUPR512-09|LBPV-26663
Trachelyopterus galeatus|FUPR1349-10|LBPV-44984
Iheringichthys labrosus|FUPR671-09|LBPV-29269
Iheringichthys labrosus|FUPR667-09|LBPV-37208
Iheringichthys labrosus|FUPR669-09|LBPV-37210
Iheringichthys labrosus|FUPR668-09|LBPV-37209
Iheringichthys labrosus|FUPR670-09|LBPV-37211
Pimelodus maculatus|FUPR672-09|LBPV-29302
Pimelodus maculatus|FUPR673-09|LBPV-32040
Pimelodus maculatus|FUPR674-09|LBPV-37266
Pinirampus pirinampu|FUPR675-09|LBPV-26733
Pinirampus pirinampu|FUPR677-09|LBPV-26735
Pinirampus pirinampu|FUPR676-09|LBPV-26734
Pinirampus pirinampu|FUPR679-09|LBPV-26737
Pinirampus pirinampu|FUPR680-09|LBPV-36615
Pinirampus pirinampu|FUPR678-09|LBPV-26736
Pinirampus pirinampu|FUPR681-09|LBPV-36945
Hypophthalmus oremaculatus|FUPR664-09|LBPV-36613
Hypophthalmus oremaculatus|FUPR665-09|LBPV-36617
Hypophthalmus oremaculatus|FUPR666-09|LBPV-37265
Steindachneridion scriptum|FUPR686-09|LBPV-25638
Pseudoplatystoma corruscans|FUPR298-09|LBPV-26757
Pseudoplatystoma corruscans|FUPR684-09|LBPV-36614
Pseudoplatystoma corruscans|FUPR836-09|LBPV-38169
Pseudoplatystoma corruscans|FUPR685-09|LBPV-36616
Pseudoplatystoma reticulatum|FUPR682-09|LBPV-36610
Pseudoplatystoma reticulatum|FUPR683-09|LBPV-36611
Zungaro jahu|FUPR691-09|LBPV-15143
Zungaro jahu|FUPR690-09|LBPV-15142
Zungaro jahu|FUPR687-09|LBPV-26717
Zungaro jahu|FUPR692-09|LBPV-36602
Zungaro jahu|FUPR689-09|LBPV-15141
Zungaro jahu|FUPR688-09|LBPV-26738
Erythrinus erythrinus|FUPR162-09|LBPV-24835
Erythrinus erythrinus|FUPR164-09|LBPV-31955
Erythrinus erythrinus|FUPR165-09|LBPV-31956
Erythrinus erythrinus|FUPR163-09|LBPV-24836
Hoplerythrinus unitaeniatus|FUPR167-09|LBPV-31940
Hoplerythrinus unitaeniatus|FUPR166-09|LBPV-26700
Hoplerythrinus unitaeniatus|FUPR168-09|LBPV-31984
Hoplerythrinus unitaeniatus|FUPR249-09|LBPV-26699
Hoplerythrinus unitaeniatus|FUPR250-09|LBPV-26701
Hoplias intermedius|FUPR210-09|LBPV-21847
Hoplias intermedius|FUPR209-09|LBPV-21846
Hoplias malabaricus|FUPR171-09|LBPV-32186
Hoplias malabaricus|FUPR169-09|LBPV-32184
Hoplias malabaricus|FUPR170-09|LBPV-32185
Cyphocharax modestus|FUPR153-09|LBPV-19391
Cyphocharax modestus|FUPR152-09|LBPV-25948
Cyphocharax modestus|FUPR154-09|LBPV-19392
Cyphocharax modestus|FUPR349-09|LBPV-32082
Cyphocharax modestus|FUPR350-09|LBPV-32083
Cyphocharax modestus|FUPR351-09|LBPV-32084
Cyphocharax modestus|FUPR352-09|LBPV-32085
Cyphocharax modestus|FUPR353-09|LBPV-32086
Cyphocharax nagelii|FUPR161-09|LBPV-15936
Cyphocharax nagelii|FUPR155-09|LBPV-31915
Cyphocharax nagelii|FUPR160-09|LBPV-15935
Cyphocharax nagelii|FUPR156-09|LBPV-31729
Cyphocharax nagelii|FUPR157-09|LBPV-31730
Cyphocharax nagelii|FUPR159-09|LBPV-31732
Cyphocharax nagelii|FUPR158-09|LBPV-31731
Cyphocharax vanderi|FUPR148-09|LBPV-17372
Cyphocharax vanderi|FUPR149-09|LBPV-17373
Cyphocharax vanderi|FUPR150-09|LBPV-22449
Cyphocharax vanderi|FUPR151-09|LBPV-22450
Cyphocharax vanderi|FUPR379-09|LBPV-37587
Cyphocharax vanderi|FUPR381-09|LBPV-37589
Cyphocharax vanderi|FUPR383-09|LBPV-37591
Cyphocharax vanderi|FUPR382-09|LBPV-37590
Cyphocharax vanderi|FUPR380-09|LBPV-37588
Steindachnerina brevipinna|FUPR357-09|LBPV-32156
Steindachnerina brevipinna|FUPR358-09|LBPV-32157
Steindachnerina brevipinna|FUPR356-09|LBPV-32155
Steindachnerina brevipinna|FUPR355-09|LBPV-32154
Steindachnerina brevipinna|FUPR354-09|LBPV-32153
Steindachnerina insculpta|FUPR219-09|LBPV-32081
Steindachnerina insculpta|FUPR359-09|LBPV-32158
Steindachnerina insculpta|FUPR217-09|LBPV-19389
Steindachnerina insculpta|FUPR218-09|LBPV-19390

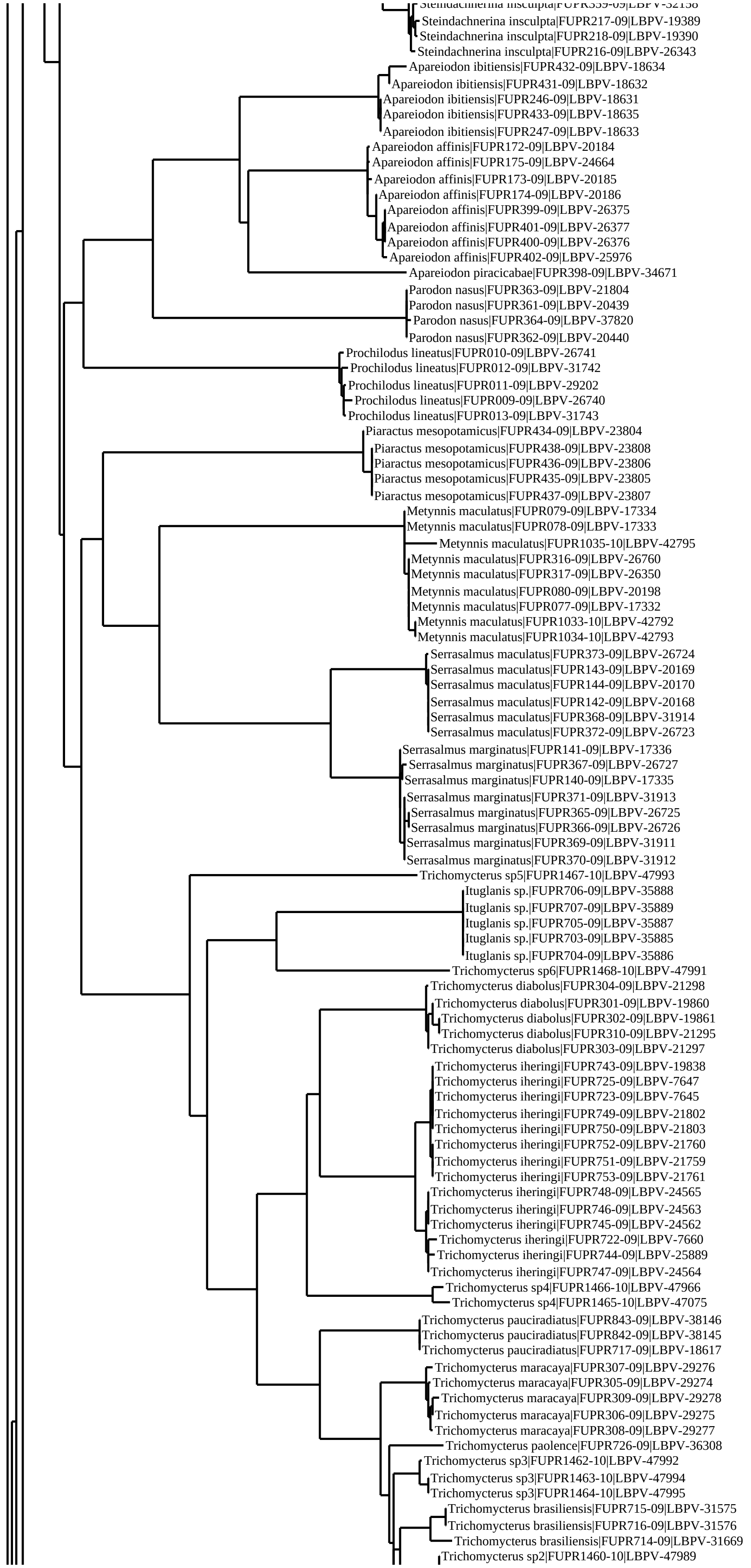
Steindachnerina insculpta|FUPR217-09|LBPV-19389
Steindachnerina insculpta|FUPR218-09|LBPV-19390
Steindachnerina insculpta|FUPR216-09|LBPV-26343
Apareiodon ibitiensis|FUPR432-09|LBPV-18634
Apareiodon ibitiensis|FUPR431-09|LBPV-18632
Apareiodon ibitiensis|FUPR246-09|LBPV-18631
Apareiodon ibitiensis|FUPR433-09|LBPV-18635
Apareiodon ibitiensis|FUPR247-09|LBPV-18633
Apareiodon affinis|FUPR172-09|LBPV-20184
Apareiodon affinis|FUPR175-09|LBPV-24664
Apareiodon affinis|FUPR173-09|LBPV-20185
Apareiodon affinis|FUPR174-09|LBPV-20186
Apareiodon affinis|FUPR399-09|LBPV-26375
Apareiodon affinis|FUPR401-09|LBPV-26377
Apareiodon affinis|FUPR400-09|LBPV-26376
Apareiodon affinis|FUPR402-09|LBPV-25976
Apareiodon piracicabae|FUPR398-09|LBPV-34671
Parodon nasus|FUPR363-09|LBPV-21804
Parodon nasus|FUPR361-09|LBPV-20439
Parodon nasus|FUPR364-09|LBPV-37820
Parodon nasus|FUPR362-09|LBPV-20440
Prochilodus lineatus|FUPR010-09|LBPV-26741
Prochilodus lineatus|FUPR012-09|LBPV-31742
Prochilodus lineatus|FUPR011-09|LBPV-29202
Prochilodus lineatus|FUPR009-09|LBPV-26740
Prochilodus lineatus|FUPR013-09|LBPV-31743
Piaractus mesopotamicus|FUPR434-09|LBPV-23804
Piaractus mesopotamicus|FUPR438-09|LBPV-23808
Piaractus mesopotamicus|FUPR436-09|LBPV-23806
Piaractus mesopotamicus|FUPR435-09|LBPV-23805
Piaractus mesopotamicus|FUPR437-09|LBPV-23807
Metynnis maculatus|FUPR079-09|LBPV-17334
Metynnis maculatus|FUPR078-09|LBPV-17333
Metynnis maculatus|FUPR1035-10|LBPV-42795
Metynnis maculatus|FUPR316-09|LBPV-26760
Metynnis maculatus|FUPR317-09|LBPV-26350
Metynnis maculatus|FUPR080-09|LBPV-20198
Metynnis maculatus|FUPR077-09|LBPV-17332
Metynnis maculatus|FUPR1033-10|LBPV-42792
Metynnis maculatus|FUPR1034-10|LBPV-42793
Serrasalmus maculatus|FUPR373-09|LBPV-26724
Serrasalmus maculatus|FUPR143-09|LBPV-20169
Serrasalmus maculatus|FUPR144-09|LBPV-20170
Serrasalmus maculatus|FUPR142-09|LBPV-20168
Serrasalmus maculatus|FUPR368-09|LBPV-31914
Serrasalmus maculatus|FUPR372-09|LBPV-26723
Serrasalmus marginatus|FUPR141-09|LBPV-17336
Serrasalmus marginatus|FUPR367-09|LBPV-26727
Serrasalmus marginatus|FUPR140-09|LBPV-17335
Serrasalmus marginatus|FUPR371-09|LBPV-31913
Serrasalmus marginatus|FUPR365-09|LBPV-26725
Serrasalmus marginatus|FUPR366-09|LBPV-26726
Serrasalmus marginatus|FUPR369-09|LBPV-31911
Serrasalmus marginatus|FUPR370-09|LBPV-31912
Trichomycterus sp5|FUPR1467-10|LBPV-47993
Ituglanis sp.|FUPR706-09|LBPV-35888
Ituglanis sp.|FUPR707-09|LBPV-35889
Ituglanis sp.|FUPR705-09|LBPV-35887
Ituglanis sp.|FUPR703-09|LBPV-35885
Ituglanis sp.|FUPR704-09|LBPV-35886
Trichomycterus sp6|FUPR1468-10|LBPV-47991
Trichomycterus diabolus|FUPR304-09|LBPV-21298
Trichomycterus diabolus|FUPR301-09|LBPV-19860
Trichomycterus diabolus|FUPR302-09|LBPV-19861
Trichomycterus diabolus|FUPR310-09|LBPV-21295
Trichomycterus diabolus|FUPR303-09|LBPV-21297
Trichomycterus iheringi|FUPR743-09|LBPV-19838
Trichomycterus iheringi|FUPR725-09|LBPV-7647
Trichomycterus iheringi|FUPR723-09|LBPV-7645
Trichomycterus iheringi|FUPR749-09|LBPV-21802
Trichomycterus iheringi|FUPR750-09|LBPV-21803
Trichomycterus iheringi|FUPR752-09|LBPV-21760
Trichomycterus iheringi|FUPR751-09|LBPV-21759
Trichomycterus iheringi|FUPR753-09|LBPV-21761
Trichomycterus iheringi|FUPR748-09|LBPV-24565
Trichomycterus iheringi|FUPR746-09|LBPV-24563
Trichomycterus iheringi|FUPR745-09|LBPV-24562
Trichomycterus iheringi|FUPR722-09|LBPV-7660
Trichomycterus iheringi|FUPR744-09|LBPV-25889
Trichomycterus iheringi|FUPR747-09|LBPV-24564
Trichomycterus sp4|FUPR1466-10|LBPV-47966
Trichomycterus sp4|FUPR1465-10|LBPV-47075
Trichomycterus pauciradiatus|FUPR843-09|LBPV-38146
Trichomycterus pauciradiatus|FUPR842-09|LBPV-38145
Trichomycterus pauciradiatus|FUPR717-09|LBPV-18617
Trichomycterus maracaya|FUPR307-09|LBPV-29276
Trichomycterus maracaya|FUPR305-09|LBPV-29274
Trichomycterus maracaya|FUPR309-09|LBPV-29278
Trichomycterus maracaya|FUPR306-09|LBPV-29275
Trichomycterus maracaya|FUPR308-09|LBPV-29277
Trichomycterus paolence|FUPR726-09|LBPV-36308
Trichomycterus sp3|FUPR1462-10|LBPV-47992
Trichomycterus sp3|FUPR1463-10|LBPV-47994
Trichomycterus sp3|FUPR1464-10|LBPV-47995
Trichomycterus brasiliensis|FUPR715-09|LBPV-31575
Trichomycterus brasiliensis|FUPR716-09|LBPV-31576
Trichomycterus brasiliensis|FUPR714-09|LBPV-31669
Trichomycterus sp2|FUPR1460-10|LBPV-47989

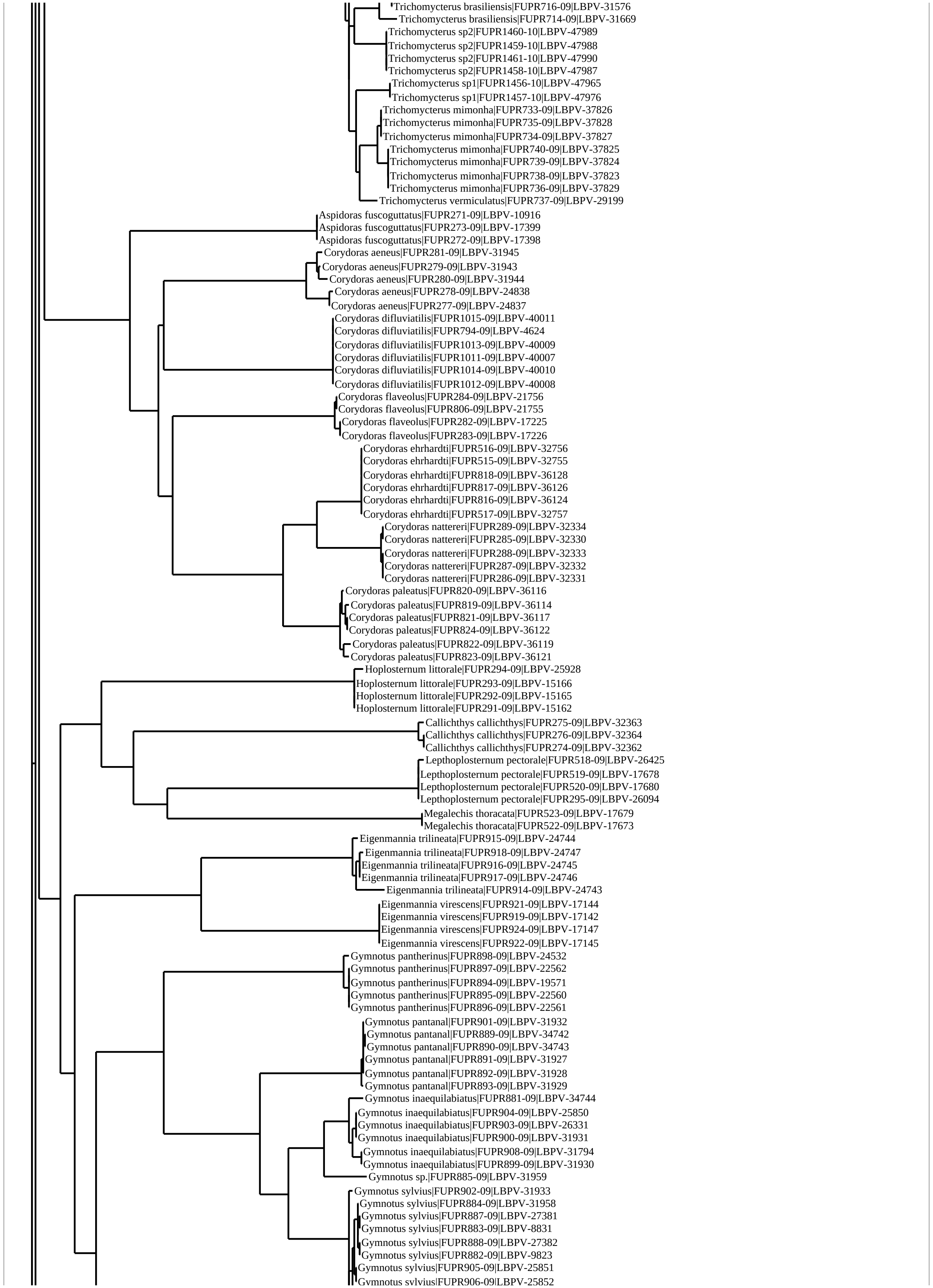
Trichomycterus brasiliensis|FUPR716-09|LBPV-31576
Trichomycterus brasiliensis|FUPR714-09|LBPV-31669
Trichomycterus sp2|FUPR1460-10|LBPV-47989
Trichomycterus sp2|FUPR1459-10|LBPV-47988
Trichomycterus sp2|FUPR1461-10|LBPV-47990
Trichomycterus sp2|FUPR1458-10|LBPV-47987
Trichomycterus sp1|FUPR1456-10|LBPV-47965
Trichomycterus sp1|FUPR1457-10|LBPV-47976
Trichomycterus mimonha|FUPR733-09|LBPV-37826
Trichomycterus mimonha|FUPR735-09|LBPV-37828
Trichomycterus mimonha|FUPR734-09|LBPV-37827
Trichomycterus mimonha|FUPR740-09|LBPV-37825
Trichomycterus mimonha|FUPR739-09|LBPV-37824
Trichomycterus mimonha|FUPR738-09|LBPV-37823
Trichomycterus mimonha|FUPR736-09|LBPV-37829
Trichomycterus vermiculatus|FUPR737-09|LBPV-29199
Aspidoras fuscoguttatus|FUPR271-09|LBPV-10916
Aspidoras fuscoguttatus|FUPR273-09|LBPV-17399
Aspidoras fuscoguttatus|FUPR272-09|LBPV-17398
Corydoras aeneus|FUPR281-09|LBPV-31945
Corydoras aeneus|FUPR279-09|LBPV-31943
Corydoras aeneus|FUPR280-09|LBPV-31944
Corydoras aeneus|FUPR278-09|LBPV-24838
Corydoras aeneus|FUPR277-09|LBPV-24837
Corydoras difluviatilis|FUPR1015-09|LBPV-40011
Corydoras difluviatilis|FUPR794-09|LBPV-4624
Corydoras difluviatilis|FUPR1013-09|LBPV-40009
Corydoras difluviatilis|FUPR1011-09|LBPV-40007
Corydoras difluviatilis|FUPR1014-09|LBPV-40010
Corydoras difluviatilis|FUPR1012-09|LBPV-40008
Corydoras flaveolus|FUPR284-09|LBPV-21756
Corydoras flaveolus|FUPR806-09|LBPV-21755
Corydoras flaveolus|FUPR282-09|LBPV-17225
Corydoras flaveolus|FUPR283-09|LBPV-17226
Corydoras ehrhardti|FUPR516-09|LBPV-32756
Corydoras ehrhardti|FUPR515-09|LBPV-32755
Corydoras ehrhardti|FUPR818-09|LBPV-36128
Corydoras ehrhardti|FUPR817-09|LBPV-36126
Corydoras ehrhardti|FUPR816-09|LBPV-36124
Corydoras ehrhardti|FUPR517-09|LBPV-32757
Corydoras nattereri|FUPR289-09|LBPV-32334
Corydoras nattereri|FUPR285-09|LBPV-32330
Corydoras nattereri|FUPR288-09|LBPV-32333
Corydoras nattereri|FUPR287-09|LBPV-32332
Corydoras nattereri|FUPR286-09|LBPV-32331
Corydoras paleatus|FUPR820-09|LBPV-36116
Corydoras paleatus|FUPR819-09|LBPV-36114
Corydoras paleatus|FUPR821-09|LBPV-36117
Corydoras paleatus|FUPR824-09|LBPV-36122
Corydoras paleatus|FUPR822-09|LBPV-36119
Corydoras paleatus|FUPR823-09|LBPV-36121
Hoplosternum littorale|FUPR294-09|LBPV-25928
Hoplosternum littorale|FUPR293-09|LBPV-15166
Hoplosternum littorale|FUPR292-09|LBPV-15165
Hoplosternum littorale|FUPR291-09|LBPV-15162
Callichthys callichthys|FUPR275-09|LBPV-32363
Callichthys callichthys|FUPR276-09|LBPV-32364
Callichthys callichthys|FUPR274-09|LBPV-32362
Lepthoplosternum pectorale|FUPR518-09|LBPV-26425
Lepthoplosternum pectorale|FUPR519-09|LBPV-17678
Lepthoplosternum pectorale|FUPR520-09|LBPV-17680
Lepthoplosternum pectorale|FUPR295-09|LBPV-26094
Megalechis thoracata|FUPR523-09|LBPV-17679
Megalechis thoracata|FUPR522-09|LBPV-17673
Eigenmannia trilineata|FUPR915-09|LBPV-24744
Eigenmannia trilineata|FUPR918-09|LBPV-24747
Eigenmannia trilineata|FUPR916-09|LBPV-24745
Eigenmannia trilineata|FUPR917-09|LBPV-24746
Eigenmannia trilineata|FUPR914-09|LBPV-24743
Eigenmannia virescens|FUPR921-09|LBPV-17144
Eigenmannia virescens|FUPR919-09|LBPV-17142
Eigenmannia virescens|FUPR924-09|LBPV-17147
Eigenmannia virescens|FUPR922-09|LBPV-17145
Gymnotus pantherinus|FUPR898-09|LBPV-24532
Gymnotus pantherinus|FUPR897-09|LBPV-22562
Gymnotus pantherinus|FUPR894-09|LBPV-19571
Gymnotus pantherinus|FUPR895-09|LBPV-22560
Gymnotus pantherinus|FUPR896-09|LBPV-22561
Gymnotus pantanal|FUPR901-09|LBPV-31932
Gymnotus pantanal|FUPR889-09|LBPV-34742
Gymnotus pantanal|FUPR890-09|LBPV-34743
Gymnotus pantanal|FUPR891-09|LBPV-31927
Gymnotus pantanal|FUPR892-09|LBPV-31928
Gymnotus pantanal|FUPR893-09|LBPV-31929
Gymnotus inaequilabiatus|FUPR881-09|LBPV-34744
Gymnotus inaequilabiatus|FUPR904-09|LBPV-25850
Gymnotus inaequilabiatus|FUPR903-09|LBPV-26331
Gymnotus inaequilabiatus|FUPR900-09|LBPV-31931
Gymnotus inaequilabiatus|FUPR908-09|LBPV-31794
Gymnotus inaequilabiatus|FUPR899-09|LBPV-31930
Gymnotus sp.|FUPR885-09|LBPV-31959
Gymnotus sylvius|FUPR902-09|LBPV-31933
Gymnotus sylvius|FUPR884-09|LBPV-31958
Gymnotus sylvius|FUPR887-09|LBPV-27381
Gymnotus sylvius|FUPR883-09|LBPV-8831
Gymnotus sylvius|FUPR888-09|LBPV-27382
Gymnotus sylvius|FUPR882-09|LBPV-9823
Gymnotus sylvius|FUPR905-09|LBPV-25851
Gymnotus sylvius|FUPR906-09|LBPV-25852

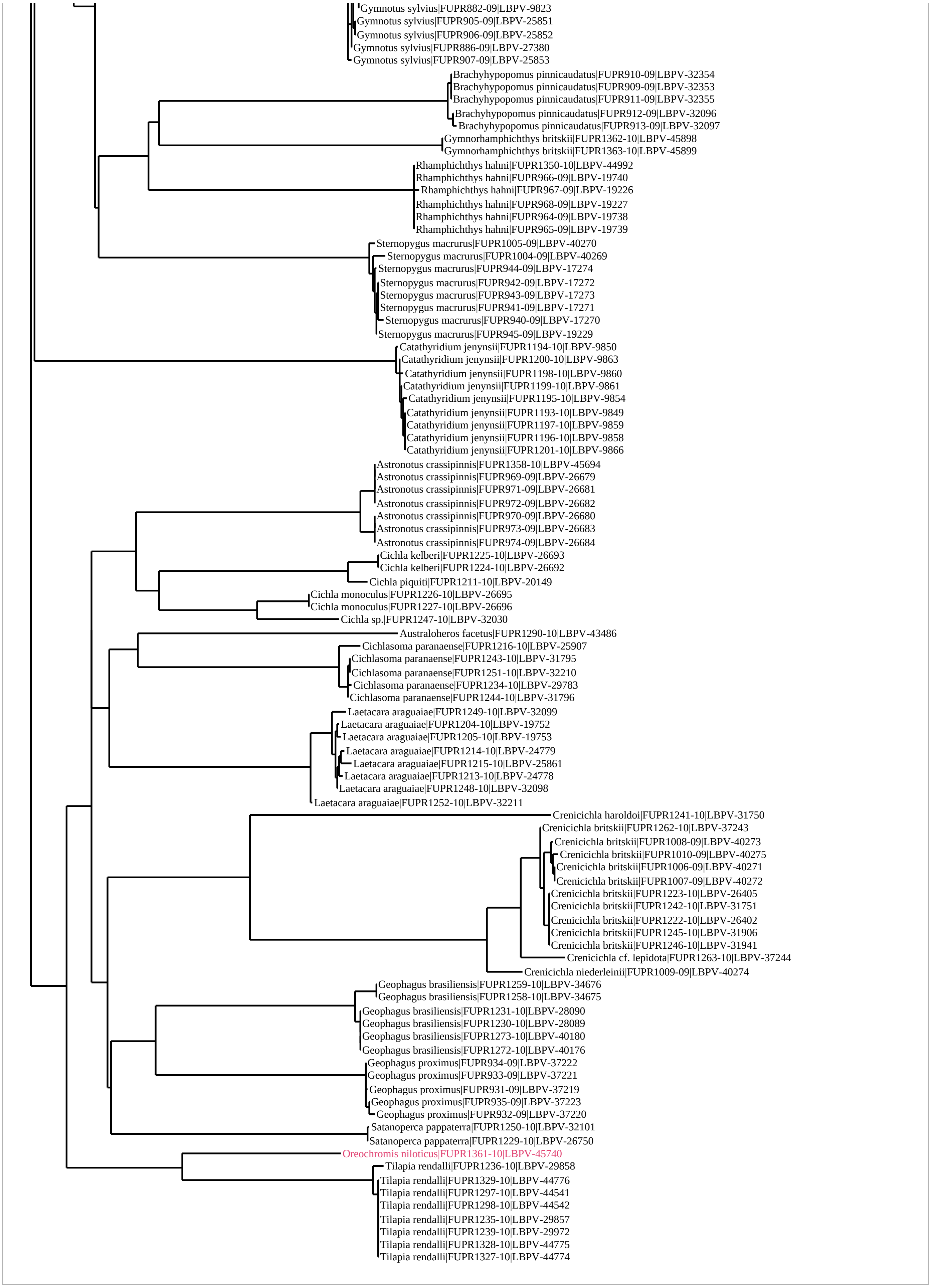
Gymnotus sylvius|FUPR882-09|LBPV-9823
Gymnotus sylvius|FUPR905-09|LBPV-25851
Gymnotus sylvius|FUPR906-09|LBPV-25852
Gymnotus sylvius|FUPR886-09|LBPV-27380
Gymnotus sylvius|FUPR907-09|LBPV-25853
Brachyhypopomus pinnicaudatus|FUPR910-09|LBPV-32354
Brachyhypopomus pinnicaudatus|FUPR909-09|LBPV-32353
Brachyhypopomus pinnicaudatus|FUPR911-09|LBPV-32355
Brachyhypopomus pinnicaudatus|FUPR912-09|LBPV-32096
Brachyhypopomus pinnicaudatus|FUPR913-09|LBPV-32097
Gymnorhamphichthys britskii|FUPR1362-10|LBPV-45898
Gymnorhamphichthys britskii|FUPR1363-10|LBPV-45899
Rhamphichthys hahni|FUPR1350-10|LBPV-44992
Rhamphichthys hahni|FUPR966-09|LBPV-19740
Rhamphichthys hahni|FUPR967-09|LBPV-19226
Rhamphichthys hahni|FUPR968-09|LBPV-19227
Rhamphichthys hahni|FUPR964-09|LBPV-19738
Rhamphichthys hahni|FUPR965-09|LBPV-19739
Sternopygus macrurus|FUPR1005-09|LBPV-40270
Sternopygus macrurus|FUPR1004-09|LBPV-40269
Sternopygus macrurus|FUPR944-09|LBPV-17274
Sternopygus macrurus|FUPR942-09|LBPV-17272
Sternopygus macrurus|FUPR943-09|LBPV-17273
Sternopygus macrurus|FUPR941-09|LBPV-17271
Sternopygus macrurus|FUPR940-09|LBPV-17270
Sternopygus macrurus|FUPR945-09|LBPV-19229
Catathyridium jenynsii|FUPR1194-10|LBPV-9850
Catathyridium jenynsii|FUPR1200-10|LBPV-9863
Catathyridium jenynsii|FUPR1198-10|LBPV-9860
Catathyridium jenynsii|FUPR1199-10|LBPV-9861
Catathyridium jenynsii|FUPR1195-10|LBPV-9854
Catathyridium jenynsii|FUPR1193-10|LBPV-9849
Catathyridium jenynsii|FUPR1197-10|LBPV-9859
Catathyridium jenynsii|FUPR1196-10|LBPV-9858
Catathyridium jenynsii|FUPR1201-10|LBPV-9866
Astronotus crassipinnis|FUPR1358-10|LBPV-45694
Astronotus crassipinnis|FUPR969-09|LBPV-26679
Astronotus crassipinnis|FUPR971-09|LBPV-26681
Astronotus crassipinnis|FUPR972-09|LBPV-26682
Astronotus crassipinnis|FUPR970-09|LBPV-26680
Astronotus crassipinnis|FUPR973-09|LBPV-26683
Astronotus crassipinnis|FUPR974-09|LBPV-26684
Cichla kelberi|FUPR1225-10|LBPV-26693
Cichla kelberi|FUPR1224-10|LBPV-26692
Cichla piquiti|FUPR1211-10|LBPV-20149
Cichla monoculus|FUPR1226-10|LBPV-26695
Cichla monoculus|FUPR1227-10|LBPV-26696
Cichla sp.|FUPR1247-10|LBPV-32030
Australoheros facetus|FUPR1290-10|LBPV-43486
Cichlasoma paranaense|FUPR1216-10|LBPV-25907
Cichlasoma paranaense|FUPR1243-10|LBPV-31795
Cichlasoma paranaense|FUPR1251-10|LBPV-32210
Cichlasoma paranaense|FUPR1234-10|LBPV-29783
Cichlasoma paranaense|FUPR1244-10|LBPV-31796
Laetacara araguaiae|FUPR1249-10|LBPV-32099
Laetacara araguaiae|FUPR1204-10|LBPV-19752
Laetacara araguaiae|FUPR1205-10|LBPV-19753
Laetacara araguaiae|FUPR1214-10|LBPV-24779
Laetacara araguaiae|FUPR1215-10|LBPV-25861
Laetacara araguaiae|FUPR1213-10|LBPV-24778
Laetacara araguaiae|FUPR1248-10|LBPV-32098
Laetacara araguaiae|FUPR1252-10|LBPV-32211
Crenicichla haroldoi|FUPR1241-10|LBPV-31750
Crenicichla britskii|FUPR1262-10|LBPV-37243
Crenicichla britskii|FUPR1008-09|LBPV-40273
Crenicichla britskii|FUPR1010-09|LBPV-40275
Crenicichla britskii|FUPR1006-09|LBPV-40271
Crenicichla britskii|FUPR1007-09|LBPV-40272
Crenicichla britskii|FUPR1223-10|LBPV-26405
Crenicichla britskii|FUPR1242-10|LBPV-31751
Crenicichla britskii|FUPR1222-10|LBPV-26402
Crenicichla britskii|FUPR1245-10|LBPV-31906
Crenicichla britskii|FUPR1246-10|LBPV-31941
Crenicichla cf. lepidota|FUPR1263-10|LBPV-37244
Crenicichla niederleinii|FUPR1009-09|LBPV-40274
Geophagus brasiliensis|FUPR1259-10|LBPV-34676
Geophagus brasiliensis|FUPR1258-10|LBPV-34675
Geophagus brasiliensis|FUPR1231-10|LBPV-28090
Geophagus brasiliensis|FUPR1230-10|LBPV-28089
Geophagus brasiliensis|FUPR1273-10|LBPV-40180
Geophagus brasiliensis|FUPR1272-10|LBPV-40176
Geophagus proximus|FUPR934-09|LBPV-37222
Geophagus proximus|FUPR933-09|LBPV-37221
Geophagus proximus|FUPR931-09|LBPV-37219
Geophagus proximus|FUPR935-09|LBPV-37223
Geophagus proximus|FUPR932-09|LBPV-37220
Satanoperca pappaterra|FUPR1250-10|LBPV-32101
Satanoperca pappaterra|FUPR1229-10|LBPV-26750
Oreochromis niloticus|FUPR1361-10|LBPV-45740
Tilapia rendalli|FUPR1236-10|LBPV-29858
Tilapia rendalli|FUPR1329-10|LBPV-44776
Tilapia rendalli|FUPR1297-10|LBPV-44541
Tilapia rendalli|FUPR1298-10|LBPV-44542
Tilapia rendalli|FUPR1235-10|LBPV-29857
Tilapia rendalli|FUPR1239-10|LBPV-29972
Tilapia rendalli|FUPR1328-10|LBPV-44775
Tilapia rendalli|FUPR1327-10|LBPV-44774
