# Supplementary material for: Can DNA barcoding accurately discriminate megadiverse Neotropical freshwater fish fauna?
Source: BMC Genet. 2013 Mar 9;14:20. doi: 10.1186/1471-2156-14-20 (PMC3608943; doi:10.1186/1471-2156-14-20)
Supplement: Additional file 3 — Pairs of species that showed low K2P distance genetic values (<2%). [file 1471-2156-14-20-S3.docx]

**Additional file 3:** Pairs of species that showed low K2P distance genetic values (<2%).

| **Pair of Species** | | | |  |  |
| --- | --- | --- | --- | --- | --- |
| **Mean Intra-specific**  **Divergence (%)** | | | | **Inter-specific Divergence (%)** | **Barcode Gap** |
| *Astyanax schubarti* | 0.2 | 0.3 | *Astyanax trierytjropterus* | 0.3 | 1-1.5 |
| *Astyanax bockmanni* | 0.1 | 0.2 | *Astyanax paranae* | 0.7 | 3.5-7 |
| *Characidium aff. Zebra* | 0.1 | 0.1 | *Characidium xantopterum* | 1.0 | 10 |
| *Serrapinnus notomelas* | 0.5 | - | *Serrapinnus sp1* | 1.4 | 2.8 |
| *Astyanax bockmanni* | 0.1 | 0 | *Hyphessobrycon sp1* | 1.5 | 15 |
| *Oligosarcus paranensis* | 1.3 | 0 | *Oligosarcus pintoi* | 1.6 | 1.2 |
| *Corumbataia britskii* | 0.6 | 0 | *Corumbataia cuestae* | 1.6 | 2.7 |
| *Steindachnerina brevipinna* | 0.3 | 0.2 | *Steindachnerina insculpta* | 1.6 | 5.3-8 |
| *Trichomycterus mimonha* | 0.3 | - | *Trichomycterus vermiculatus* | 1.6 | 5.3 |
| *Astyanax fasciatus* | 0 | 0.4 | *Astyanax biotae* | 1.7 | 5.2 |
| *Cichla kilberi* | 0 | - | *Cichla piquiti* | 1.8 | - |
| *Pimelodella meeki* | 0 | - | *Pimelodella sp1* | 1.8 | - |
| *Potamotrygon motoro* | 0.1 | 0 | *Potamotrygon falkineri* | 1.8 | 18 |
| *Hypostomus heraldoi* | 0 | 0 | *Hypostomus albapunctatus* | 0.6 | - |
| *Hypostomus regani* | 0.5 | 0 | *Hypostomus myersi* | 0.6 | 3 |
| *Hypostomus hernanni* | 0.4 | 0 | *Hypostomus nigromaculatus* | 0.8 | 2 |
| *Hypostomus hernanni* | 0.4 | 0.3 | *Hypostomus sp.* | 0.8 | 2-2.6 |
| *Hypostomus regani* | 0.5 | 0 | *Hypostomus nigromaculatus* | 0.8 | 1.6 |
| *Hypostomus regani* | 0.5 | 0.3 | *Hypostomus sp.* | 0.8 | 1.6-2.6 |
| *Hypostomus myersi* | 0 | 0 | *Hypostomus nigromaculatus* | 0.8 | - |
| *Hypostomus commersoni* | 0.4 | 0.2 | *Hypostomus ancistroides* | 0.9 | 2.2-4.5 |
| *Hypostomus hernanni* | 0.4 | 0.5 | *Hypostomus regani* | 0.9 | 1.8-2.2 |
| *Hypostomus hernanni* | 0.4 | 0 | *Hypostomus myersi* | 0.9 | 2.2 |
| *Hypostomus myersi* | 0 | 0.3 | *Hypostomus sp.* | 0.9 | 3 |
| *Hypostomus topovae* | 0.3 | 0.3 | *Hypostomus sp.* | 1.0 | 3.3 |
| *Hypostomus ancistroides* | 0.2 | 0.8 | *Hypostomus derbyi* | 1.0 | 1.2-5 |
| *Hypostomus nigromaculatus* | 0 | 0.3 | *Hypostomus sp.* | 1.0 | 3.3 |
| *Hypostomus commersoni* | 0.4 | 0.8 | *Hypostomus derbyi* | 1.1 | 1.4-2.7 |
| *Hypostomus strigaticeps* | 0.6 | 0.3 | *Hypostomus sp.* | 1.1 | 1.8-3.7 |
| *Hypostomus hernanni* | 0.4 | 0.3 | *Hypostomus topovae* | 1.1 | 2.7-3.7 |
| *Hypostomus strigaticeps* | 0.6 | 0.5 | *Hypostomus regani* | 1.2 | 2-2.4 |
| *Hypostomus topovae* | 0.3 | 0 | *Hypostomus myersi* | 1.2 | 4 |
| *Hypostomus strigaticeps* | 0.6 | 0 | *Hypostomus myersi* | 1.3 | 4.3 |
| *Hypostomus regani* | 0.5 | 0.3 | *Hypostomus topovae* | 1.3 | 2.6-4.3 |
| *Hypostomus sp.* | 0.3 | 0.3 | *Hypostomus microstomus* | 1.3 | 4.3 |
| *Hypostomus strigaticeps* | 0.6 | 0.4 | *Hypostomus hernanni* | 1.4 | 2.3-3.5 |
| *Hypostomus strigaticeps* | 0.6 | 0 | *Hypostomus nigromaculatus* | 1.4 | 2.3 |
| *Hypostomus hernanni* | 0.4 | 0.3 | *Hypostomus microstomus* | 1.4 | 3.5-4.6 |
| *Hypostomus regani* | 0.5 | 0.3 | *Hypostomus microstomus* | 1.4 | 2.8-4.6 |
| *Hypostomus myersi* | 0 | 0.3 | *Hypostomus microstomus* | 1.4 | 4.6 |
| *Hypostomus topovae* | 0.3 | 0 | *Hypostomus nigromaculatus* | 1.5 | 5 |
| *Hypostomus nigromaculatus* | 0 | 0.3 | *Hypostomus microstomus* | 1.5 | 5 |
| *Hypostomus sp.* | 0.3 | - | *Hypostomus iheringi* | 1.5 | 5 |
| *Hypostomus strigaticeps* | 0.6 | 0.3 | *Hypostomus topovae* | 1.6 | 2.7-5.3 |
| *Hypostomus brevis* | 0.3 | 0.2 | *Hypostomus ancistroides* | 1.6 | 5.3-8 |
| *Hypostomus hernanni* | 0.4 | - | *Hypostomus iheringi* | 1.6 | 4 |
| *Hypostomus regani* | 0.5 | - | *Hypostomus iheringi* | 1.6 | 3.2 |
| *Hypostomus myersi* | 0 | - | *Hypostomus iheringi* | 1.6 | - |
| *Hypostomus topovae* | 0.3 | - | *Hypostomus iheringi* | 1.7 | 5.7 |
| *Hypostomus topovae* | 0.3 | 0.3 | *Hypostomus microstomus* | 1.7 | 5.7 |
| *Hypostomus iheringi* | - | 0.3 | *Hypostomus microstomus* | 1.7 | 5.7 |
| *Hypostomus commersoni* | 0.4 | 0.3 | *Hypostomus brevis* | 1.8 | 4.5-6 |
| *Hypostomus nigromaculatus* | 0 | - | *Hypostomus iheringi* | 1.8 | - |
| *Hypostomus strigaticeps* | 0.6 | 0.3 | *Hypostomus microstomus* | 1.9 | 3.2-6.3 |
| *Hypostomus strigaticeps* | 0.6 | 0 | *Hypostomus albapunctatus* | 1.9 | 3.2 |
| *Hypostomus brevis* | 0.3 | 0.8 | *Hypostomus derbyi* | 1.9 | 2.4-6.3 |
| *Neoplecostomus sp3* | 0 | 0 | *Neoplecostomus sp1* | 0.7 | - |
| *Neoplecostomus sp3* | 0 | 0.3 | *Neoplecostomus sp4* | 0.7 | 2.3 |
| *Neoplecostomus botucatu* | 0 | 0 | *Neoplecostomus selenae* | 0.9 | - |
| *Neoplecostomus langeani* | 0 | 0 | *Neoplecostomus sp3* | 1.0 | - |
| *Neoplecostomus langeani* | 0 | 0 | *Neoplecostomus sp1* | 1.0 | - |
| *Neoplecostomus sp8* | 0 | 0 | *Neoplecostomus sp3* | 1.1 | - |
| *Neoplecostomus sp1* | 0 | 0.3 | *Neoplecostomus sp4* | 1.1 | 2.7 |
| *Neoplecostomus sp8* | 0 | 0 | *Neoplecostomus sp1* | 1.3 | - |
| *Neoplecostomus sp8* | 0 | 0.3 | *Neoplecostomus sp4* | 1.4 | 4.7 |
| *Neoplecostomus langeani* | 0 | 0.3 | *Neoplecostomus sp4* | 1.4 | 4.7 |
| *Neoplecostomus sp3* | 0 | 0.3 | *Neoplecostomus paranensis* | 1.4 | 4.7 |
| *Neoplecostomus sp1* | 0 | 0.3 | *Neoplecostomus paranensis* | 1.4 | 4.7 |
| *Neoplecostomus sp5* | 0.3 | 0 | *Neoplecostomus langeani* | 1.5 | 5 |
| *Neoplecostomus langeani* | 0 | 0.2 | *Neoplecostomus sp10* | 1.5 | 7.5 |
| *Neoplecostomus sp2* | 0 | 0 | *Neoplecostomus sp3* | 1.6 | - |
| *Neoplecostomus sp2* | 0 | 0 | *Neoplecostomus sp1* | 1.6 | - |
| *Neoplecostomus sp8* | 0 | 0 | *Neoplecostomus langeani* | 1.6 | - |
| *Neoplecostomus sp5* | 0.3 | 0 | *Neoplecostomus sp1* | 1.7 | 5.7 |
| *Neoplecostomus langeani* | 0 | 0.3 | *Neoplecostomus paranensis* | 1.7 | 5.7 |
| *Neoplecostomus sp10* | 0.2 | 0 | *Neoplecostomus sp3* | 1.7 | 8.5 |
| *Neoplecostomus sp10* | 0.2 | 0 | *Neoplecostomus sp1* | 1.7 | 8.5 |
| *Neoplecostomus paranensis* | 0.3 | 0.3 | *Neoplecostomus sp4* | 1.7 | 5.7 |
| *Neoplecostomus sp2* | 0 | 0.3 | *Neoplecostomus sp4* | 1.8 | 6 |
| *Neoplecostomus sp8* | 0 | 0.2 | *Neoplecostomus bandeirante* | 1.8 | 9 |
| *Neoplecostomus sp8* | 0 | 0.2 | *Neoplecostomus sp10* | 1.9 | 9.5 |
| *Neoplecostomus sp8* | 0 | 0.3 | *Neoplecostomus paranensis* | 1.9 | 6.3 |
| *Neoplecostomus sp10* | 0.2 | 0.9 | *Neoplecostomus sp9* | 1.9 | 2.1-9.5 |
| *Neoplecostomus sp3* | 0 | 0.2 | *Neoplecostomus bandeirante* | 1.9 | 9.5 |
