# Supplementary material for: Can DNA barcoding accurately discriminate megadiverse Neotropical freshwater fish fauna?
Source: BMC Genet. 2013 Mar 9;14:20. doi: 10.1186/1471-2156-14-20 (PMC3608943; doi:10.1186/1471-2156-14-20)
Supplement: Additional file 4 — Nucleotide diagnostic approach (ND) of all pairs of species that showed low K2P distance genetic values (<2%). [file 1471-2156-14-20-S4.docx]

**Additional file 4:** Nucleotide diagnostic approach (ND) of all pairs of species that showed low K2P distance genetic values (<2%). The positions in red show the partial NDs.

| **Species/position** | 207 | 225 | 276 | 337 | 366 | 570 | 571 | 582 |
| --- | --- | --- | --- | --- | --- | --- | --- | --- |
| *Astyanax fasciatus* | C | A | A | T | A | G | G | T |
| *Astyanax biotae* | T | G | G | C | G | C/T | A | C |

| **Species/position** | 285 | 342 | 597 |
| --- | --- | --- | --- |
| *Oligosarcus pintoi* | T | G | G |
| *Oligosarcus paranensis* | C | A | A |

| **Species/position** | 297 | 369 |
| --- | --- | --- |
| *Astyanax bockmanni* | T | A |
| *Astyanax paranae* | C | G |

| **Species/position** | 303 | 387 | 531 | 609 | 633 |
| --- | --- | --- | --- | --- | --- |
| *Characidium aff. Zebra* | C | C | T | G | G |
| *Characidium xanthopterum* | T | T | C | A | A |

| **Species/position** | 111 | 159 | 282 | 347 | 396 | 454 | 459 | 624 | 633 | 643 |
| --- | --- | --- | --- | --- | --- | --- | --- | --- | --- | --- |
| *Cichla kilberi* | C | C | C | T | G | T | T | T | C | G |
| *Cichla piquiti* | T | T | T | C | A | C | C | C | T | A |

| **Species/position** | 249 | 285 | 318 | 354 | 495 | 510 | 559 | 669 |
| --- | --- | --- | --- | --- | --- | --- | --- | --- |
| *Corumbataia britskii* | T | C | C | C | T | C | T | A |
| *Corumbataia cuestae* | C | A | T | T | C | T | G | G |

| **Species/position** | 129 | 144 | 264 | 399 | 423 | 465 | 477 | 510 | 534 | 588 | 597 |
| --- | --- | --- | --- | --- | --- | --- | --- | --- | --- | --- | --- |
| *Pimelodella meeki* | G | G | C | T | T | G | G | T | G | C | A |
| *Pimelodella sp1* | A | T | T | C | C | A | A | C | A | T | G |

| **Species/position** | 219 | 243 | 264 | 273 | 312 | 318 | 366 | 615 |
| --- | --- | --- | --- | --- | --- | --- | --- | --- |
| *Potamotrygon motoro* | T | A | A | T | T | A | C | C |
| *Potamotrygon falkineri* | C | G | G | C | C | C | T | T |

| **Species/position** | 156 | 264 | 288 | 339 | 405 | 453 | 576 |
| --- | --- | --- | --- | --- | --- | --- | --- |
| *Serrapinnus notomelas* | A | G | C | G | C | T | C |
| *Serrapinnus sp1* | G | A | T | A | T | C | T |

| **Species/position** | 246 | 408 | 531 | 576 | 606 | 636 |
| --- | --- | --- | --- | --- | --- | --- |
| *Steindachnerina brevipinna* | C | T | C | A | A | A |
| *Steindachnerina insculpta* | T | C | T | G | C | G |

| **Species/position** | 159 | 222 | 273 | 297 | 321 | 360 | 444 | 516 | 571 |
| --- | --- | --- | --- | --- | --- | --- | --- | --- | --- |
| *Astyanax bockmanni* | T | T | C | T | A | T | C | G | A |
| *Hyphessobrycon sp1* | C | C | T | C | G | C | T | A | G |

| **Species/position** | 246 | 261 | 279 | 315 | 318 | 432 | 456 |
| --- | --- | --- | --- | --- | --- | --- | --- |
| *Trichomycterus mimonha* | C | C | A | G | G | C | T |
| *Trichomycterus vermiculatus* | T | T | C | A | T | A | C |

| **Species/position** | 63 | 67 | 70 | 87 | 92 | 93 | 104 | 105 | 106 | 108 | 126 | 163 | 181 | 183 |
| --- | --- | --- | --- | --- | --- | --- | --- | --- | --- | --- | --- | --- | --- | --- |
| *Neoplecostomus Corumbá* | A | T | C | G | A | G | C | C | C | A | C | C | A | C |
| *Neoplecostomus paranensis* | . | C | . | . | . | A | . | . | . | . | . | . | . | . |
| *Neoplecostomus selenae* | . | C | . | . | . | A | . | . | . | . | . | . | . | . |
| *Neoplecostomus yapo* | . | C | . | . | . | A | . | A | . | . | . | . | . | . |
| *Neoplecostomus sp* | . | C | . | . | . | A | . | . | . | . | T | . | . | . |
| *Neoplecostomus sp1* | . | C | . | . | . | A | . | . | . | . | . | . | . | . |
| *Neoplecostomus sp2* | . | C | . | A | . | A | . | . | . | G | . | T | . | T |
| *Neoplecostomus sp3* | . | C | . | . | . | A | . | . | . | . | . | . | G | . |
| *Neoplecostomus sp4* | . | C | C/T | . | . | A | . | . | . | . | . | . | . | . |
| *Neoplecostomus sp5* | . | C | . | . | . | A/C | . | . | . | . | . | . | . | . |
| *Neoplecostomus langeani* | . | C | . | . | . | A | . | . | . | . | . | . | . | . |
| *Neoplecostomus bandeirante* | G | C | A | . | . | A | . | . | T | . | . | . | . | . |
| *Neoplecostomus sp8* | . | C | . | . | . | A | . | . | . | . | . | . | . | . |
| *Neoplecostomus sp9* | . | C | . | . | C | A | A | . | . | . | . | . | . | . |
| *Neoplecostomus sp10* | . | C/A | . | . | . | A | . | . | . | . | . | . | . | . |
| *Neoplecostomus botucatu* | . | C | . | . | . | A | . | . | . | . | . | . | . | . |

| 190 | 208 | 203 | 223 | 258 | 261 | 274 | 288 | 251 | 357 | 393 | 399 | 405 | 418 | 435 | 465 | 472 | 490 |
| --- | --- | --- | --- | --- | --- | --- | --- | --- | --- | --- | --- | --- | --- | --- | --- | --- | --- |
| G | C | T | G | C | C | C | C | T | C | T | A | C | C | A | C | A | A |
| . | . | . | A | . | T | T | . | C | T | . | G | . | . | . | T | . | . |
| . | . | . | A | . | . | T | . | C | T | . | G | . | . | . | T | . | . |
| . | . | . | A | . | . | T | . | C | T | . | . | . | . | G | T | . | G |
| . | T | . | A | . | . | T | . | C | T | . | . | . | . | . | T | . | . |
| . | . | G | A | . | . | T | . | C | T | . | G | . | . | . | T | . | . |
| . | . | . | A | T | . | T | A | C | T | . | . | . | . | . | T | . | . |
| . | . | . | A | . | . | T | . | C | T | . | . | . | . | . | T | . | . |
| . | . | . | A | . | . | T | T | C | T | . | . | . | . | . | T | . | . |
| . | . | . | A | . | . | T | . | C | T | C | G | . | . | . | T | . | . |
| . | . | . | A | . | . | T | . | C | T | . | G | . | . | . | T | . | . |
| . | . | . | A | . | . | T | . | C | T | . | G | . | . | . | T | . | . |
| . | . | . | A | . | . | T | . | C | T | . | C | T | . | . | T | . | . |
| C | . | . | A | . | . | T | . | C | T | . | G | . | G | . | T | C | . |
| . | . | . | A | . | . | T | . | C | T | . | G | . | . | . | T | . | . |
| . | . | . | A | . | . | T | . | C | T | . | G | . | . | . | T | . | . |

| 507 | 511 | 516 | 517 | 534 | 549 | 570 | 582 | 612 | 633 | 636 | 639 | 642 |
| --- | --- | --- | --- | --- | --- | --- | --- | --- | --- | --- | --- | --- |
| C | T | T | G | C | T | G | C | T | G | G | T | A |
| T | . | . | . | . | . | . | T | . | . | . | . | . |
| . | C | . | . | . | . | . | T | . | . | . | . | . |
| . | . | C | A | . | C | . | T | . | . | . | C/G | . |
| . | . | . | . | . | . | . | T | . | . | . | . | . |
| . | . | . | . | . | . | . | T | . | . | . | . | . |
| . | . | . | . | . | . | . | T | . | . | . | . | . |
| . | . | . | . | . | . | . | T | . | . | . | . | . |
| . | . | . | . | . | . | . | T | . | . | . | . | . |
| . | . | . | . | T | . | . | T | C | . | . | . | G |
| . | . | . | . | . | . | . | T | . | . | A | . | . |
| . | . | . | . | . | . | . | T | . | . | . | . | . |
| . | . | . | . | . | . | A | T | . | A | . | . | . |
| . | . | . | . | . | . | . | T | . | A | . | . | . |
| . | . | . | . | . | . | . | T | . | . | . | . | . |
| . | . | . | . | . | . | . | T | . | T | . | . | T |

| **Species/position** | 61 | 66 | 97 | 102 | 105 | 168 | 172 | 183 | 186 | 192 | 195 | 199 | 204 | 216 |
| --- | --- | --- | --- | --- | --- | --- | --- | --- | --- | --- | --- | --- | --- | --- |
| *Hypostomus topovae* | C | T | C | T | T | G | A | G | T | A | C | C | T/C | T |
| *Hypostomus heraldoi* | T | C | T | . | . | . | G | . | . | . | . | . | C | . |
| *Hypostomus paulinus* | T | . | . | C | . | . | . | A | C | . | T | . | C | C |
| *Hypostomus ancistroides* | T | . | . | . | C | . | . | . | C | . | . | . | T | . |
| *Hypostomus sp1* | T | . | . | . | . | A | . | . | . | . | . | . | C | . |
| *Hypostomus cochliodon* | T | . | . | . | . | . | . | . | C | G | . | T | T | . |
| *Hypostomus myersi* | T | . | . | . | . | . | . | . | . | . | . | . | A | . |
| *Hypostomus iheringi* | T | . | . | . | . | . | . | . | . | . | . | . | C | . |
| *Hypostomus commersoni* | T | . | . | . | . | . | . | . | C | . | . | . | T | . |
| *Hypostomus brevis* | T | . | . | . | . | . | . | . | C | . | . | . | T | . |
| *Hypostomus nigromaculatus* | T | . | . | . | . | . | . | . | . | . | . | . | C | . |
| *Hypostomus sp.* | T | . | . | . | . | . | . | . | . | . | . | . | C | . |
| *Hypostomus strigaticeps* | T | . | T | . | . | . | . | A/G | . | . | . | . | C | . |
| *Hypotomus albopunctatus* | T | . | T | . | . | . | G | . | . | G | . | . | C | . |
| *Hupostomus hermanni* | T | . | . | . | . | . | . | . | . | . | . | . | C | . |
| *Hypostomus regani* | T | . | . | . | . | . | . | . | . | . | . | . | C | . |
| *Hypostomus microstomus* | T | . | . | . | . | . | . | . | . | . | . | . | C | . |
| *Hypostomus derbyi* | T | . | . | . | . | . | . | . | C | . | . | . | T | . |

| 219 | 225 | 228 | 231 | 240 | 243 | 249 | 265 | 267 | 289 | 294 | 306 | 309 | 315 | 318 | 330 | 336 | 339 |
| --- | --- | --- | --- | --- | --- | --- | --- | --- | --- | --- | --- | --- | --- | --- | --- | --- | --- |
| A | C | T | G | A | A | T | C | G | T | A | A | T | A | G | T | A | A |
| . | . | . | . | . | G | . | . | A | . | G | G | . | G | . | . | T | . |
| . | . | . | . | . | . | . | . | A | . | . | . | . | . | . | . | T | G |
| . | . | . | . | . | . | . | T | A | . | . | . | C | G | A | . | T | . |
| G | . | . | . | . | . | . | . | . | . | . | . | . | . | . | . | T | . |
| . | . | C | . | . | . | C | . | A | . | G | G | . | . | . | . | T | . |
| . | . | . | . | . | . | . | . | . | . | . | . | . | . | . | . | T | . |
| . | . | . | . | . | . | . | . | . | . | . | . | . | . | . | C | T | . |
| . | . | . | . | . | . | . | T | A | . | . | . | C | G | A | . | T | . |
| . | . | . | A | G | . | . | T | A | C | . | . | C | G | A | . | T | . |
| . | T | . | . | . | . | . | . | A | . | . | . | . | . | . | . | T | . |
| . | . | . | . | . | . | . | . | . | . | . | . | . | . | . | . | T | . |
| . | . | . | . | . | G | . | . | A | . | . | G/T | . | G | . | . | T | . |
| . | . | . | . | . | G | . | . | A | . | G | G | . | G | . | . | T | . |
| . | . | . | . | . | . | . | . | A/G | . | . | G/A | . | . | . | . | T | . |
| . | . | . | . | . | . | . | . | . | . | . | . | . | . | . | . | T | . |
| . | . | . | . | . | . | . | . | . | . | . | . | . | . | A | . | T | . |
| . | . | . | . | . | . | . | T | A | . | . | G/A | C | G | A | . | T | A/G |

| 342 | 345 | 351 | 357 | 361 | 363 | 366 | 375 | 378 | 387 | 396 | 420 | 432 | 435 | 456 | 465 | 474 | 477 |
| --- | --- | --- | --- | --- | --- | --- | --- | --- | --- | --- | --- | --- | --- | --- | --- | --- | --- |
| T | A | C | A | C | A | C | A | C | C | C | T | A | A | C | C | C | A |
| . | . | . | . | . | T | . | . | T | . | T | C | . | . | T | . | T | . |
| . | . | . | G | . | . | T | . | T | . | . | . | . | . | . | . | . | . |
| . | . | . | . | T | . | T | G | T | . | . | . | . | . | T | T | . | . |
| C | . | . | . | . | . | . | . | . | . | . | . | . | . | . | . | . | . |
| . | . | . | . | T | . | . | . | T | T | . | . | G | G | T | T | . | . |
| . | . | . | . | . | . | . | . | . | . | . | . | . | . | . | . | . | . |
| . | . | . | . | . | . | T | . | T | . | . | . | . | . | . | . | . | . |
| . | . | . | . | T | . | T | G | T | . | . | . | . | . | A/T | T | . | T |
| . | . | . | . | T | . | T | A/G | T | . | . | . | . | . | T | T | . | . |
| . | . | . | . | . | . | . | . | . | . | . | . | . | . | . | T/C | . | . |
| . | . | . | . | . | . | . | . | . | . | . | . | . | . | . | . | . | . |
| . | T | T | A/G | . | T | . | . | T | . | T | C | . | . | T | . | . | . |
| . | . | T | . | . | T | . | . | T | . | T | C | . | . | T | . | T | . |
| . | . | . | . | . | . | . | . | . | . | . | . | . | . | . | . | . | . |
| . | . | . | . | . | . | . | . | . | . | . | . | . | . | . | . | . | . |
| . | . | . | . | . | . | T | . | . | . | . | . | . | . | . | . | . | . |
| . | . | . | . | T | . | T | G | T | . | . | . | . | . | . | T | . | . |

| 489 | 492 | 501 | 507 | 525 | 531 | 535 | 543 | 546 | 552 | 558 | 564 | 567 | 573 | 579 | 580 | 582 | 585 | 591 | 609 |
| --- | --- | --- | --- | --- | --- | --- | --- | --- | --- | --- | --- | --- | --- | --- | --- | --- | --- | --- | --- |
| A | C | T | T | C | T | T | G | C | A | T | T | T | C | G | T | G | A | C | C |
| . | T | . | . | . | . | . | A | . | . | . | C | . | T | . | C | . | . | . | . |
| . | T | . | . | . | . | . | . | . | . | . | C | . | T | . | . | . | . | T | . |
| . | T | . | . | T/C | . | C | . | . | . | C | C | . | T | . | . | A | . | . | . |
| . | T | . | . | . | . | . | . | . | . | . | . | . | . | . | . | . | C | . | . |
| G | T | . | . | . | . | C | . | . | . | . | C | . | T | A | . | . | . | . | . |
| . | T | . | . | . | . | . | . | . | . | . | . | . | . | . | . | . | T | . | T |
| . | . | . | C | . | . | . | . | . | . | . | C | . | T | . | . | . | . | . | . |
| . | T | . | . | . | . | C | . | . | . | C | C | C | T | . | . | A | . | . | . |
| . | . | . | . | . | C | C | . | T | . | C | C | C | T | . | . | A | . | . | . |
| . | T | . | . | . | . | . | . | . | . | . | . | C/T | . | A | . | . | T | . | . |
| . | T | . | . | . | . | . | . | . | T | . | . | . | . | . | . | . | . | . | . |
| . | T | C | . | T | . | . | A | . | . | . | C | . | T | . | . | . | . | . | . |
| . | T | . | . | . | . | . | A | . | . | . | C | . | T | . | C | . | . | . | . |
| . | T | . | . | . | . | . | . | . | . | . | . | . | . | . | . | . | . | . | . |
| . | T | . | . | T/C | . | . | . | . | . | . | . | . | . | . | . | . | T | . | . |
| . | T | C | . | . | . | . | A | . | . | . | . | . | . | . | . | . | . | . | . |
| . | T | . | . | . | . | C | . | . | . | C | C | C | T | . | . | A | . | . | . |
